# Synthetic strategy and antiviral evaluation of diamide containing heterocycles targeting dengue and yellow fever virus^☆^

**DOI:** 10.1016/j.ejmech.2016.05.043

**Published:** 2016-10-04

**Authors:** Milind Saudi, Joanna Zmurko, Suzanne Kaptein, Jef Rozenski, Bharat Gadakh, Patrick Chaltin, Arnaud Marchand, Johan Neyts, Arthur Van Aerschot

**Affiliations:** a KU Leuven, Rega Institute for Medical Research, Medicinal Chemistry, Minderbroedersstraat 10, 3000 Leuven, Belgium; b KU Leuven, Rega Institute for Medical Research, Laboratory of Virology and Chemotherapy, Minderbroedersstraat 10, 3000 Leuven, Belgium; c CISTIM Leuven vzw, Gaston Geenslaan 2, 3001 Leuven, Belgium; d Centre for Drug Design and Discovery (CD3), KU Leuven Research and Development, Waaistraat 6, 3000 Leuven, Belgium

**Keywords:** Flavivirus inhibitors, Dengue virus, Diamide, Ortho-phtalic acid, Pyrazine dicarboxylic acid

## Abstract

High-throughput screening of a subset of the CD3 chemical library (Centre for Drug Design and Discovery; KU Leuven) provided us with a lead compound **1**, displaying low micromolar potency against dengue virus and yellow fever virus. Within a project aimed at discovering new inhibitors of flaviviruses, substitution of its central imidazole ring led to synthesis of variably substituted pyrazine dicarboxylamides and phthalic diamides, which were evaluated in cell-based assays for cytotoxicity and antiviral activity against the dengue virus (DENV) and yellow fever virus (YFV). Fourteen compounds inhibited DENV replication (EC_50_ ranging between 0.5 and 3.4 μM), with compounds **6b** and **6d** being the most potent inhibitors (EC_50_ 0.5 μM) with selectivity indices (SI) > 235. Compound **7a** likewise exhibited anti-DENV activity with an EC_50_ of 0.5 μM and an SI of >235. In addition, good antiviral activity of seven compounds in the series was also noted against the YFV with EC_50_ values ranging between 0.4 and 3.3 μM, with compound **6n** being the most potent for this series with an EC_50_ 0.4 μM and a selectivity index of >34. Finally, reversal of one of the central amide bonds as in series **13** proved deleterious to the inhibitory activity.

## Highlights

• Two heterocyclic scaffold series were evaluated for *in vitro* Flavivirus inhibition.
• Activities at micromolar levels were noted for inhibition of dengue virus.
• Some remarkable inhibitory properties for yellow fever virus were recorded.
• Several pyrazine-2,3-dicarboxylic amides are endowed with anti-flavivirus activity.
• Phthalic acid diamides likewise provide an interesting scaffold for antivirals.

## Graphical abstract

**Figure:**
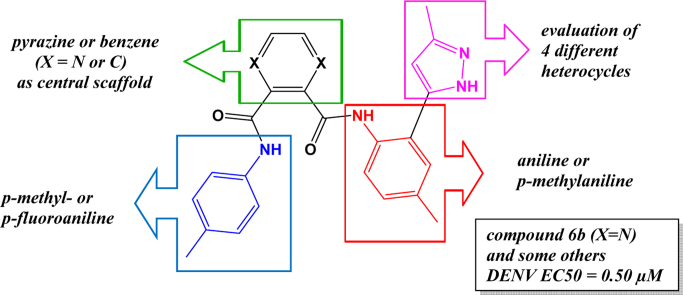

## 1 Introduction

The dengue virus (DENV), a mosquito-borne disease, is the most common arthropod-borne viral infection in the world [2]. Dengue incidence and prevalence are rising in endemic areas of the tropical and subtropical regions. On the basis of mathematical model estimates, approximately 390 million infections occur each year [2]. Dengue infection occurs in more than 100 countries in the Asia-Pacific, the Americas, the Middle East, and Africa, and cases continue to rise worldwide [3]. *Aedes aegypti* is the primary mosquito vector; however, other species from the genus *Aedes*, such as *Aedes albopictus*, can also be vectors of dengue virus transmission. The viruses have been grouped into four serotypes (DENV-1 to DENV-4) belonging to the genus *Flavivirus* (family Flaviviridae) with the existence of a fifth subtype being claimed more recently [4]. Each of these serotypes can cause disease symptoms ranging from self-limited febrile illness called dengue fever (DF) to dengue hemorrhagic fever (DHF), or dengue shock syndrome (DSS). Currently, there is no antiviral therapy available for DENV [5], [6].

Many different viral targets to inhibit or control dengue infection already have been envisaged over the last 10–15 years, among which the entry phase where the envelope glycoprotein is targeted by various strategies, or the NS3 helicase, the dengue protease, the RNA dependent RNA polymerase, the NS4b and the methyltransferase. Recently, a new series of 2-aroyl-3-arylquinoline was identified with strong inhibition of DENV2 RNA expression. Some compounds within this series inhibited DENV replication in both viral protein and mRNA levels, without significant cell cytotoxicity [7]. In parallel, a recent review discussed the potential of targeting non-structural proteins and more specifically proteases for combating neglected diseases as caused by arboviruses with in particular Dengue virus [8]. In addition, inhibition of host factors or processes indispensable for viral proliferation have been studied at several instances. An excellent review covering all aspects on the medicinal chemistry of dengue virus of Klein et al. recently appeared [9].

**Fig. 1:**
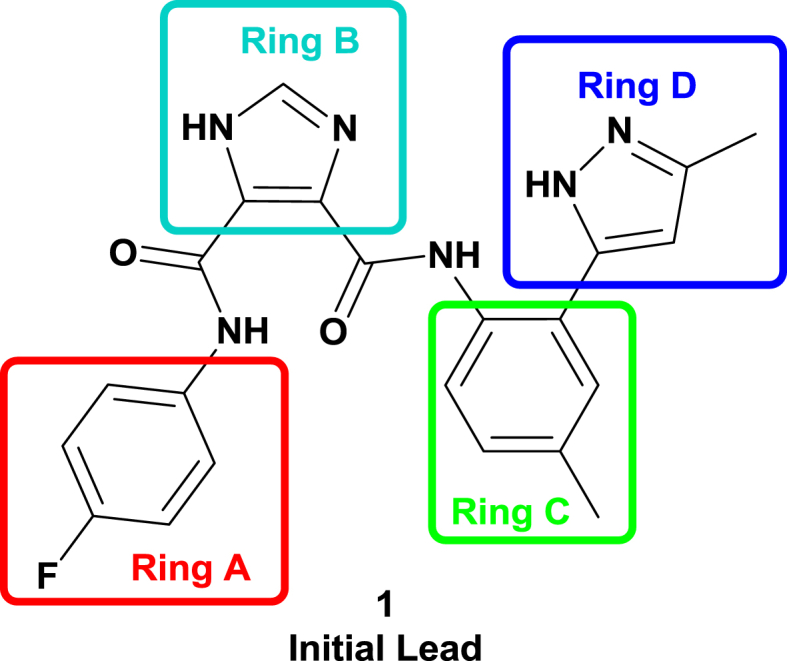
Structure of the initial lead compound endowed with antiviral properties (DENV: EC_50_ = 2.5 μM; YFV: EC_50_ = 3.5 μM).

**Fig. 2:**
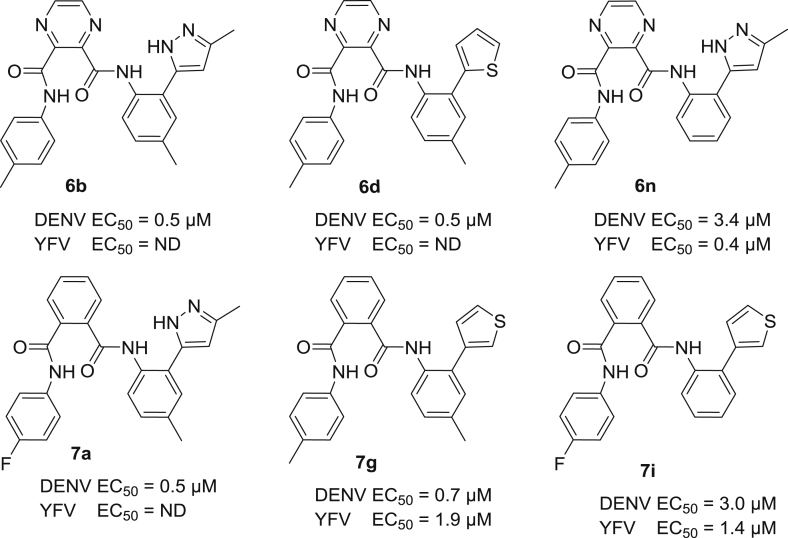
Overview of all new compounds most inhibitory to either DENV or YFV as reported in this communication.

In this communication we discuss our progress on the discovery of new flavivirus inhibitors. Hereto, at first a high-throughput screening (HTS) campaign was conducted with a subset of the CD3 chemical library (Centre for Drug Design and Discovery; KU Leuven) in order to identify novel non-nucleoside inhibitors of DENV (work supported by a Wellcome Trust Seeding Drug Discovery Award). This screening delivered an interesting hit compound **1** which turned to be active in low micromolar range against DENV and YFV (Fig. 1). Hence, a systematic modification program was setup examining substitutions at the four aromatic rings of the lead molecule. In a first report [1], the central imidazole ring B was retained resulting in a first series of analogues with modifications at the aniline moieties. The same paper discusses a second series where the central imidazole ring was substituted for a pyrazine 2,3-dicarboxylic acid moiety. Overall, these efforts allowed to uncover several inhibitors with activities in the low micromolar range. In this communication, we further elaborate on the central pyrazine scaffold, while a second series of compounds focuses on substitution of the original imidazole-4,5-dicarboxylic acid moiety by an ortho-phthalic acid central ring on which the different anilines are attached. The remaining three structural domains allowed for optimization of hit **1**: the aromatic moiety (ring A), the substituted aromatic moiety (ring C) and the heterocyclic group (ring D). These efforts resulted in several compounds with enhanced anti-DENV and anti-YFV inhibitory properties (see Fig. 2).

## 2 Results and discussion

### 2.1 Synthetic aspect

**Scheme 1:**
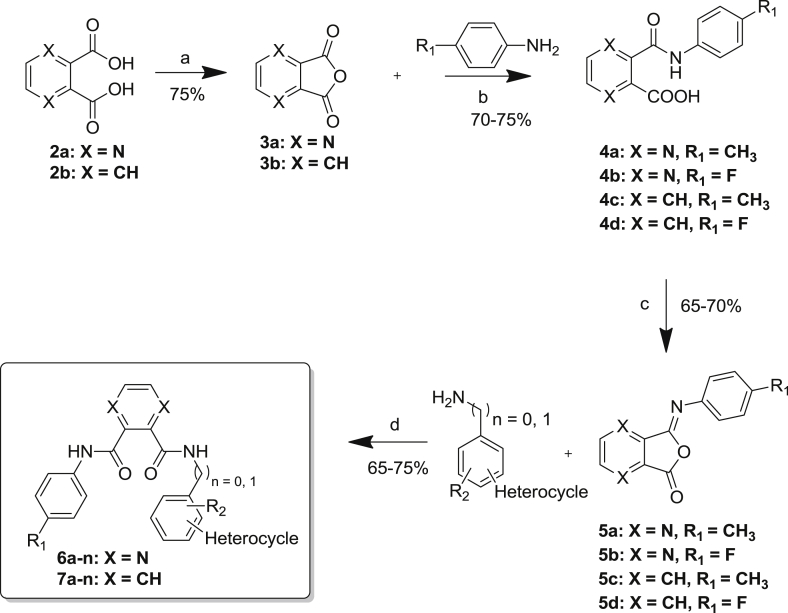
Reaction Conditions: a) acetic anhydride; b) acetonitrile: water (1:1), aniline, dodecyl hydrogen sulfate sodium salt, rt; c) trifluoroacetic anhydride, TEA, THF; d) aniline, THF, rt.

Pyrazines are important pharmacophores present in a number of biologically active compounds such as antimycobacterial, antibacterial, antidiabetic, and hypnotic agents [10], [11], [12]. Functionalization of the pyrazine started from commercial pyrazine-2,3-dicarboxylic acid and a four-step synthesis of the target compound is described in Scheme 1.

The commercially available pyrazine-2,3-dicarboxilic acid **2a** was allowed to reflux with acetic anhydride for 15 min. On cooling to 0 °C, pure anhydride **3a** was conveniently found to crystallize out which was collected by filtration, washed with ether and dried to obtain 85% yield. While benzoic anhydride can be directly added to an aqueous solution of the amine, the reaction times are longer and some benzoic anhydride remains unreacted, resulting into lower yields and an impure product. However, better results are obtained by adding acetonitrile to an equimolar amount of the respective anhydride and the appropriate aromatic amine dissolved in water containing sodium dodecyl sulfate (SDS) affording the *N*-arylphthalamic acids **4a,b**. The reaction was usually completed within 15 min. Removal of acetonitrile under reduced pressure led to precipitation of the benzoylated product along with benzoic acid. The desired product was further purified by column chromatography giving excellent yields (75–80%). Further, trifluoroacetic anhydride was added drop wise to a solution of the respective *N*-arylphthalamic acid and TEA in dioxane at 0 °C. The pale yellow intermediates **5a,b** resulting from simple dehydration of *N*-arylphthalamic acids were obtained by simply pouring the reaction mass into cold water. The precipitate formed was filtered and washed with water and the spectroscopic data were as could be expected. The analogous procedure starting from phthalic acid **2b** afforded the intermediates **5c,d**. Each of the intermediates **5a–d** were subsequently reacted with preformed heterocycle substituted anilines to give the target compounds **6a–n** and **7a–n** in 60–80% overall yields. All final products were purified by silica gel chromatography and were fully characterized.

**Scheme 2:**
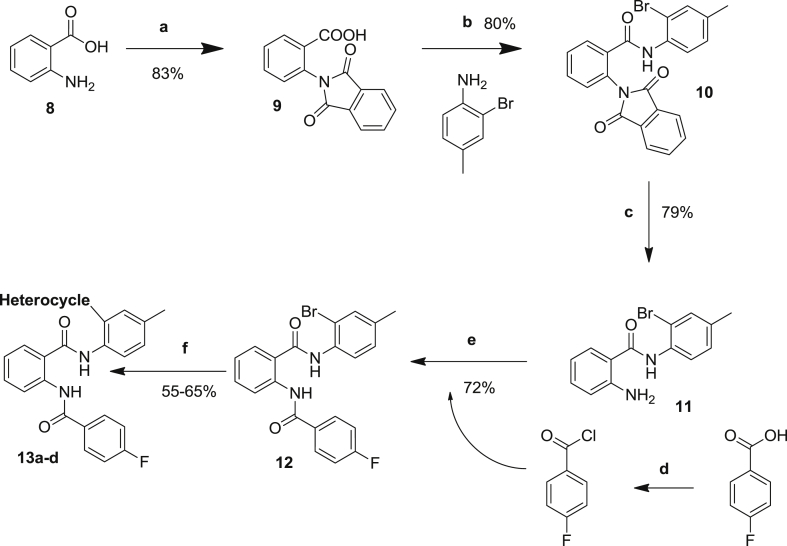
Reaction Conditions: a) phthalic anhydride, AcOH, reflux, 1 h, 83%; b) SOCl_2_, DMF(cat.), reflux, 1 h; aniline, DIPEA, CH_2_Cl_2_, rt, 1 h, 80%; c) hydrazine hydrate, THF, 60 C, 16 h; d) SOCl_2_, DMF(cat.), reflux, 2 h; e) DIPEA, CH_2_Cl_2_, rt, 2 h; f) K_2_CO_3_, water: dioxane (1:1), Pd(TPP)_2_Cl_2_, boronic acid.

To evaluate whether the orientation of the amide bonds for the lead compound were critical for biological activity, four compounds were synthesized having one of the amide bonds reversed. The 5-step synthesis of the target compound series **13a–d** is described in Scheme 2.

Hereto, anthranilic acid **8** was converted to its corresponding phthalimide derivative **9** simply by refluxing in presence of phthalic anhydride with acetic acid for 1 h. The obtained product was allowed to reflux with SOCl_2_ and catalytic DMF to afford the corresponding acid chloride. Anilines/amines subsequently provided straightforward coupling at reduced temperature resulting in **10**. Hydrazinolysis at 60 °C resulted in deprotection of the amine which was further coupled to another acid chloride to give target compound **12**. Coupling reactions of the latter with four different commercially available heterocyclic boronic acids afforded the desired novel molecules with general structure **13a–13d** in 60–70% yield. All final products were purified by silica gel chromatography and fully characterized. The obtained products were evaluated by the laboratory of virology for their inhibitory properties against dengue and yellow fever virus.

### 2.2 Assessment of antiviral activity

Based on the high throughput lead compound **1**, a total of 32 new analogues were synthesized and evaluated for their inhibitory properties against DENV (serotype 2) and YFV using Green monkey kidney cells [Vero-B cells (ECACC for DENV assays and ATCCCCL-81 for YFV assays)]. The 50% effective concentration (EC_50_), which is defined as the compound concentration that is required to inhibit the virus-induced CPE by 50%, and the 50% cytotoxic concentration (CC_50_), which is defined as the compound concentration that is required to inhibit the cell growth by 50%, was determined. Original data were obtained as μg/mL concentrations and were recalculated to μM concentrations as shown here. Compound **1** was included as a reference compound.

The initial lead compound **1** comprised the central imidazole ring B. In our efforts to understand SAR properties we replaced this imidazole ring (Fig. 1) with either a six-membered pyrazine (series **6**) or a phenyl ring (series **7**). Apart from a slightly different orientation of the attached substituents, we also expected this to provide us some information on the requirement for an imidazole ring B to interact with its target, or whether this 5-membered scaffold only serves to perfectly orient the pending substituents. While pyrazine preserves the polarity of the imidazole ring, the former only has H-bond accepting capacity. In contrast, the phenyl ring leads to increased lipophilicity. In addition, both six-membered rings give a slightly different 3D orientation for the attached aromatic moieties compared to the lead structure **1**. In another SAR study, we preserved the imidazole core and studied the effect of different aromatic substituents within this series of compounds [1]. As in the present study not all results could be obtained for inhibition of YFV, mainly the inhibitory properties for dengue infections are discussed more thoroughly below.

As to potential interactions of the original central imidazole ring, the pyrazine analogue **6a** of the lead structure **1** showed a 10-fold reduction in inhibitory properties against dengue virus (EC_50_ = 26.5 μM). In contrast however, its phenyl counterpart **7a** was 5-fold more inhibitory (EC_50_ = 0.5 μM) compared to lead compound **1**, and is endowed with a selectivity index (SI) of more than 235. Substituting the electron accepting fluorine for a hydrophobic methyl moiety increased the DENV inhibitory properties for **6b** 50-fold in comparison with **6a**, attaining likewise an EC_50_ of 0.5 μM with a SI above 235. Comparison of **6c** and **6d** or **6k** with **6l** confirmed this finding with a 10-fold increase in activity for the methyl congener, where for other analogous pairs of compounds this activity increase was less clear-cut (e.g. the couples **6g,6h**, **6i,6j**). In contrast, within series **7** with a central phenyl ring, the analogue **7c** carrying the *p*-tolyl moiety lost 60-fold in DENV inhibitory activity (EC_50_ = 29 μM) compared to **7a**, while for other compound pairs the para-methyl substituent at ring A again slightly outperformed the para-fluorine congener (e.g. **7e,7f** and **7g,7h**). The YFV inhibitory activity could not be determined for all compounds in view of the more demanding assay, and no analogous conclusions regarding the advantage of a *p*-tolyl moiety for ring A could be drawn (see Table 1, Table 2).

**Table 1:**
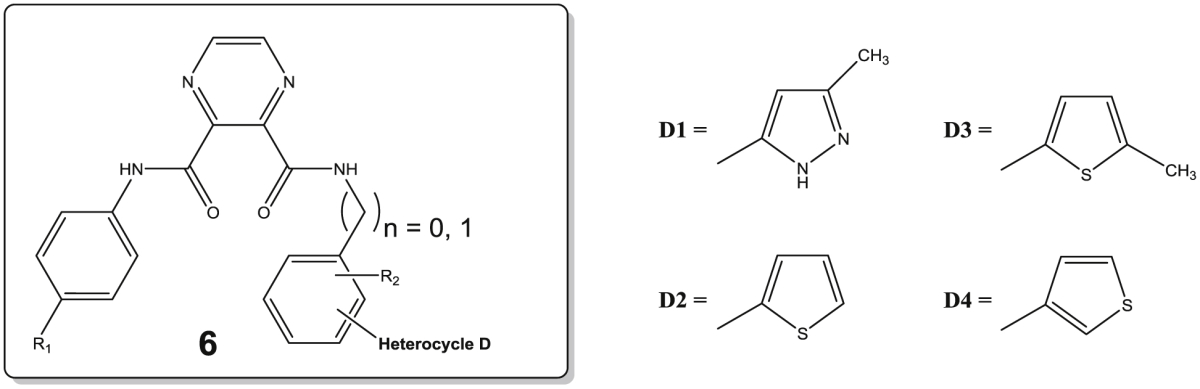
Antiviral activity of compounds **6a–6n**.

Sr. No.	n	R_1_	R_2_	Heterocycle (position^a^)	Dengue			Yellow fever		
					EC_50_ (μM)	CC_50_ (μM)	SI	EC_50_ (μM)	CC_50_ (μM)	SI
**6a**	0	F	*p*-methyl	D1 (ortho)	26.5	>116	>4	ND	ND	NA
**6b**	0	CH_3_	*p*-methyl	D1 (ortho)	0.5	>117	>235	ND	ND	NA
**6c**	0	F	*p*-methyl	D2 (ortho)	4.6	>116	>25	23	>116	5
**6d**	0	CH_3_	*p*-methyl	D2 (ortho)	0.5	>117	>235	ND	ND	NA
**6e**	0	F	*p*-methyl	D3 (ortho)	>112	>112	1	ND	ND	NA
**6f**	0	CH_3_	*p*-methyl	D3 (ortho)	>113	>113	1	ND	ND	NA
**6g**	0	F	*p*-methyl	D4 (ortho)	2.1	>116	>55	10.6	>116	>11
**6h**	0	CH_3_	*p*-methyl	D4 (ortho)	<0.9	26	>29	>116	>116	1
**6i**	1	F	H	D4 (ortho)	24	52	2	3.9	76	19
**6j**	1	CH_3_	H	D4 (ortho)	>48	48	1	5.4	75	14
**6k**	1	F	H	D4 (para)	>116	>116	1	18.5	>116	>6
**6l**	1	CH_3_	H	D4 (para)	14	>117	>8	21.5	>117	>5
**6m**	0	F	H	D1 (ortho)	2.2	>120	>55	7.8	77	10
**6n**	0	CH_3_	H	D1 (ortho)	3.4	24	7	0.4	>13.6	>34

a Indicates the attachment point of the heterocycle on the phenyl ring C.

**Table 2:**
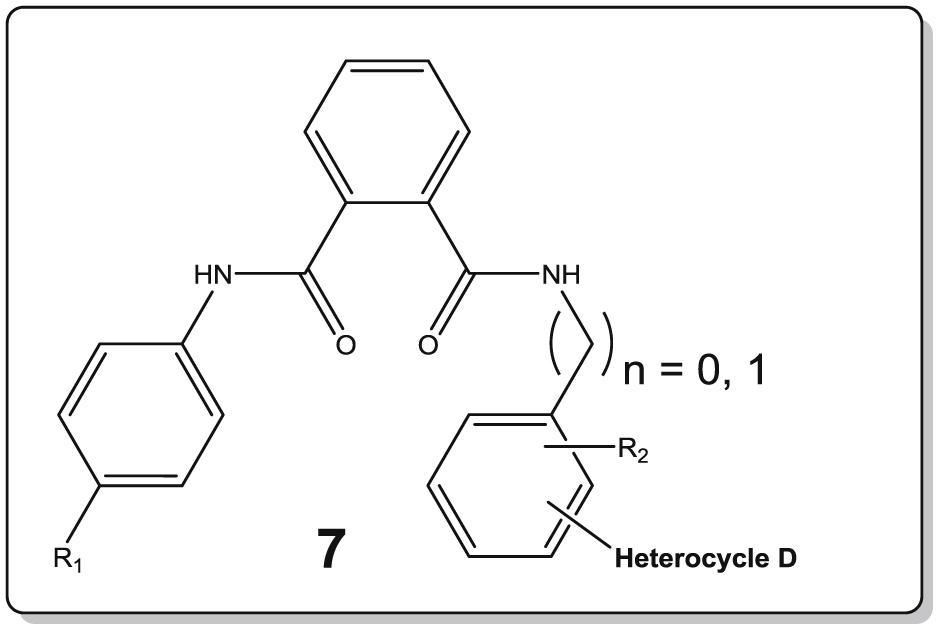
Antiviral activity of compounds **7a–7n**.

Sr. No.	n	R_1_	R_2_	Heterocycle (position^a^)	Dengue			Yellow fever		
					EC_50_ (μM)	CC_50_ (μM)	SI	EC_50_ (μM)	CC_50_ (μM)	SI
**7a**	0	F	*p*-methyl	D1 (ortho)	0.5	>117	>235	ND	ND	NA
**7b**	0	F	*p*-methyl	D2 (ortho)	4.0	>117	>29	23.2	>37	>1.6
**7c**	0	CH_3_	*p*-methyl	D1 (ortho)	29	>117	>4	ND	ND	NA
**7d**	0	CH_3_	*p*-methyl	D2 (ortho)	14	>117	>8	23.2	>37	>1.6
**7e**	0	CH_3_	*p*-methyl	D3 (ortho)	2.0	>114	>57	6.8	23	3.4
**7f**	0	F	*p*-methyl	D3 (ortho)	2.9	>113	>39	6.8	>113	>17
**7g**	0	CH_3_	*p*-methyl	D4 (ortho)	0.7	12.9	41	1.9	>117	>60
**7h**	0	F	*p*-methyl	D4 (ortho)	3.0	47	16	6.5	67	10
**7i**	1	F	H	D4 (ortho)	3.0	50	17	1.4	75	53
**7j**	1	CH_3_	H	D4 (ortho)	3.0	50	17	2.6	61	23
**7k**	1	F	H	D4 (para)	16	>116	>7	7.4	>117	>16
**7l**	1	CH_3_	H	D4 (para)	13.8	>117	>8	3.3	>117	>35
**7m**	0	F	H	D1 (ortho)	>120	>120	1	12.0	>120	>10
**7n**	0	CH_3_	H	D1 (ortho)	14.1	>122	>9	5.8	>122	>21

a indicates the attachment point of the heterocycle on the phenyl ring.

Next, taking a look at the effect of ring C and its various substitutions, we notice that within the pyrazine series **6**, using a benzyl moiety (n = 1, compounds **6i–6l**) instead of a (substituted) phenyl ring lead to strongly reduced inhibitory activity when comparing **6g** versus **6i** and **6h** versus **6j**. Removal of the para methyl substituent of ring C leads to conflicting conclusions within series **6**, with a 12-fold improvement for the *p*-fluorophenyl (ring A) compound **6m** over **6a**, but a 7-fold reduced inhibitory activity for the *p*-tolyl containing compound **6n** versus **6b**. For series **7** carrying a benzene central ring B, removal of the para-methyl moiety on ring C leading to **7m** was not well tolerated and led to strongly reduced inhibitory activity for DENV as compared to the initial lead compound **7a**. The negative effect of a benzyl substituent for ring C was less outspoken within series **7**, with DENV inhibitory activities varying from 3 to 16 μM for all compounds **7i–7l**. Likewise, strong inhibitory properties were noted against YFV for this series ranging from 1.4 to 7.4 μM.

The general trend observed for replacement of the five-membered pyrazole moiety (ring D) in the initial lead **1** with other heterocycles led to progressively inferior inhibition [1]. For evaluation of the present series with either a pyrazine (**6**) or a phenyl (**7**) moiety as the central core B, mainly the ortho position on ring C has been targeted to attach heterocyclic ring D as was found in the initial lead compound **1**. As well the original 3-methylpyrazol-5-yl, thien-2-yl and thien-3-yl ring displayed inhibitory activity with slight preference for the latter within both series **6** and **7**. This resulted in strong DENV inhibitory properties for compounds **6d** (EC_50_ = 0.5 μM) and **6h** (EC_50_ = <0.9 μM), resulting in selectivity indices (SI) of >235 and >29, respectively. Introduction of the 5-methylthien-2-yl moiety in contrast induced complete loss of DENV inhibitory activity only within series **6** (**6e** and **6f** in contrast to **7e** and **7f**). Within the latter series the 5-methylthien-2-yl moiety seems to be accommodated well affording DENV inhibitory activity at 2.0 and 2.9 μM, respectively, as well as strong YFV inhibition. Finally, a 3-thienyl at the para position of ring C proved less rewarding.

In addition, some analogues were synthesized comprising one reversed amide bond starting from anthranilic acid (Scheme 2) as displayed in Table 3. Unfortunately this modification mostly annihilated the inhibitory activity. Only compound **13b** displayed nice DENV inhibitory properties (EC_50_ = 3.2 μM), comparable in strength to its congener **7f** where the latter was based on the ortho-phthalic acid central scaffold of series **7**.

**Table 3:**
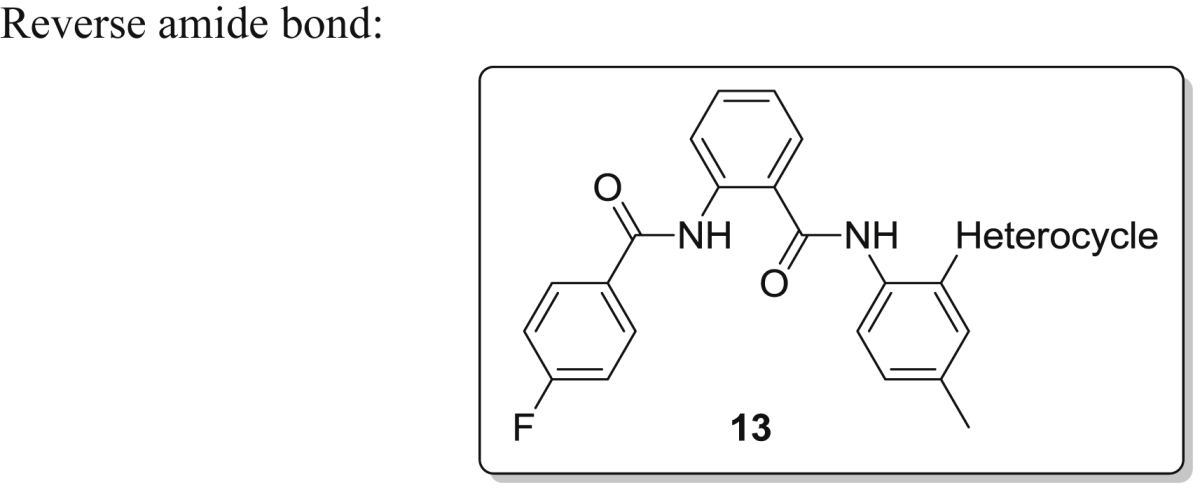
Antiviral activity of compounds **13a–13d** having a reversed amide bond.

Sr. No.	Heterocycle (position^a^)	Dengue			Yellow fever		
		EC_50_ (μM)	CC_50_ (μM)	SI	EC_50_ (μM)	CC_50_ (μM)	SI
**13a**	D4	>116	>116	NA	>116	>116	NA
**13b**	D3	3.2	>113	>35	>113	>113 (12.5 visual)	1
**13c**	Pyridin-4-yl	17	>117	>7	>117	>50 (30 visual)	1
**13d**	Pyridin-3-yl	23	>117	>5	>117	>50 (39 visual)	1

a Indicates the attachment point of the heterocycle on the phenyl ring.

Unfortunately, several attempts have been made to generate resistant viruses without success, and no clue so far has been obtained regarding the target of these polyaromatic compounds. Hence, all “rationalized” activities for the different modifications remain hypothetical, as no clear-cut structure-activity relationship can be worked out in absence of an interacting target.

Summarizing the antiviral results for all synthesized compounds, fourteen of them (**6b, 6d, 6g, 6h, 6m, 6n, 7a, 7e–j** and **13b**) exhibited selective activity against DENV in the low micromolar range (EC_50_ = 0.5–3.4 μM). In particular, derivatives **6b**, **6d** and **7g** turned out to be most potent against DENV (EC_50_ = 0.5 μM) and they were found to be non-cytotoxic at concentrations up to >116 μM resulting in selectivity indices above 235. As far as the activity against YFV is concerned, seven of the tested compounds (**6i, 6j, 6n, 7g, 7i, 7j** and **7l**) showed inhibition in the low micromolar range (EC_50_ = 0.4–3.3 μM). Among these derivatives, **6n** and **7i** were found to be most potent against YFV with an EC_50_ = 0.4 μg/mL and 1.4 μM, respectively.

## 3 Conclusion

We performed the synthesis and SAR of a series of phthalic diamide and pyrazine-2, 3-dicarboxamide derivatives for the inhibition of DENV and YFV. In total, 17 hits have been identified to inhibit either DENV or YFV during *in vitro* evaluation, five of which inhibit both viruses. Two of these molecules **6b** and **6d** displayed most interesting activities against DENV with EC_50_ = 0.5 μM and SI above 235. Compound **7a** likewise exhibited anti-DENV activity with an EC_50_ = 0.5 μM and a SI of >235. In addition, compound **6n** demonstrated strong inhibition of YFV growth with an EC_50_ = 0.4 μM and a selectivity index of >34. Reversal of one of the central amide bonds proved deleterious to the inhibitory activity.

## 4 Materials and methods

### 4.1 General procedure for synthesis of **4a–d**

[13] Sodium dodecyl sulfate (SDS, 6 mmol) was added to a stirred heterogeneous suspension of amine (5 mmol) in water (20 mL) until a homogeneous solution was formed, (in case of turbidity, the mixture was warmed to obtain a clear solution). Phthalic anhydride (5 mmol) dissolved in acetonitrile (5 mL) was added to this solution in one lot. After stirring for 1 h at room temperature, the acetonitrile was evaporated and the product precipitated from the aqueous layer. To the aqueous solution containing precipitate, solid sodium hydrogen carbonate was added pinch-wise until the effervescence ceased and the pH reached 6.0. The remaining precipitated product was filtered, washed with water (20 mL), and dried in a vacuum desiccator. In cases where the product did not precipitate, the reaction mixture was extracted with ethyl acetate (2 × 25 mL). The combined organic extracts were dried with anhydrous Na_2_SO_4_ and the solvent was removed in a rotary evaporator under reduced pressure to yield the pure product, as solids.

#### 4.1.1 3-(*p*-tolylcarbamoyl)pyrazine-2-carboxylic acid **(4a)**

Yield: 75%; ^1^H NMR (300 MHz, DMSO-*d*_6_) δ: 10.68 (bs, 1H, COOH), 8.89 (s, 2H), 7.66 (d, 2H, *J* = 8.4 Hz), 7.18 (d, 2H, *J* = 8.4 Hz), 2.29 (s, 3H, CH_3_); ^13^C NMR (75 MHz, DMSO-*d*_6_) δ: 166.5, 162.3, 146.5, 145.8, 145.6, 144.5, 136.0, 133.4, 129.3 (2xC), 120.2 (2xC), 20.7; HRMS calcd. for C_13_H_10_N_3_O_3_ [M-H]^−^: 256.0728; found: 256.0743.

#### 4.1.2 3-((4-fluorophenyl)carbamoyl)pyrazine-2-carboxylic acid **(4b)**

Yield: 70%; ^1^H NMR (300 MHz, DMSO-*d*_6_) δ: 10.86 (bs, 1H, COOH), 8.90 (s, 2H), 7.83–7.79 (m, 2H), 7.25–7.19 (m, 2H); ^13^C NMR (75 MHz, DMSO-*d*_6_) δ: 165.9 (br), 164.1, 160.0, 156.8, 144.7 (br), 143.8 (br), 135.5 (J_C,F_ = 6 Hz), 121.6 (2xC, J_C,F_ = 30 Hz), 115.5 (2xC, J_C,F_ = 87 Hz) + missing quaternary signal; HRMS calcd. For C_12_H_7_N_3_O_3_F_1_ [M-H]^-^: 260.0477; found: 260.0480.

#### 4.1.3 2-(*p*-tolylcarbamoyl)benzoic acid **(4c)**

Yield: 72%; ^1^H NMR (300 MHz, DMSO-*d*_6_) δ: 10.69 (bs, 1H, COOH), 9.08 (s, 1H), 8.89 (s, 2H), 7.69 (d, 2H, *J* = 8.4 Hz), 7.36 (s, 1H), 7.18 (d, 2H, *J* = 8.4 Hz), 2.29 (s, 3H, CH_3_); ^13^C NMR (75 MHz, DMSO-*d*_6_) δ: 166.5, 162.3, 149.0, 145.7, 144.5, 136.0, 133.4, 130.0, 129.6 (2xC), 129.3, 127.3 (2xC), 120.2, 20.6; HRMS calcd. for C_15_H_14_N_1_O_3_ [M+H]^+^: 256.0968; found: 256.0970.

#### 4.1.4 2-((4-fluorophenyl)carbamoyl)benzoic acid **(4d)**

Yield: 71%; ^1^H NMR (300 MHz, DMSO-*d*_6_) δ: 13.05 (bs, 1H, COOH), 10.38 (bs, 1H, NH), 7.90–7.21 (m, 6H), 7.18 (t, 2H, *J* = 9.0 Hz); ^13^C NMR (75 MHz, DMSO-*d*_6_) δ: 167.4, 167.3, 159.7, 156.5, 138.8, 136.0, 131.7, 129.9, 129.5, 127.8, 121.3, 121.2, 115.3, 115.1; HRMS calcd. for C_14_H_9_N_1_O_3_F_1_ [M-H]^−^: 258.0572; found: 258.0571.

### 4.2 General procedure for synthesis of **5a–d**

[14]Trifluoroacetic anhydride (1.5 equiv) was added dropwise to a stirring solution of acid **4a–d** (1 equiv) and Et_3_N (TEA, 3 equiv) in 1,4-dioxane (20 mL) that was kept at 0 °C with an ice bath. After 15 min the yellow solution was allowed to warm to room temperature and was stirred for 30 min, then it was poured in cold H_2_O (100 mL). A yellowish precipitate formed, which was collected by filtration using a Buchner funnel and washed with H_2_O. The product was dried overnight under high vacuum.

#### 4.2.1 (Z)-7-(*p*-tolylimino)furo [3,4-b]pyrazin-5(7H)-one **(5a)**

Yield: 70%; ^1^H NMR (300 MHz, CDCl_3_) δ: 9.08–9.03 (m, 2H), 7.63 (d, 2H, *J* = 8.1 Hz), 7.29 (d, 2H, *J* = 6.0 Hz), 2.43 (s, 3H, CH_3_); ^13^C NMR (75 MHz, CDCl_3_) δ: 150.5, 149.0, 129.5, 126.4, 21.0. + missing quaternary C signals; HRMS calcd. for C_13_H_10_N_3_O_2_ [M+H]^+^: 240.0766; found: 240.0766.

#### 4.2.2 (Z)-7-((4-fluorophenyl)imino)furo [3,4-b]pyrazin-5(7H)-one **(5b)**

Yield: 68%; ^1^H NMR (300 MHz, CDCl_3_) δ: 9.09–9.05 (m, 2H), 7.76–7.47 (m, 2H), 7.28–7.14 (m, 2H). ^13^C NMR (75 MHz, CDCl_3_) δ: 150.9, 149.5, 128.7, 128.6, 116.2, 115.9 + missing quaternary C signals; HRMS calcd. for C_12_H_7_N_3_O_2_F_1_ [M+H]^+^: 244.0517; found: 244.0508.

#### 4.2.3 (Z)-3-(*p*-tolylimino)isobenzofuran-1(3H)-one **(5c)**

Yield: 65%; ^1^H NMR (300 MHz, CDCl_3_) δ: 8.11–7.98 (m, 2H), 7.86–7.76 (m, 2H), 7.42 (d, 2H, *J* = 8.4 Hz), 7.23 (d, 2H, *J* = 8.4 Hz), 2.40 (s, 3H, CH_3_); ^13^C NMR (75 MHz, DMSO-*d*_6_) δ: 164.8, 147.3, 141.6, 136.5, 136.0, 135.5, 133.7, 129.6, 129.1, 127.6, 125.4, 124.1, 123.4, 20.8 + missing C=N-Ph signal; HRMS calcd. for C_15_H_12_N_1_O_2_ [M+H]^+^: 238.0862; found: 238.0843.

#### 4.2.4 (Z)-3-((4-fluorophenyl)imino)isobenzofuran-1(3H)-one **(5d)**

Yield: 67%; ^1^H NMR (300 MHz, DMSO-*d*_6_) δ: 8.13–7.88 (m, 4H), 7.42–7.30 (m, 2H), 7.30–7.22 (m, 2H); ^13^C NMR (75 MHz, DMSO-*d*_6_) δ: 164.7, 161.8, 158.5, 140.9, 136.3, 136.1, 134.0, 127.9, 125.9, 125.8, 125.5, 123.5, 116.0, 115.7; HRMS calcd. for C_14_H_9_N_1_O_2_F_1_ [M+H]^+^: 242.0612; found: 242.0615.

#### 4.2.5 General procedure for synthesis of **6a–n** and **7a–n**

[14] Phthalisoimide **5a–d** (1 equiv) was added to a stirring solution of the aniline/benzylamine (1.2 equiv) in THF. The mixture was stirred overnight at room temperature. Two alternative work-up procedures were followed depending on whether the product precipitated out of solution or not.

*Work-up 1 (for soluble products)*: The THF was evaporated on a rotavapor. The mixture was diluted with EtOAc (using the triple volume of THF) and washed three times with 1 M HCl. The organic phase was dried with Na_2_SO_4_ and evaporated. The product was purified by flash chromatography on silica gel using a MeOH gradient in DCM and affording a greyish precipitate following evaporation.

*Work-up 2 (for insoluble products)*: The precipitate was collected by filtration using a Buchner funnel and washed on the frit with small volumes of 1:1 Et_2_O/n-hexane until the yellow impurities had disappeared. The solid product obtained from this work-up procedure did not need further chromatographic purification.

#### 4.2.6 *N*^2^-(4-fluorophenyl)-*N*^3^-(4-methyl-2-(3-methyl-1H-pyrazol-5-yl)phenyl) pyrazine-2,3 dicarboxamide **(6a)**

Yield: 72%; ^1^H NMR (300 MHz, DMSO-*d*_6_) δ: 13.10 (bs, 1H), 13.00 (bs, 1H), 10.62 (bs, 1H), 8.97–8.90 (bs, 2H), 8.50 (d, 1H, *J* = 8.1 Hz), 7.70–7.57 (m, 3H), 7.25–7.11 (m, 3H), 6.58 (bs, 1H), 2.33 (s, 6H, 2CH_3_); ^13^C NMR (150 MHz, CDCl_3_) δ: 207.0, 163.1, 161.3, 160.5, 158.9, 149.6, 149.2, 144.7, 143.3, 133.6, 133.4, 129.3, 128.4, 121.8, 121.8, 121.7, 121.6, 121.1, 116.4, 115.8, 103.4, 21.0, 11.0; HRMS calcd. for C_23_H_20_F_1_N_6_O_2_ [M+H]^+^: 431.1626; found: 431.1620.

#### 4.2.7 *N*^2^-(4-methyl-2-(3-methyl-1H-pyrazol-5-yl)phenyl)-*N*^3^-(*p*-tolyl)pyrazine-2,3-dicarboxamide **(6b)**

Yield: 65%; ^1^H NMR (600 MHz, CDCl_3_) δ: 11.93 (bs, 0.6H), 9.12 (bs, 0.7H), 8.66–8.59 (bs, 2H), 7.60–7.13 (m, 7H), 6.36 (s, 1H), 2.37 (s, 3H, CH_3_), 2.32 (s, 3H, CH_3_), 2.28 (s, 3H, CH_3_); ^13^C NMR (150 MHz, CDCl_3_) δ: 207.0, 163.2, 161.1, 151.3, 149.2, 145.6, 144.6, 143.2, 142.0, 140.3, 134.9, 134.4, 133.4, 129.6, 129.5, 128.3, 121.5, 121.1, 120.0, 119.9, 103.2, 21.0, 20.9, 10.9; HRMS calcd. for C_24_H_22_N_6_O_2_Na_1_ [M+Na]^+^: 449.1697; found: 449.1697.

#### 4.2.8 *N*^2^-(4-fluorophenyl)-*N*^3^-(4-methyl-2-(thiophen-2-yl)phenyl)pyrazine-2,3-dicarboxamide **(6c)**

Yield: 73%; ^1^H NMR (600 MHz, CDCl_3_) δ: 8.99 (bs, 0.7H, NH), 8.92 (bs, 0.8H, NH), 8.64–8.59 (m, 2H), 8.36 (d, 1H, *J* = 4.2 Hz), 7.70–7.68 (m, 2H), 7.48–7.42 (m, 2H) + coinciding CHCl_3_ peak, 7.27–7.15 (m, 3H), 7.09–7.03 (m, 2H), 2.37 (s, 3H, CH_3_); ^13^C NMR (150 MHz, CDCl_3_) δ: 161.7, 149.6, 146.9, 144.2, 144.1, 138.2, 134.7, 133.4, 131.8, 130.5, 129.1, 128.7, 128.3, 128.3, 127.5, 126.6, 123.8121.9, 121.7, 116.4, 115.8, 115.7, 20.9; HRMS calcd. for C_23_H_18_F_1_N_4_O_2_S_1_ [M+H]^+^: 433.1129; found: 433.1133.

#### 4.2.9 *N*^2^-(4-methyl-2-(thiophen-2-yl)phenyl)-*N*^3^-(*p*-tolyl)pyrazine-2,3-dicarboxamide **(6d)**

Yield: 78%; ^1^H NMR (600 MHz, CDCl_3_) δ: 8.95 (bs, 1H, NH), 8.78 (bs, 1H, NH), 8.64–8.59 (m, 2H), 8.38 (d, 1H, *J* = 4.2 Hz), 7.60 (d, 2H, *J* = 3.9 Hz), 7.47 (bs, 1H), 7.42–7.38 (m, 1H), 7.28–7.15 (m, 5H), 2.37 (s, 3H, CH_3_), 2.34 (s, 3H, CH_3_); ^13^C NMR (150 MHz, CDCl_3_) δ: 162.0, 161.3, 147.3, 146.4, 144.2, 143.9, 138.3, 134.8, 134.5, 134.5, 131.9, 130.4, 129.5 (2xC), 129.0, 128.7, 127.4, 126.5, 123.8, 121.9, 120.1, 120.0, 20.9 (2xC); HRMS calcd. for C_24_H_21_N_4_O_2_S_1_ [M+H]^+^: 429.1380; found: 449.1376.

#### 4.2.10 *N*^2^-(4-fluorophenyl)-*N*^3^-(4-methyl-2-(5-methylthiophen-2-yl)phenyl)pyrazine-2,3-dicarboxamide **(6e)**

Yield: 69%; ^1^H NMR (600 MHz, CDCl_3_) δ: 9.19 (bs, 0.7H, NH), 8.91 (bs, 0.7H, NH), 8.73–8.63 (m, 2H), 8.45–8.38 (m, 1H), 7.71–7.69 (m, 2H), 7.23–7.14 (m, 2H), 7.09–7.03 (m, 3H), 6.75 (bs, 1H), 2.51 (s, 3H, CH_3_), 2.36 (s, 3H, CH_3_); ^13^C NMR (150 MHz, CDCl_3_) δ: 161.7, 160.3, 158.7, 149.6, 146.5, 144.2, 144.1, 141.2, 136.9, 134.6, 133.2, 131.1, 129.3, 128.0, 127.0, 125.9, 125.0, 122.0, 121.8, 116.4, 115.9, 115.7, 20.9, 15.5; HRMS calcd. for C_24_H_20_F_1_N_4_O_2_S_1_ [M+H]^+^: 447.1285; found: 447.1283.

#### 4.2.11 *N*^2^-(4-methyl-2-(5-methylthiophen-2-yl)phenyl)-*N*^3^-(*p*-tolyl)pyrazine-2,3-dicarboxamide **(6f)**

Yield: 75%; ^1^H NMR (600 MHz, CDCl_3_) δ: 9.07 (bs, 0.7H, NH), 8.89 (bs, 0.7H, NH), 8.67–8.65 (m, 2H), 8.42–8.40 (m, 1H), 7.61 (d, 2H, *J* = 3.9 Hz), 7.25–7.12 (m, 4H), 7.07 (d, 1H), 6.74 (bs, 1H), 2.50 (s, 3H, CH_3_), 2.36 (s, 3H, CH_3_), 2.34 (s, 3H, CH_3_); ^13^C NMR (150 MHz, CDCl_3_) δ: 161.9, 161.4, 147.3, 146.6, 144.2, 143.9, 141.1, 137.0, 134.8, 134.5, 131.8, 131.0, 129.6 (2xC), 129.3, 127.0, 125.9, 125.4, 121.9, 121.8, 120.2, 120.1, 21.0, 20.9, 15.3; HRMS calcd. for C_25_H_21_N_4_O_2_S_1_ [M-H]^−^: 441.1391; found: 441.1367.

#### 4.2.12 *N*^2^-(4-fluorophenyl)-*N*^3^-(4-methyl-2-(thiophen-3-yl)phenyl)pyrazine-2,3- dicarboxamide **(6g)**

Yield: 64%; ^1^H NMR (500 MHz, CDCl_3_) δ: 8.95 (bs, 1H, NH), 8.89 (bs, 1H, NH), 8.65 (bs, 1H), 8.62 (bs, 1H), 8.38 (d, 1H, *J* = 7.0 Hz), 7.72–7.69 (m, 2H), 7.46–7.42 (m, 2H), 7.30–7.16 (m, 3H), 7.08–7.05 (t, 2H, *J* = 7.25 Hz), 2.37 (s, 3H, CH_3_); ^13^C NMR (125 MHz, CDCl_3_) δ: 161.8, 161.6, 160.5, 158.9, 147.1, 146.4, 144.3, 144.0, 138.2, 134.7, 133.4, 131.8, 130.5, 129.1, 127.5, 126.6, 123.8, 122.0, 121.9, 121.8, 115.8, 115.7, 20.9; HRMS calcd. for C_23_H_18_F_1_N_4_O_2_S_1_ [M+H]^+^: 433.1129; found: 433.1121.

#### 4.2.13 *N*^2^-(4-methyl-2-(thiophen-3-yl)phenyl)-*N*^3^-(*p*-tolyl)pyrazine-2,3-dicarboxamide **(6h)**

Yield: 69%; ^1^H NMR (500 MHz, CDCl_3_) δ: 8.94 (bs, 1H, NH), 8.76 (bs, 1H, NH), 8.66 (s, 1H), 8.63 (s, 1H), 8.39 (d, 1H, *J* = 7.0 Hz), 7.62–7.61 (d, 2H, *J* = 7.0 Hz), 7.48 (bs, 1H), 7.42–7.41 (m, 1H), 7.28–7.27 (d, 1H, *J* = 4.0 Hz), 7.22–7.17 (m, 4H), 2.37 (s, 3H, CH_3_), 2.34 (s, 3H, CH_3_); ^13^C NMR (125 MHz, CDCl_3_) δ: 162.1, 161.3, 149.0, 147.4, 146.3, 144.3, 143.8, 138.3, 134.8, 134.6, 131.9, 130.4, 129.6 (2xC), 129.0, 128.8, 127.4, 126.5, 123.8, 121.8, 120.1 (2xC), 21.0, 20.9; HRMS calcd. for C_24_H_21_N_4_O_2_S_1_ [M+H]^+^: 429.1380; found: 429.1366.

#### 4.2.14 *N*^2^-(4-fluorophenyl)-*N*^3^-(2-(thiophen-3-yl)benzyl)pyrazine-2,3-dicarboxamide **(6i)**

Yield: 68%; ^1^H NMR (300 MHz, DMSO-*d*_6_) δ: 10.67 (bs, 1H, NH), 9.44–9.24 (dt, 1H, NH rotamers), 8.91–8.87 (m, 2H), 7.79–7.18 (m, 10H), 4.50 (d, 2H, *J* = 6.0 Hz, CH_2_); ^13^C NMR (75 MHz, DMSO-*d*_6_) δ: 164.2, 163.5, 160.0, 156.8, 148.1, 147.6, 146.1, 145.3, 144.9, 144.5, 140.4, 137.4, 136.0, 135.3, 132.4, 129.7, 129.1, 129.0, 128.5, 127.7, 127.4, 127.0, 126.2, 123.8, 122.1, 121.6, 121.6, 121.5, 121.5, 115.6, 115.3, 42.9 (many double peaks as of rotamers); HRMS calcd. for C_23_H_18_F_1_N_4_O_2_S_1_ [M+H]^+^: 433.1129; found: 433.1121.

#### 4.2.15 *N*^2^-(2-(thiophen-3-yl)benzyl)-*N*^3^-(*p*-tolyl)pyrazine-2,3-dicarboxamide **(6j)**

Yield: 65%; ^1^H NMR (300 MHz, DMSO-*d*_6_) δ: 10.65 (bs, 1H, NH), 9.39 (t, 1H), 9.24 (t, 1H), 8.91–8.86 (m, 2H), 7.67–7.15 (m, 10H), 4.50 (d, 2H, *J* = 6.0 Hz, CH_2_), 2.29 (s, 3H, CH_3_); ^13^C NMR (75 MHz, DMSO-*d*_6_) δ: 164.4, 163.4, 148.3, 147.8, 146.2, 145.2, 144.9, 144.6, 140.5, 137.5, 136.5, 135.1, 132.9, 132.4, 129.7, 129.3, 129.1, 128.6, 127.8, 127.5, 127.0, 126.3, 123.9, 122.2, 119.8, 42.9, 20.6; HRMS calcd. for C_24_H_21_N_4_O_2_S_1_ [M+H]^+^: 429.1380; found: 429.1380.

#### 4.2.16 *N*^2^-(4-fluorophenyl)-*N*^3^-(4-(thiophen-3-yl)benzyl)pyrazine-2,3-dicarboxamide **(6k)**

Yield: 72%; white solid; ^1^H NMR (300 MHz, DMSO-*d*_6_) δ: 10.65 (bs, 1H, NH), 9.34 (t, 1H, *J* = 5.3 Hz), 8.89–8.87 (m, 2H), 7.85–7.54 (m, 7H), 7.40 (d, 2H, *J* = 8.1 Hz), 7.21 (t, 2H, *J* = 8.5 Hz), 4.50 (d, 2H, *J* = 6.3 Hz, CH_2_); ^13^C NMR (75 MHz, DMSO-*d*_6_) δ: 164.0, 163.7, 160.1, 156.9, 148.2, 145.5, 145.2, 144.5, 141.4, 138.1, 135.5, 133.9, 128.0 (2xC), 127.2, 126.3, 126.1 (2xC), 121.7, 121.6, 120.8, 115.7, 115.4, 42.2; HRMS calcd. for C_23_H_18_F_1_N_4_O_2_S_1_ [M+H]^+^: 433.1129; found: 433.1122.

#### 4.2.17 *N*^2^-(4-(thiophen-3-yl)benzyl)-*N*^3^-(*p*-tolyl)pyrazine-2,3-dicarboxamide **(6l)**

Yield: 75%; white solid; ^1^H NMR (300 MHz, DMSO-*d*_6_) δ: 10.49 (bs, 1H, NH), 9.36 (t, 1H, *J* = 5.3 Hz), 8.88–8.85 (m, 2H), 7.85–7.84 (m, 1H), 7.68–7.54 (m, 6H), 7.40 (d, 2H, *J* = 8.1 Hz), 7.17 (d, 2H, *J* = 8.4 Hz), 4.49 (d, 2H, *J* = 6.3 Hz), 2.29 (s, 3H, CH_3_); ^13^C NMR (75 MHz, DMSO-*d*_6_) δ: 164.0, 163.6, 148.4, 145.6, 145.1, 144.4, 141.4, 138.1, 136.6, 133.9, 132.8, 129.2 (2xC), 128.0 (2xC), 127.1, 126.3, 126.1 (2xC), 120.8, 119.8, 119.7, 42.2, 20.7; HRMS calcd. for C_24_H_21_N_4_O_2_S_1_ [M+H]^+^: 429.1380; found: 429.1375.

#### 4.2.18 *N*^2^-(4-fluorophenyl)-*N*^3^-(2-(3-methyl-1H-pyrazol-5-yl)phenyl)pyrazine-2,3-dicarboxamide **(6m)**

Yield: 65%; white solid; ^1^H NMR (300 MHz, DMSO-*d*_6_) δ: 13.20 (bs, 1H, NH), 13.00 (bs, 1H, NH), 10.64 (bs, 1H, NH), 8.98–8.91 (m, 2H), 8.62–8.60 (m, 1H), 7.76–7.70 (m, 3H), 7.33–7.15 (m, 4H), 6.60 (s, 1H), 2.32 (s, 3H, CH_3_); ^13^C NMR (75 MHz,, DMSO-*d*_6_) δ: 164.1, 161.7, 159.4, 157.4, 150.2, 149.7, 149.1, 146.1, 144.1, 143.7, 139.9, 135.3, 128.0, 127.9, 124.1, 121.8, 121.4, 120.3, 115.6, 115.5, 102.7, 10.4; HRMS calcd. for C_22_H_16_F_1_N_6_O_2_ [M-H]^-^: 415.1324; found: 415.1308.

#### 4.2.19 *N*^2^-(2-(3-methyl-1H-pyrazol-5-yl)phenyl)-*N*^3^-(*p*-tolyl)pyrazine-2,3-di-carboxamide **(6n)**

Yield: 62%; white solid; ^1^H NMR (300 MHz,, DMSO-*d*_6_) δ: 13.17 (bs, 1H, NH), 13.00 (bs, 1H, NH), 10.48 (bs, 1H, NH), 8.97–8.90 (m, 2H), 8.62–8.60 (m, 1H), 7.76–7.74 (m, 1H), 7.57 (d, 2H, *J* = 8.5 Hz), 7.30–7.16 (m, 4H), 6.59 (s, 1H), 2.32 (s, 3H, CH_3_), 2.29 (s, 3H, CH_3_); ^13^C NMR (75 MHz,, DMSO-*d*_6_) δ: 164.0, 161.7, 150.2, 149.9, 146.1, 144.0, 143.8, 139.9, 136.6, 135.3, 132.8, 129.3 (2xC), 128.0, 127.9, 124.1, 121.8, 120.3, 119.6 (2xC), 102.7, 20.6, 10.4; HRMS calcd. for C_23_H_21_N_6_O_2_ [M+H]^+^: 413.1720; found: 413.1727.

#### 4.2.20 *N*^1^-(4-fluorophenyl)-*N*^*2*^-(4-methyl-2-(3-methyl-1H-pyrazol-5-yl)phenyl)phthal-amide **(7a)**

Yield: 65%; ^1^H NMR (600 MHz, DMSO-*d*_6_) δ: 12.90 (bs, 1H), 12.27 (bs, 1H), 10.50 (bs, 1H, NH), 10.44 (s, 1H), 8.46 (d, 1H, *J* = 8.4 Hz), 7.83 (m, 1H), 7.75–7.53 (m, 6H), 7.17–7.08 (m, 3H), 6.61 (s, 1H), 2.32 (s, 3H, CH_3_), 2.28 (s, 3H, CH_3_); ^13^C NMR (150 MHz, DMSO-*d*_6_) δ: 166.7, 166.5, 165.7, 159.0, 157.4, 150.7, 139.7, 137.6, 136.7, 136.4, 135.9, 133.9, 132.3, 130.4, 130.1, 128.4, 128.3, 127.2, 121.5 (2xC), 120.5, 119.8, 115.3 (2xC), 102.6, 20.6, 10.4; HRMS calcd. for C_25_H_20_F_1_N_4_O_2_ [M-H]^-^: 427.1576; found: 427.1577.

#### 4.2.21 *N*^1^-(4-fluorophenyl)-*N*^2^-(4-methyl-2-(thiophen-2-yl)phenyl)phthalamide **(7b)**

Yield: 68%; ^1^H NMR (600 MHz, DMSO-*d*_6_) δ: 10.43 (s, 0.4H), 9.78 (s, 1H), 9.12 (bs, 0.4H), 8.12–7.00 (m, 13H), 7.68–7.52 (m, 1H), 2.50 (s, 3H, CH_3_); ^13^C NMR (150 MHz, DMSO-*d*_6_) δ: 167.7, 167.6, 165.0, 155.5, 145.5, 141.0, 139.7, 137.3–115.1 (complex aromatic pattern), 22.5; HRMS calcd. for C_25_H_20_F_1_N_2_O_2_S_1_ [M+H]^+^: 431.1223; found: 431.1221.

#### 4.2.22 *N*^1^-(4-methyl-2-(3-methyl-1H-pyrazol-5-yl)phenyl)-*N*^2^-(*p*-tolyl)phthalamide **(7c)**

Yield: 65%; ^1^H NMR (600 MHz, DMSO-*d*_6_) δ: 12.89 (bs, 0.7H), 12.24 (bs, 0.7H), 10.31 (s, 1H), 8.46 (bs, 0.7H), 8.03–7.52 (m, 7H), 7.23–7.00 (m, 3H), 6.73–6.68 (m, 1H), 2.33 (s, 3H, CH_3_), 2.26 (s, 3H, CH_3_), 2.18 (s, 3H, CH_3_); ^13^C NMR (150 MHz, DMSO-*d*_6_) δ: 170.1, 166.9, 165.9, 139.5, 137.8, 137.0, 136.5, 133.9, 132.3, 130.3, 130.0, 129.7, 129.0 (2xC), 128.4, 128.0, 127.9, 127.1, 119.7 (2xC), 115.7, 102.6, 22.1, 21.2, 14.1; HRMS calcd. for C_26_H_25_N_4_O_2_ [M+H]^+^: 425.1972; found: 425.1971.

#### 4.2.23 *N*^1^-(4-methyl-2-(thiophen-2-yl)phenyl)-*N*^2^-(*p*-tolyl)phthalamide **(7d)**

Yield: 69%; ^1^H NMR (500 MHz, DMSO-*d*_6_) δ: 13.05 (bs, 1H), 8.89 (bs, 0.6H), 7.85 (d, 1H), 7.78–6.95 (m, 11H), 6.75–6.55 (m, 2H), 2.23 (s, 3H, CH_3_), 2.15 (s, 3H, CH_3_); ^13^C NMR (150 MHz, DMSO-*d*_6_) δ: 167.6 (2xC), 154.5, 146.0, 141.1, 137.4, 137.2, 132.1, 131.1, 130.7, 130.6, 130.2, 129.4, 129.1 (2xC), 128.6, 128.3 (2xc), 125.3, 123.8, 122.7, 122.4, 120.1, 119.6, 20.9, 20.8; HRMS calcd. for C_26_H_23_N_2_O_2_S_1_ [M+H]^+^: 427.1475; found: 427.1471.

#### 4.2.24 *N*^1^-(4-methyl-2-(5-methylthiophen-2-yl)phenyl)-*N*^2^-(*p*-tolyl)phthalamide **(7e)**

Yield: 78%; ^1^H NMR (600 MHz, DMSO-*d*_6_) δ: 10.66 (bs, 1H), 10.26 (s, 1H), 9.09 (s, 1H), 8.92 (d, 1H), 8.89 (d, 1H), 7.76–7.73 (m, 3H), 7.32 (d, 1H), 7.25–7.17 (m, 5H), 6.82 (d, 1H), 2.51 (s, coinciding with DMSO-d_5_, CH_3_), 2.45 (s, 3H, CH_3_), 2.33 (s, 3H, CH_3_); ^13^C NMR (150 MHz, DMSO-*d*_6_) δ: 163.8, 163.4, 162.3, 159.2, 157.7, 149.0, 147.8, 145.5, 144.8, 144.6, 140.2, 136.9, 135.4, 131.2, 129.9, 128.6, 128.3, 126.8, 126.3, 125.5, 121.7 (2xC), 115.6 (2xC), 20.6 (2xC), 15.0; HRMS calcd. for C_27_H_24_N_2_O_2_S_1_Na_1_ [M+Na]^+^: 463.1451; found: 463.1450.

#### 4.2.25 *N*^1^-(4-fluorophenyl)-*N*^2^-(4-methyl-2-(5-methylthiophen-2-yl)phenyl)phthalamide **(7f)**

Yield: 65%; ^1^H NMR (500 MHz, DMSO-*d*_6_) δ: 10.40 (s, 1H, NH), 9.87 (s, 1H, NH), 7.74–7.05 (m, 12H), 6.79 (d, 1H), 2.45 (s, 3H, CH_3_), 2.32 (s, 3H, CH_3_); ^13^C NMR (150 MHz, DMSO-*d*_6_) δ: 167.2, 167.0, 159.0, 157.3, 139.7, 137.6, 137.0, 136.2, 136.0, 132.8, 131.7, 130.5, 129.9, 129.4, 128.7, 128.4, 128.0, 127.5, 126.3, 125.9, 121.4 (2xC), 115.3 (2xC), 115.2, 20.6, 15.0; HRMS calcd. for C_26_H_21_F_1_N_2_O_2_S_1_Na_1_ [M+Na]^+^: 467.1200; found: 467.1198.

#### 4.2.26 *N*^1^-(4-methyl-2-(thiophen-3-yl)phenyl)-*N*^2^-(*p*-tolyl)phthalamide **(7g)**

Yield: 68%; ^1^H NMR (500 MHz, CDCl_3_) δ: 8.85 (bs, 1H, NH), 8.08 (d, 1H, *J* = 7.8 Hz), 7.84 (d, 1H, *J* = 6 Hz), 7.80 (m, 1H), 7.53–7.15 (m, 12H), 2.37 (s, 3H, CH_3_), 2.34 (s, 3H, CH_3_); ^13^C NMR (125 MHz, CDCl_3_) δ: 168.1, 165.8, 138.2, 135.4, 135.2, 134.2, 131.8, 130.7, 130.5, 129.7 (multiple), 129.0, 128.7, 128.2, 127.2, 126.8, 123.7 (2xC), 122.7, 120.2 (2xC), 20.9 (2xC); HRMS calcd. for C_26_H_22_N_2_O_2_S_1_Na_1_ [M+Na]^+^: 449.1294; found: 449.1289.

#### 4.2.27 *N*^1^-(4-fluorophenyl)-*N*^2^-(4-methyl-2-(thiophen-3-yl)phenyl)phthalamide **(7h)**

Yield: 64%; ^1^H NMR (500 MHz, CDCl_3_) δ: 9.30 (bs, 1H, NH), 8.07 (d, 1H, *J* = 7.0 Hz), 7.80 (d, 2H, *J* = 7.0 Hz), 7.60 (m, 2H), 7.50–7.35 (m, 3H), 7.30 (m, 2H), 7.20–7.12 (m, 3H), 7.00–6.94 (m, 2H), 2.36 (s, 3H, CH_3_); ^13^C NMR (125 MHz, CDCl_3_) δ: 168.2, 165.9, 160.2, 158.6, 156.0, 138.2, 135.3, 135.1, 134.2, 131.8, 130.7, 130.5, 129.0, 128.7, 128.2, 127.0 (2xc), 123.7, 122.6, 121.9, 121.8, 115.6, 115.4, 20.9; HRMS calcd. for C_25_H_19_F_1_N_2_O_2_S_1_Na_1_ [M+Na]^+^: 453.1044; found: 453.1038.

#### 4.2.28 *N*^1^-(4-fluorophenyl)-*N*^2^-(2-(thiophen-3-yl)benzyl)phthalamide **(7i)**

Yield: 74%; ^1^H NMR (500 MHz, DMSO-*d*_6_) δ: 10.41 (bs, 1H, NH), 9.00–8.83 (dt, 1H, NH rotamers), 7.75–7.14 (m, 15H), 4.44 (m, 2H); ^13^C NMR (75 MHz, DMSO-*d*_6_) δ: 168.1, 167.1, 159.1, 157.2, 140.6, 137.7, 137.1, 137.0, 136.5, 136.0, 135.8, 135.3, 132.3, 130.0, 129.7, 129.6, 129.1, 128.9, 128.8, 127.9, 127.7, 127.5, 126.9, 126.2, 123.8, 122.1, 121.3 (2xC), 115.5, 115.3, 43.1 (many double peaks as of rotamers); HRMS calcd. for C_25_H_19_F_1_N_2_O_2_S_1_Na_1_ [M+Na]^+^: 453.1044; found: 453.1046.

#### 4.2.29 *N*^1^-(2-(thiophen-3-yl)benzyl)-*N*^2^-(*p*-tolyl)phthalamide **(7j)**

Yield: 68%; ^1^H NMR (300 MHz, DMSO-*d*_6_) δ: 10.27 (bs, 1H, NH), 8.98–8.81 (m, 1H), 7.69–7.50 (m, 9H), 7.30–7.08 (m, 6H), 4.44 (d, 2H, *J* = 5.7 Hz), 2.28 (s, 3H, CH_3_); ^13^C NMR (75 MHz,, DMSO-*d*_6_) δ: 168.3, 167.0, 140.6, 137.8, 137.1, 136.5, 136.2, 135.9, 135.4, 132.3, 129.9, 129.6, 129.1, 128.9, 128.8, 128.0, 127.9, 127.8, 127.5, 126.9, 126.2, 123.8, 122.2, 119.7, 43.2, 20.6; HRMS calcd. for C_26_H_21_N_2_O_2_S_1_ [M-H]^-^: 425.1329; found: 425.1333.

#### 4.2.30 *N*^1^-(4-fluorophenyl)-*N*^2^-(4-(thiophen-3-yl)benzyl)phthalamide **(7k)**

Yield: 69%; ^1^H NMR (500 MHz, DMSO-*d*_6_) δ: 10.39 (bs, 1H, NH), 8.94 (t, 1H, *J* = 5.0 Hz), 7.80 (d, 1H), 7.73–7.68 (m, 2H), 7.65–7.50 (m, 8H), 7.36 (d, 2H, *J* = 7 Hz), 7.16 (t, 2H, *J* = 7.5 Hz), 4.42 (d, 2H, *J* = 5.0 Hz); ^13^C NMR (125 MHz, DMSO-*d*_6_) δ: 168.2, 167.5, 159.3, 157.7, 141.7, 138.7, 137.5, 136.4, 134.0, 130.2 (2xC), 130.0, 128.2 (2xC), 127.5, 126.5 (2xC), 121.7, 121.6 (2xC), 115.7, 115.5, 42.4; HRMS calcd. for C_25_H_19_F_1_N_2_O_2_S_1_Na_1_ [M+Na]^+^: 453.1044; found: 453.1044.

#### 4.2.31 *N*^1^-(4-(thiophen-3-yl)benzyl)-*N*^2^-(*p*-tolyl)phthalamide **(7l)**

Yield: 61%; ^1^H NMR (300 MHz, DMSO-*d*_6_) δ: 10.25 (bs, 1H, NH), 8.90 (t, 1H, *J* = 6.2 Hz), 7.82 (dd, 1H, *J* = 2.7 Hz and 1.2 Hz), 7.70–7.45 (m, 10H), 7.38 (d, 2H, *J* = 8 Hz), 7.13 (d, 2H, *J* = 8.4 Hz), 4.44 (d, 2H, *J* = 6.0 Hz), 2.27 (s, 3H, CH_3_); ^13^C NMR (75 MHz, DMSO-*d*_6_) δ: 168.0, 167.0, 141.4, 138.4, 137.2, 136.1, 133.7, 132.3, 129.8, 129.6, 129.1 (2xC), 127.9 (2xc),127.1, 126.2, 126.0 (2xC), 120.7 (2xC), 119.7, 42.4, 20.6; HRMS calcd. for C_26_H_22_N_2_O_2_S_1_Na_1_ [M+Na]^+^: 449.1294; found: 449.1288.

#### 4.2.32 *N*^1^-(4-fluorophenyl)-*N*^2^-(2-(3-methyl-1H-pyrazol-5-yl)phenyl)phthalamide **(7m)**

Yield: 72%; ^1^H NMR (500 MHz, DMSO-*d*_6_) δ: 12.94 (bs, 1H, NH), 12.40 (bs, 1H, NH), 10.51 (bs, 1H, NH), 8.58 (d, 1H, *J* = 6.5 Hz), 7.85–7.60 (m, 8H), 7.29 (t, 1H, *J* = 6.5 Hz), 7.20–7.10 (m, 3H), 6.64 (s, 1H), 2.29 (s, 3H, CH_3_); ^13^C NMR (125 MHz, DMSO-*d*_6_) δ: 167.1, 166.1, 159.0, 157.4, 150.7, 139.9, 137.6, 136.3, 135.9, 130.5, 130.2, 128.5, 127.9 (2xC), 127.3, 123.5, 121.4 (2xC), 120.6, 119.8, 115.4, 115.2, 102.6, 10.4; HRMS calcd. for C_24_H_19_F_1_N_4_O_2_Na_1_ [M+Na]^+^: 437.1384; found: 437.1379.

#### 4.2.33 *N*^1^-(2-(3-methyl-1H-pyrazol-5-yl)phenyl)-*N*^2^-(*p*-tolyl)phthalamide **(7n)**

Yield: 61%; ^1^H NMR (500 MHz, DMSO-*d*_6_) δ: 12.94 (bs, 1H, NH), 12.37 (bs, 1H, NH), 10.34 (bs, 1H, NH), 8.60 (d, 1H, *J* = 5.0 Hz), 7.82 (t, 1H), 7.77 (dd, 1H), 7.68–7.62 (m, 3H), 7.54 (d, 2H, *J* = 7 Hz), 7.28 (t, 1H, *J* = 6.5 Hz), 7.15–7.05 (m, 2H), 6.64 (s, 1H), 2.28 (s, 3H, CH_3_), 2.26 (s, 3H, CH_3_); ^13^C NMR (125 MHz, DMSO-*d*_6_) δ: 166.9, 166.2, 150.7, 139.9, 138.0, 137.1, 136.6, 136.4, 132.4, 130.5, 130.1, 129.1 (2xC), 128.5, 128.0, 127.2, 123.5, 120.5, 119.8, 119.7 (2xC), 102.7, 20.6, 10.4; HRMS calcd. for C_25_H_23_N_4_O_2_ [M+H]^+^: 411.1815; found: 411.1811.

### 4.3 2-(1,3-dioxoisoindolin-2-yl)benzoic acid **(9)**

A mixture of anthranilic acid (1.0 mmol), phthalic anhydride (1.1 mmol) and acetic acid was refluxed for 1 h. After cooling, the precipitated product was filtered and washed with water. The obtained product was dried thoroughly *in vacuo*.

Yield: 83%; white solid; ^1^H NMR (300 MHz, DMSO-*d*_6_) δ: 11.53 (bs, 1H, COOH), 8.62 (d, 1H, *J* = 8.1 Hz), 8.03 (d, 1H, *J* = 7.8 Hz), 7.87 (d, 1H, *J* = 7.5 Hz), 7.70–7.60 (m, 4H), 7.21 (t, 1H, *J* = 7.2 Hz); ^13^C NMR (75 MHz, DMSO-*d*_6_) δ: 169.8, 167.7, 167.1, 141.2, 138.2, 134.3, 132.0, 131.3, 130.6, 130.3, 129.8, 127.4, 123.1, 120.0, 116.6; HRMS calcd. for C_15_H_8_N_1_O_4_ [M-H]^-^: 266.0459; found: 266.0463.

### 4.4 *N*-(2-bromo-4-methylphenyl)-2-(1,3-dioxoisoindolin-2-yl)benzamide **(10)**

To a solution of **9** (1 mmol) in dry DCM (5 mL), SOCl_2_ (1.5 mmol) and catalytic DMF were added to obtain a clear solution. The reaction mixture was allowed to reflux for 1.5 h. The reaction mixture was evaporated to dryness and dried for 1 h. The reaction mixture was rediluted with DCM and cooled to 0 °C. DIPEA and 2-bromotoludine were added and the reaction was allowed to stir at rt. for 2 h after which the mixture was concentrated to dryness. The oily residue was purified by silica gel column chromatography to give pure compound **10** (80%) as a white solid.

^1^H NMR (300 MHz, CDCl_3_) δ: 8.20 (bs, 2H), 7.95–7.35 (m, 9H), 7.08–7.05 (dd, 1H), 2.30 (s, 3H, CH_3_); ^13^C NMR (75 MHz, DMSO-*d*_6_) δ: 167.0 (2xC), 165.1, 137.9, 134.9, 134.8 (2xC), 133.4, 132.8, 131.8, 131.4, 130.3, 129.8, 128.9, 128.7, 128.3, 127.6, 123.6, 123.5 (2xC),120.4, 20.2 (some double peaks as of rotamers); HRMS calcd. for C_22_H_16_Br_1_N_2_O_3_ [M+H]^+^: 435.0339; found: 435.0344.

### 4.5 2-amino-*N*-(2-bromo-4-methylphenyl)benzamide **(11)**[15]

To a solution of **10** (1.0 mmol) in anhydrous THF (5 mL), was added hydrazine monohydrate (1.1 mmol) at rt. and the mixture was allowed to stir at 60 °C for 16 h. The reaction mixture was evaporated to dryness and the oily residue was purified by silica gel column chromatography to give pure compound **12** as a white solid.

Yield 79%; ^1^H NMR (300 MHz, DMSO-*d*_6_) δ: 9.66 (bs, 1H, NH), 7.72 (d, 1H, *J* = 8 Hz), 7.53 (d, 1H, *J* = 1.2 Hz), 7.41 (d, 1H, *J* = 7.8 Hz), 7.23–7.18 (m, 2H), 6.75 (d, 1H, *J* = 8.1 Hz), 6.57 (t, 1H, *J* = 8.1 Hz), 6.43 (bs, 2H, NH_2_), 2.33 (s, 3H, CH_3_); HRMS calcd. for C_14_H_14_Br_1_N_2_O_1_ [M+H]^+^: 305.0284; found: 305.0295.

### 4.6 *N*-(2-bromo-4-methylphenyl)-2-(4-fluorobenzamido)benzamide **(12)**

To a solution of **11** (1.0 mmol) and DIPEA (1.5 mmol) in dry DCM (5 mL), was added 4-fluorobenzoyl chloride (obtained from the acid with thionyl chloride) at 0 °C. The reaction mixture was allowed to stir at rt. for 2 h. The reaction mixture was evaporated to dryness and the oily residue was purified by silica gel column chromatography to afford title compound **12** as a white solid.

Yield 72%; ^1^H NMR (500 MHz, CDCl_3_) δ: 12.15 (bs, 1H, NH), 8.90 (dd, 2H, *J* = 8 Hz), 8.60 (dd, 2H, *J* = 8.1 Hz), 8.38 (d, 1H, *J* = 7 Hz), 7.75–7.68 (m, 2H), 7.43 (dd, 2H, *J* = 4 Hz and *J* = 2.5 Hz), 7.22–7.15 (m, 2H), 7.10–7.03 (m, 2H), 2.35 (s, 3H, CH_3_); ^13^C NMR (125 MHz, CDCl_3_) δ: 161.6 (2xC), 160.5, 158.9, 147.1, 146,4, 144.2, 144.0, 138.2, 134.7, 133.4, 131.8, 130.5, 129.0, 128.7, 127.5, 126.6, 123.8, 122.0, 121.8, 115.9, 115.7, 20.9; HRMS calcd. for C_21_H_17_Br_1_F_1_N_2_O_2_ [M+H]^+^: 427.0452; found: 427.0445.

### 4.7 General procedure for synthesis of **13a–d**

To a solution of **12** (1.0 mmol) in 5 mL dioxane: water (1:1), was added anhydrous K_2_CO_3_ (1.5 mmol), the respective aryl boronic acid (1.2 mmol) and Pd(TPP)_2_Cl_2_ (0.03 mmol). The mixture was heated at 100 °C for 16 h. All reactions and manipulations were run under argon atmosphere. After completion, the solvent was evaporated under reduced pressure and the residue was purified by column chromatography on silica gel to afford the desired product.

#### 4.7.1 2-(4-fluorobenzamido)-*N*-(4-methyl-2-(thiophen-3-yl)phenyl)benzamide **(13a)**

Yield: 62%; ^1^H NMR (300 MHz, CDCl_3_) δ: 12.15 (bs, 1H, NH), 8.83 (d, 1H, *J* = 8.4 Hz), 8.19–8.05 (m, 4H), 7.58–7.06 (m, 10H), 2.41 (s, 3H, CH_3_); ^13^C NMR (75 MHz, CDCl_3_) δ: 166.9, 166.4, 164.2, 163.1, 140.1, 138.0, 134.9, 132.8, 131.4, 130.4, 129.6, 129.5, 128.8, 128.3, 127.9, 126.9, 125.9, 123.4, 122.8, 122.1, 121.5, 120.1, 115.6, 115.3, 20.6; HRMS calcd. for C_25_H_18_F_1_N_2_O_2_S_1_ [M-H]^−^: 429.1078; found: 429.1082.

#### 4.7.2 2-(4-fluorobenzamido)-*N*-(4-methyl-2-(5-methylthiophen-2-yl)phenyl)benzamide **(13b)**

Yield: 65%; ^1^H NMR (300 MHz, CDCl_3_) δ: 12.09 (bs, 1H, NH), 8.84 (d, 1H, *J* = 8.7 Hz), 8.32 (bs, 1H, NH), 8.14–8.05 (m, 3H), 7.60–7.46 (m, 2H), 7.28–7.10 (m, 5H), 6.95 (d, 1H, J = 3.6 Hz), 6.75 (m, 1H), 2.52 (s, 3H, CH_3_), 2.40 (s, 3H, CH_3_); ^13^C NMR (75 MHz, CDCl_3_) δ: 167.1, 166.4, 164.2, 163.0, 141.3, 140.1, 136.3, 134.9, 132.8, 131.3, 130.9, 129.6, 129.5, 129.0, 126.6, 126.4, 126.2, 125.7, 122.8, 122.5, 121.5, 120.3, 115.6, 115.3, 20.6, 15.0; HRMS calcd. for C_26_H_20_F_1_N_2_O_2_S_1_ [M-H]^−^: 443.1235; found: 443.1229.

#### 4.7.3 2-(4-fluorobenzamido)-*N*-(4-methyl-2-(pyridin-4-yl)phenyl)benzamide **(13c)**

Yield: 59%; ^1^H NMR (300 MHz, CDCl_3_) δ: 11.95 (bs, 1H, NH), 8.79 (d, 1H, *J* = 8.7 Hz), 8.68 (d, 2H, *J* = 5.1 Hz), 8.06–7.90 (m, 4H), 7.57–7.52 (m, 1H), 7.38–7.03 (m, 8H), 2.45 (s, 3H, CH_3_); ^13^C NMR (75 MHz, CDCl_3_) δ: 167.4, 166.4, 164.2, 163.1, 150.1 (2xC), 146.1, 140.0, 136.1, 133.0, 132.0, 130.6, 130.3, 130.0, 129.6, 125.9, 124.2, 123.5, 122.8, 121.6, 119.7, 115.6, 115.3, 20.7; HRMS calcd. for C_26_H_19_F_1_N_3_O_2_ [M-H]^−^: 424.1467; found: 424.1468.

#### 4.7.4 2-(4-fluorobenzamido)-*N*-(4-methyl-2-(pyridin-3-yl)phenyl)benzamide **(13d)**

Yield: 60%; ^1^H NMR (300 MHz, CDCl_3_) δ: 11.99 (bs, 1H, NH), 8.75 (d, 1H, *J* = 8.4 Hz), 8.51–8.47 (m, 2H), 8.41 (bs, 1H), 8.04–7.71 (m, 4H), 7.51–6.99 (m, 8H), 2.44 (s, 3H, CH_3_); ^13^C NMR (75 MHz, DMSO-*d*_6_) δ: 162.4, 155.9, 138.9, 138.3, 137.0, 136.0, 135.1, 133.1, 132.7, 131.5, 130.6, 129.9, 129.6 (2xC), 129.4, 128.4 (2xC), 127.0, 126.0, 124.8, 124.2, 124.0, 120.9, 119.4 (2xC), 20.6; HRMS calcd. for C_26_H_19_F_1_N_3_O_2_ [M-H]^−^: 424.1467; found: 424.1472.

### 4.8 Antiviral activity determination for DENV and YFV

Antiviral activities were determined as described before by Saudi et al. [1] and reflect the activities as determined versus Dengue virus serotype 2.
