# Supplementary material for: Synthetic strategy and antiviral evaluation of diamide containing heterocycles targeting dengue and yellow fever virus
Source: Eur J Med Chem. 2016 Oct 4;121:158–68. doi: 10.1016/j.ejmech.2016.05.043 (PMC4999056; doi:10.1016/j.ejmech.2016.05.043)

Supporting Information

Synthetic strategy and antiviral evaluation of diamide containing heterocycles targeting dengue and yellow fever virus

Milind Saudi[a], Joanna Zmurko[b], Suzanne Kaptein[b], Jef Rozenski[a], Bharat Gadakh[a], Patrick Chaltin[c,d], Arnaud Marchand[c], Johan Neyts[b], Arthur Van Aerschot[a]*∞

[a] KU Leuven, Medicinal Chemistry, Rega Institute for Medical Research, Minderbroedersstraat 10, 3000 Leuven, Belgium

[b] KU Leuven, Laboratory of Virology and Chemotherapy, Rega Institute for Medical Research, Minderbroedersstraat 10, 3000 Leuven, Belgium

[c] CISTIM Leuven vzw, Gaston Geenslaan 2, 3001 Leuven Belgium

[d] Centre for Drug Design and Discovery (CD3), KULeuven Research and Development, Waaistraat 6, 3000 Leuven, Belgium

List of contents

General Information……………………..…………………..…………....S2

HRMS, ^{1}H and ^{13}C NMR spectra ……..……..……..……………S3-S69

General Information

Reagents and solvents were purchased from commercial suppliers (Acros, Sigma-Aldrich, Bachem, Novabiochem) and used as provided, unless indicated otherwise. All the solvents were of analytical grade and were stored over 4Å molecular sieves. Reactions were carried out in oven-dried glassware under a nitrogen atmosphere with stirring at 85ºC.

^{1}H and ^{13}C NMR spectra of the compounds dissolved in $CDCl_3$, MeOD or DMSO-d_6 were recorded on a Bruker UltraShield Avance 300 MHz or 500 MHz spectrometer. The chemical shifts are expressed as δ values in parts per million (ppm), using the residual solvent peaks ($CDCl_3$: ^{1}H, 7.26 ppm; ^{13}C, 77.00 ppm; MeOD: ^{1}H, 3.31 ppm; ^{13}C, 49.00 ppm) as a reference. Coupling constants are given in Hertz (Hz). The peak patterns are indicated by the following abbreviations: bs = broad singlet, d = doublet, m = multiplet, q = quadruplet, s = singlet and t = triplet.

Spectra were acquired on a quadrupole orthogonal acceleration time-of-flight mass spectrometer (Synapt G2 HDMS, Waters, Milford, MA). Samples were infused at 3 μL.min^{-1} and spectra were obtained in positive (or negative) ionization mode with a resolution of 15000 (FWHM) using leucine enkephalin as lock mass.

For TLC, precoated aluminium sheets were used (Merck, Silica gel 60 F_{254}). The spots were visualized by UV light at 254 nm. Column chromatography was performed on ICN silica gel 60Aº 40-60 μM.

This supporting information provides the ^{1}H and ^{13}C NMR and HRMS spectra for all new final compounds with numbering according to the manuscript. NMR spectra were not decoupled for fluorine, and especially carbon spectra are often difficult to interpret with in some cases double signals for rotamers and fluorine coupled carbon signals, leading often to missing signals for quaternary carbons.

HRMS, ^{1}H and ^{13}C NMR Spectra

3-(p-tolylcarbamoyl)pyrazine-2-carboxylic acid (4a)

HRMS

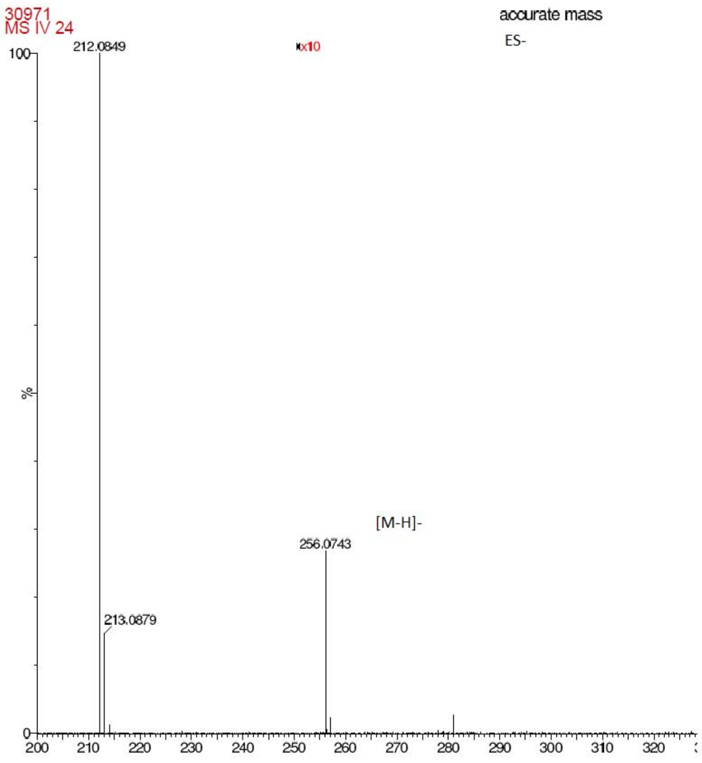

^{1}H NMR (300 MHz, DMSO)

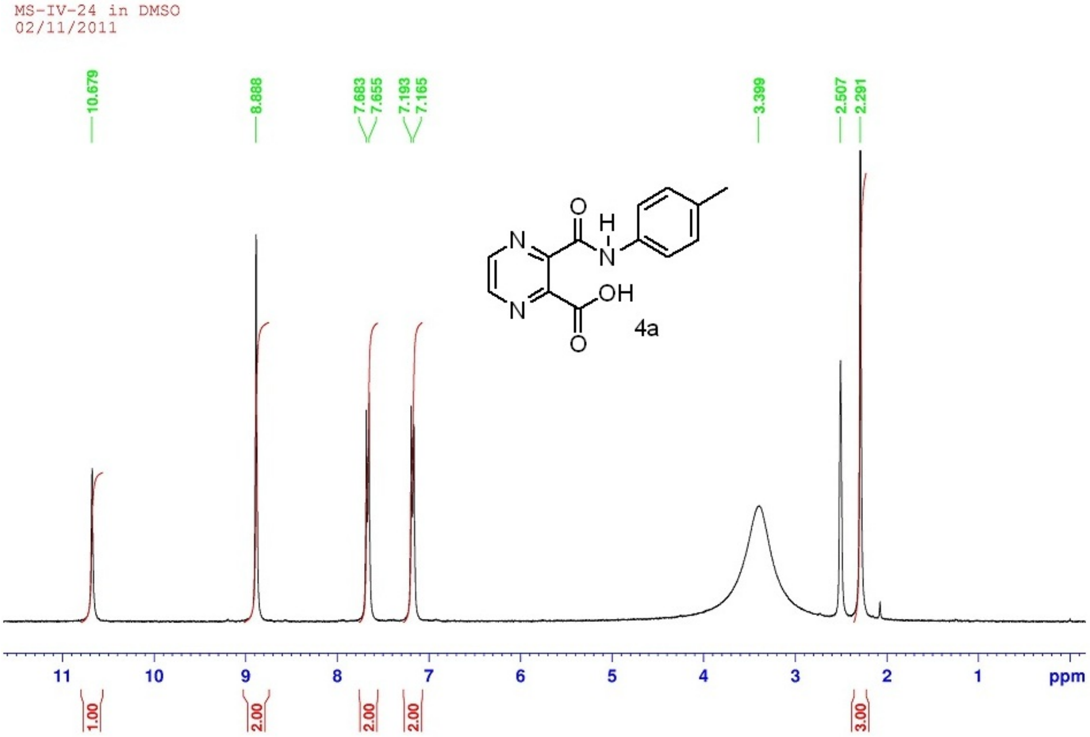

^{13}C NMR (75 MHz, DMSO)

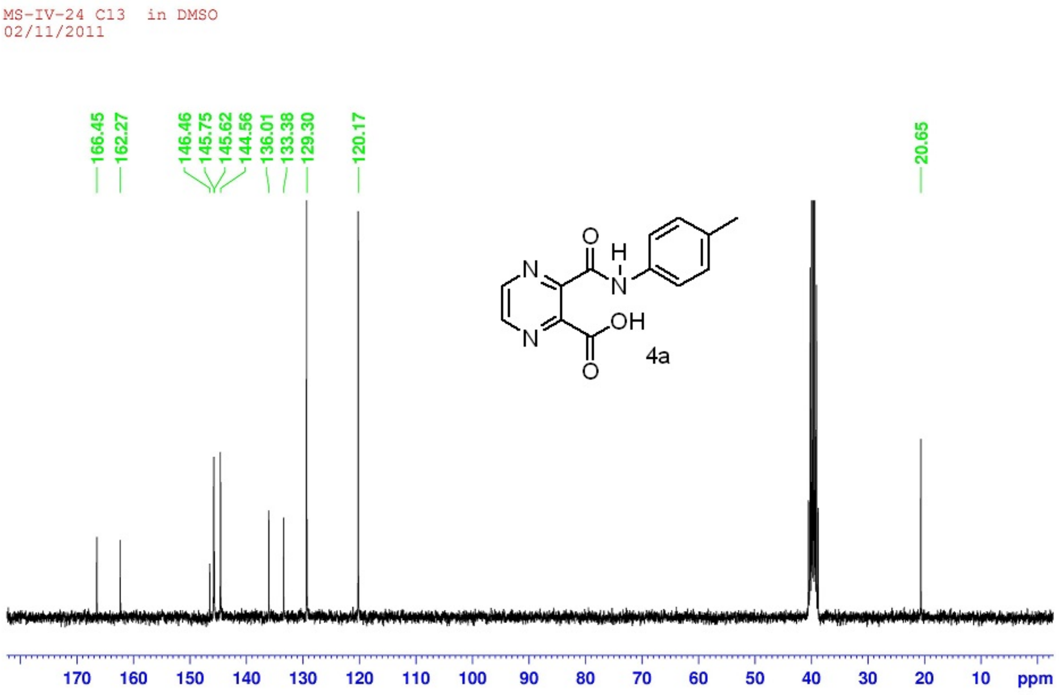

3-((4-fluorophenyl)carbamoyl)pyrazine-2-carboxylic acid (4b)
HRMS

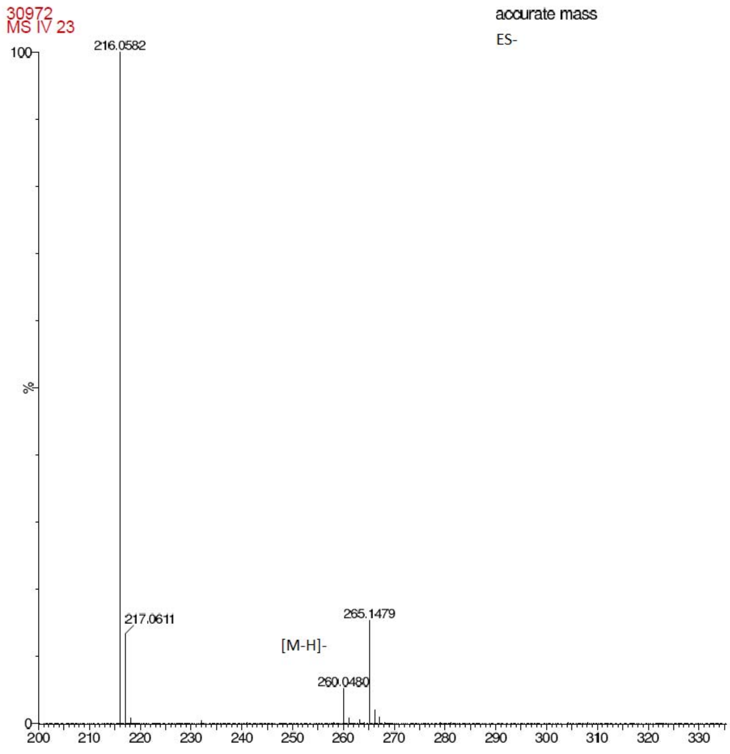

^{1}H NMR (300 MHz, DMSO)

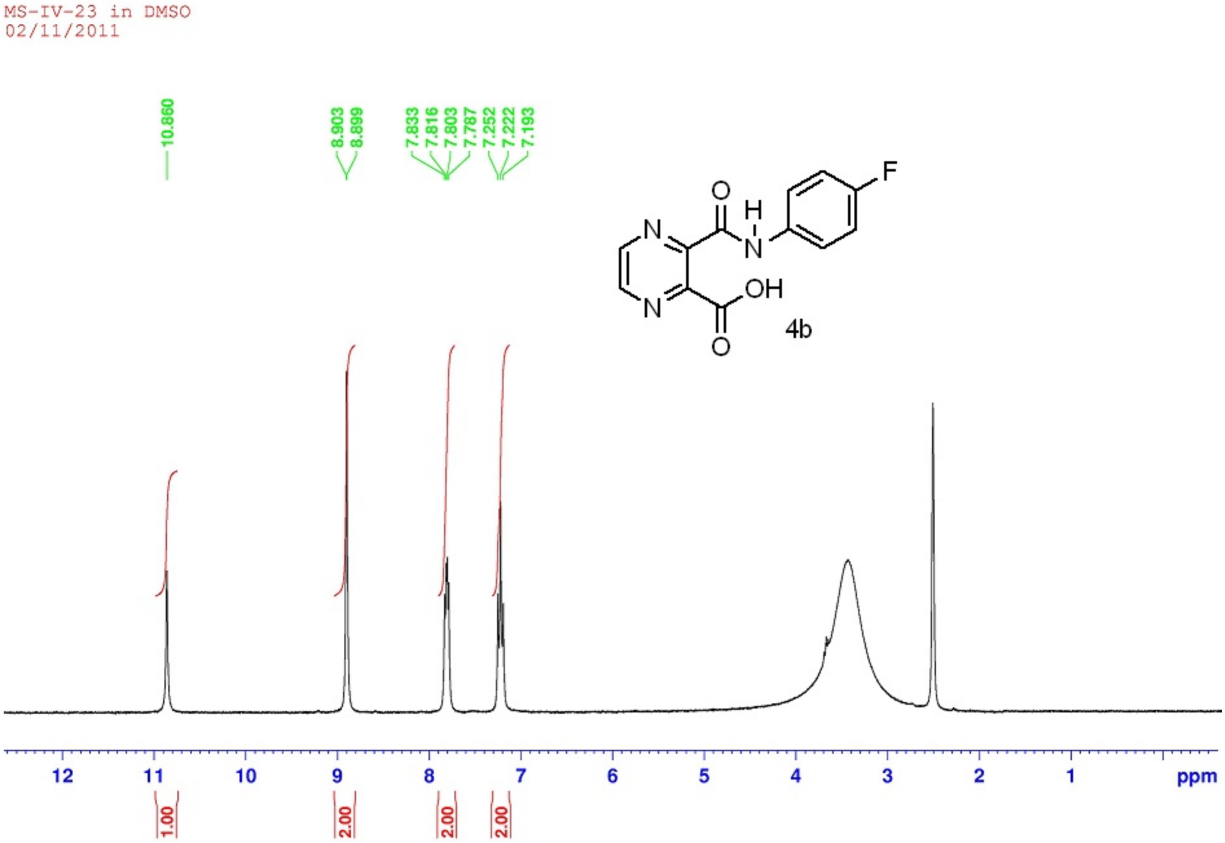

^{13}C NMR (75 MHz, DMSO)

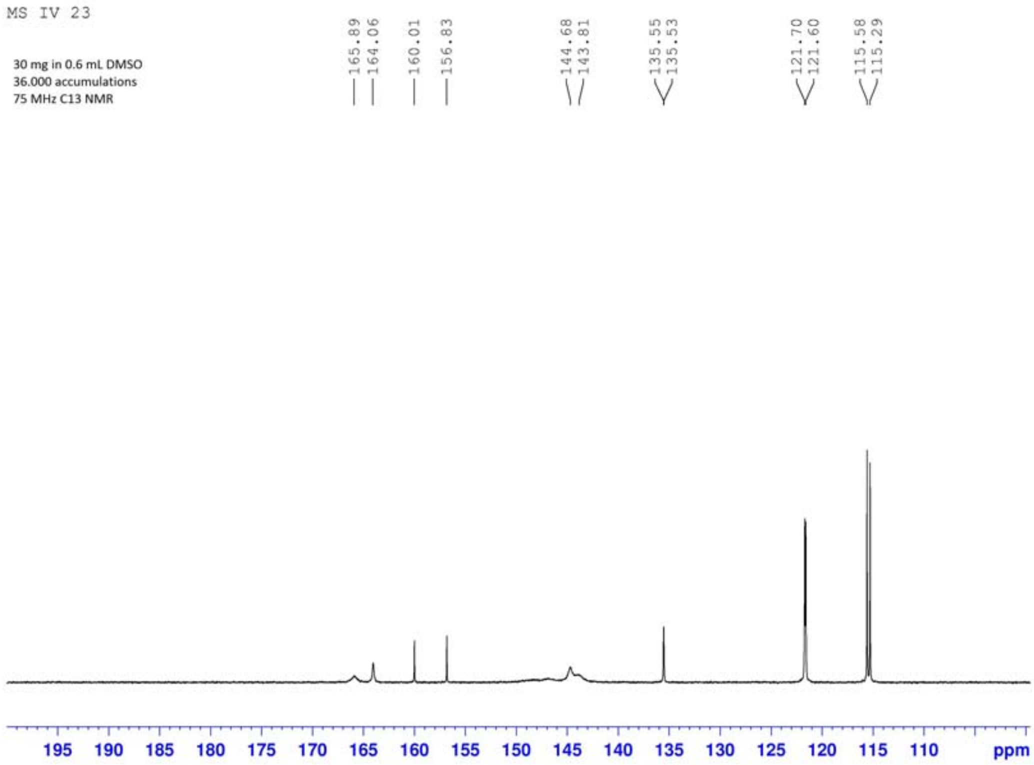

2-(p-tolylcarbamoyl)benzoic acid (4c)

HRMS

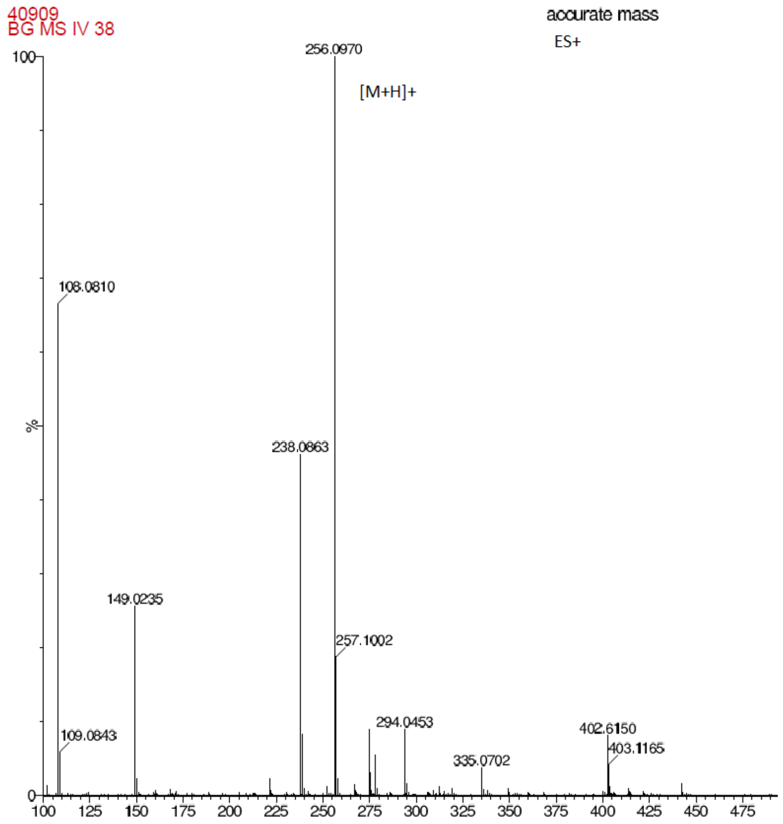

^{1}H NMR (300 MHz, DMSO)

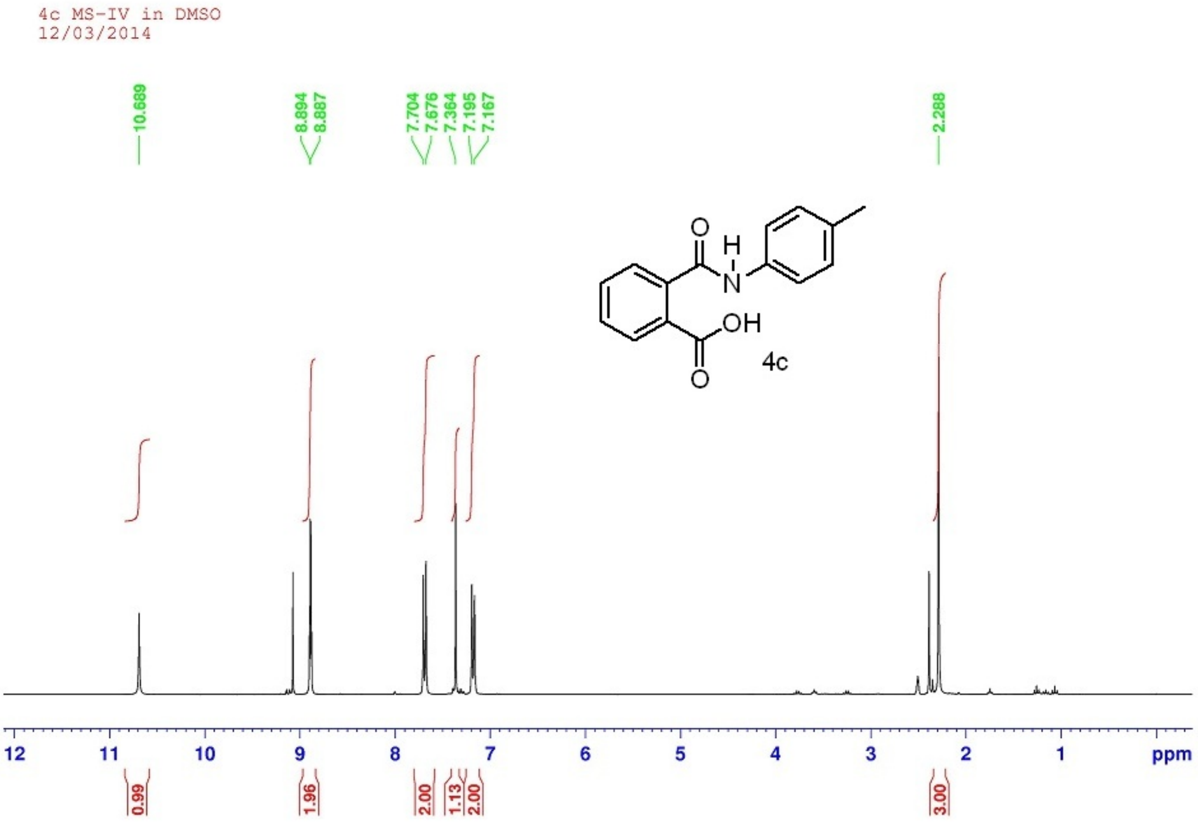

^{13}C NMR (75 MHz, DMSO)

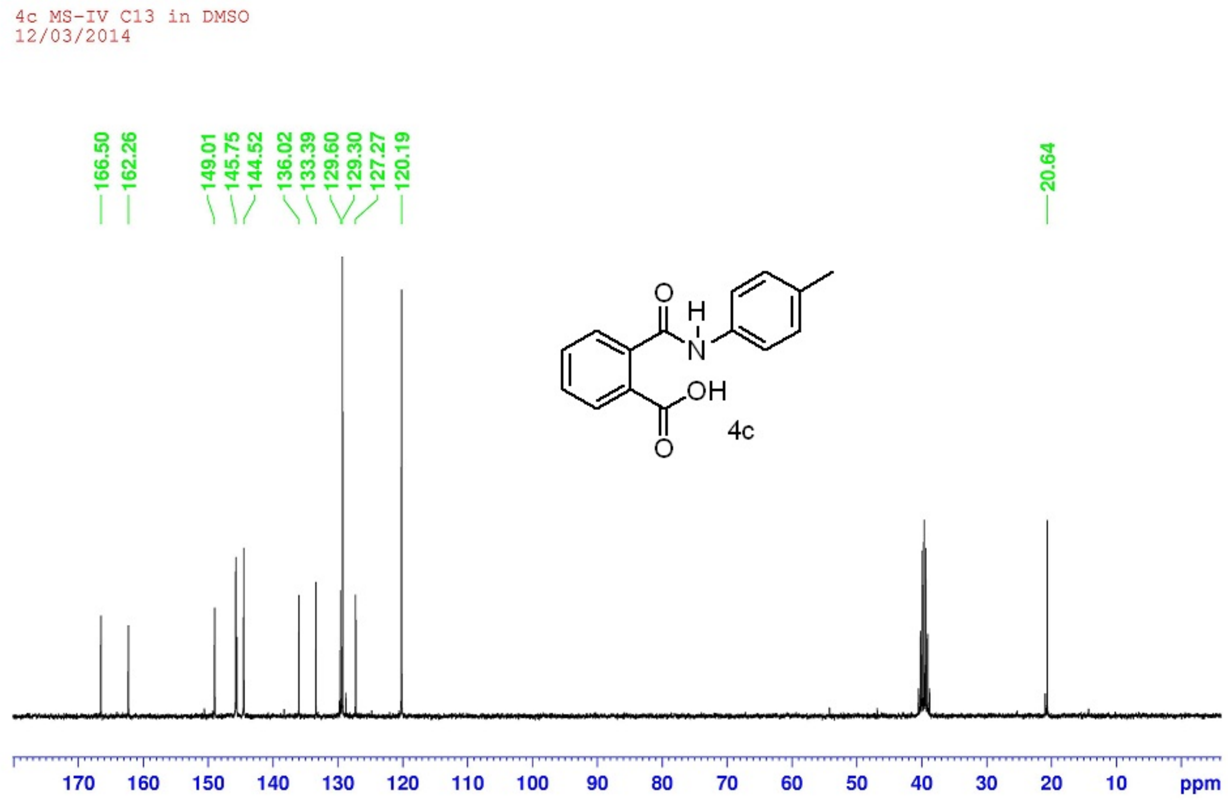

2-((4-fluorophenyl)carbamoyl)benzoic acid (4d)
HRMS

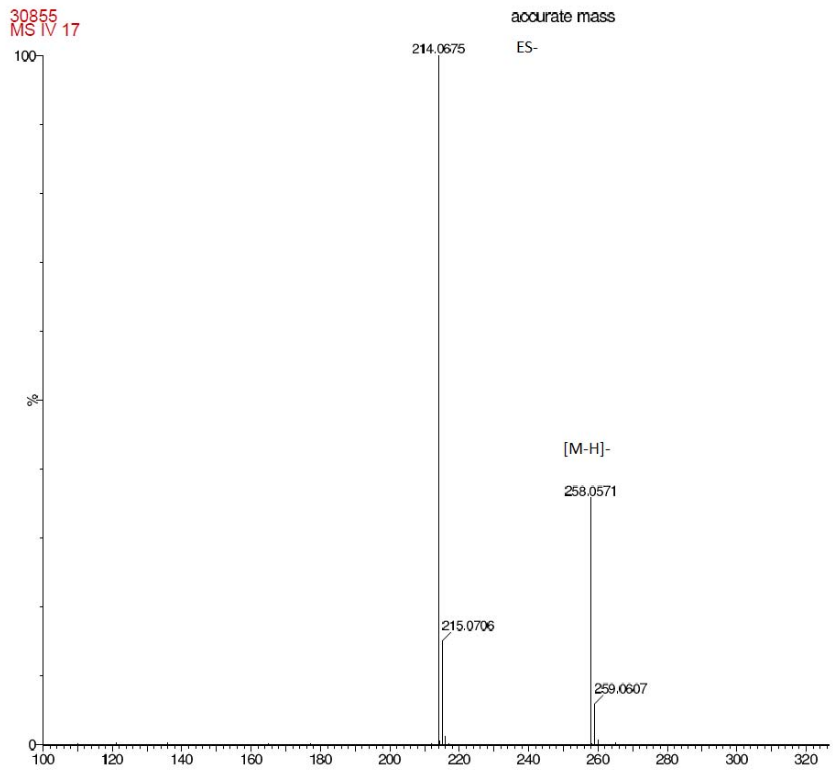

^{1}H NMR (300 MHz, DMSO)

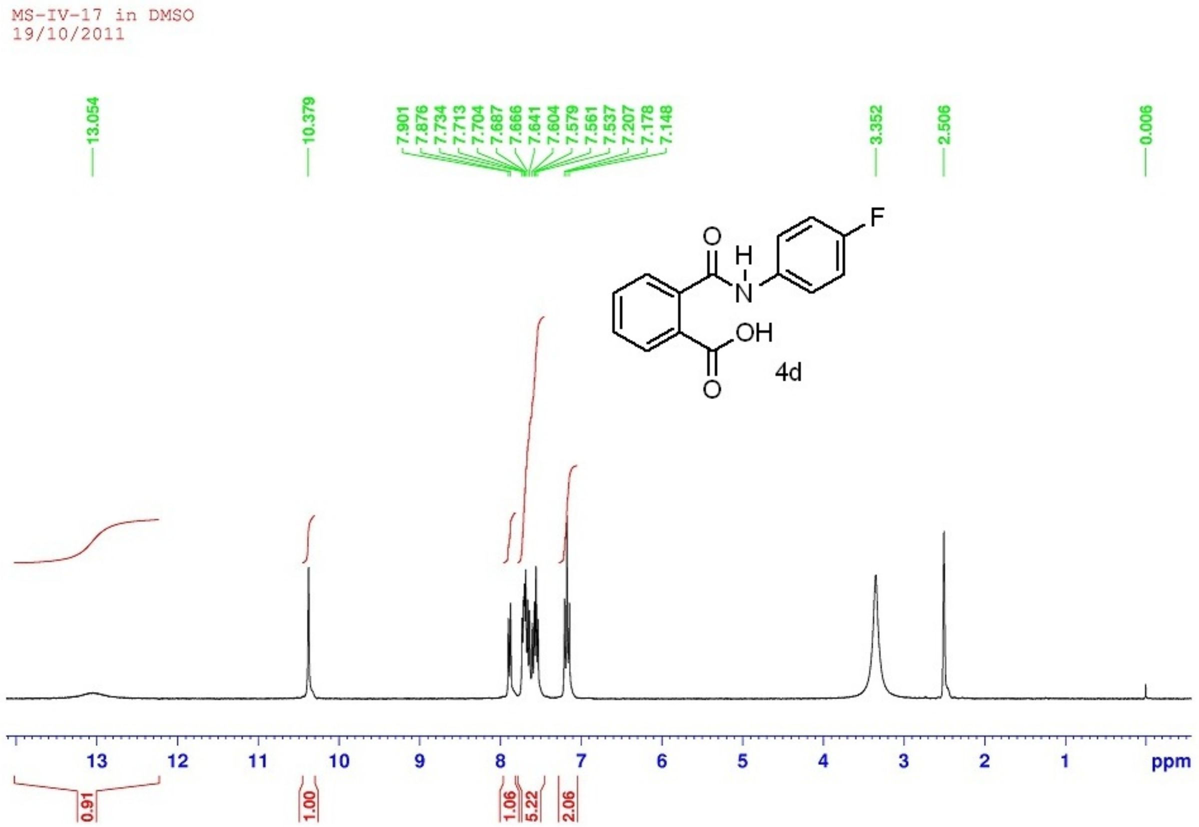

^{13}C NMR (75 MHz, DMSO)

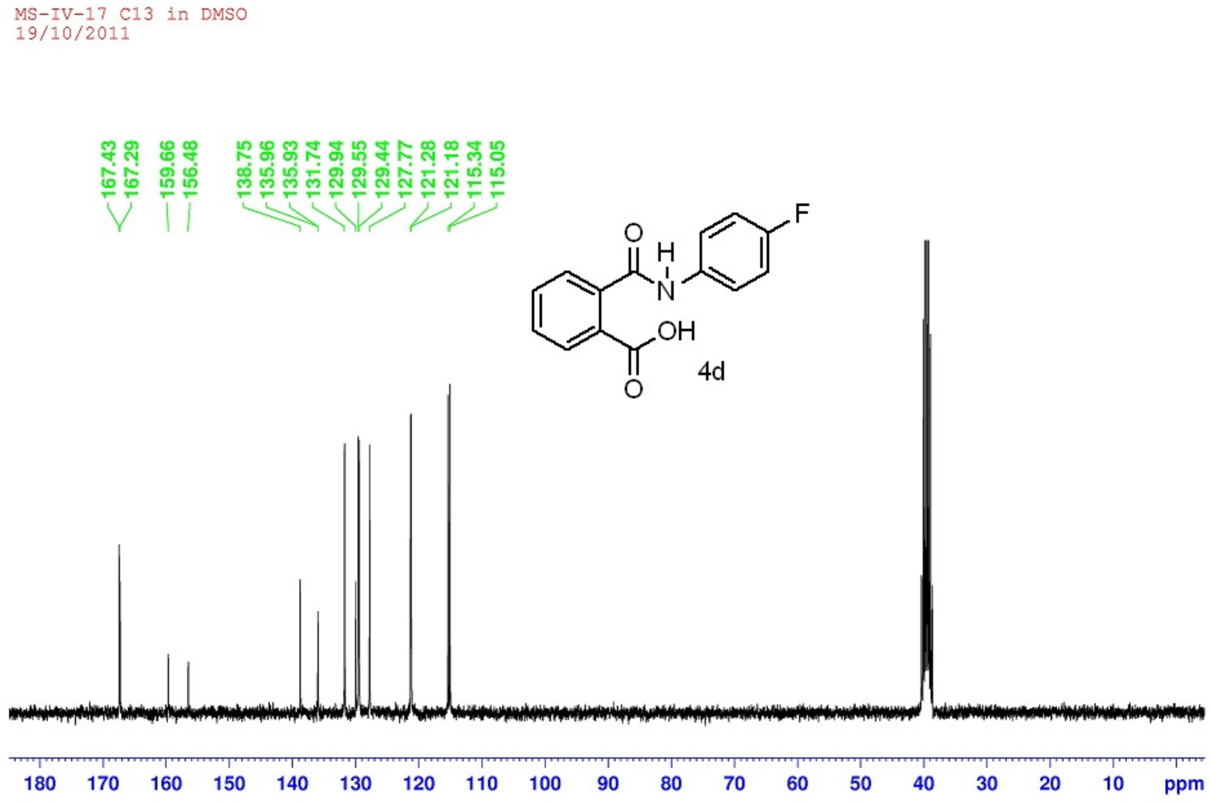

(Z)-7-(p-tolylimino)furo[3,4-b]pyrazin-5(7H)-one(5a)
HRMS

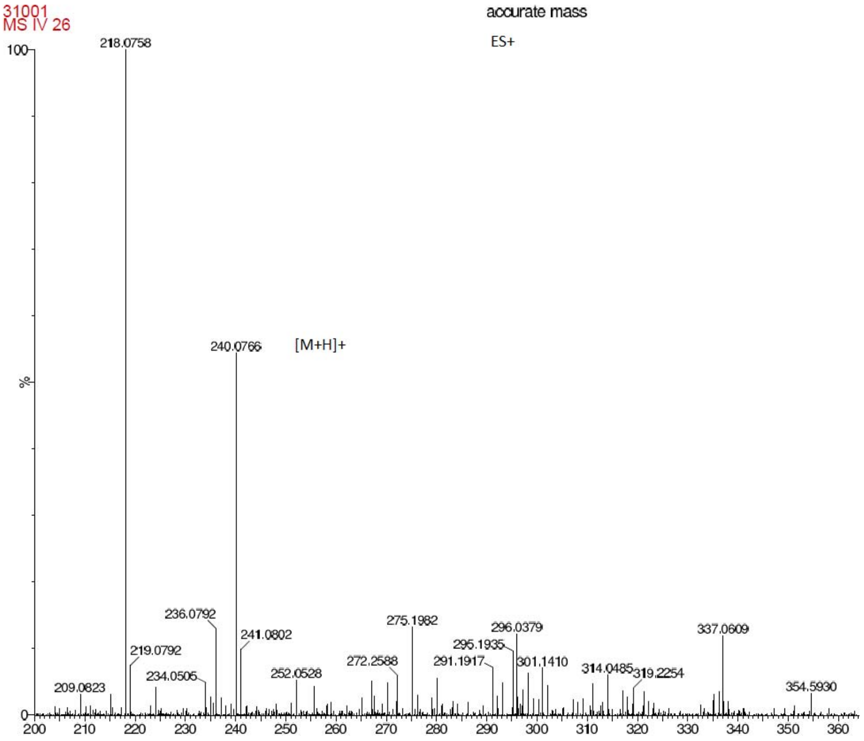

^{1}H NMR (300 MHz, $CDCl_3$)

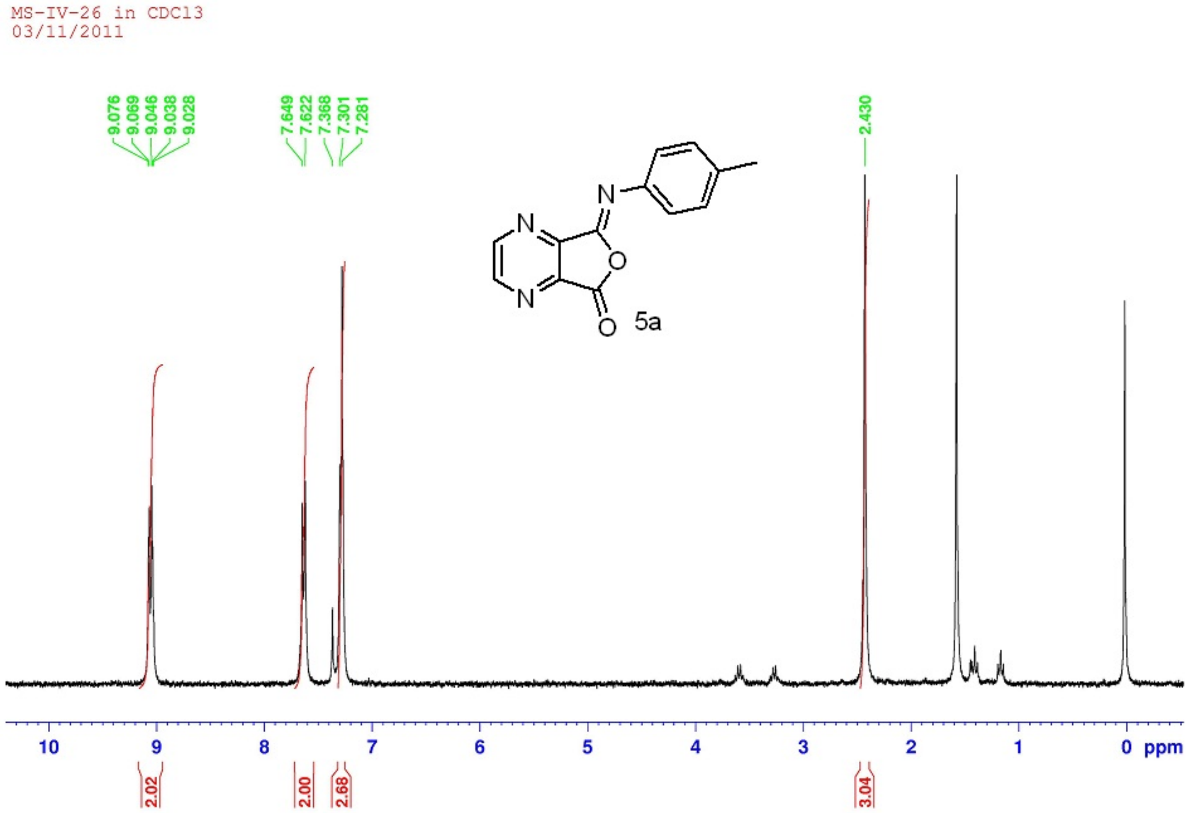

^{13}C NMR (75 MHz, $CDCl_3$)

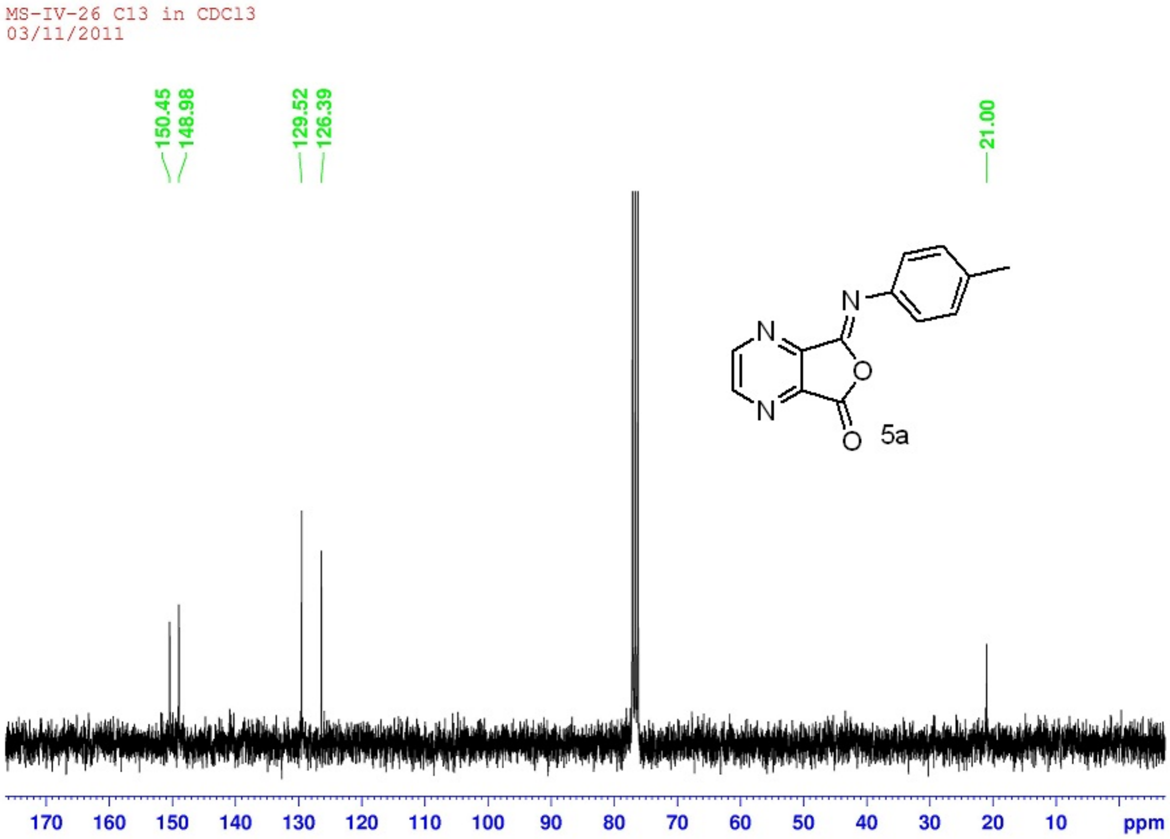

(Z)-7-((4-fluorophenyl)imino)furo[3,4-b]pyrazin-5(7H)-one (5b)

HRMS

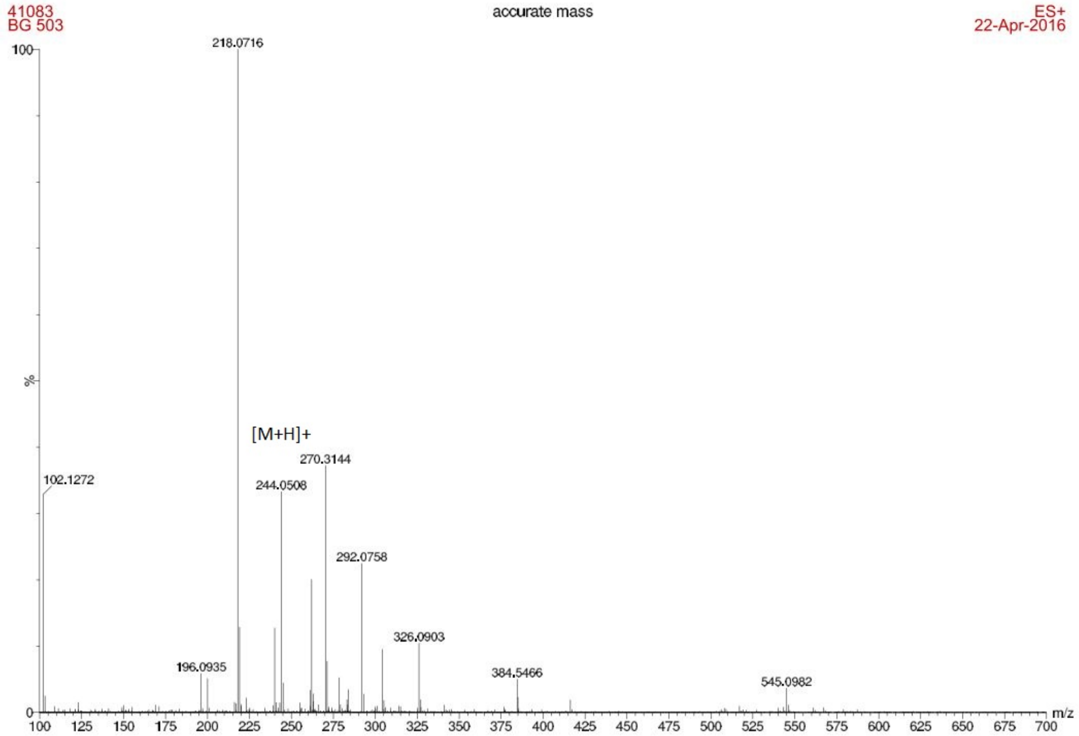

^{1}H NMR (300 MHz, $CDCl_3$)

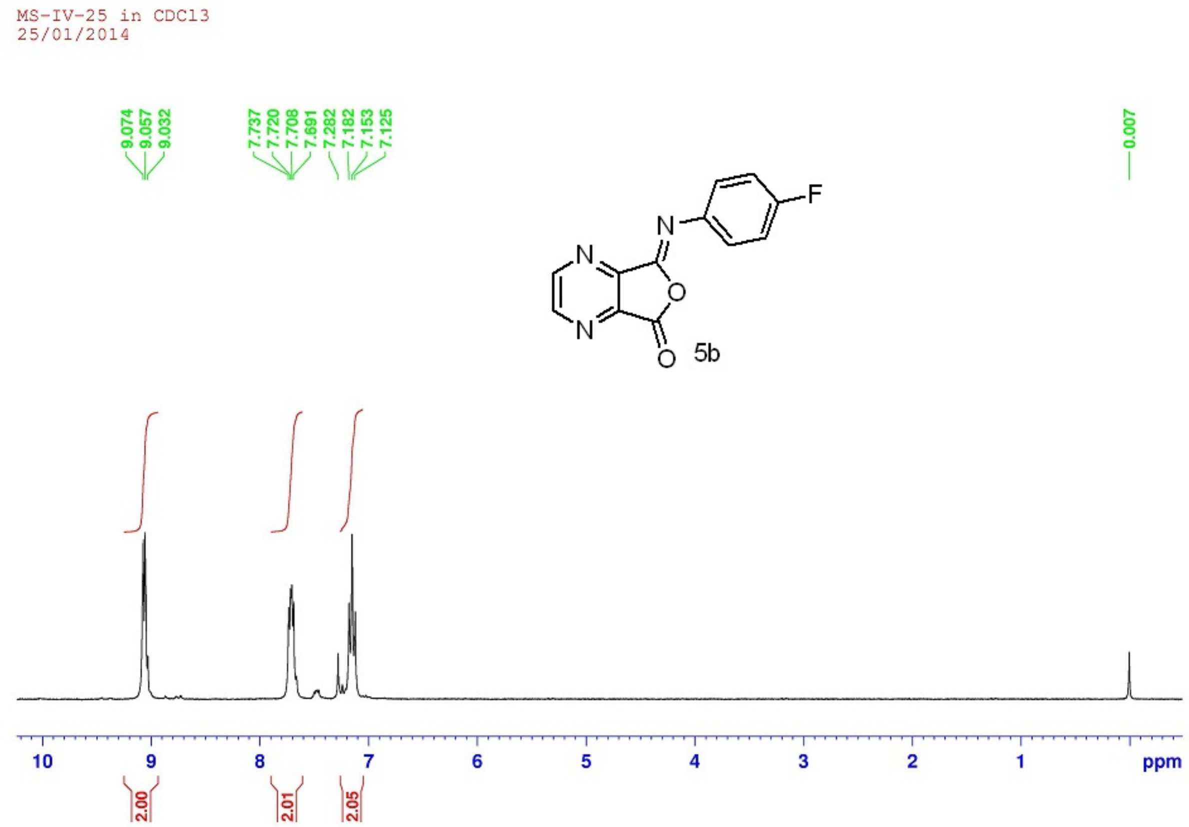

^{13}C NMR (75 MHz, $CDCl_3$)

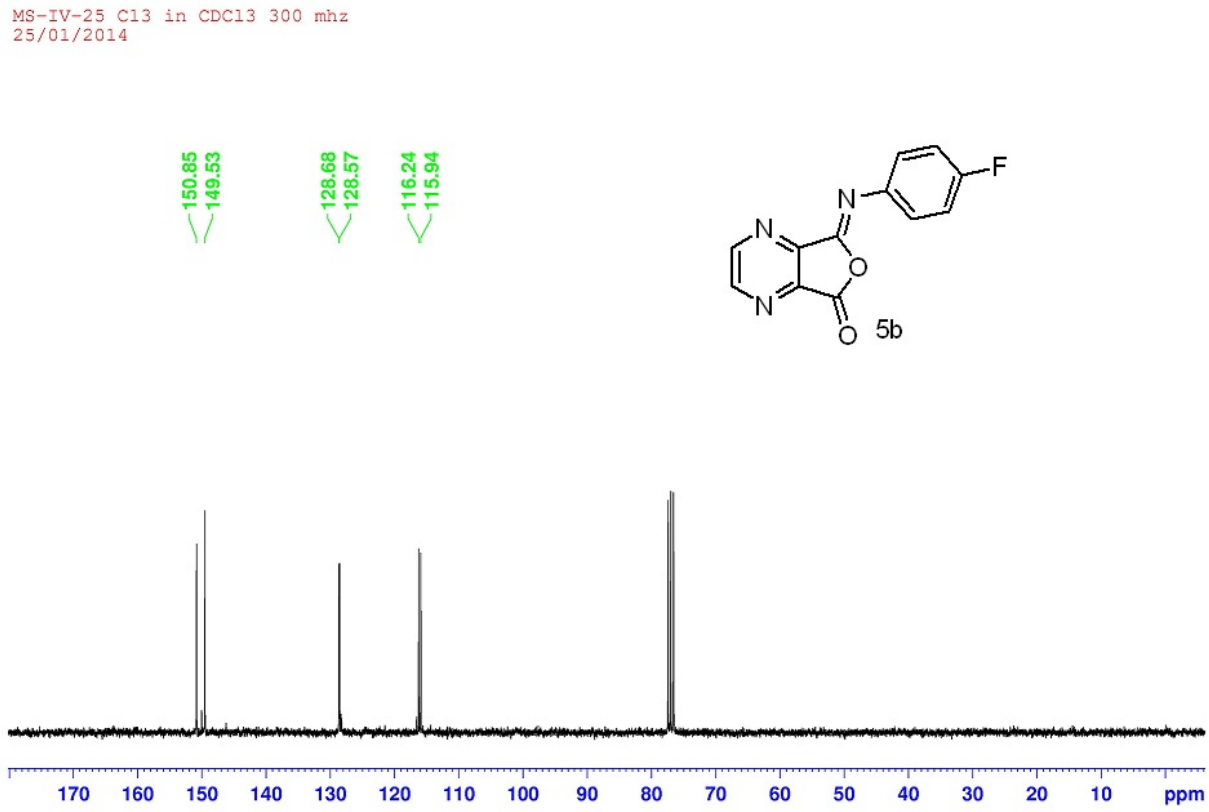

(Z)-3-(p-tolylimino)isobenzofuran-1(3H)-one (5c)
HRMS

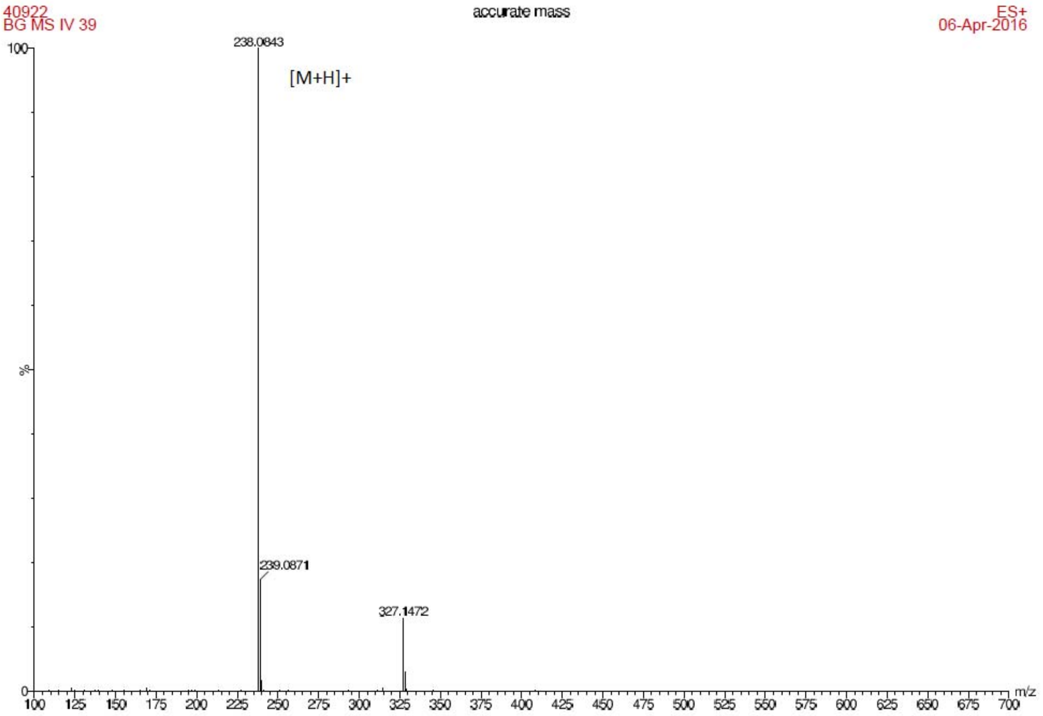

^{1}H NMR (300 MHz, $CDCl_3$)

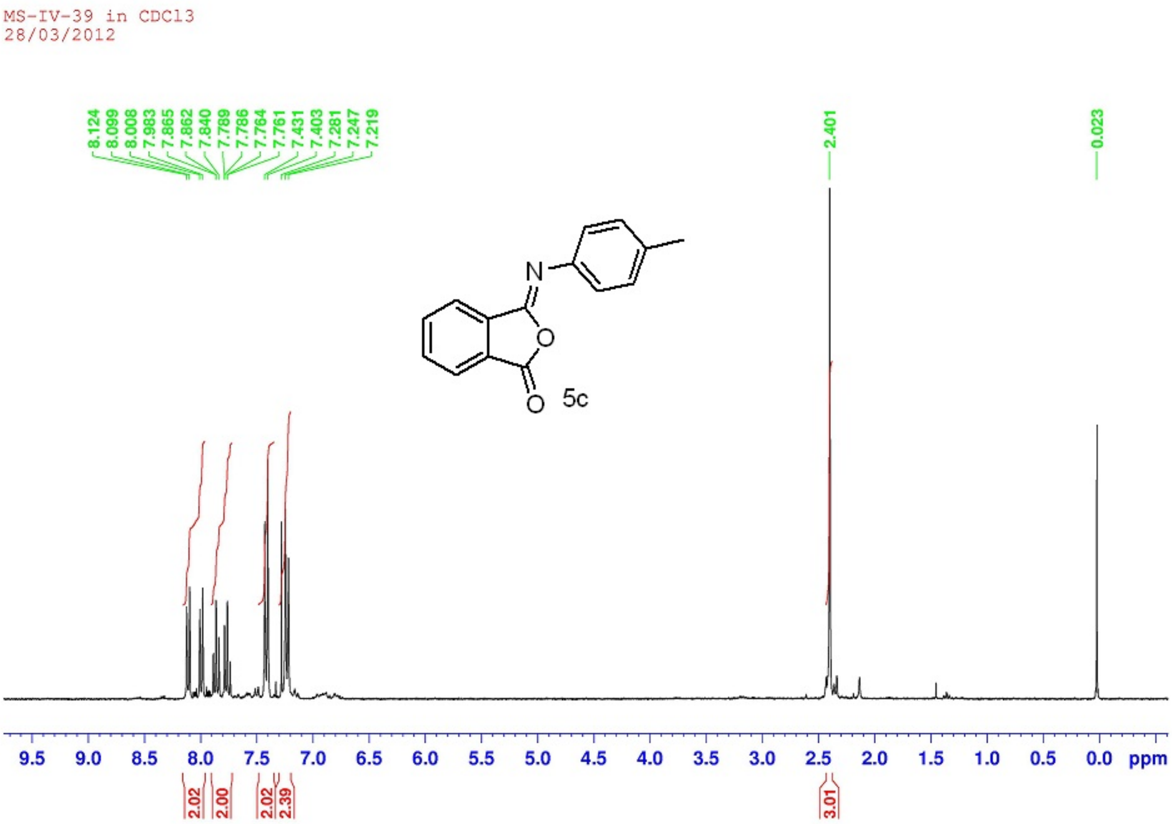

^{13}C NMR (75 MHz, DMSO)

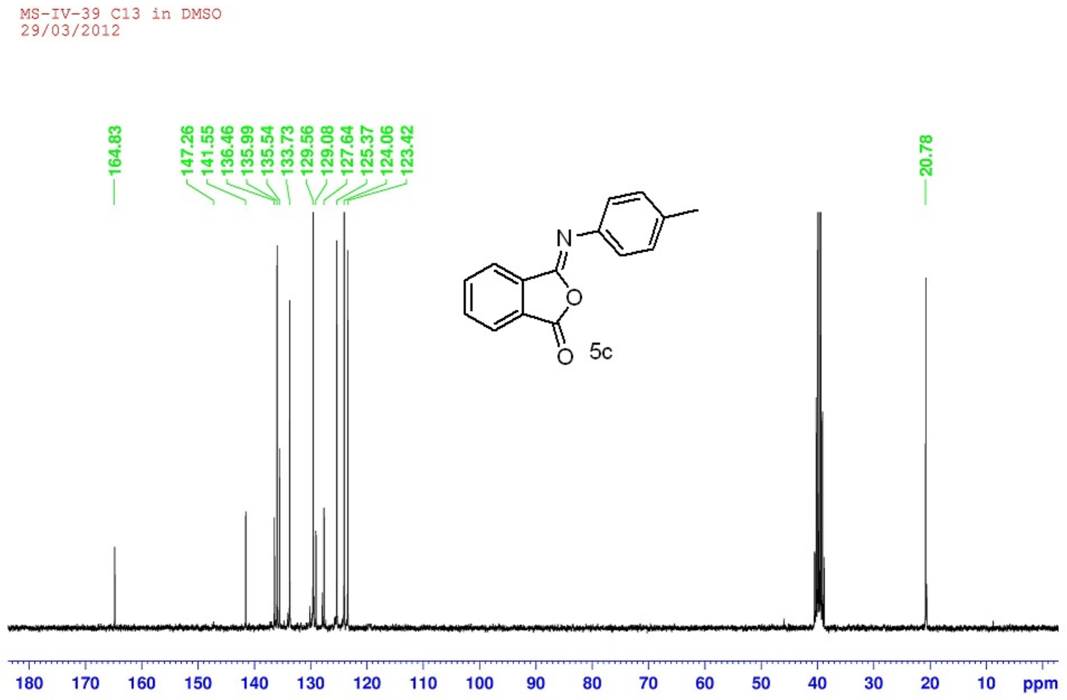

(Z)-3-((4-fluorophenyl)imino)isobenzofuran-1(3H)-one (5d)

HRMS

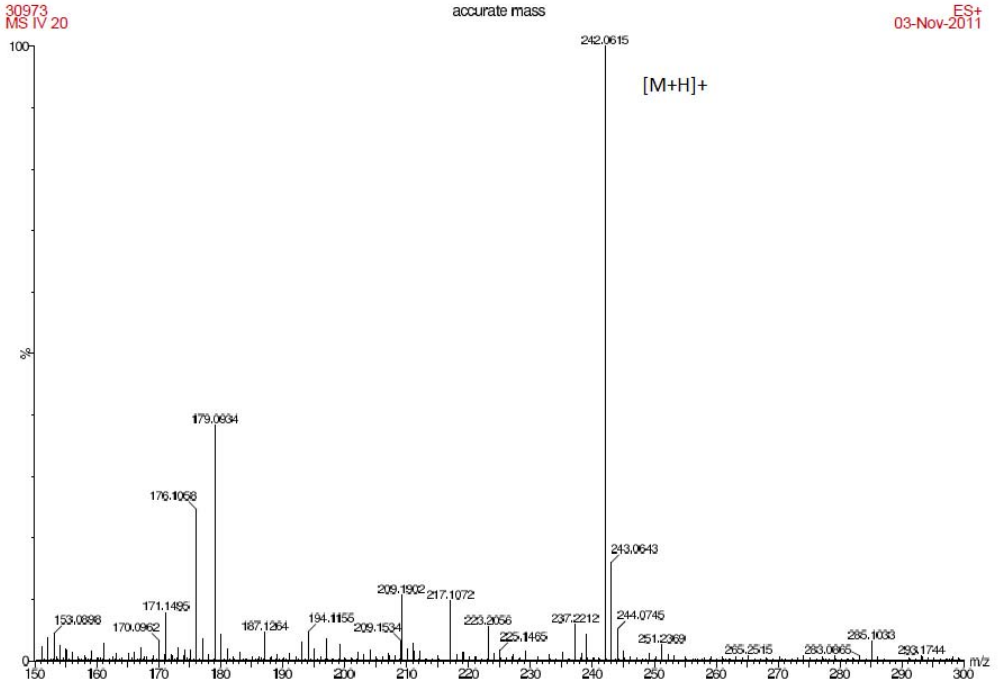

^{1}H NMR (300 MHz, DMSO)

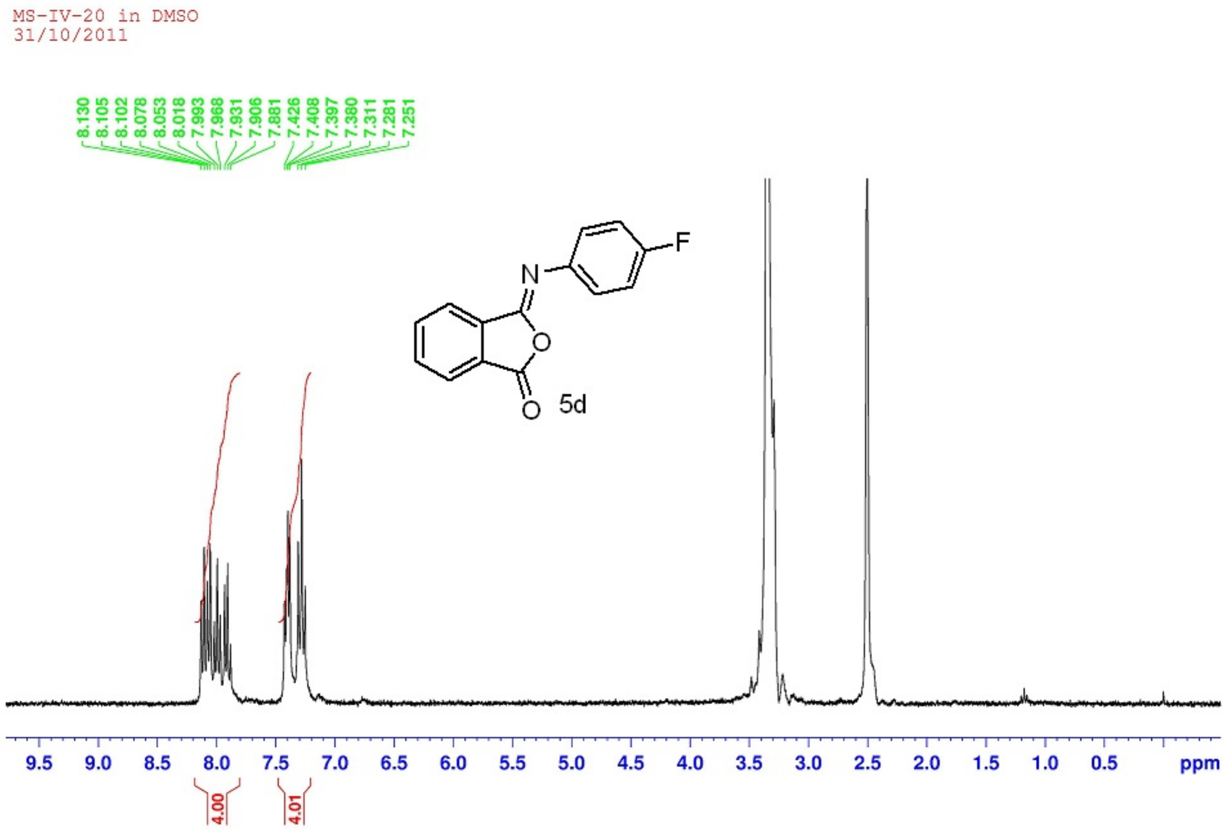

^{13}C NMR (75 MHz, DMSO)

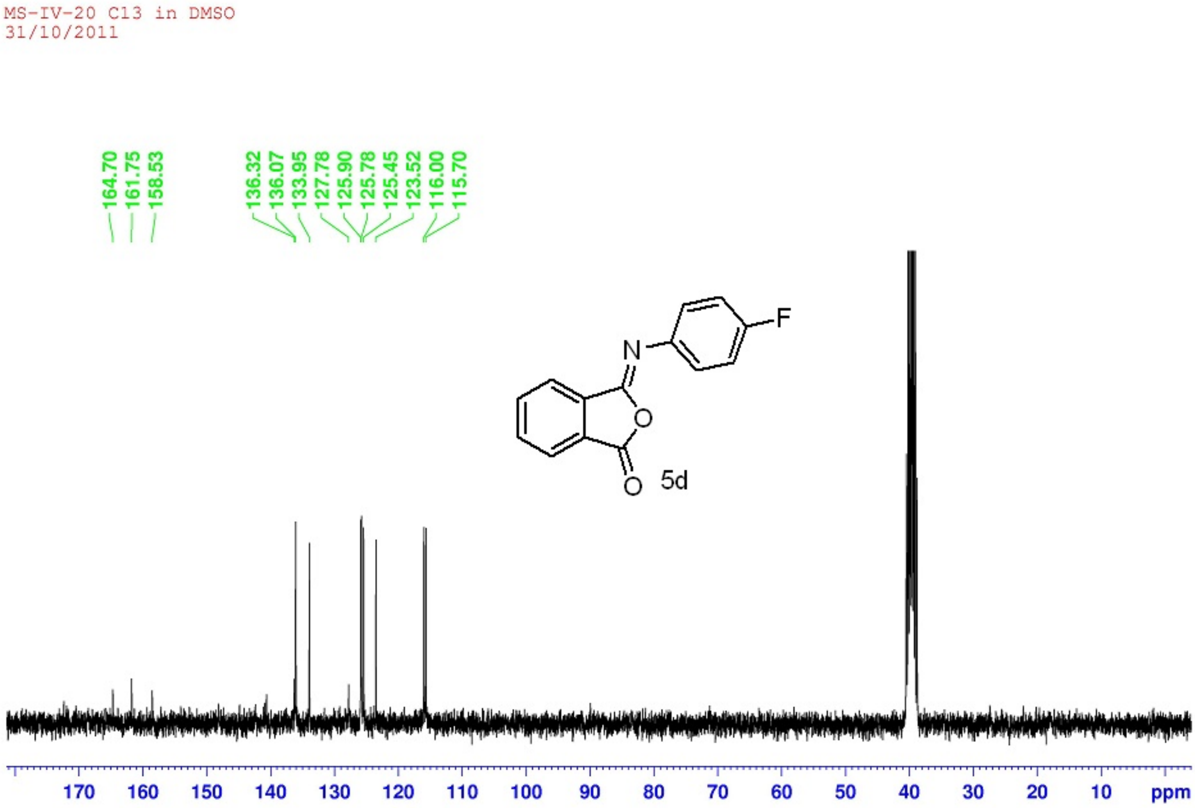

N^2-(4-fluorophenyl)-N^3-(4-methyl-2-(3-methyl-1H-pyrazol-5-yl)phenyl)pyrazine-2,3 dicarboxamide (6a):

HRMS

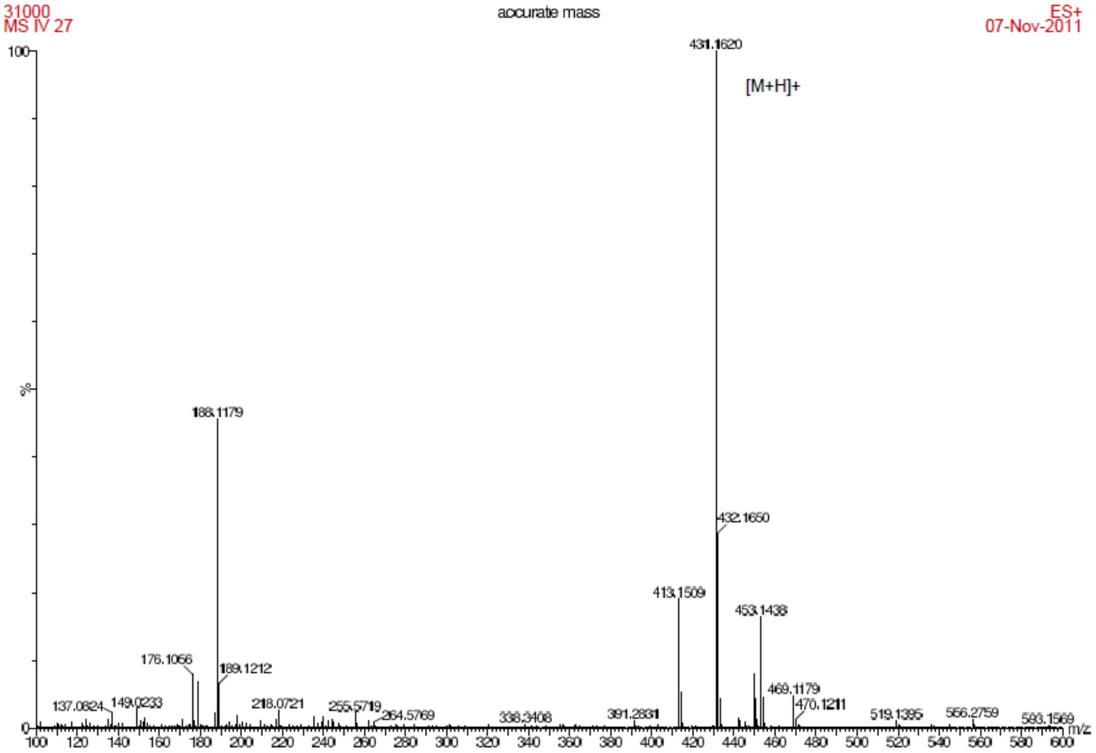

^{1}H NMR (300 MHz, DMSO)

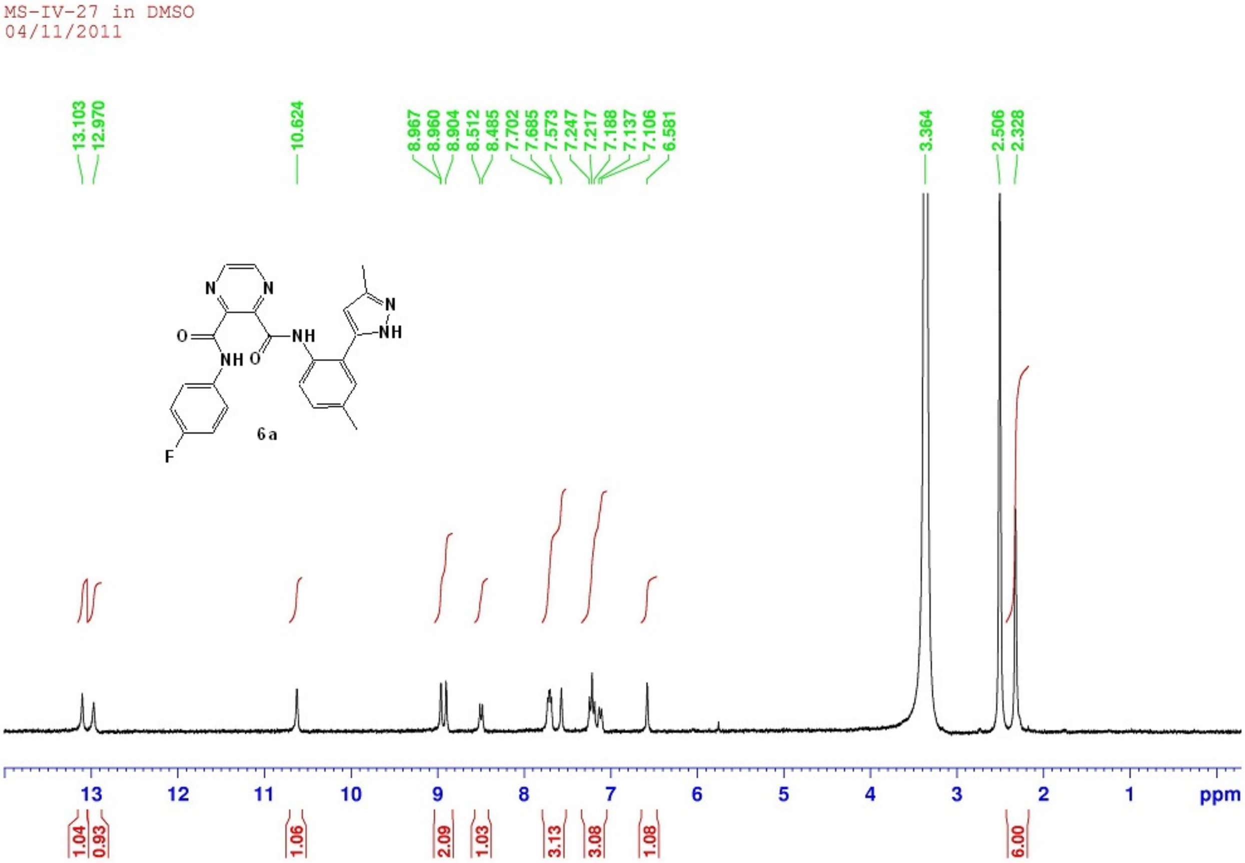

^{13}C NMR (150 MHz, $CDCl_3$)

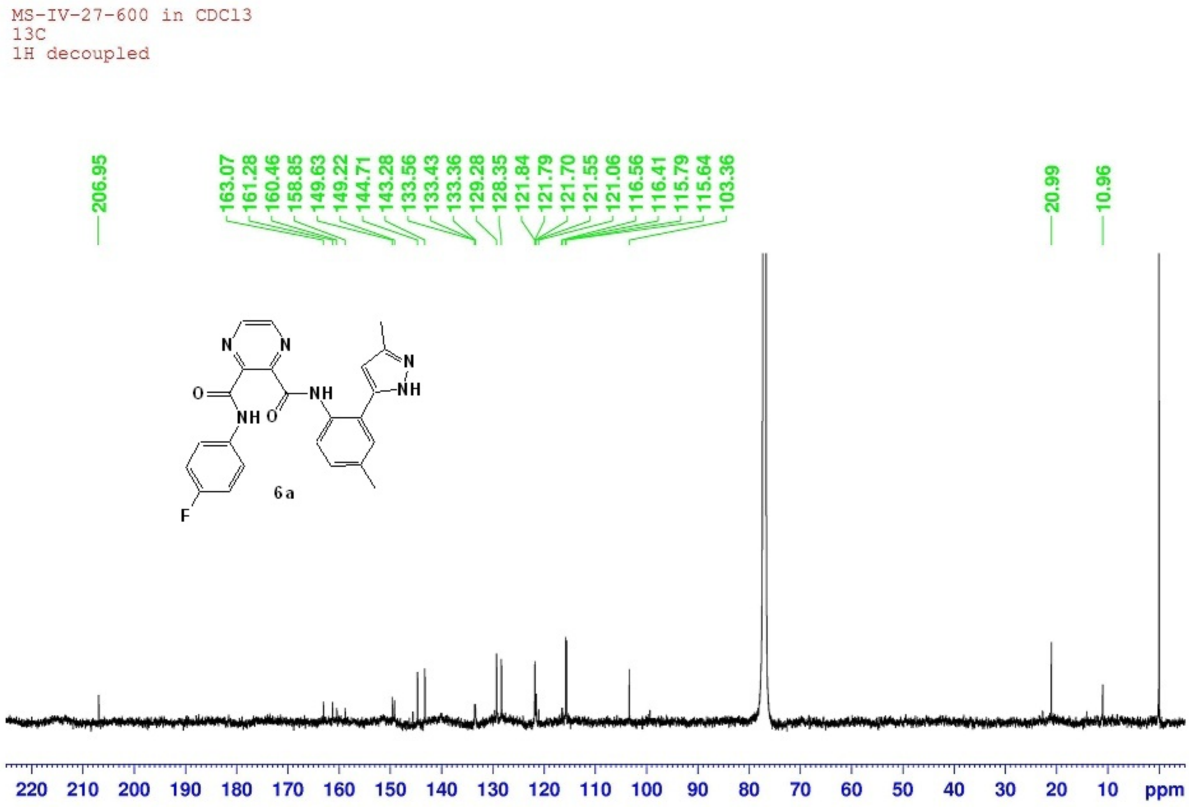

N^2-(4-methyl-2-(3-methyl-1H-pyrazol-5-yl)phenyl)-N^3-(p-tolyl)pyrazine-2,3-dicarbox amide (6b):

HRMS

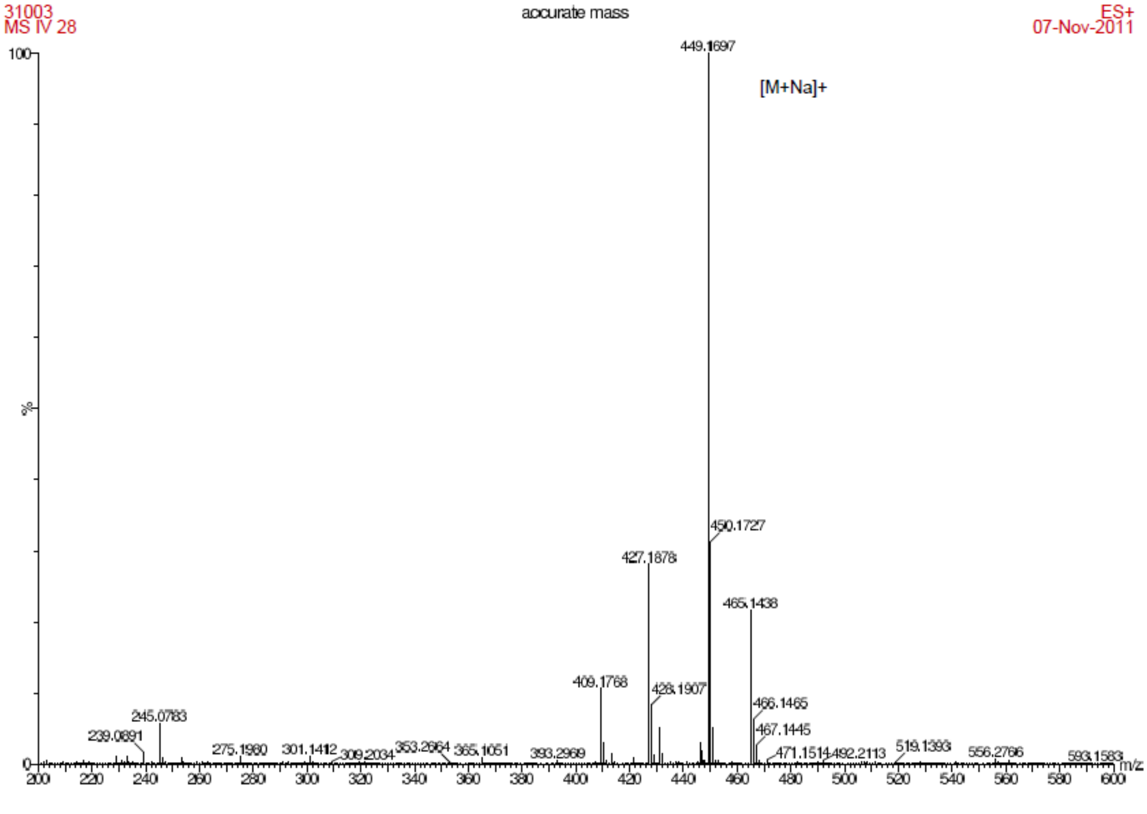

^{1}H NMR (600 MHz, $CDCl_3$)

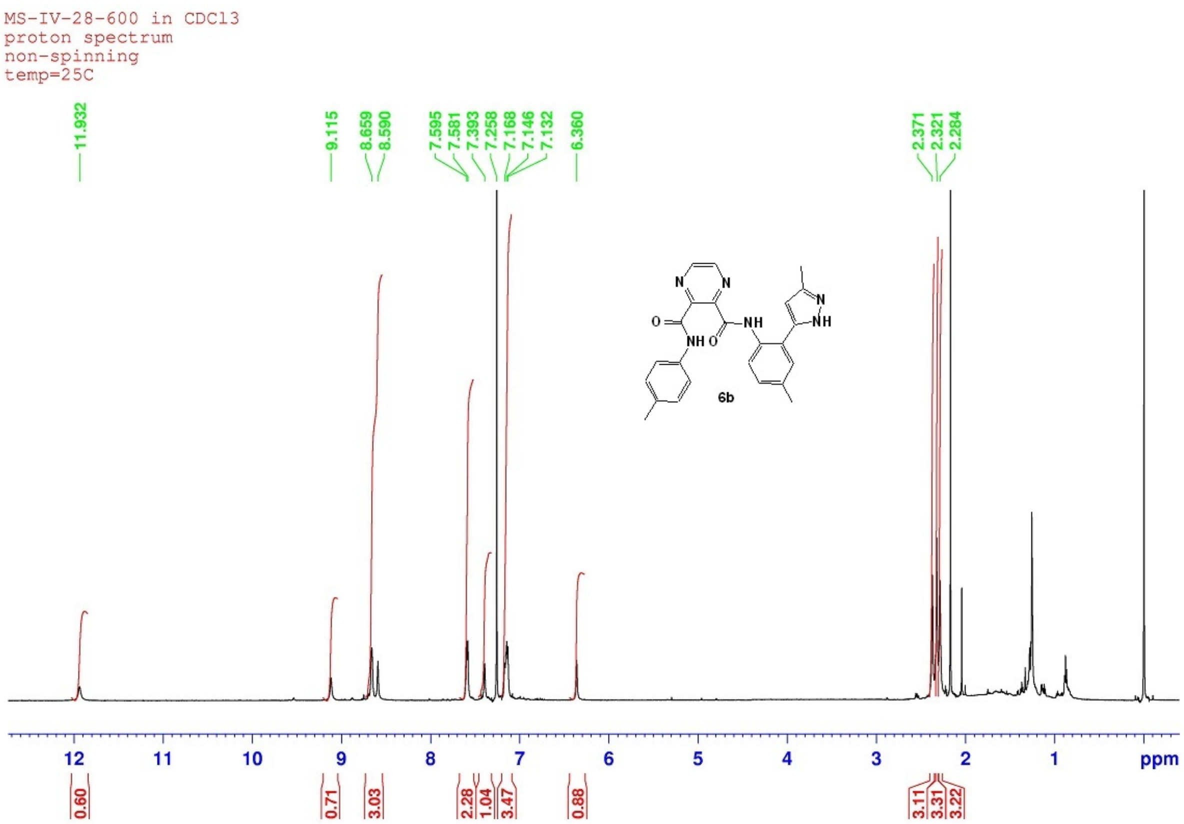

^{13}C NMR (150 MHz, $CDCl_3$)

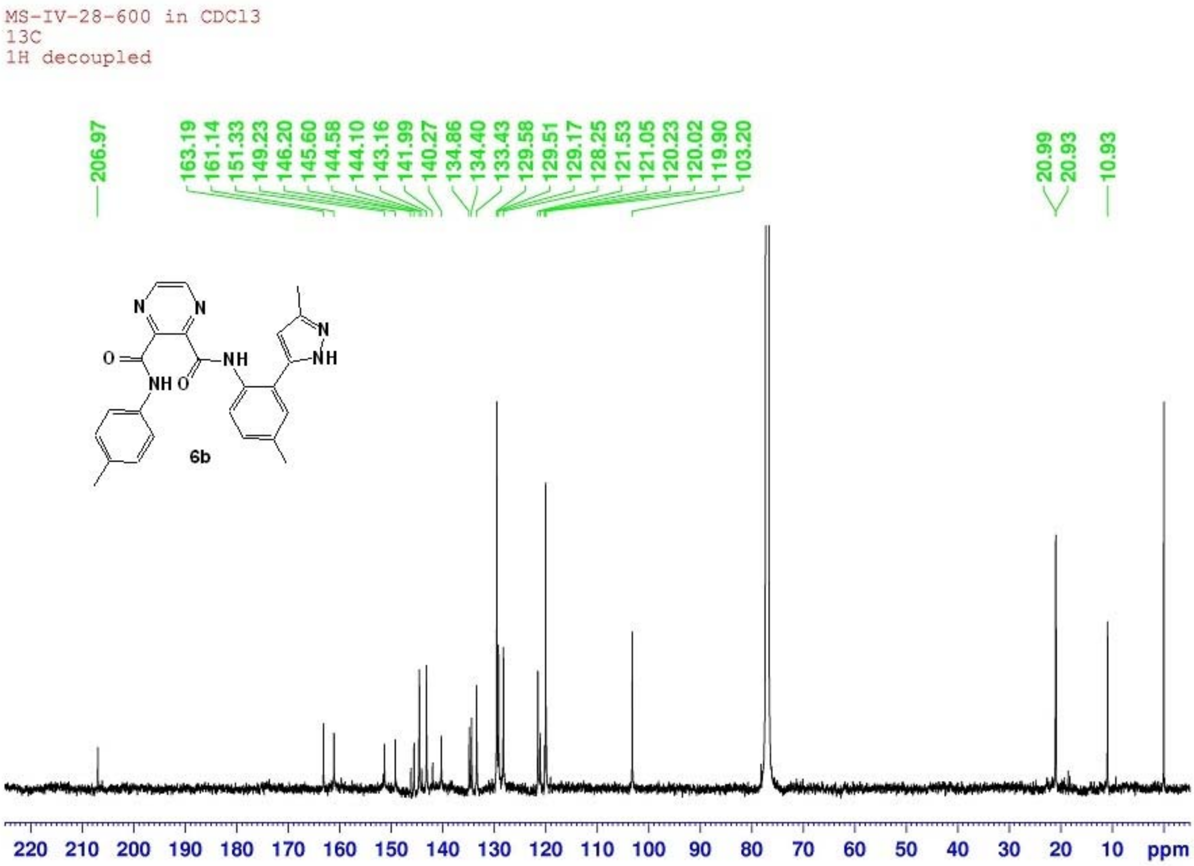

N^2-(4-fluorophenyl)-N^3-(4-methyl-2-(thiophen-2-yl)phenyl)pyrazine-2,3-dicarboxamide (6c)

HRMS

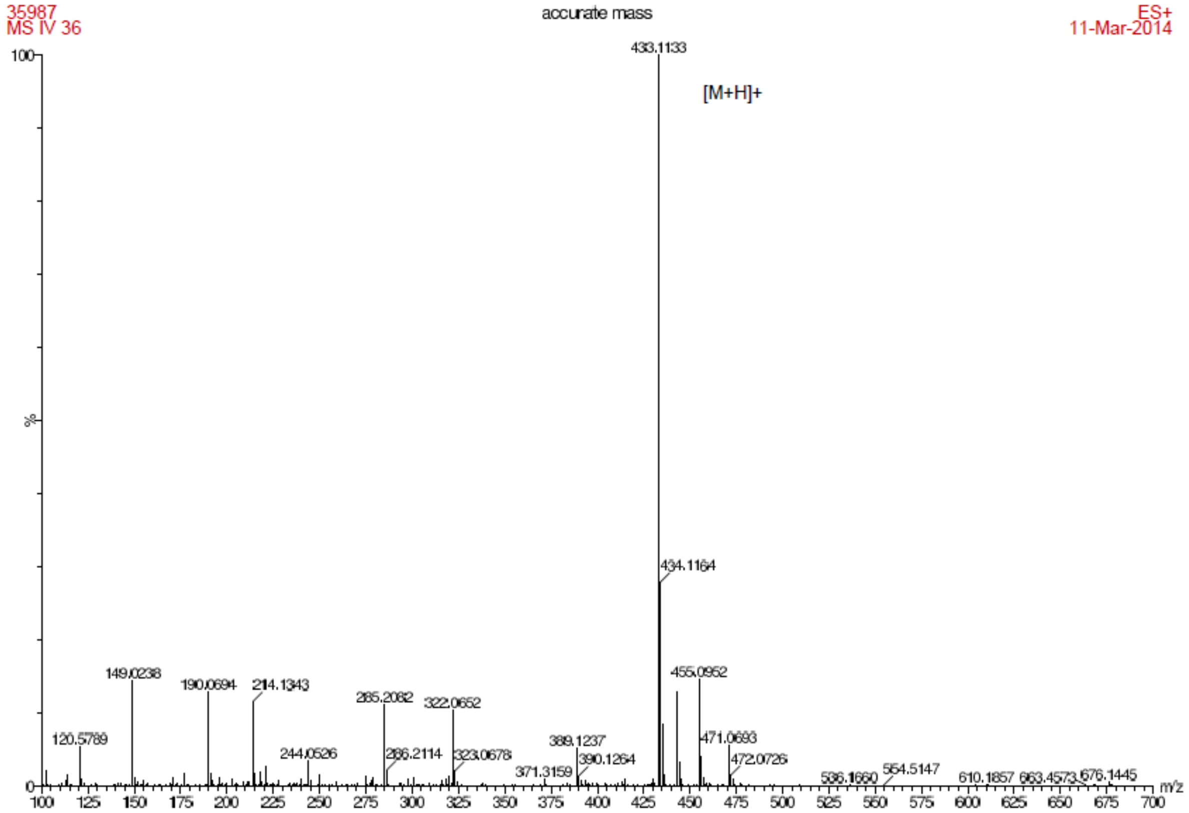

^{1}H NMR (600 MHz, $CDCl_3$)

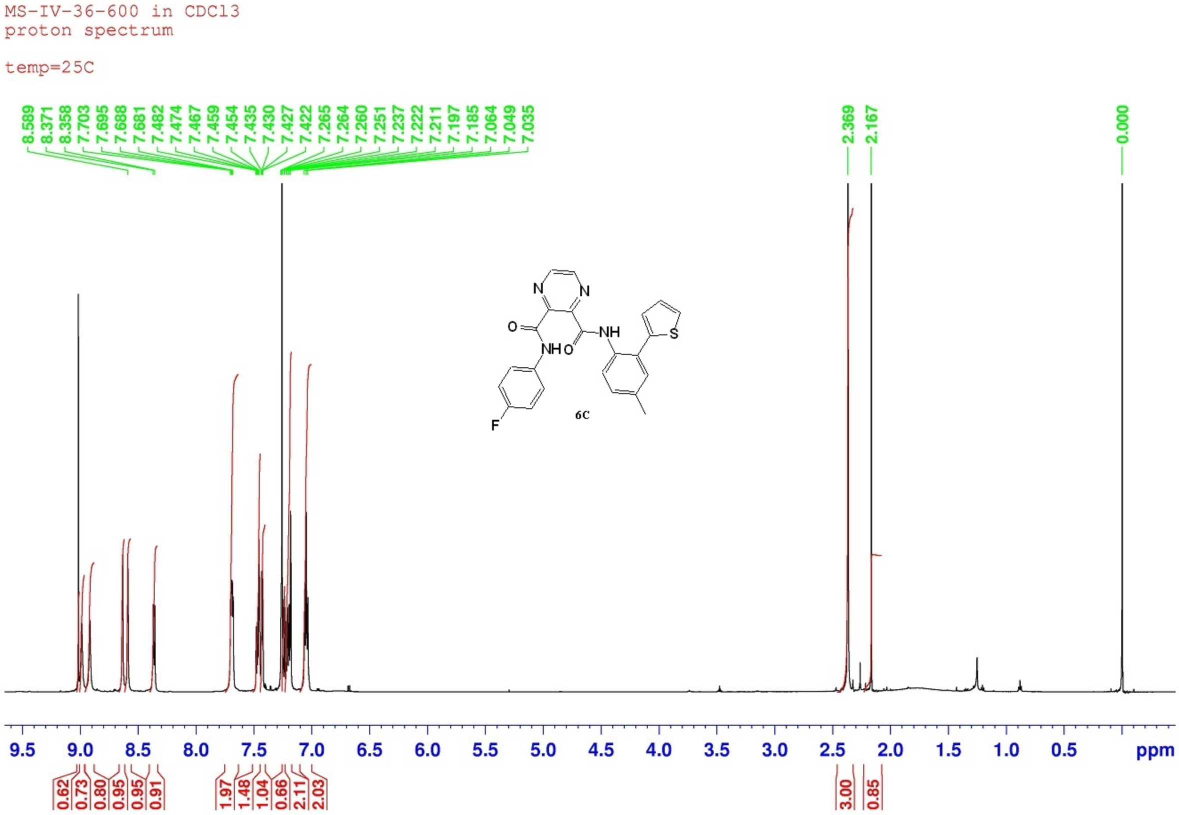

^{13}C NMR (150 MHz, $CDCl_3$)

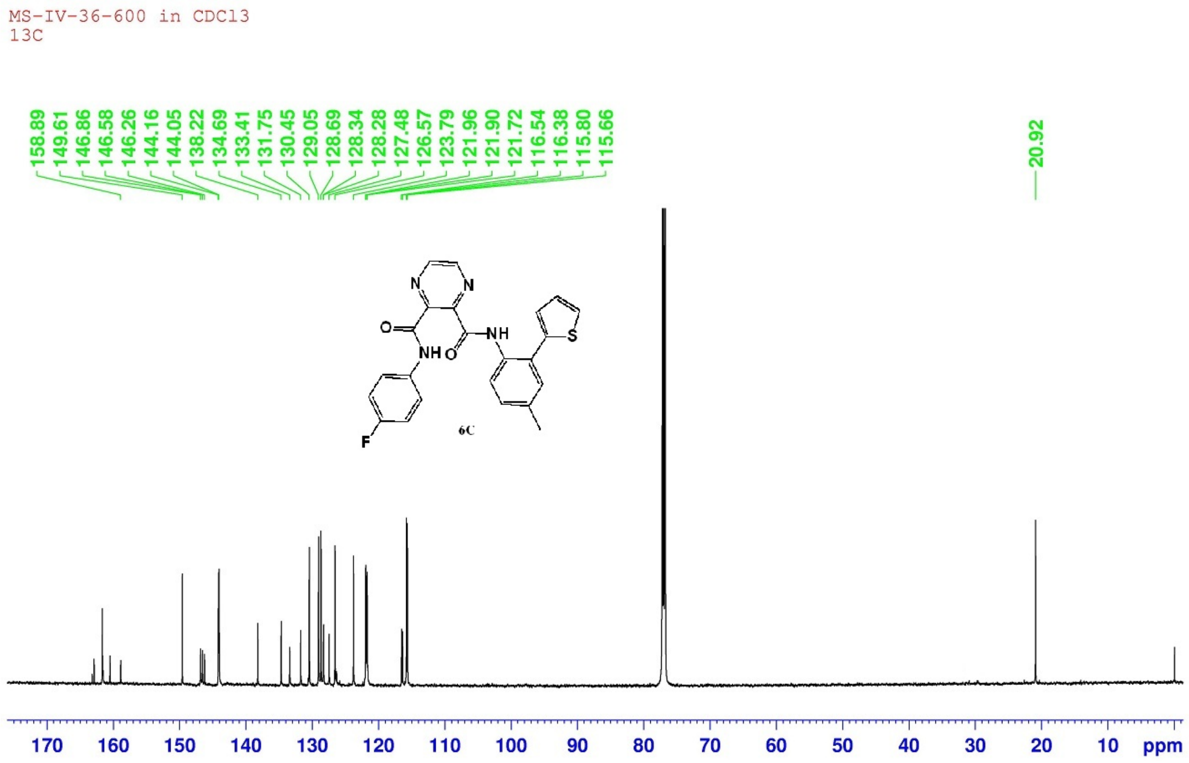

N^2-(4-methyl-2-(thiophen-2-yl)phenyl)-N^3-(p-tolyl)pyrazine-2,3-dicarboxamide (6d)

HRMS

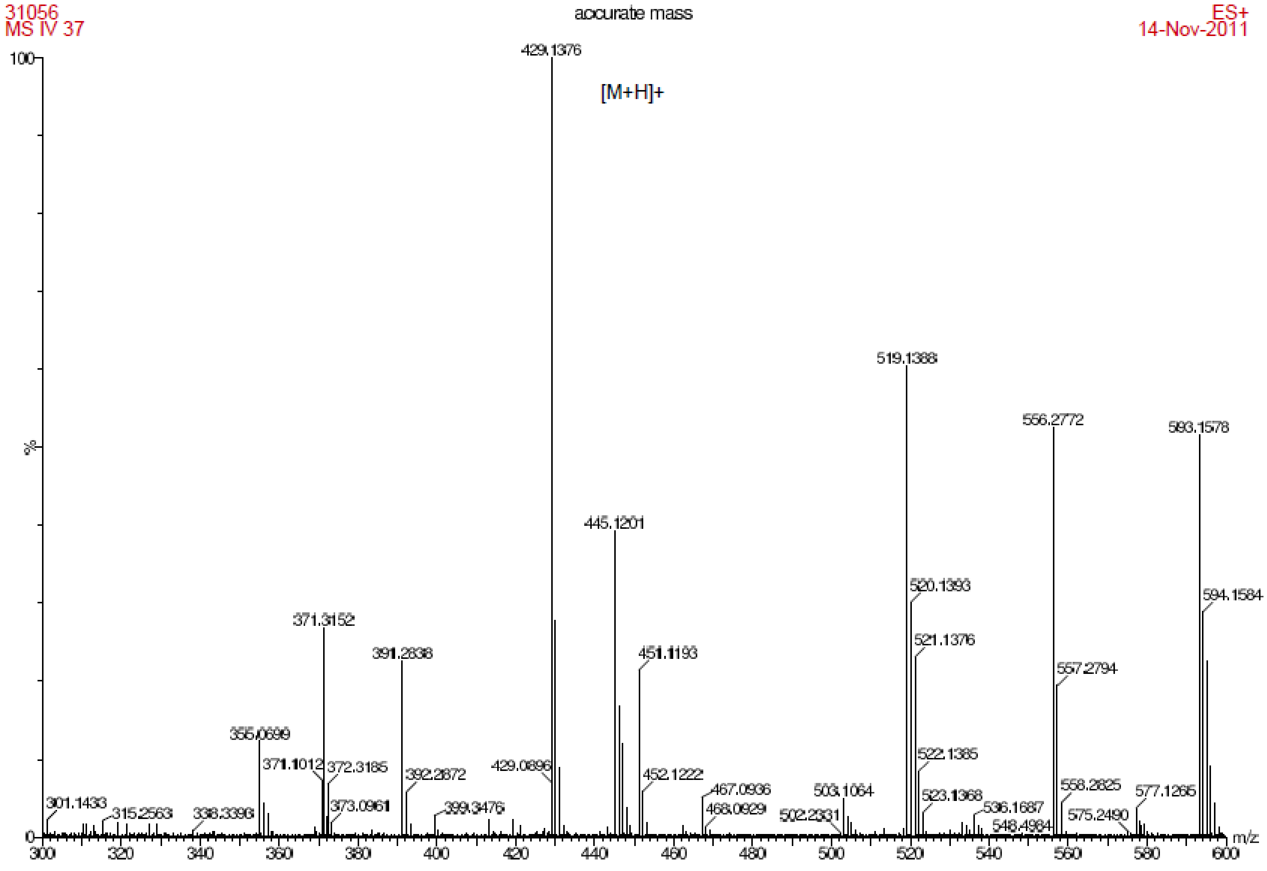

^{1}H NMR (600 MHz, $CDCl_3$)

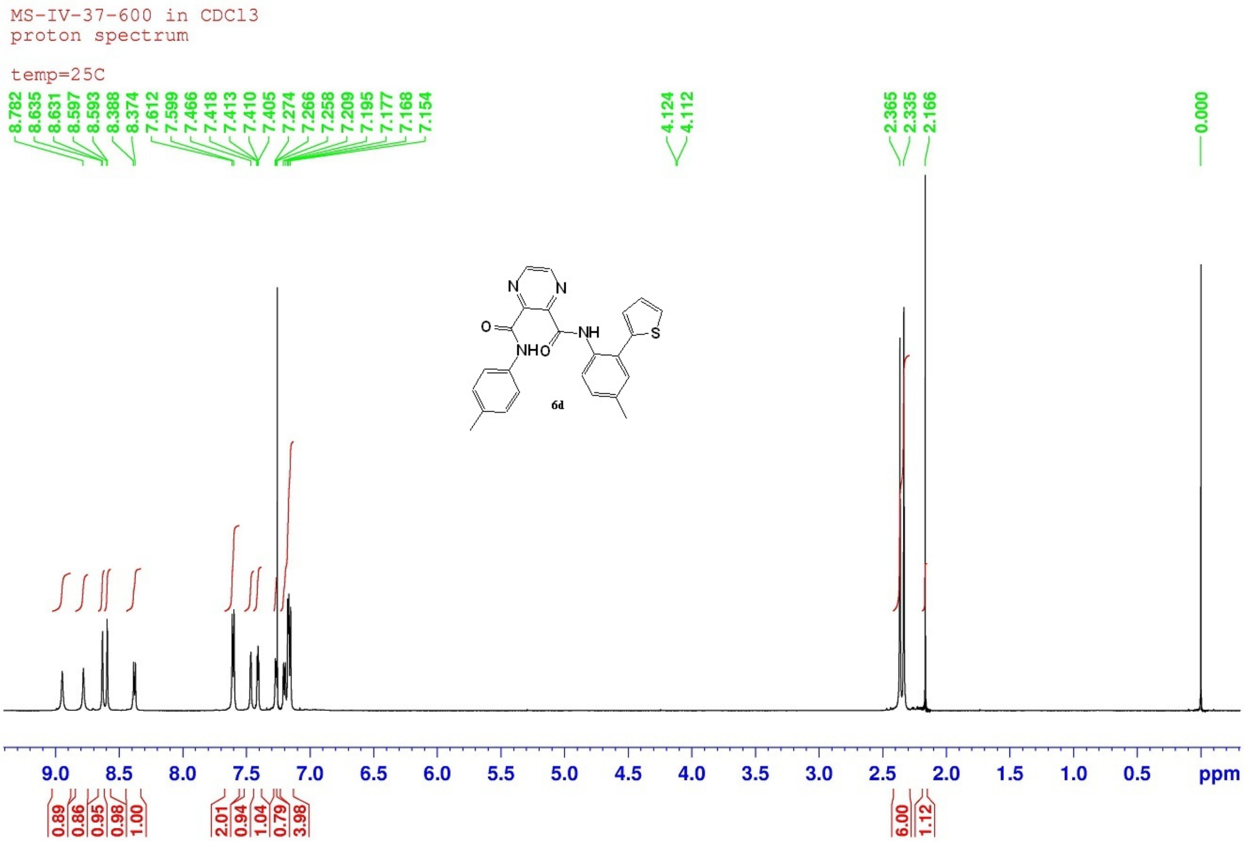

^{13}C NMR (150 MHz, $CDCl_3$)

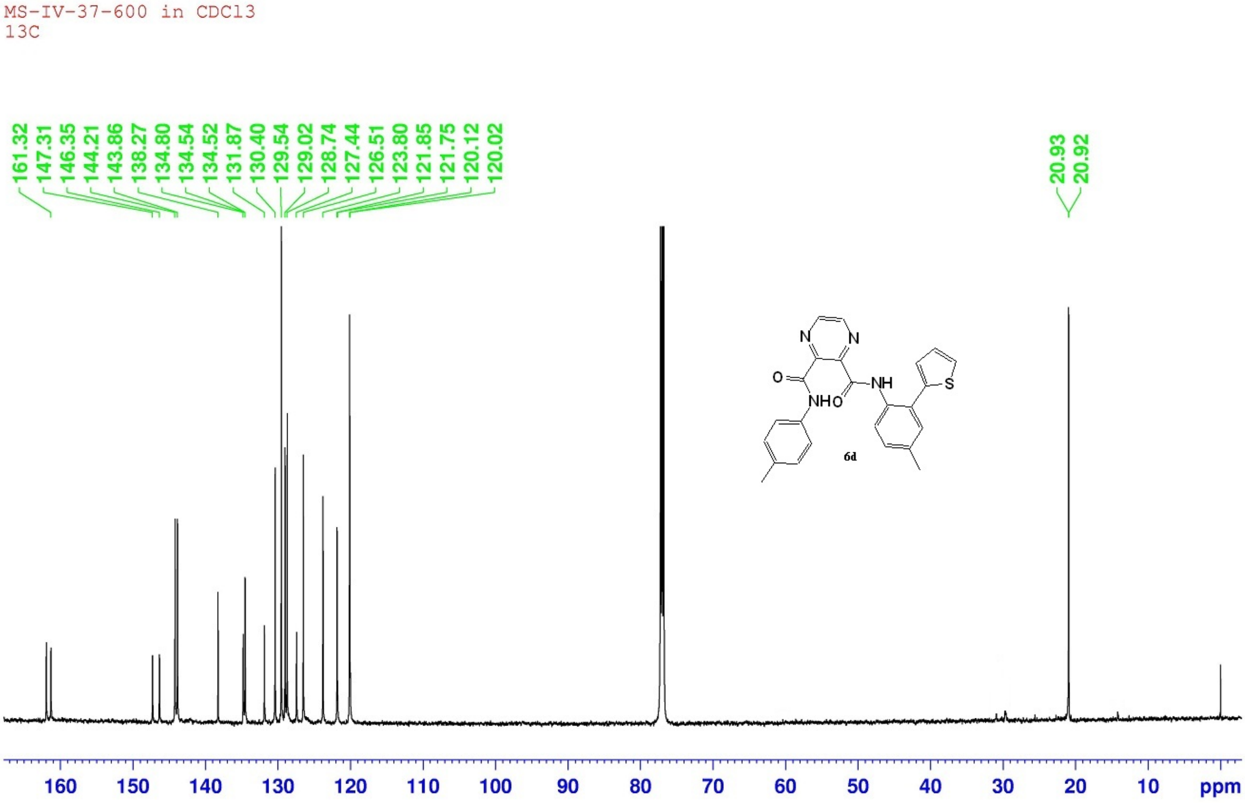

N^2-(4-fluorophenyl)-N^3-(4-methyl-2-(5-methylthiophen-2-yl)phenyl)pyrazine-2,3-dicarboxamide (6e)

HRMS

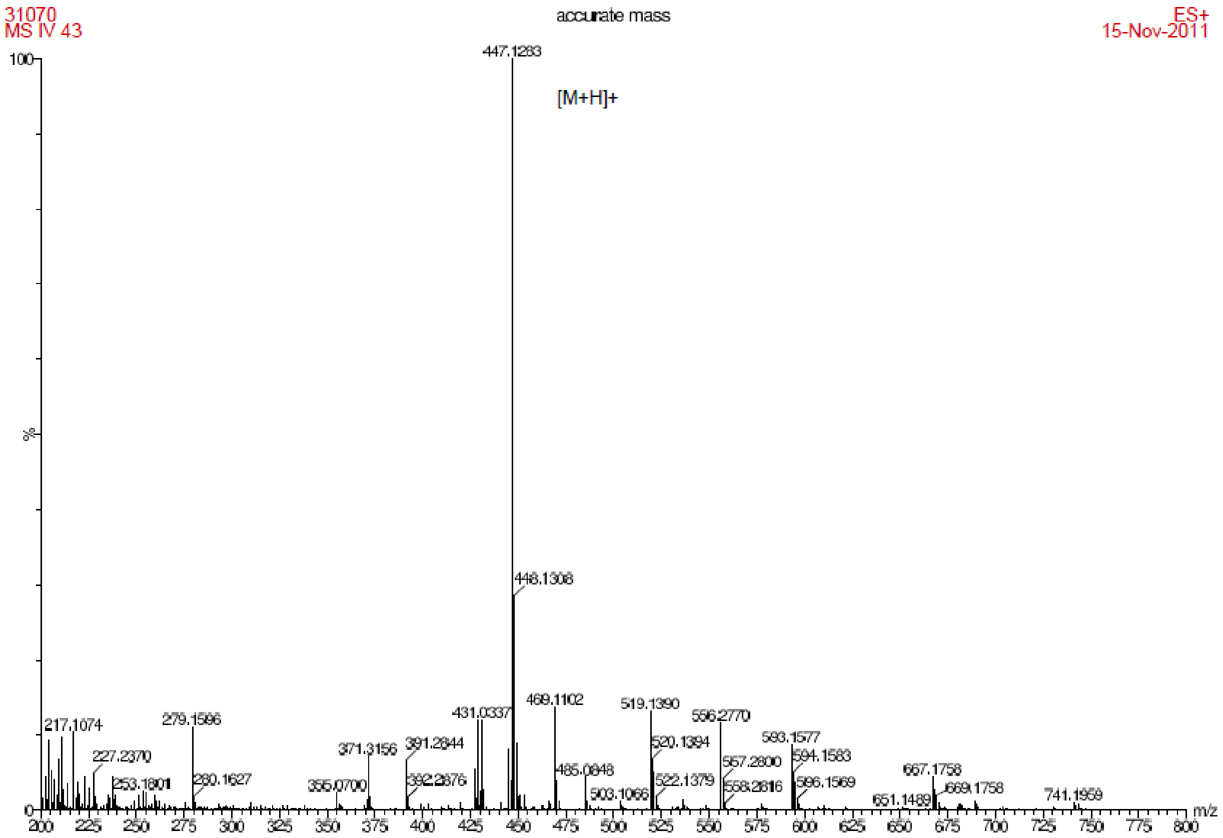

^{1}H NMR (600 MHz, $CDCl_3$)

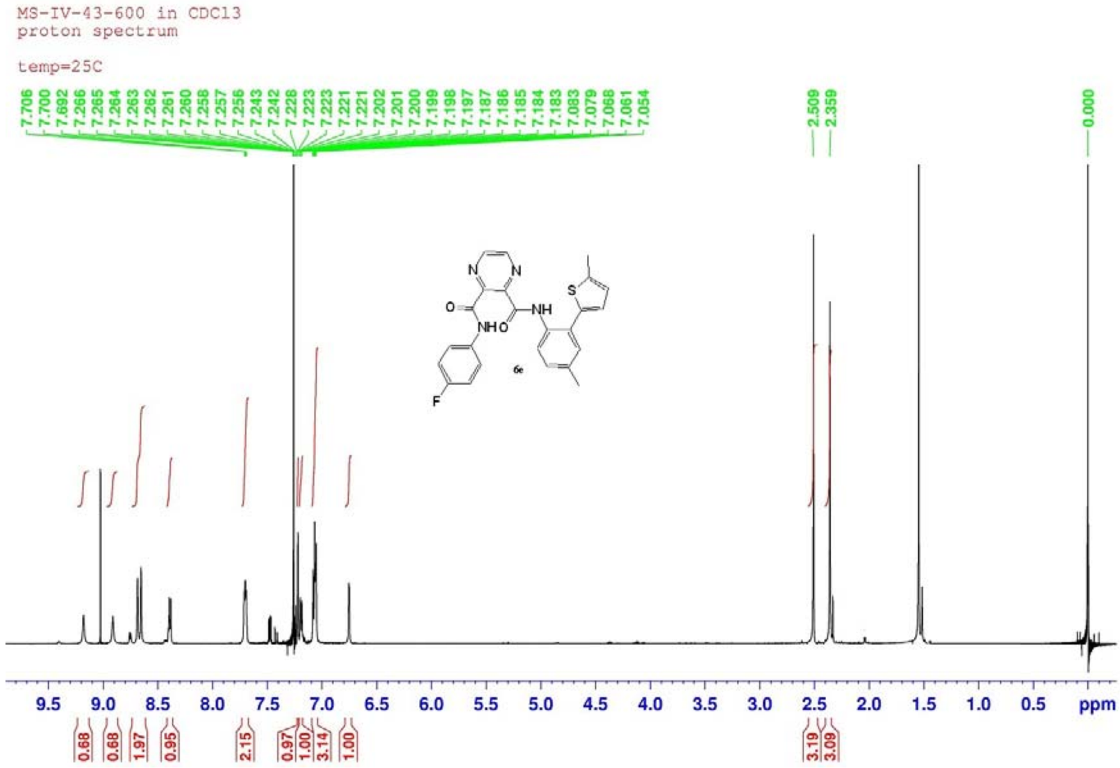

^{13}C NMR (150 MHz, $CDCl_3$)

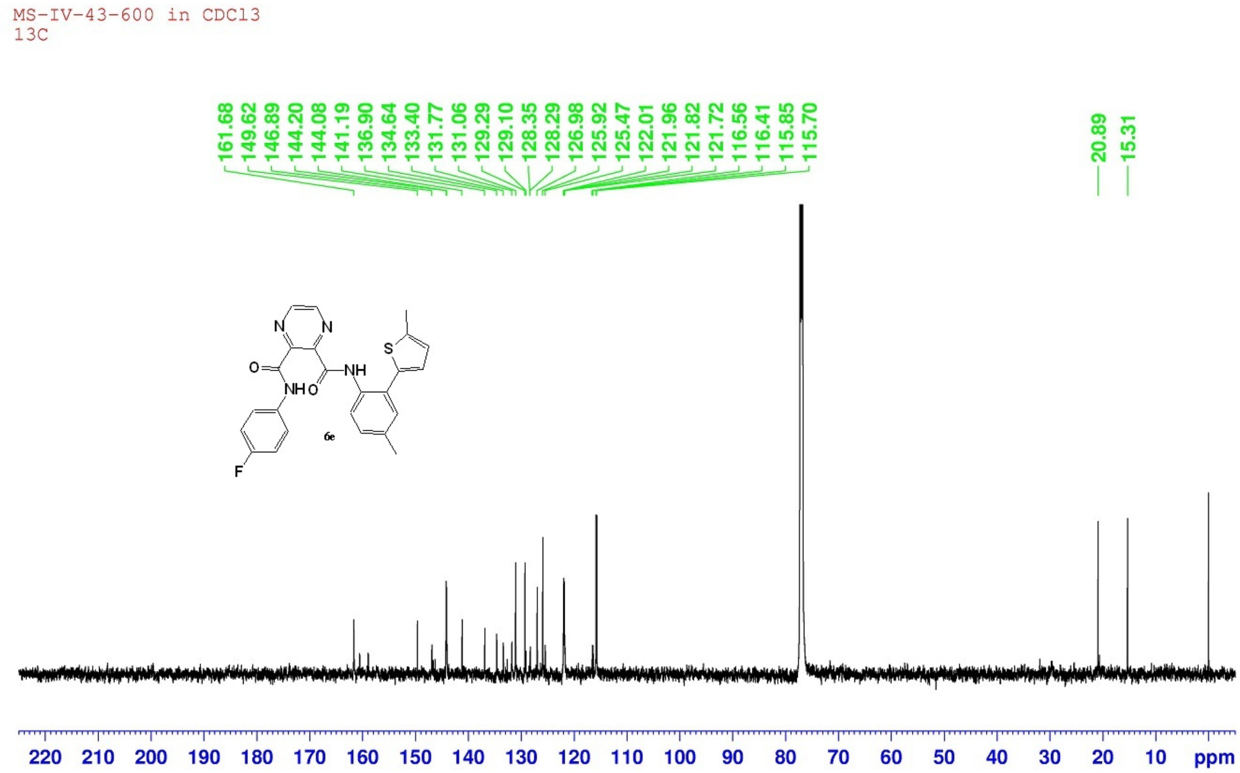

N[2]-(4-methyl-2-(5-methylthiophen-2-yl)phenyl)-*N*[3]-(p-tolyl)pyrazine-2,3-dicarboxamide (6f)

HRMS

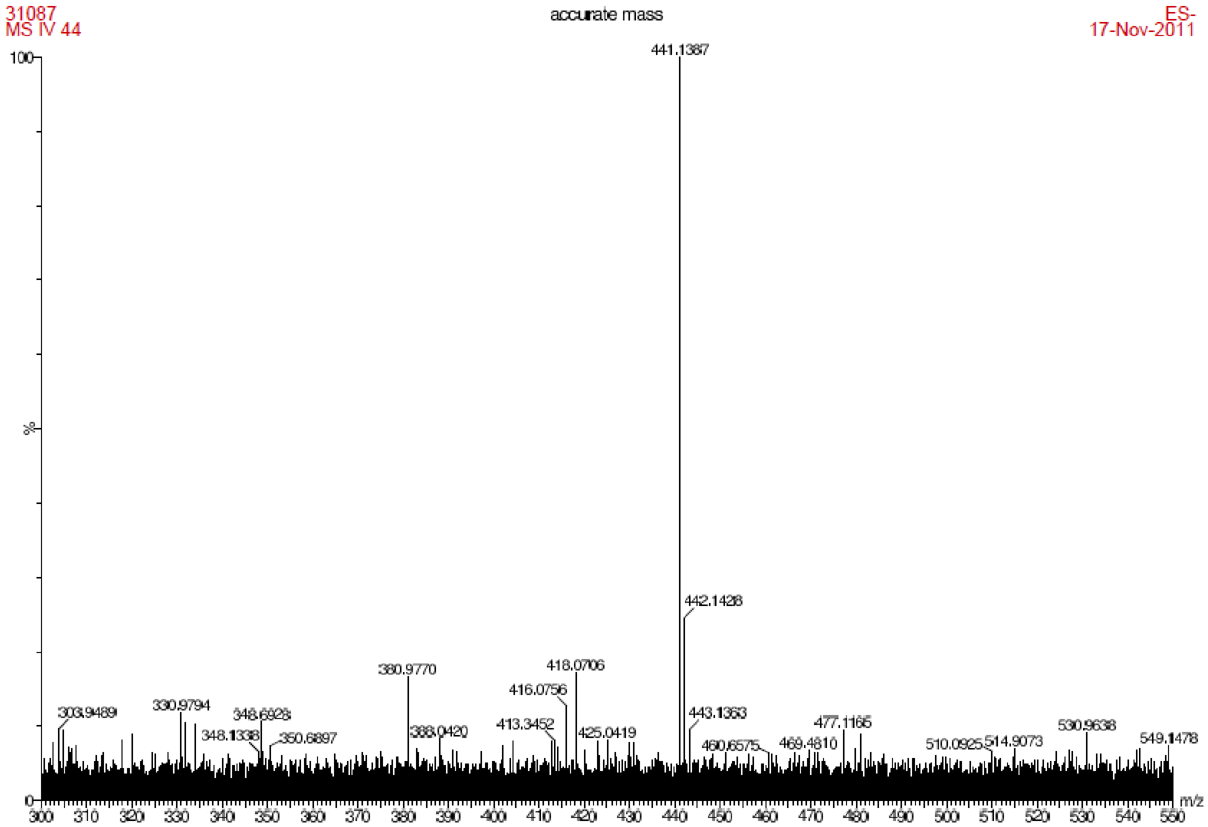

^{1}H NMR (600 MHz, $CDCl_3$)

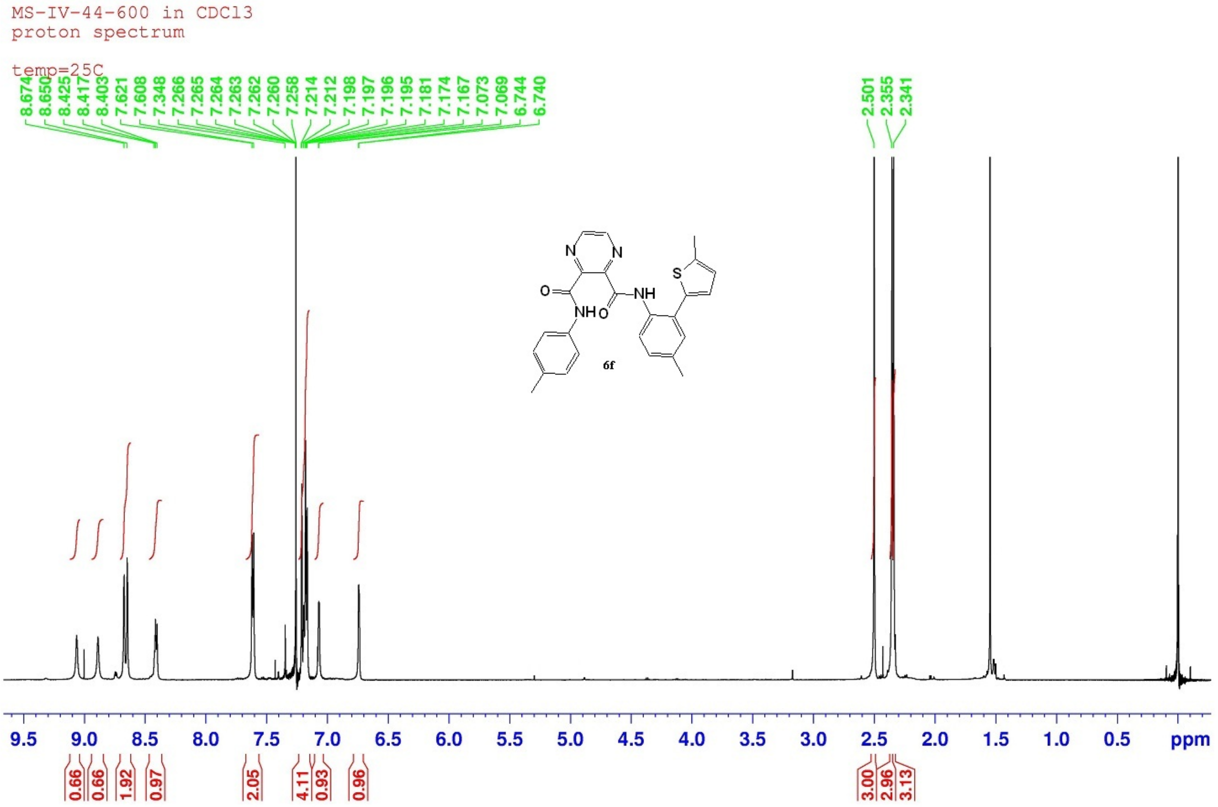

^{13}C NMR (150 MHz, $CDCl_3$)

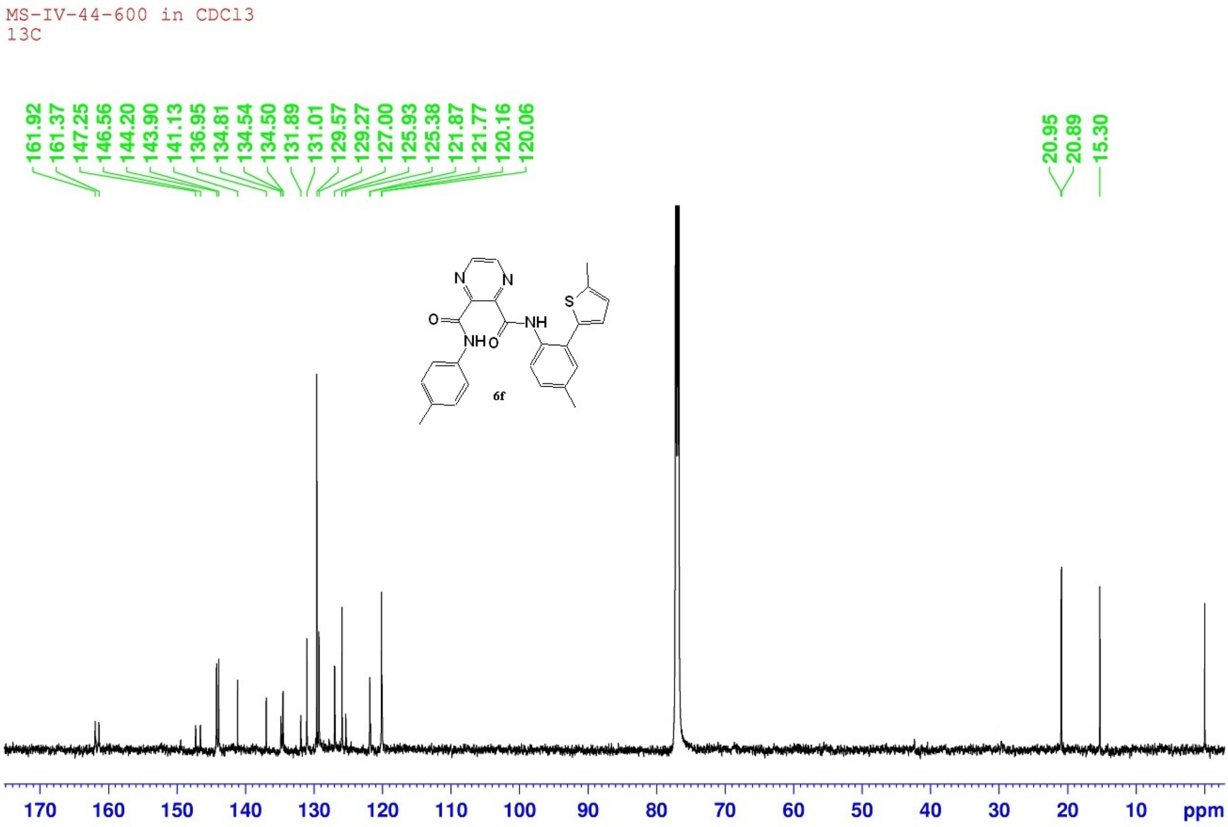

N^2-(4-fluorophenyl)-N^3-(4-methyl-2-(thiophen-3-yl)phenyl)pyrazine-2,3-dicarbox-amide (6g)

HRMS

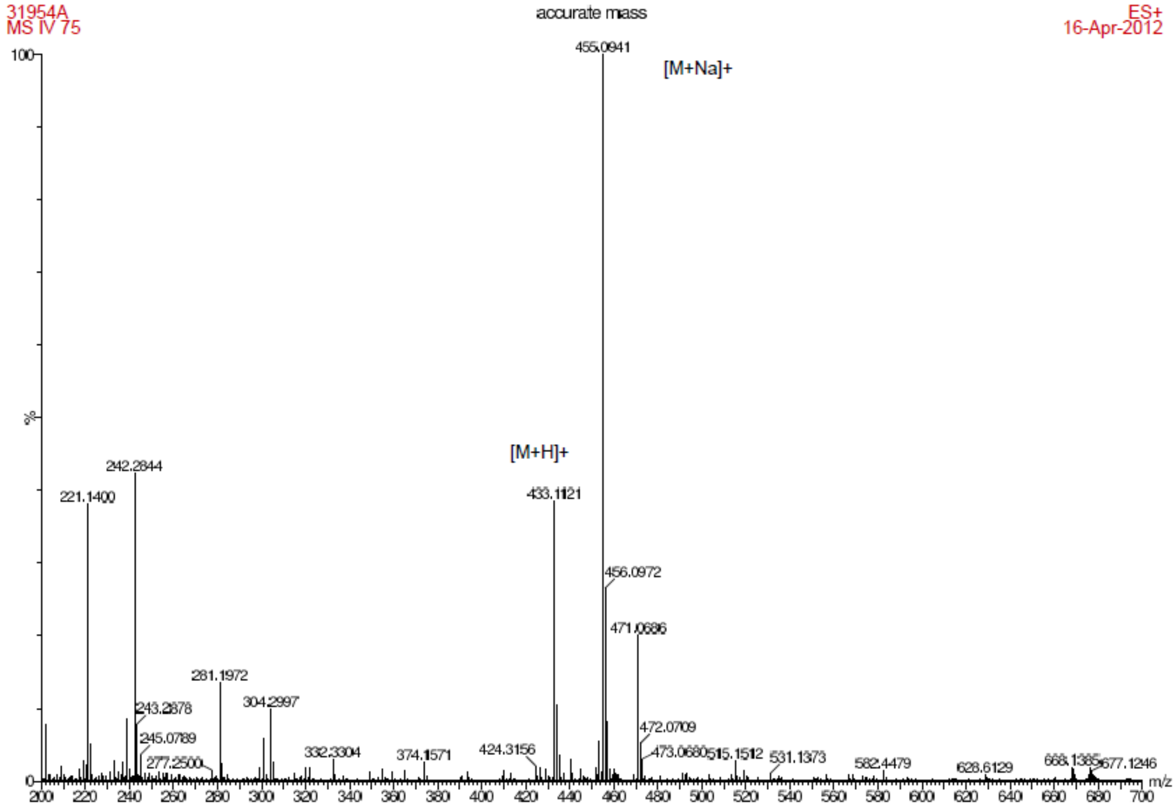

^1H NMR (500 MHz, $CDCl_3$)

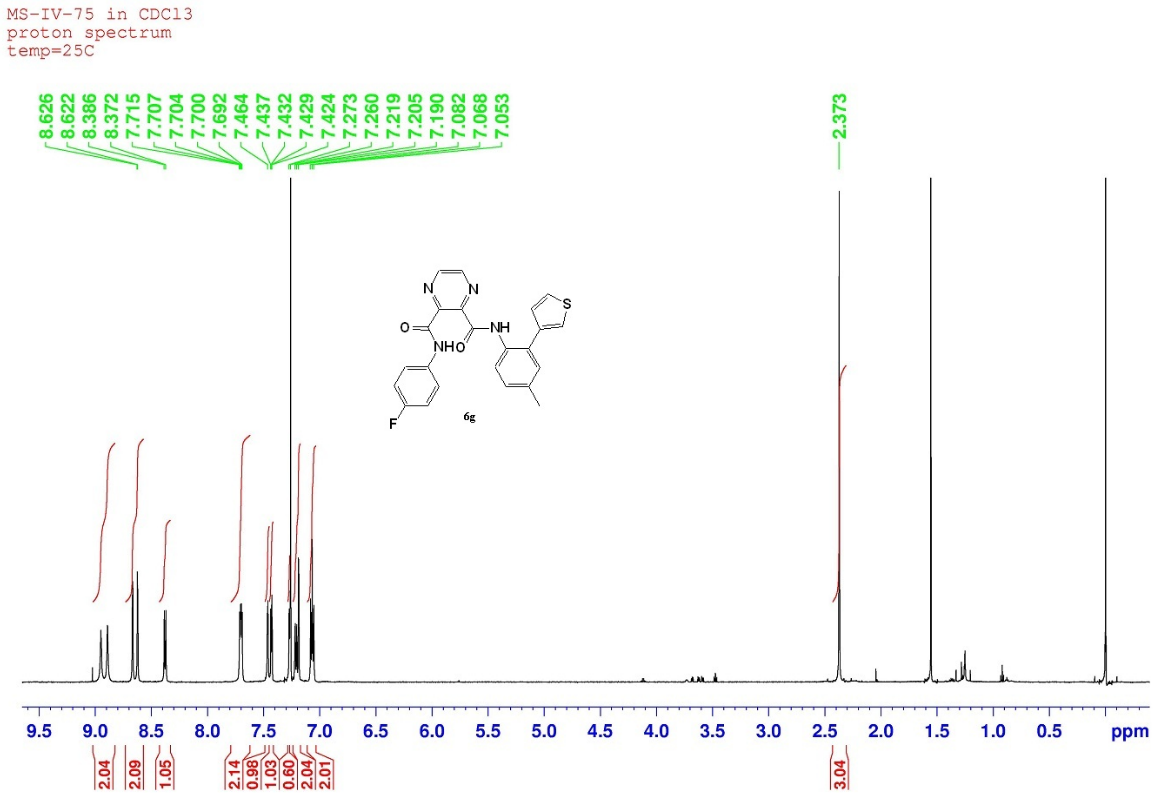

^{13}C NMR (125 MHz, CDCl$_3$)

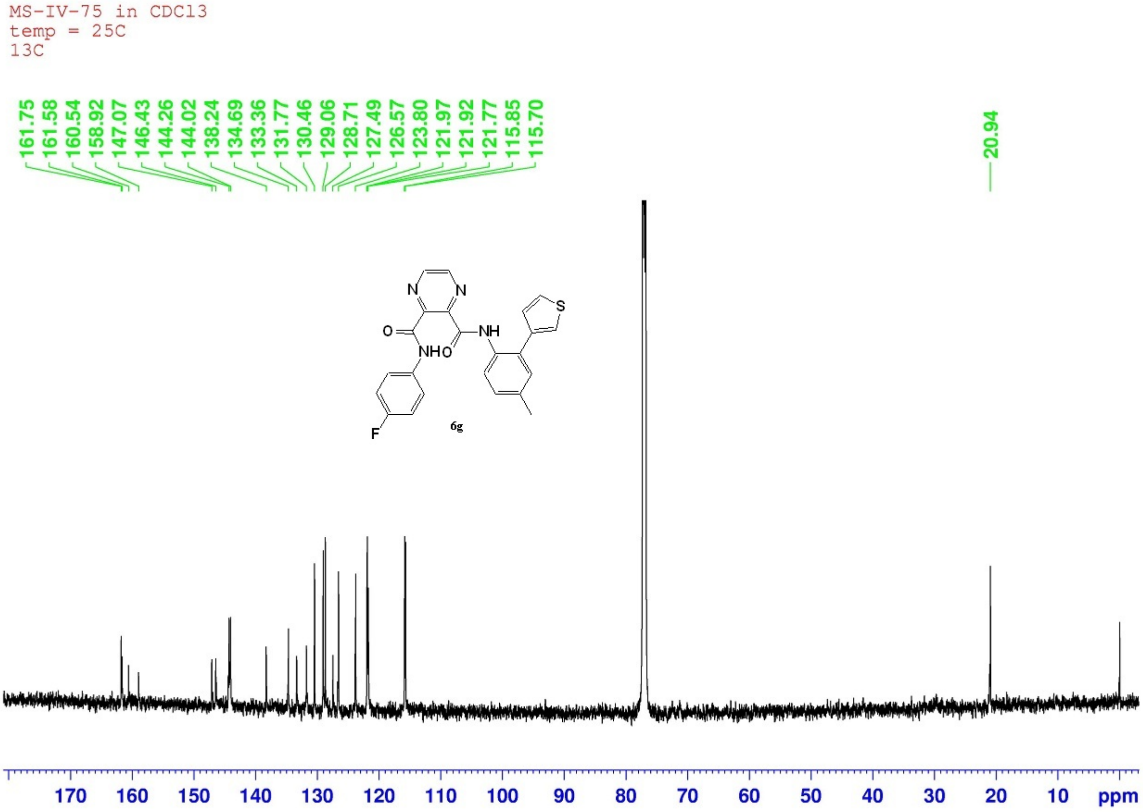

N^2-(4-methyl-2-(thiophen-3-yl)phenyl)-N^3-(p-tolyl)pyrazine-2,3-dicarboxamide (6h)

HRMS

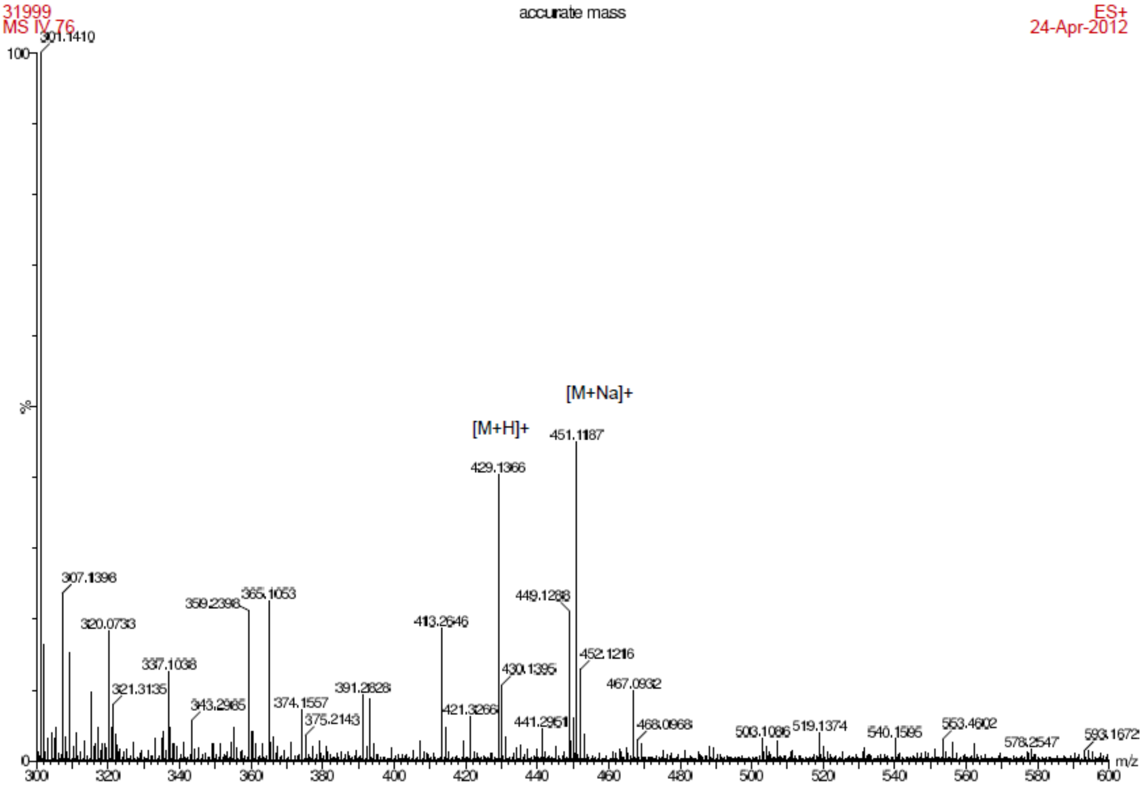

^{1}H NMR (500 MHz, $CDCl_3$)

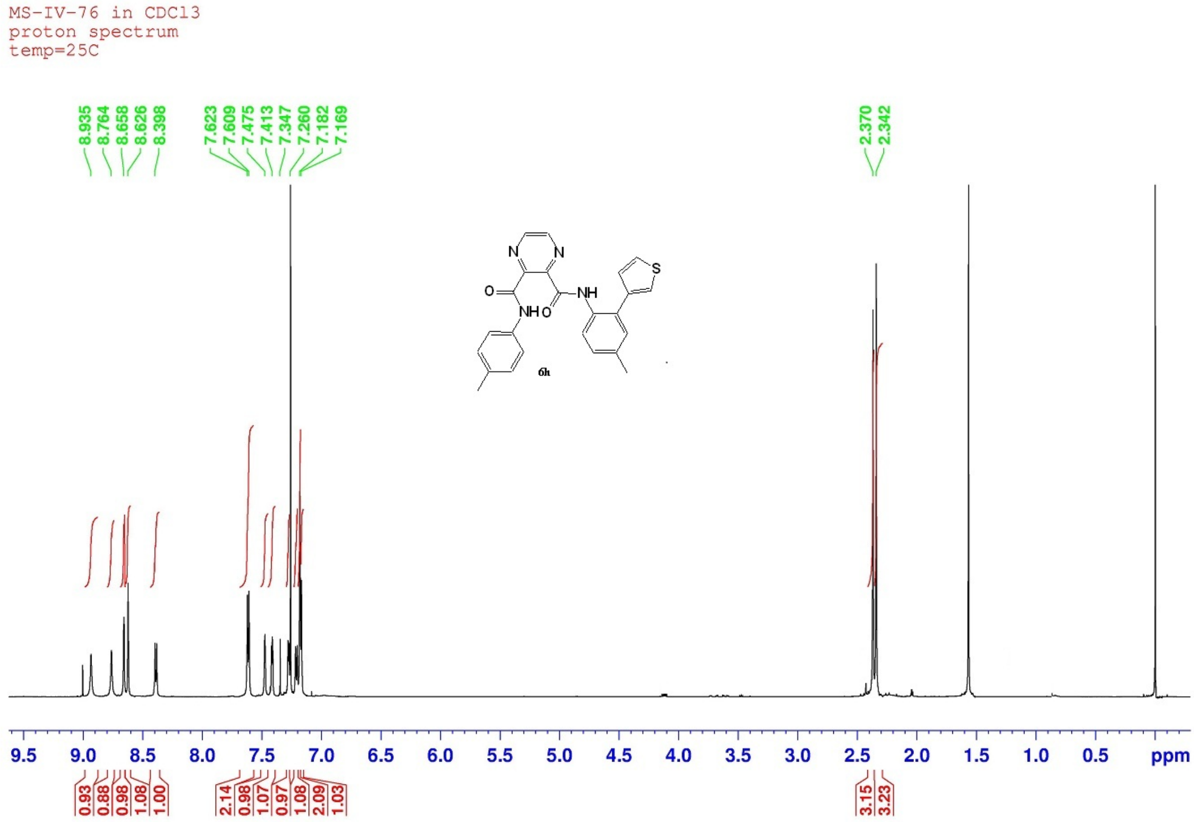

^{13}C NMR (125 MHz, $CDCl_3$)

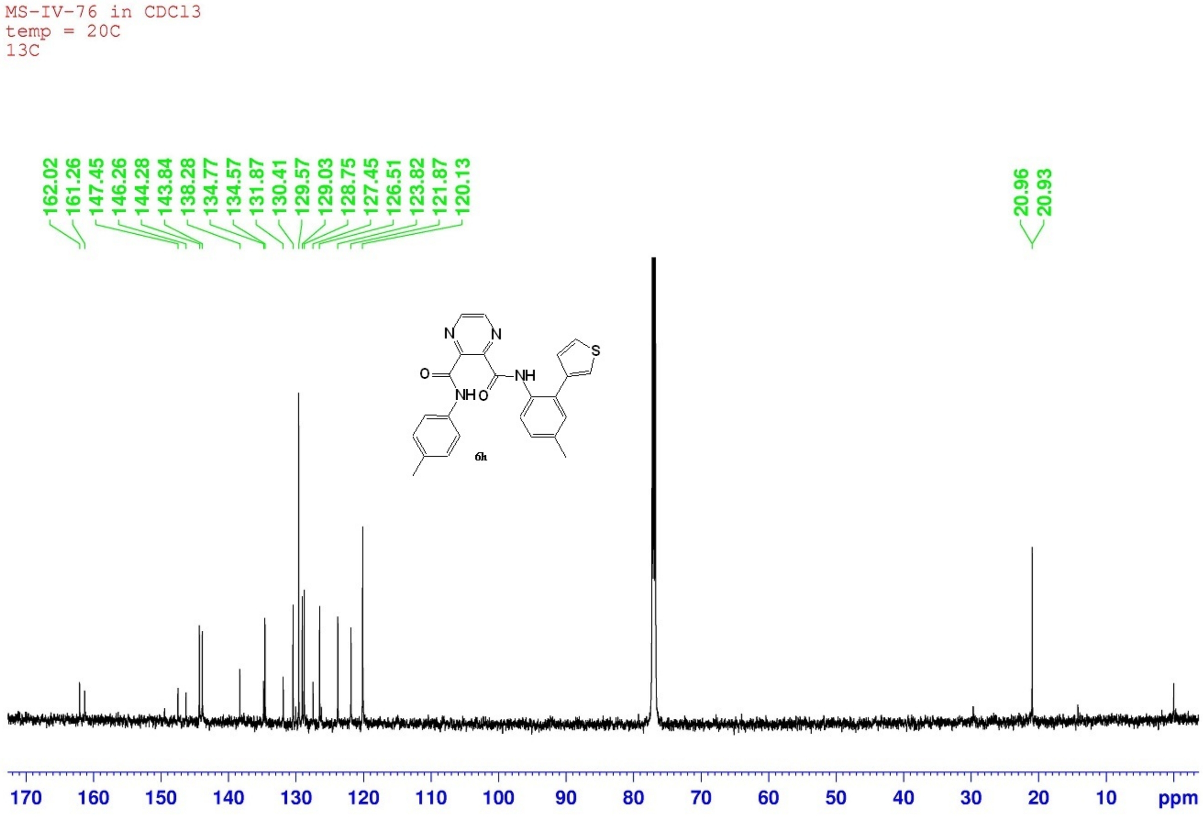

N[2]-(4-fluorophenyl)-*N*[3]-(2-(thiophen-3-yl)benzyl)pyrazine-2,3-dicarboxamide (6i)

HRMS

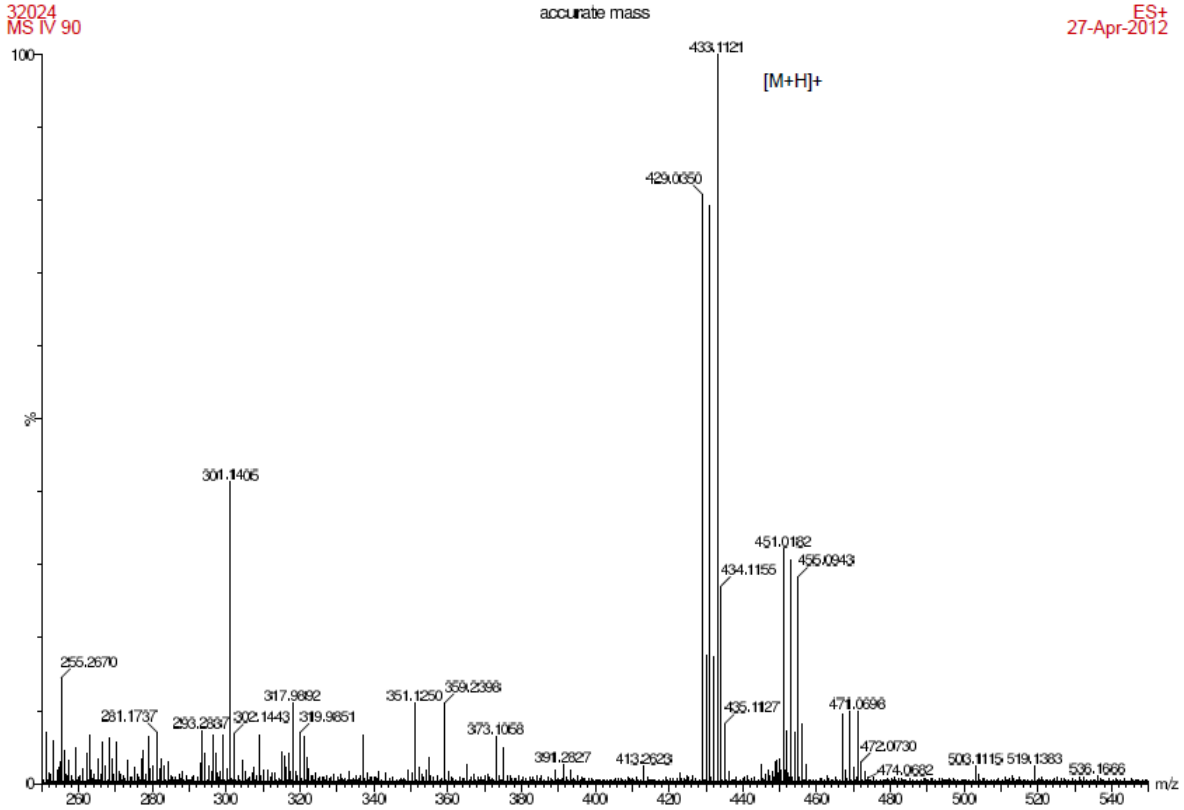

^{1}H NMR (300 MHz, DMSO)

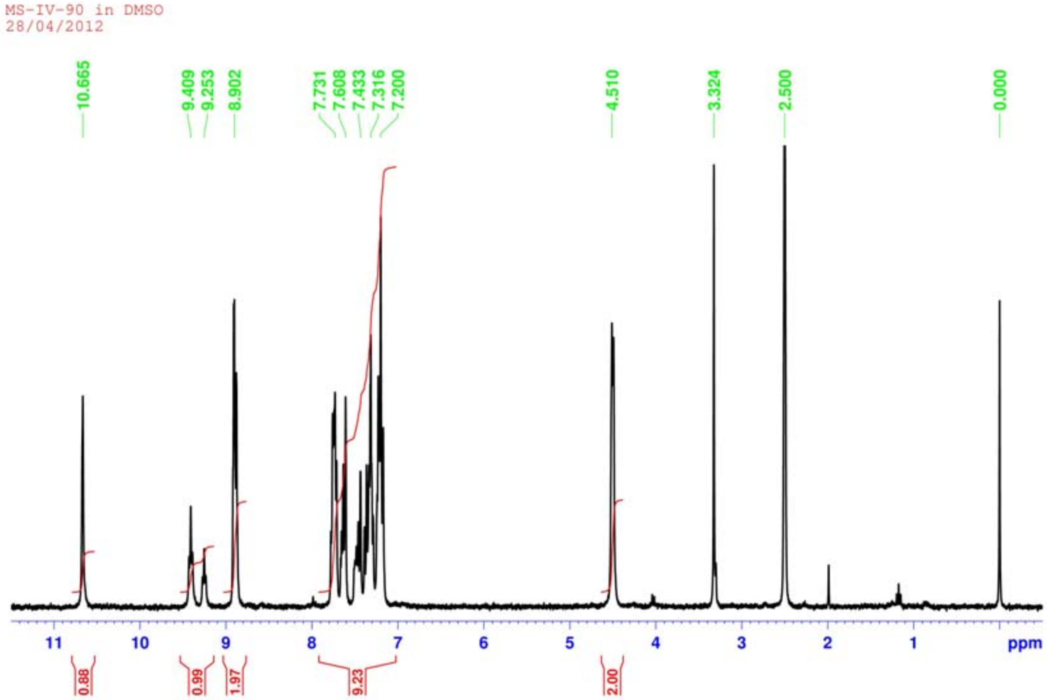

^{13}C NMR (75 MHz, DMSO)

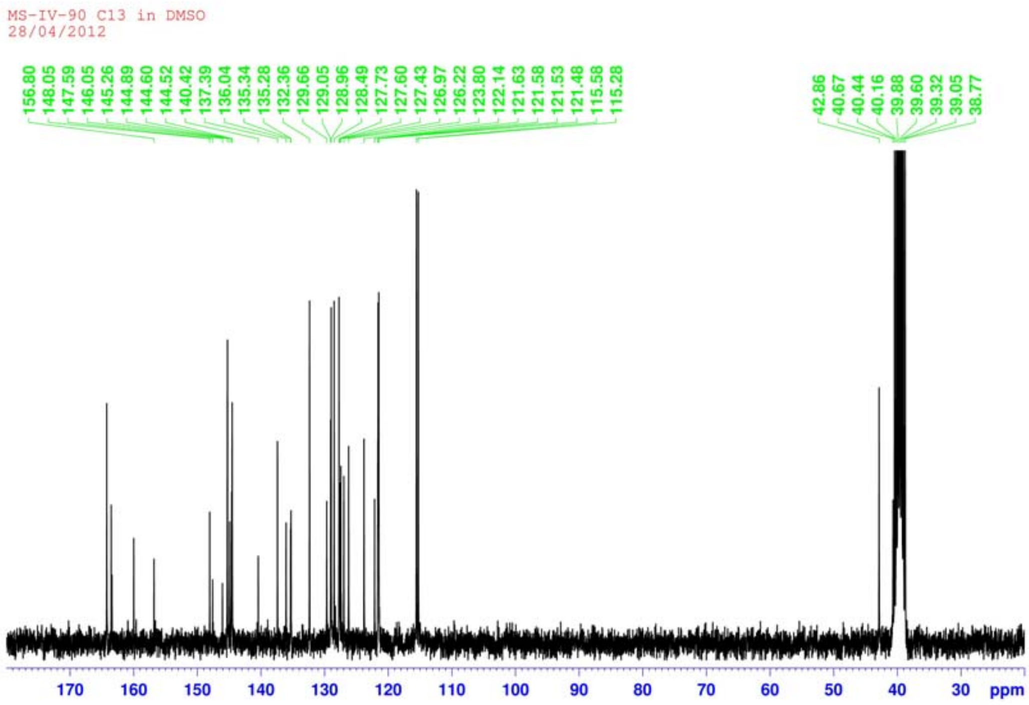

N^2-(2-(thiophen-3-yl)benzyl)-N^3-(p-tolyl)pyrazine-2,3-dicarboxamide (6j)
HRMS

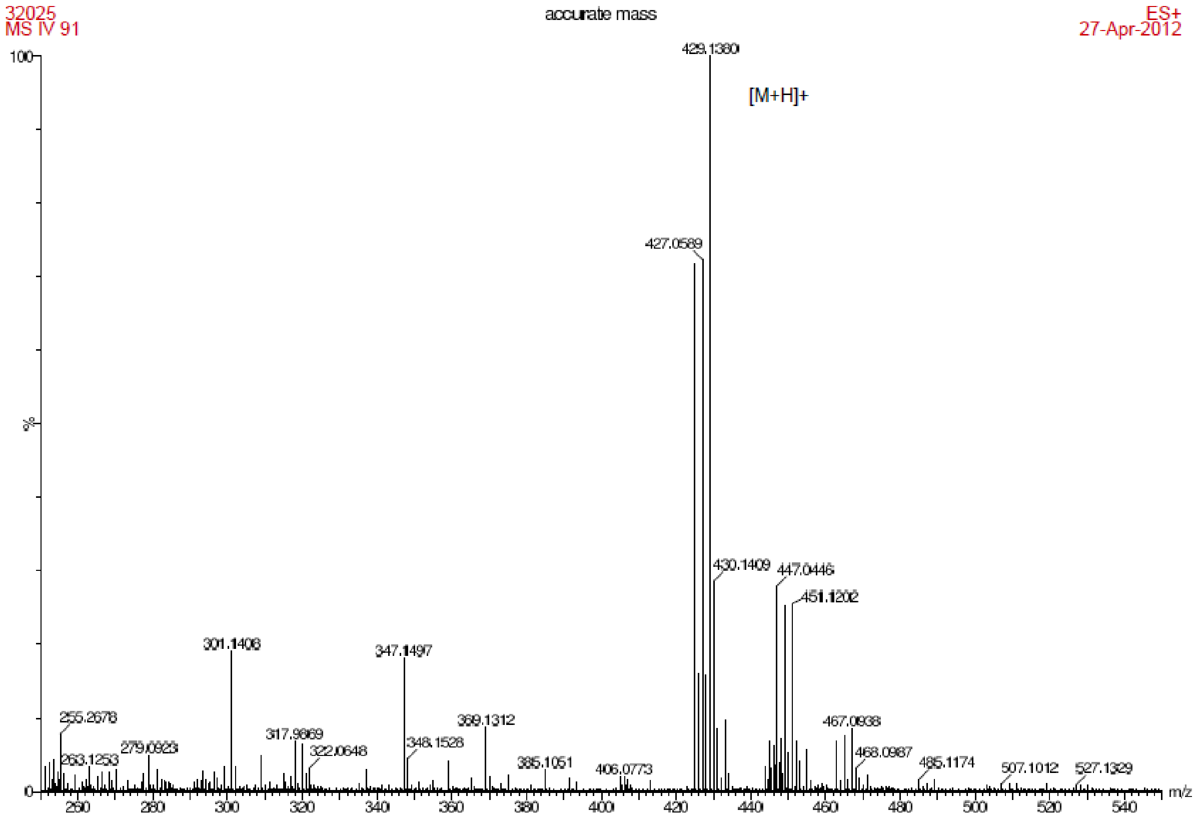

^{1}H NMR (300 MHz, DMSO)

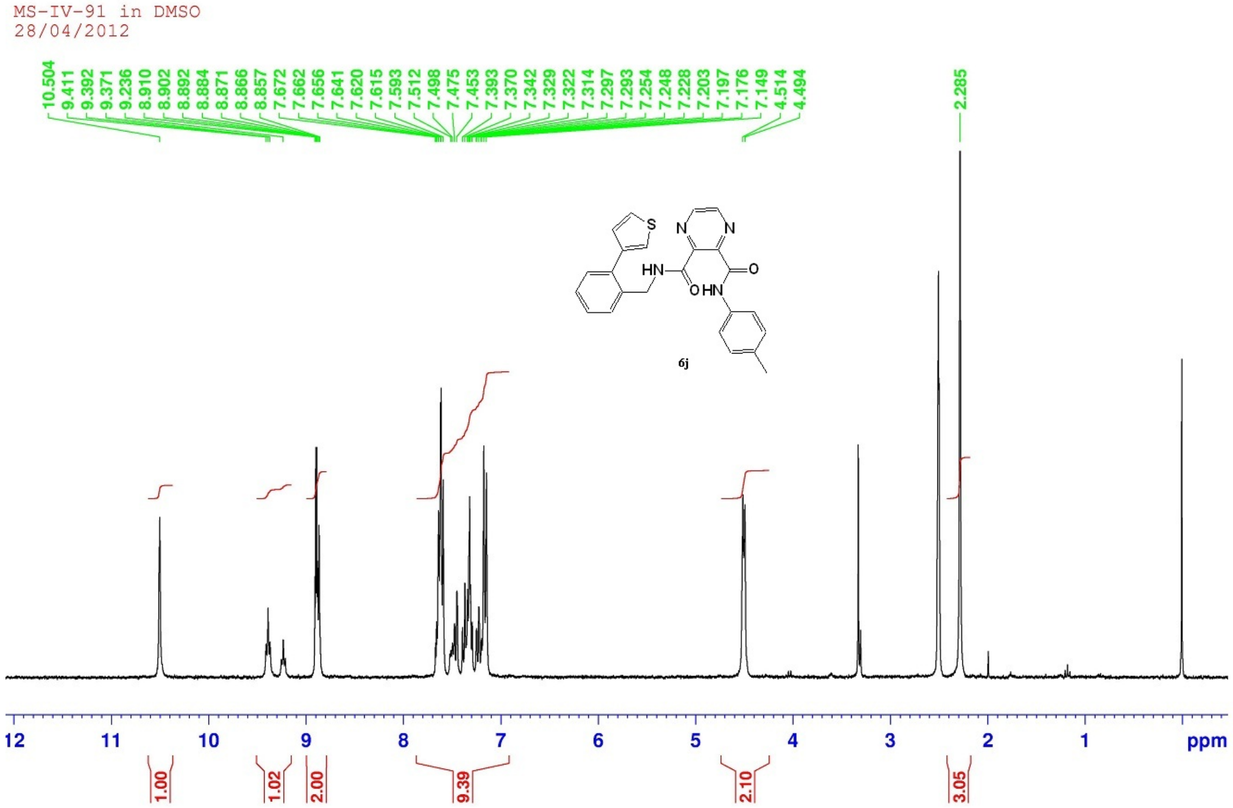

^{13}C NMR (75 MHz, DMSO)

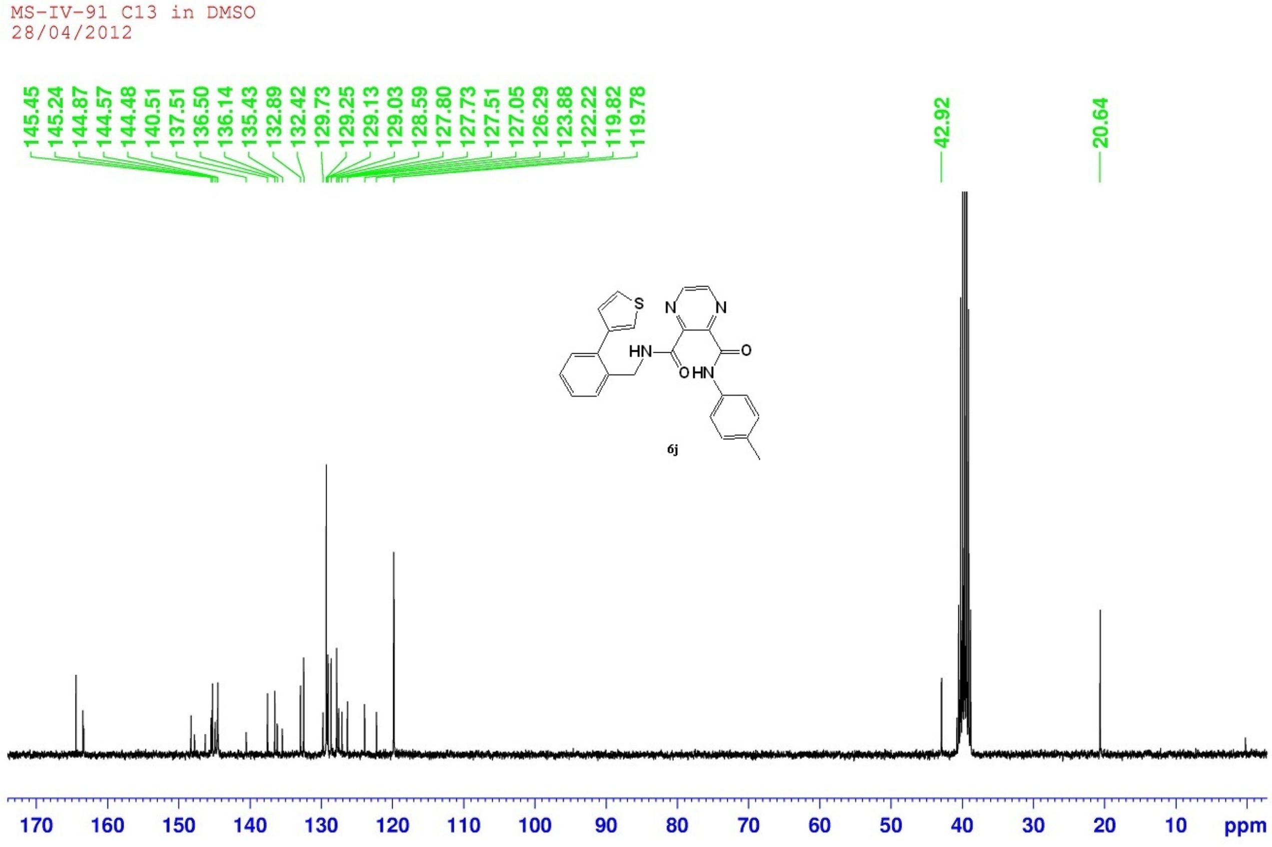

N^2-(4-fluorophenyl)-N^3-(4-(thiophen-3-yl)benzyl)pyrazine-2,3-dicarboxamide (6k)

HRMS

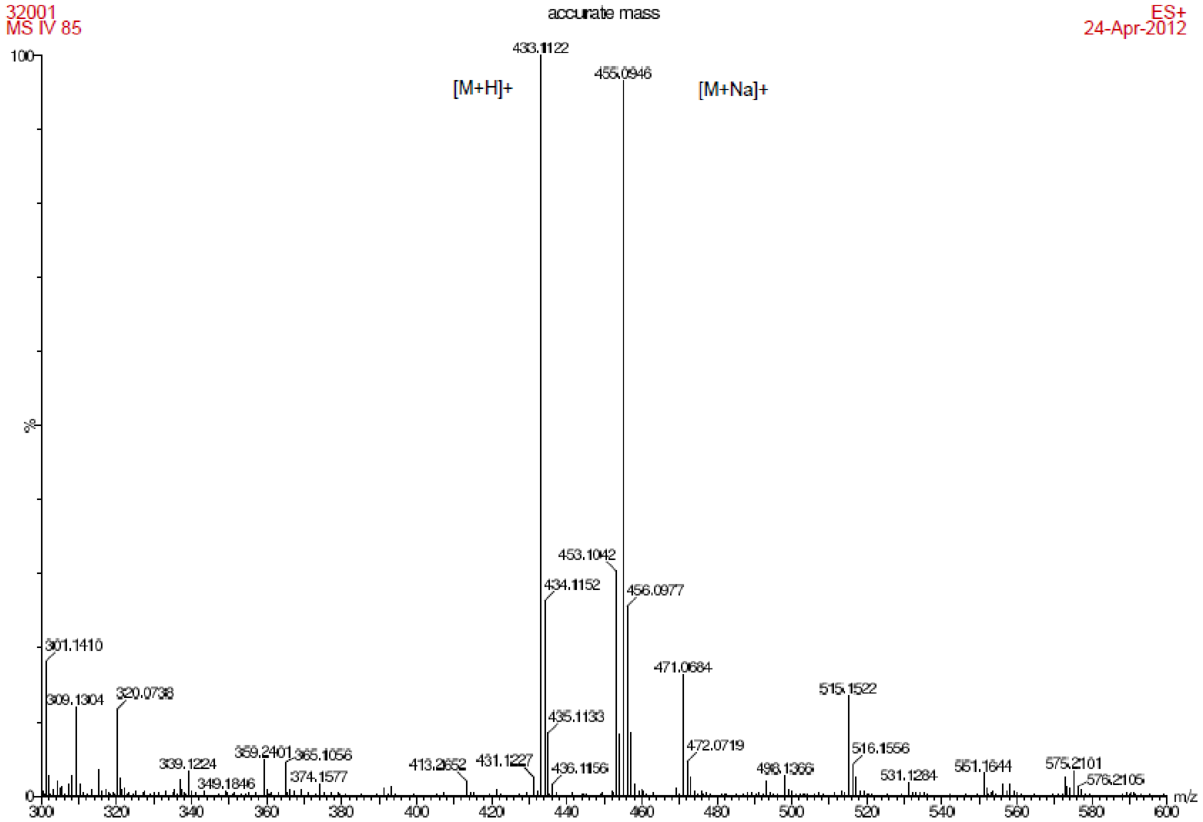

^{1}H NMR (300 MHz, DMSO)

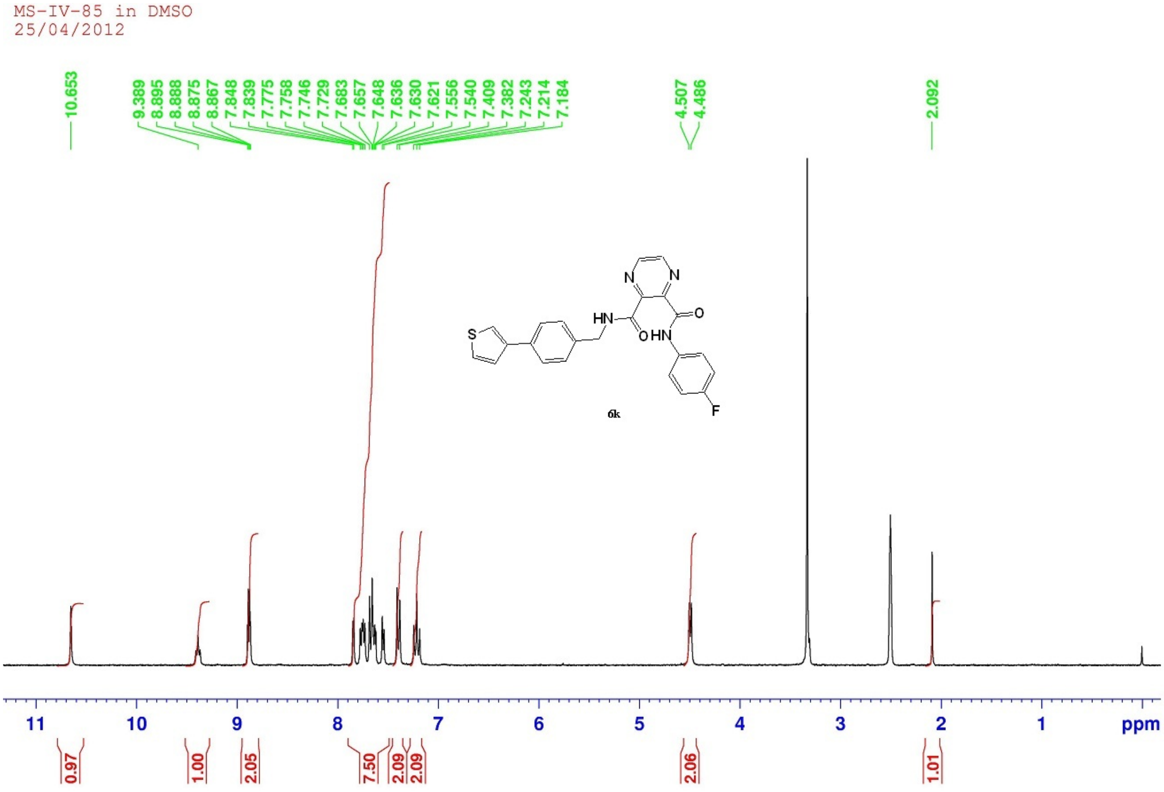

^{13}C NMR (75 MHz, DMSO)

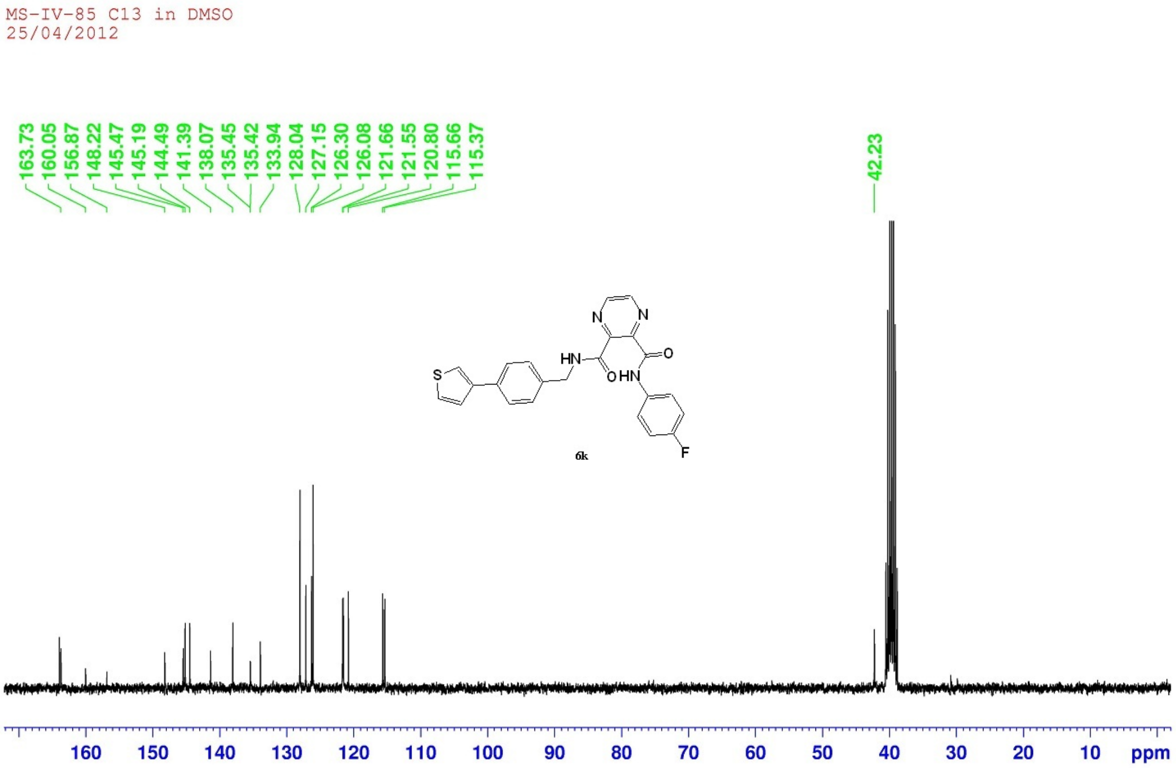

N^2-(4-(thiophen-3-yl)benzyl)-N^3-(p-tolyl)pyrazine-2,3-dicarboxamide (6l)
HRMS

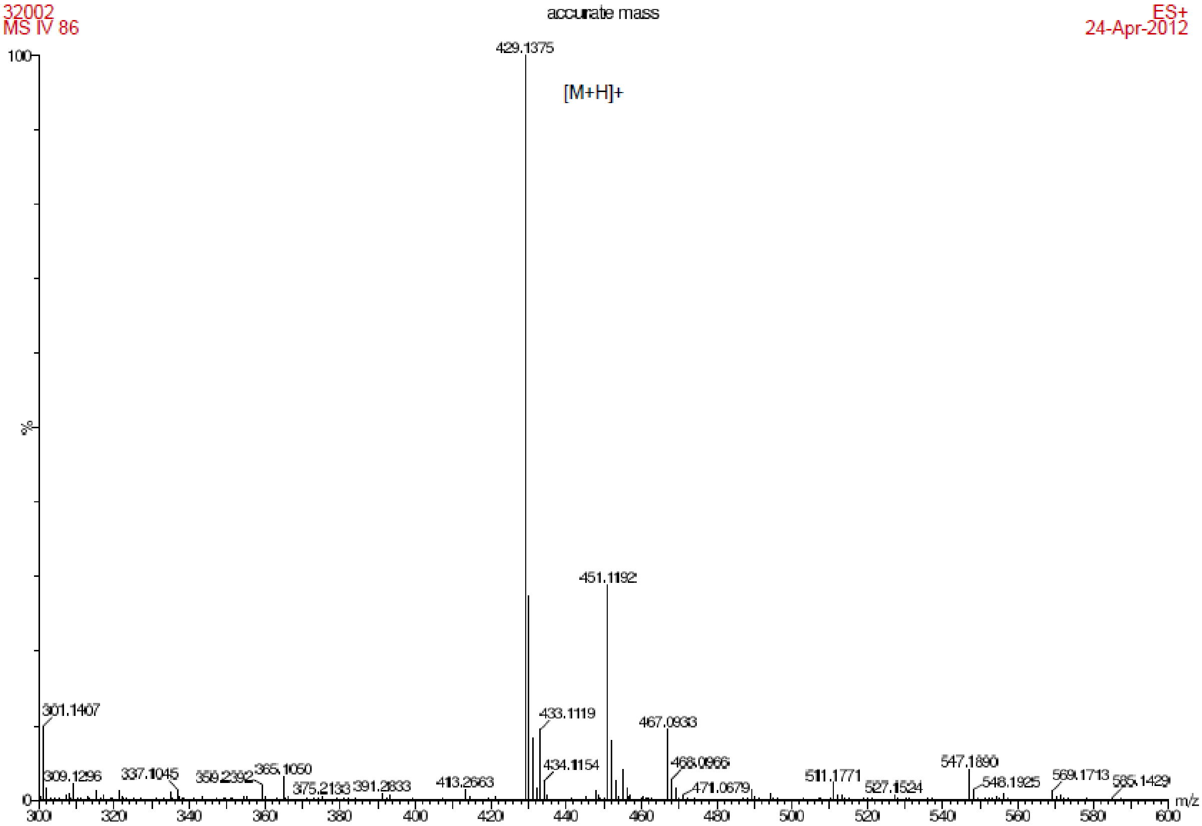

^{1}H NMR (300 MHz, DMSO)

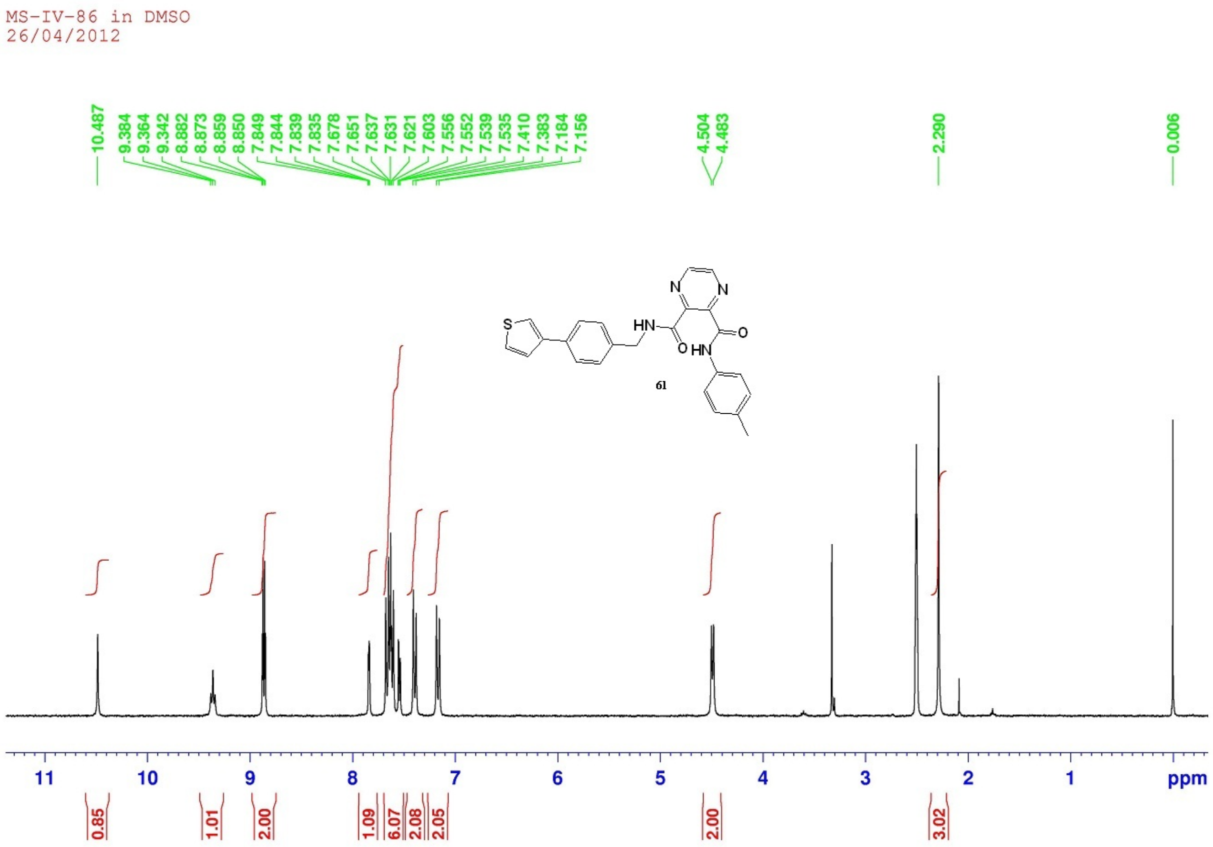

^{13}C NMR (75 MHz, DMSO)

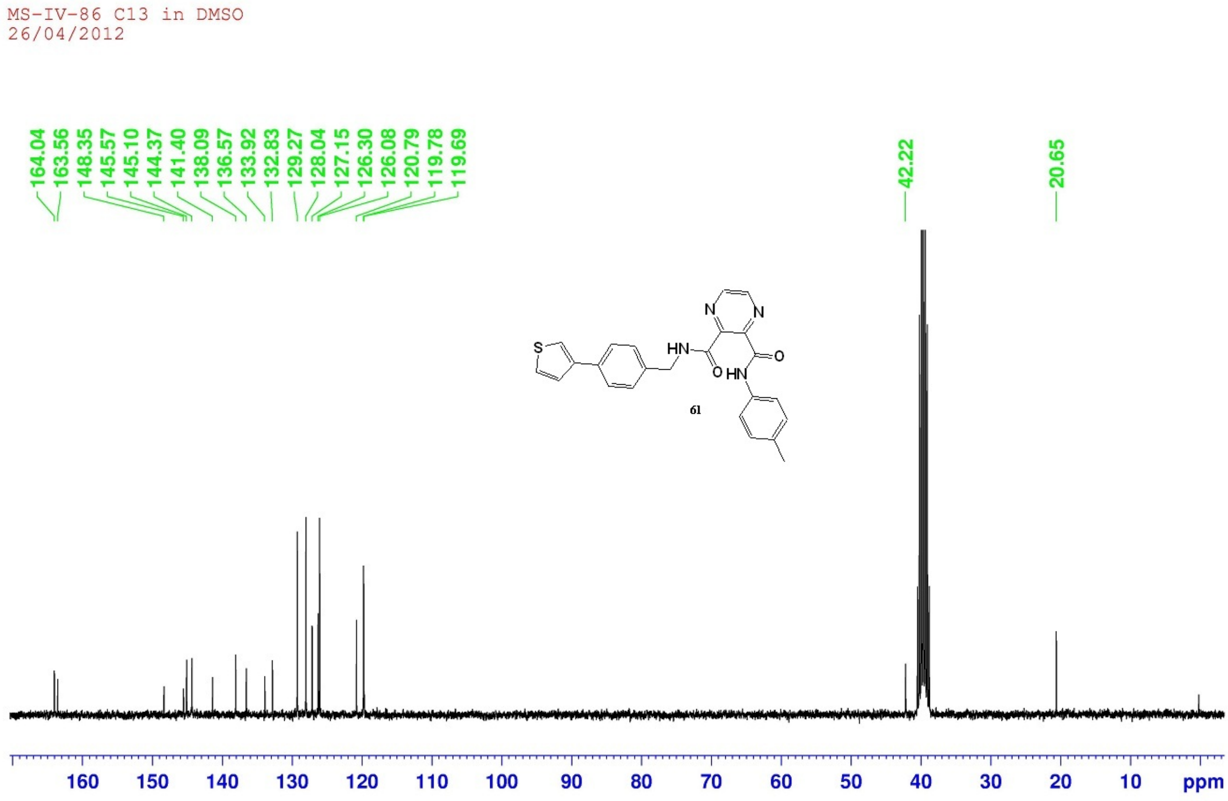

N^2-(4-fluorophenyl)-N^3-(2-(3-methyl-1H-pyrazol-5-yl)phenyl)pyrazine-2,3-dicarboxamide (6m)

HRMS

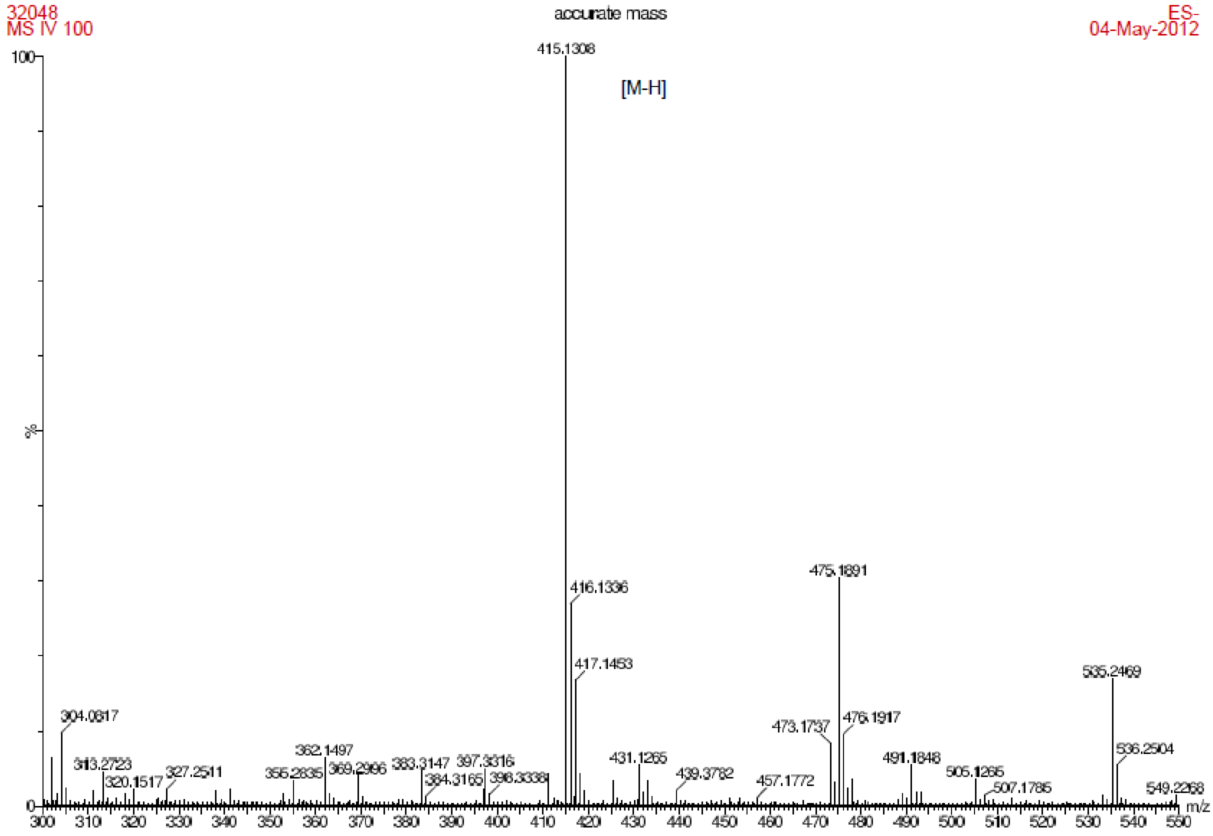

^{1}H NMR (300 MHz, DMSO)

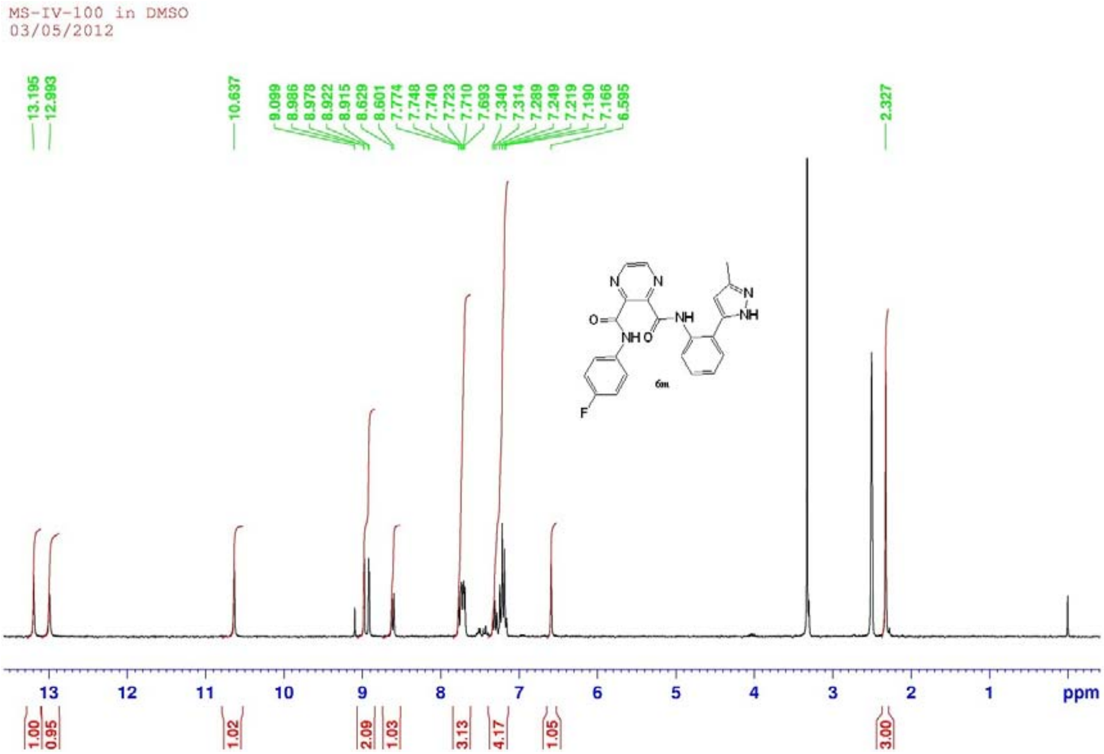

^{13}C NMR (75 MHz, DMSO)

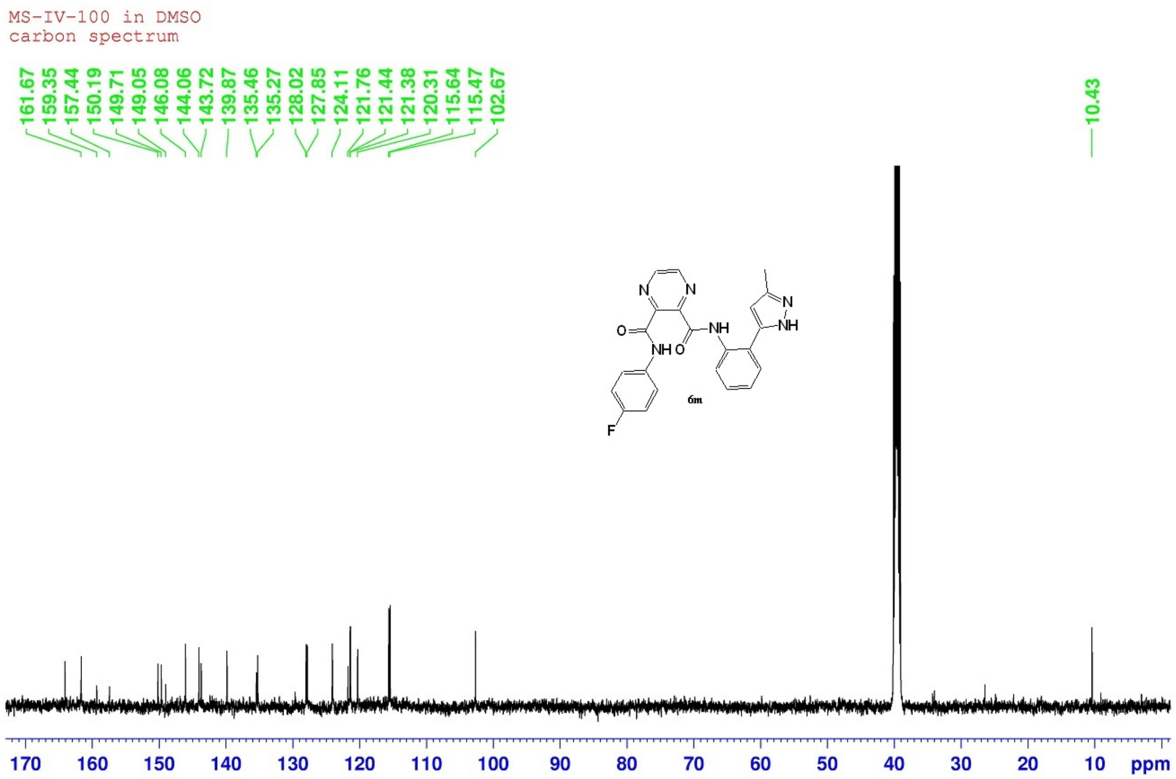

N^2-(2-(3-methyl-1H-pyrazol-5-yl)phenyl)-N^3-(p-tolyl)pyrazine-2,3-dicarboxamide (6n)

HRMS

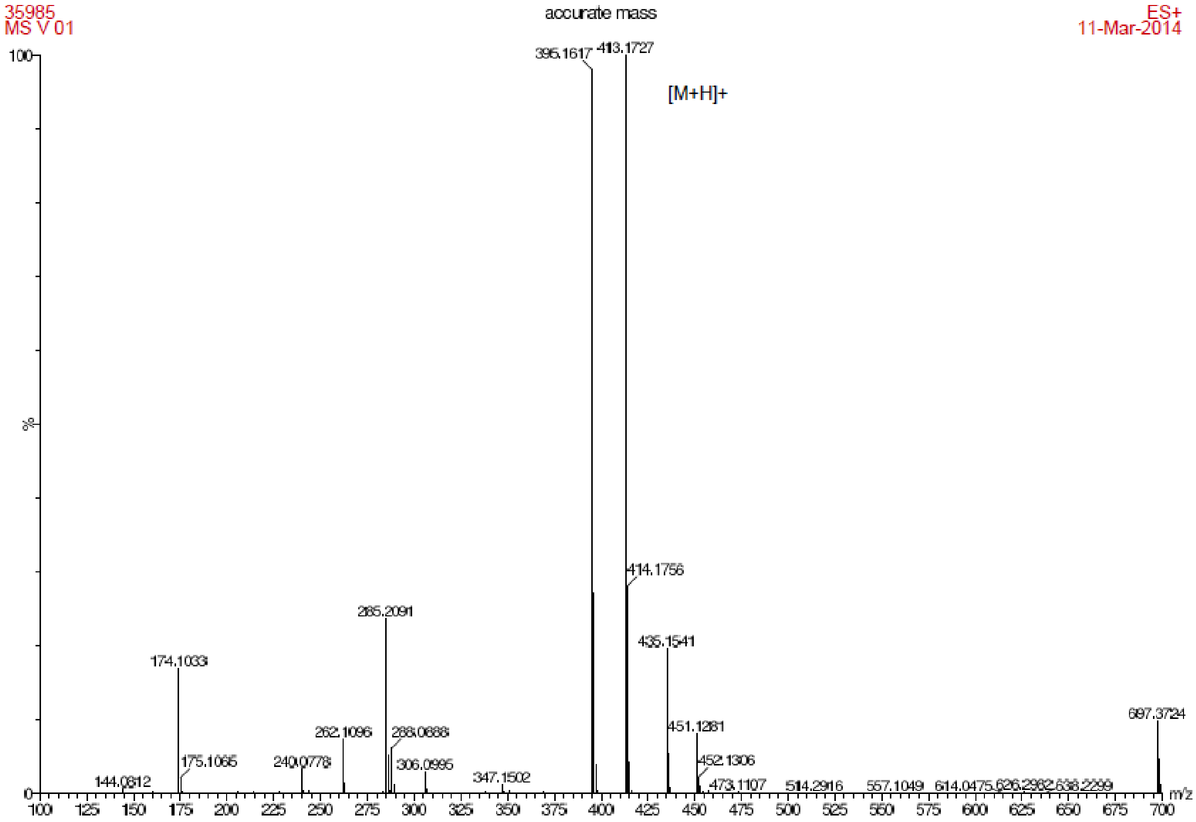

^{1}H NMR (300 MHz, DMSO)

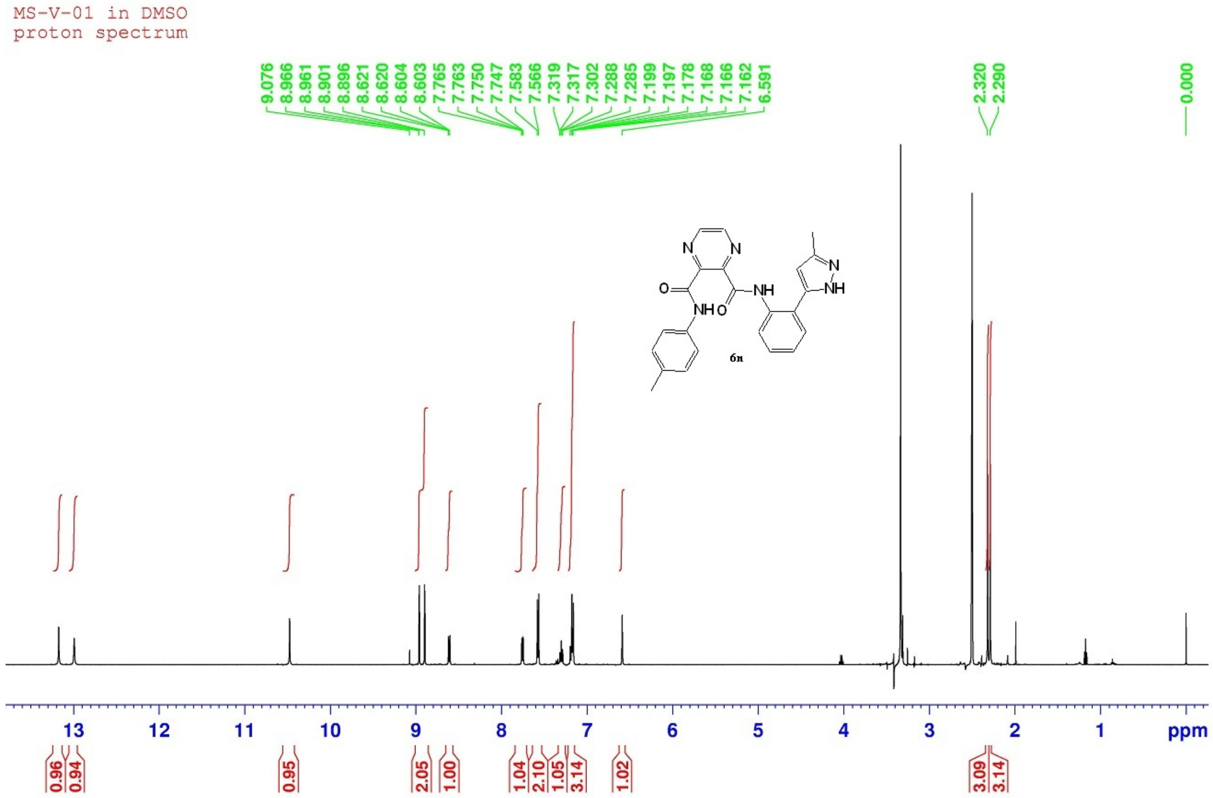

^{13}C NMR (75 MHz, DMSO)

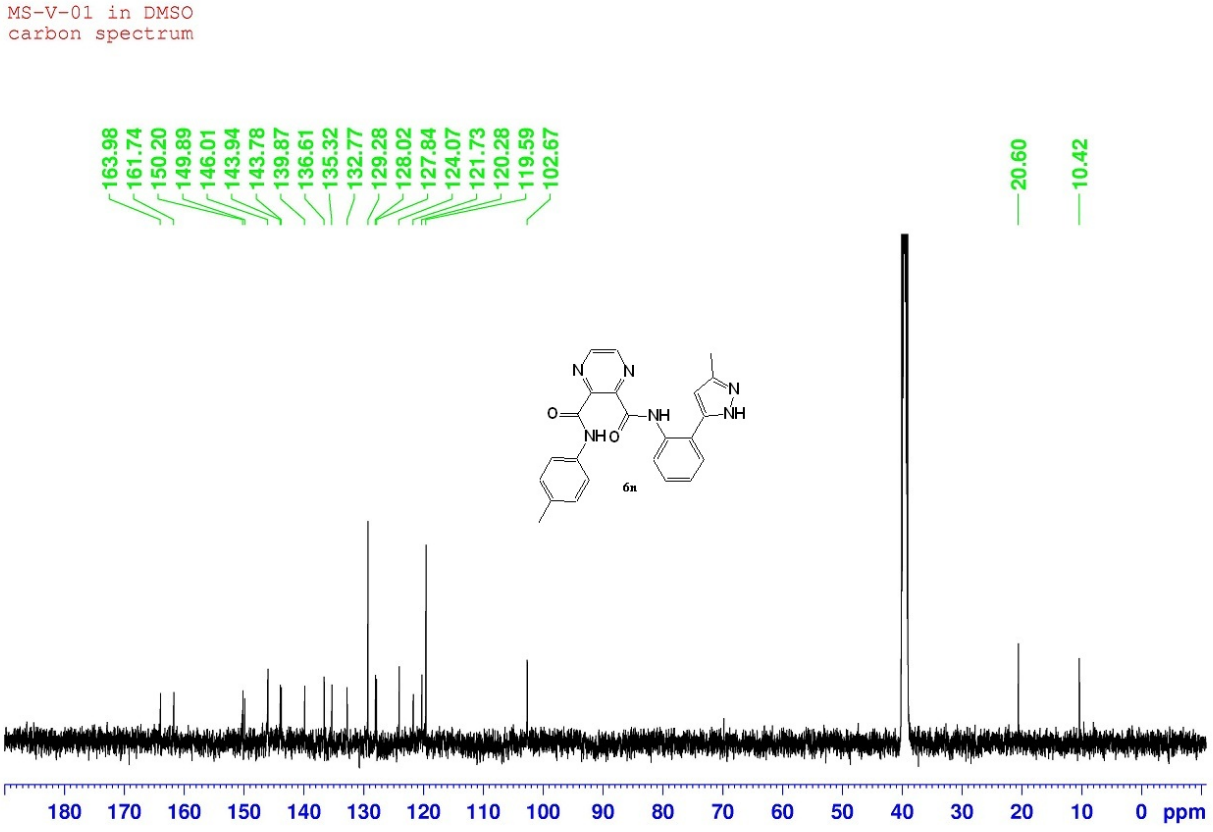

N^1-(4-fluorophenyl)-N^2-(4-methyl-2-(3-methyl-1H-pyrazol-5-yl)phenyl)phthalamide (7a)

HRMS

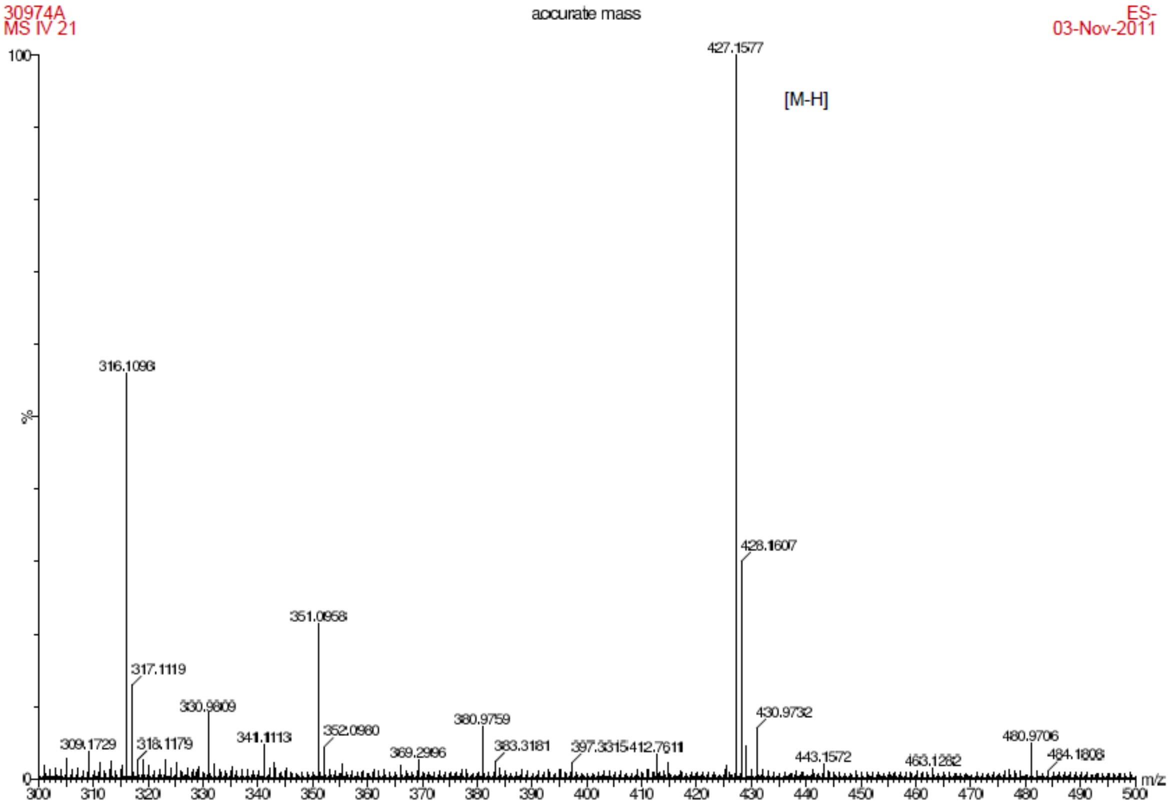

^{1}H NMR (600 MHz, DMSO)

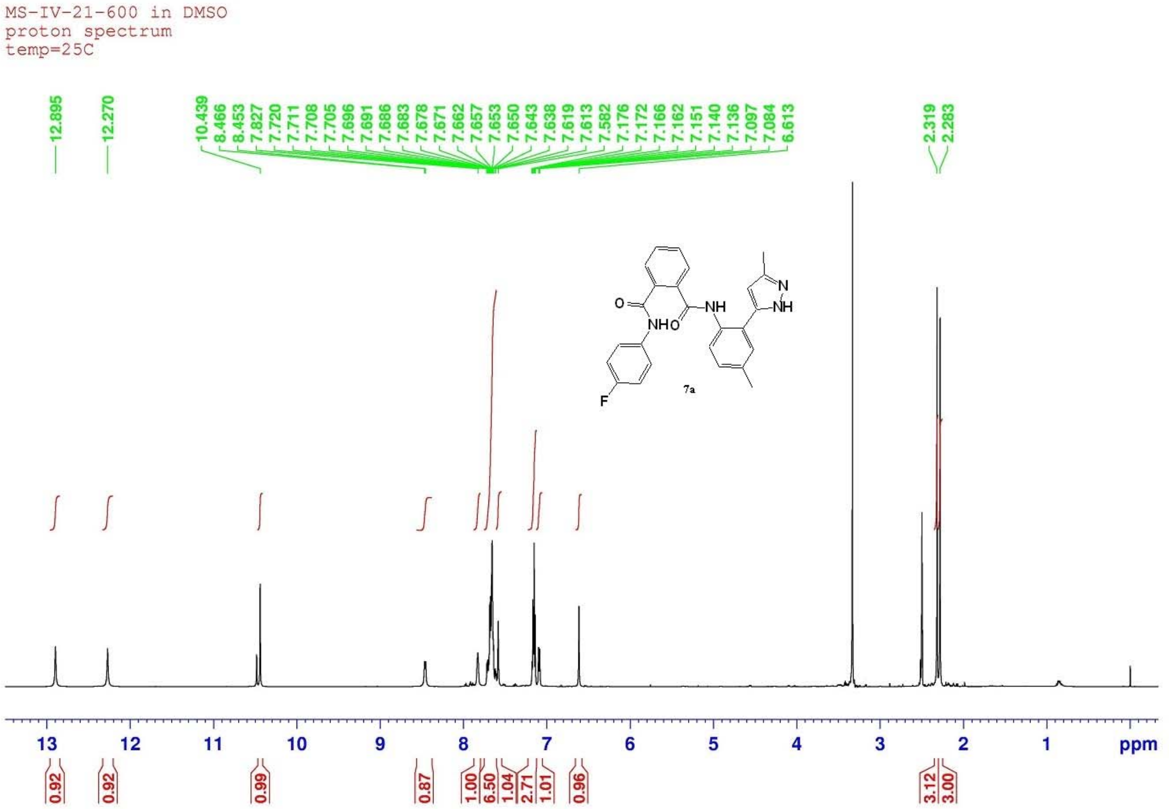

^{13}C NMR (150 MHz, DMSO)

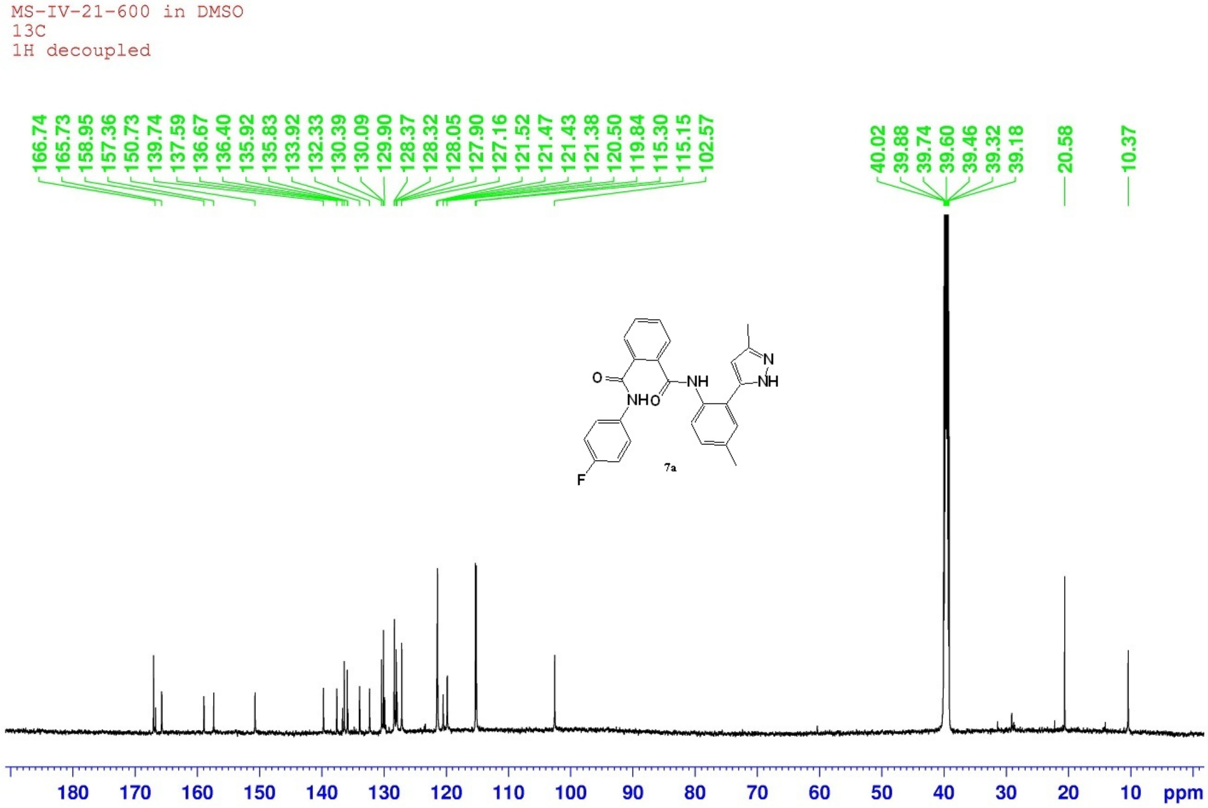

N^1-(4-fluorophenyl)-N^2-(4-methyl-2-(thiophen-2-yl)phenyl)phthalamide (7b)

HRMS

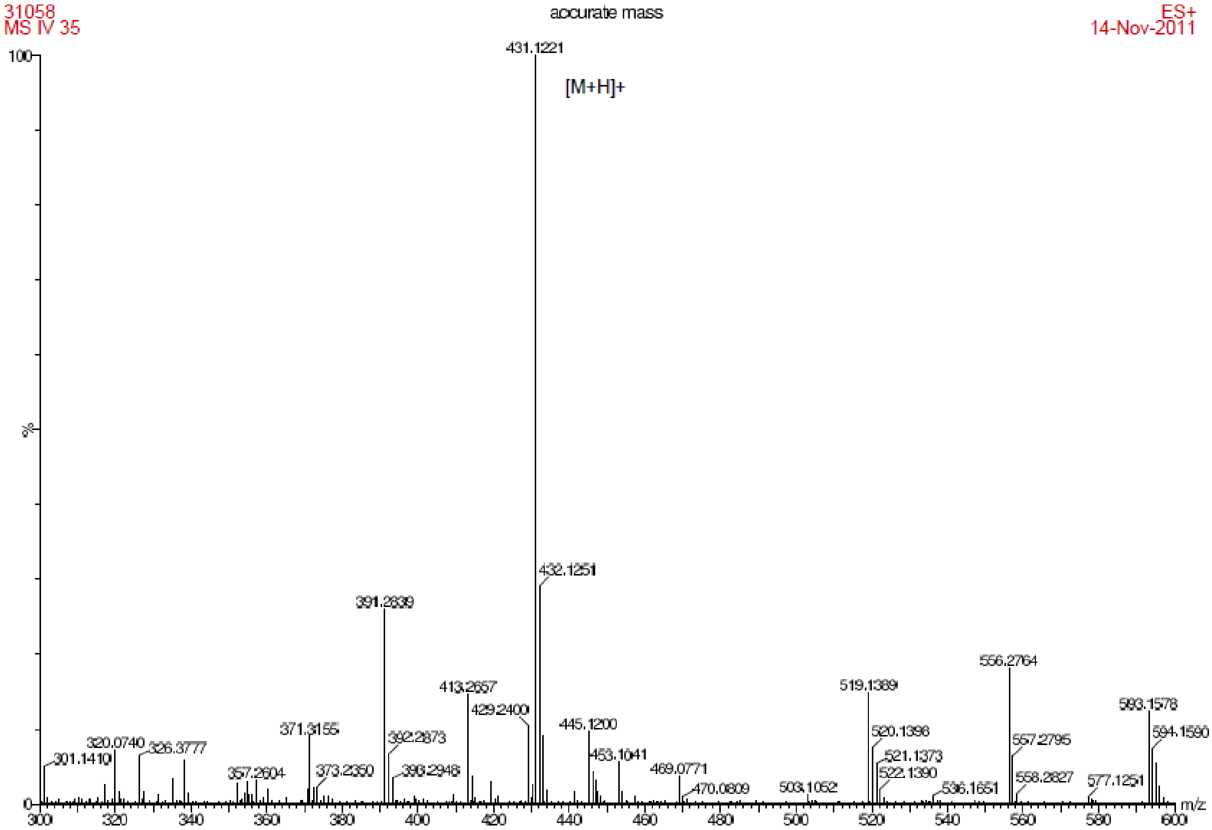

^{1}H NMR (600 MHz, DMSO)

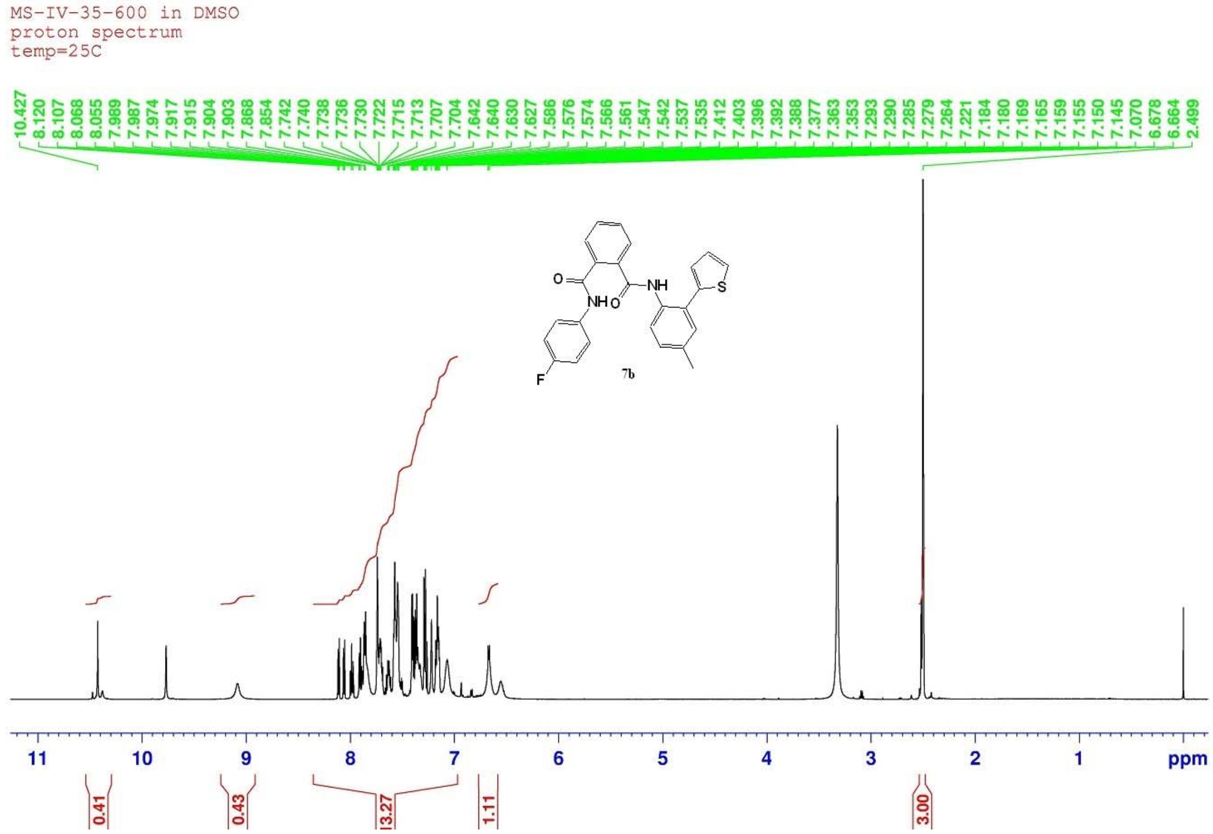

^{13}C NMR (150 MHz, DMSO)

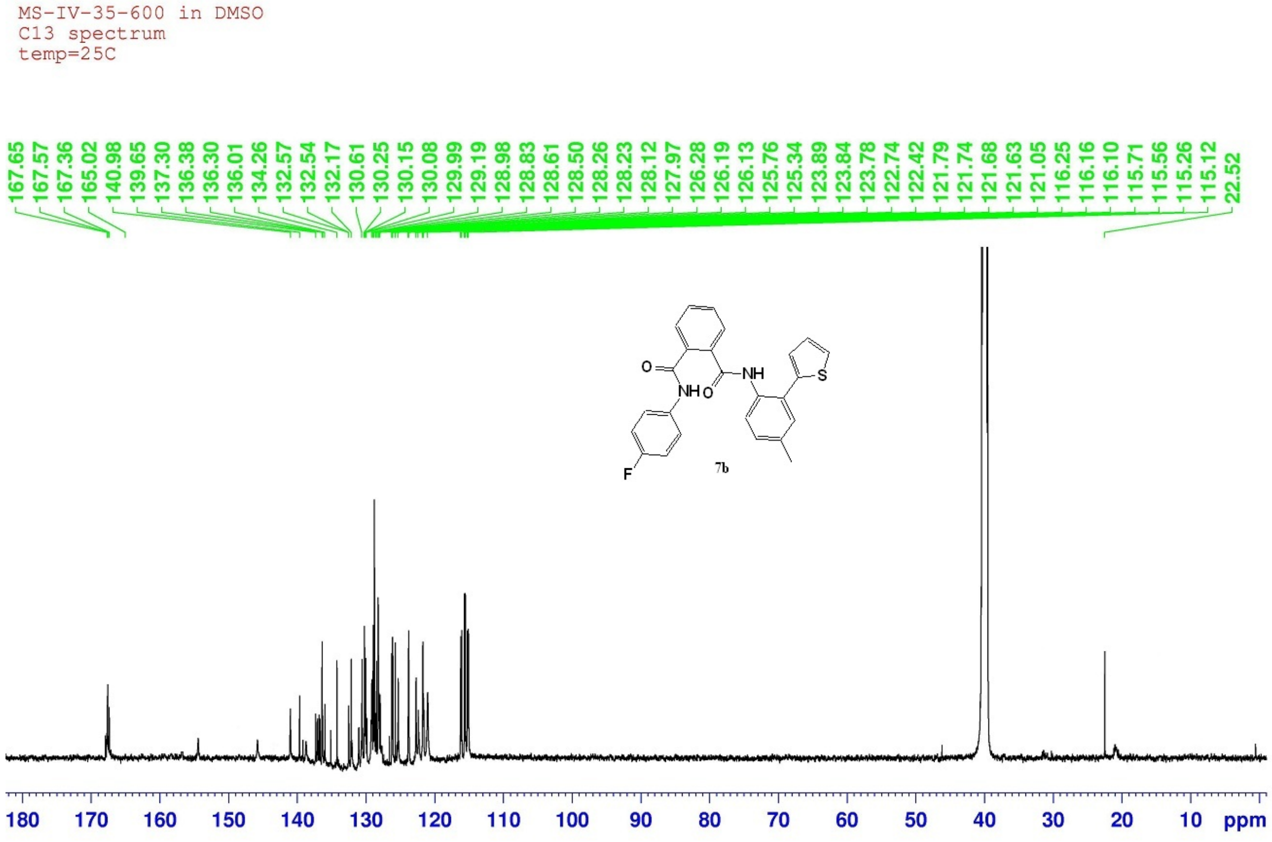

N^1-(4-methyl-2-(3-methyl-1H-pyrazol-5-yl)phenyl)-N^2-(p-tolyl)phthalamide (7c):
HRMS

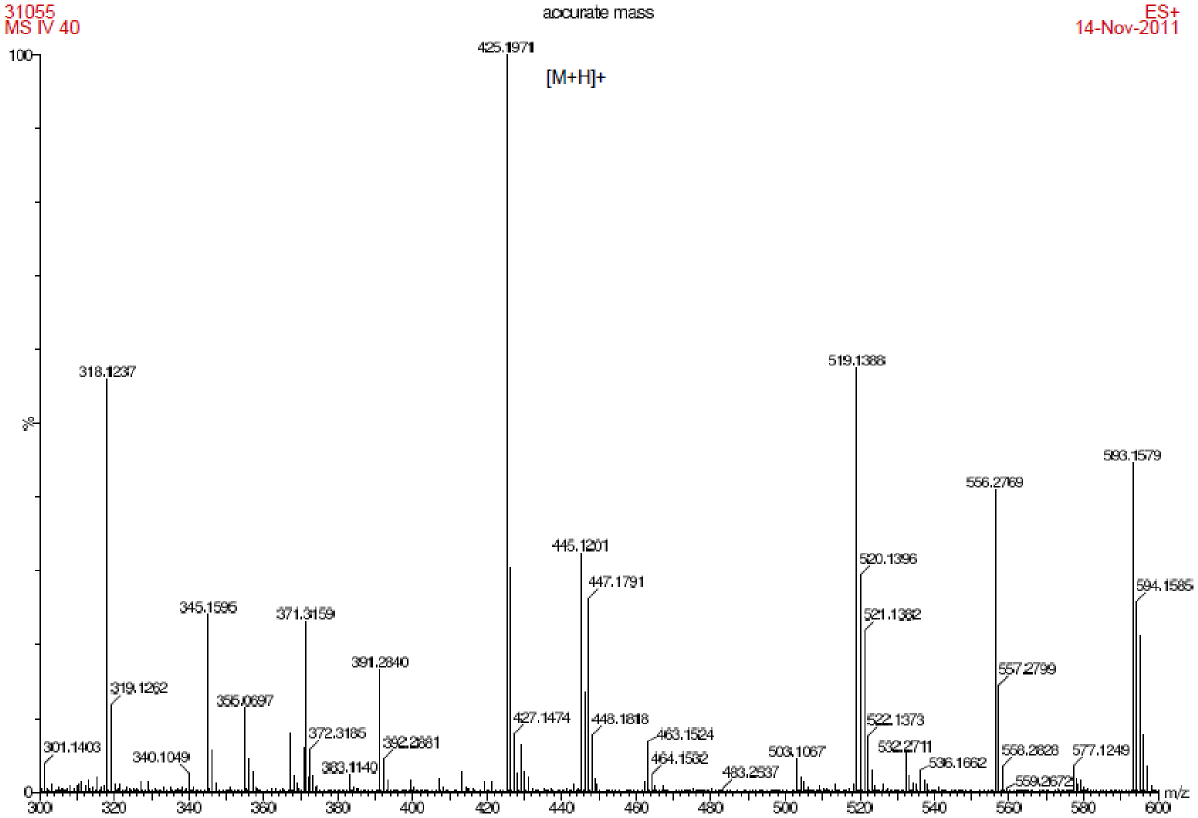

^{1}H NMR (600 MHz, DMSO)

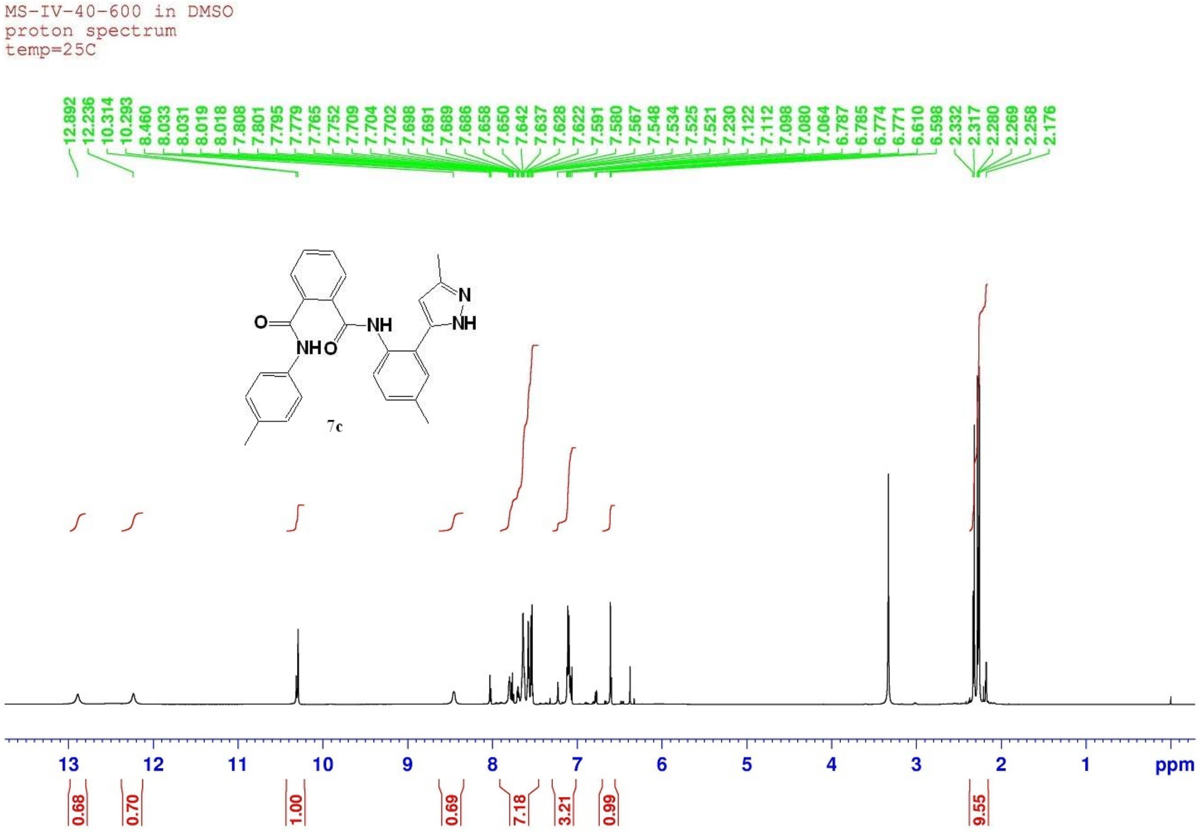

^{13}C NMR (150 MHz, DMSO)

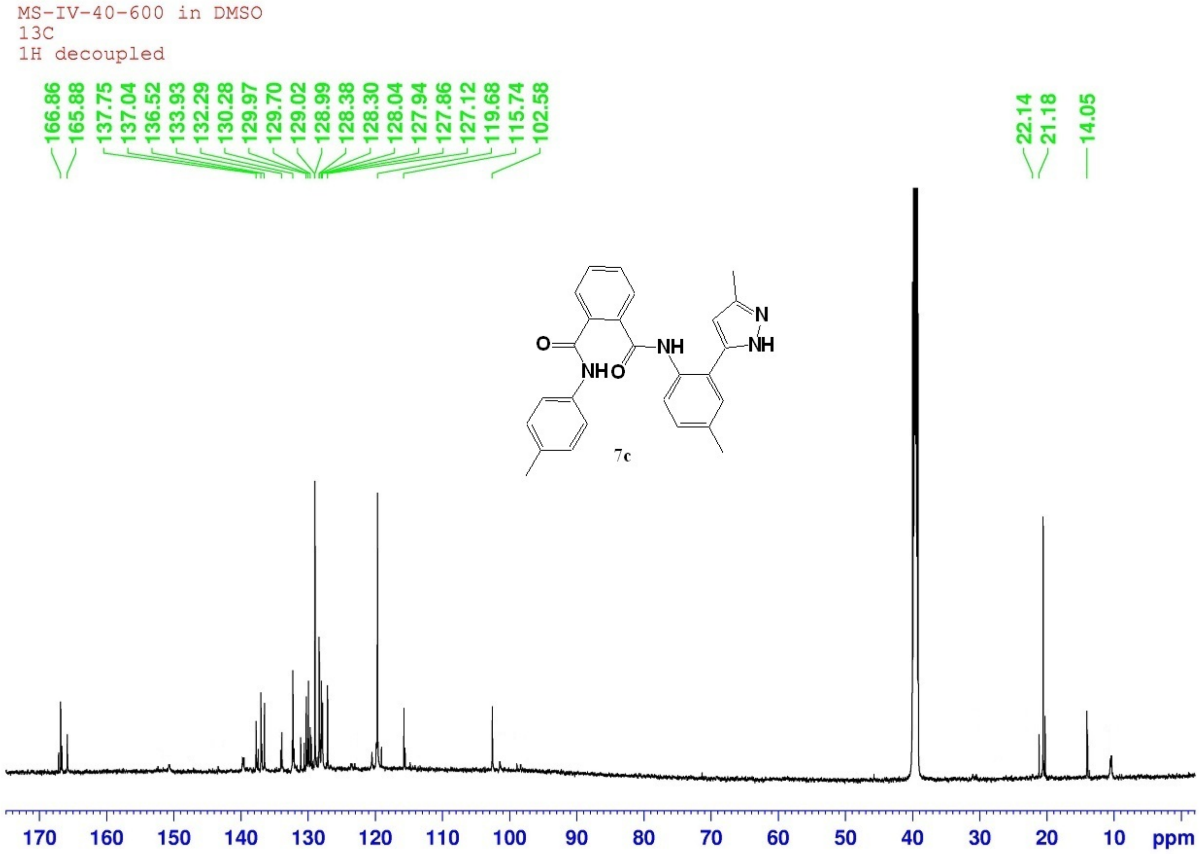

N^1-(4-methyl-2-(thiophen-2-yl)phenyl)-N^2-(p-tolyl)phthalamide (7d)

HRMS

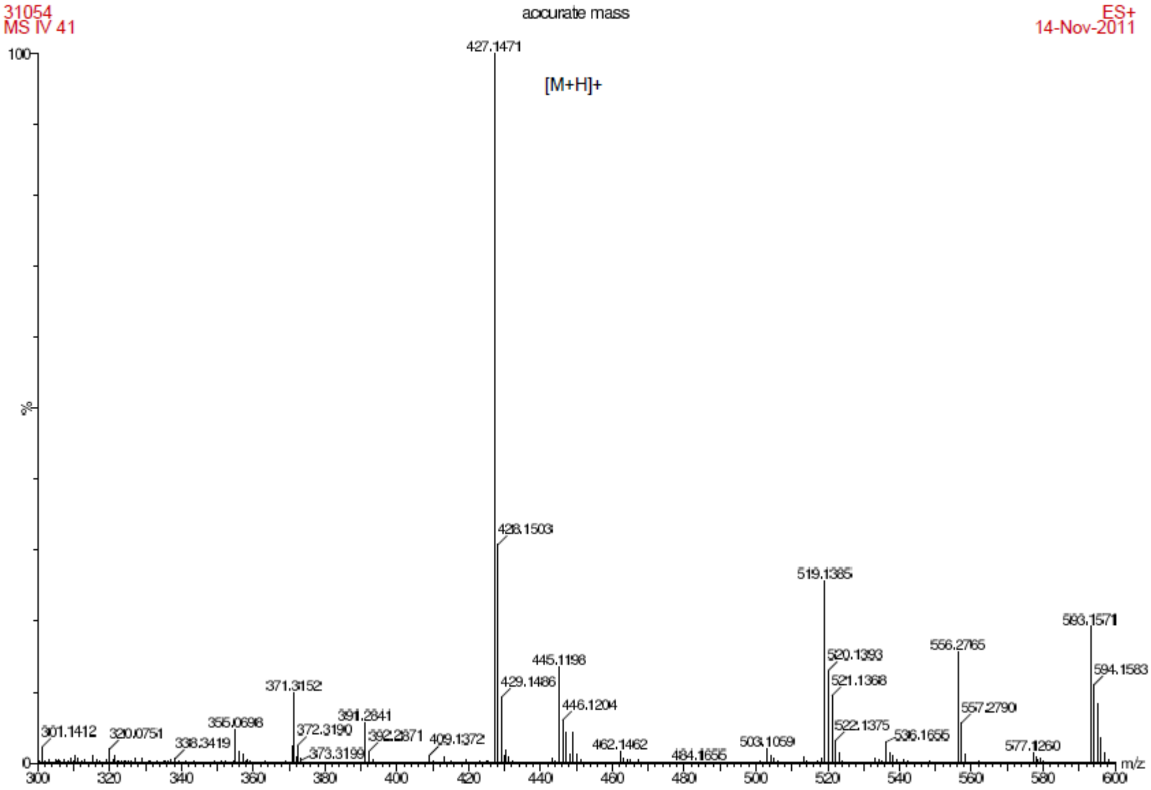

^{1}H NMR (500 MHz, DMSO)

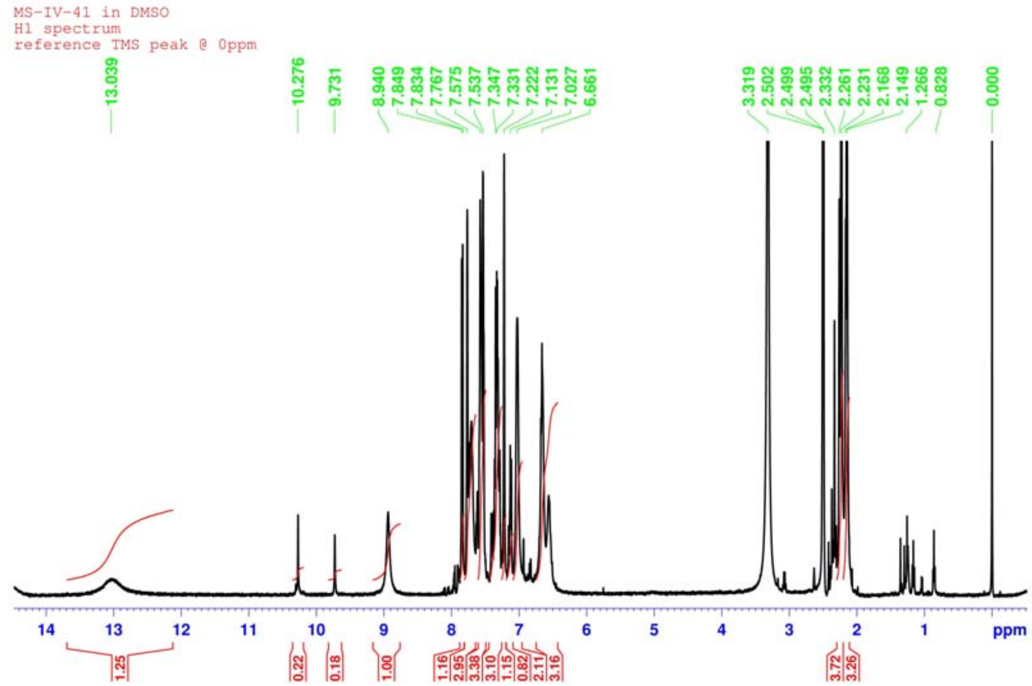

^{13}C NMR (150 MHz, DMSO)

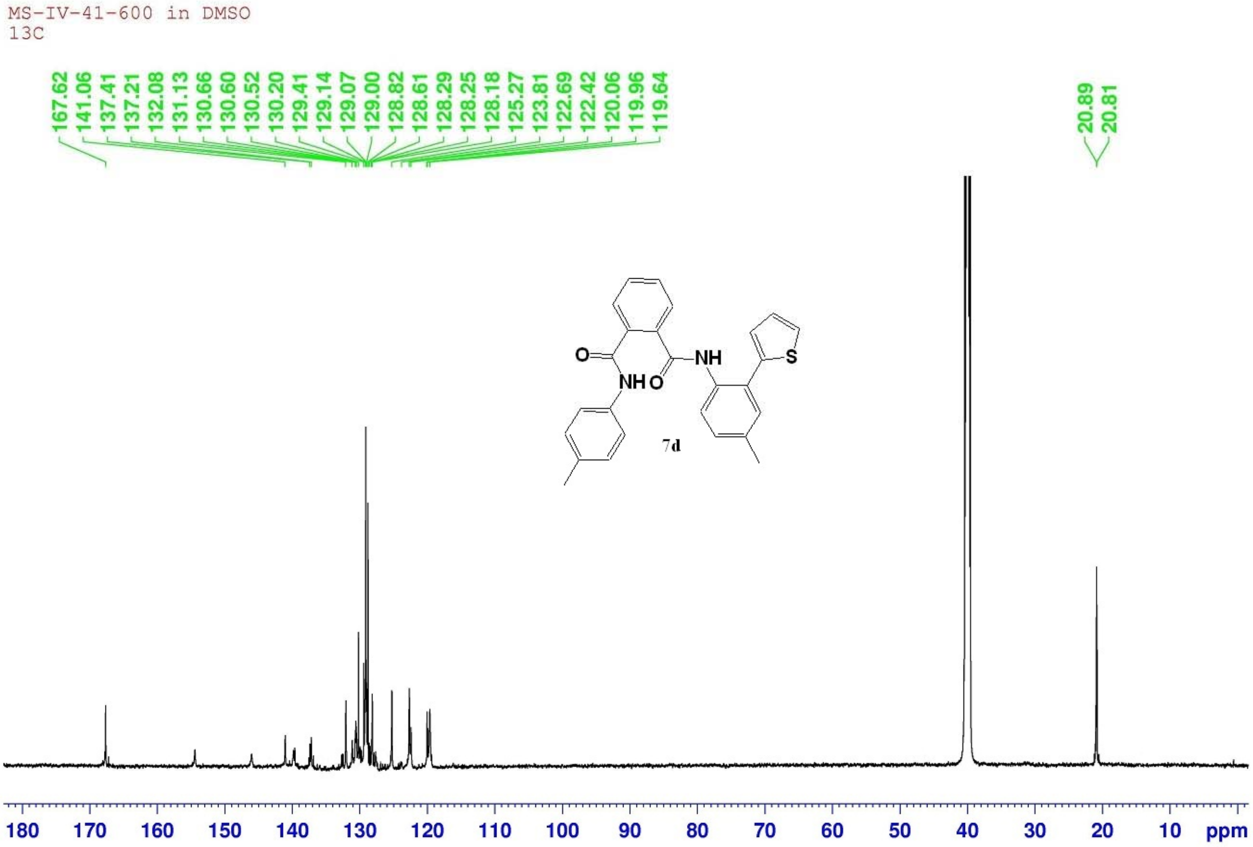

N^1-(4-methyl-2-(5-methylthiophen-2-yl)phenyl)-N^2-(p-tolyl)phthalamide (7e)

HRMS

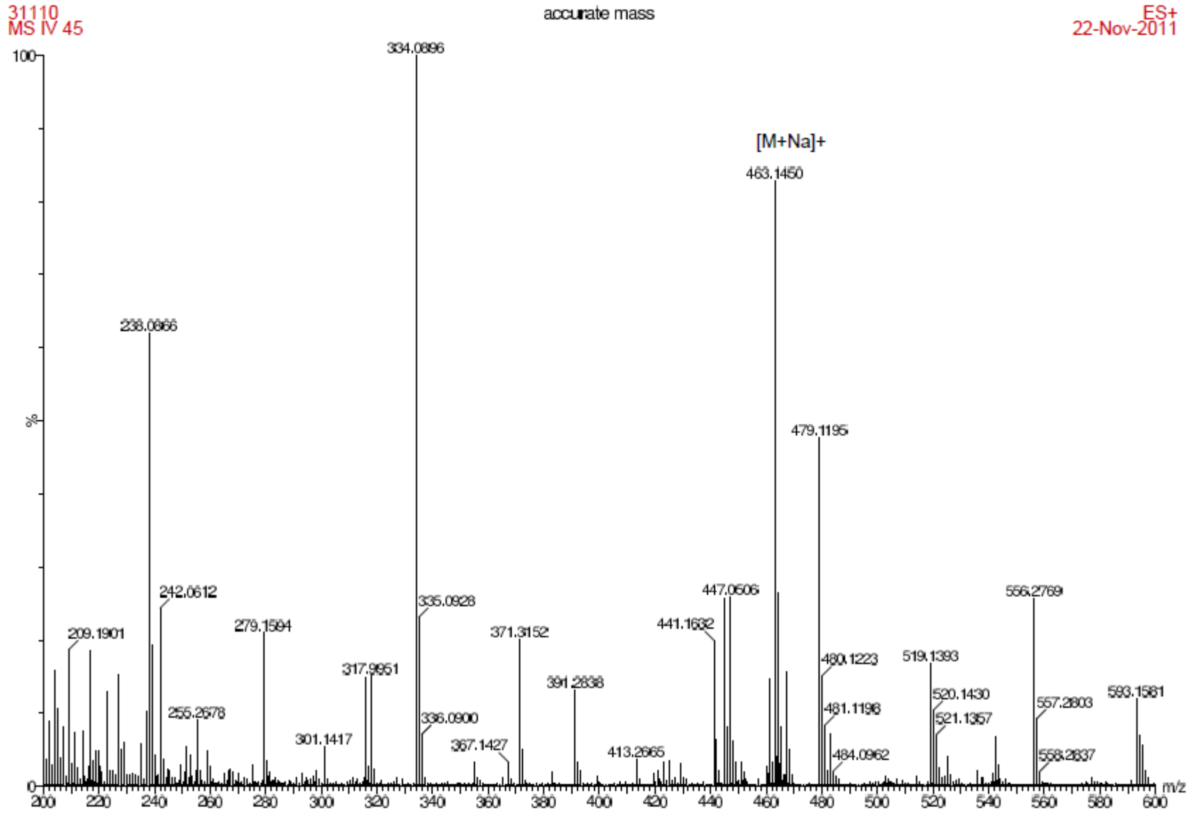

^{1}H NMR (500 MHz, DMSO)

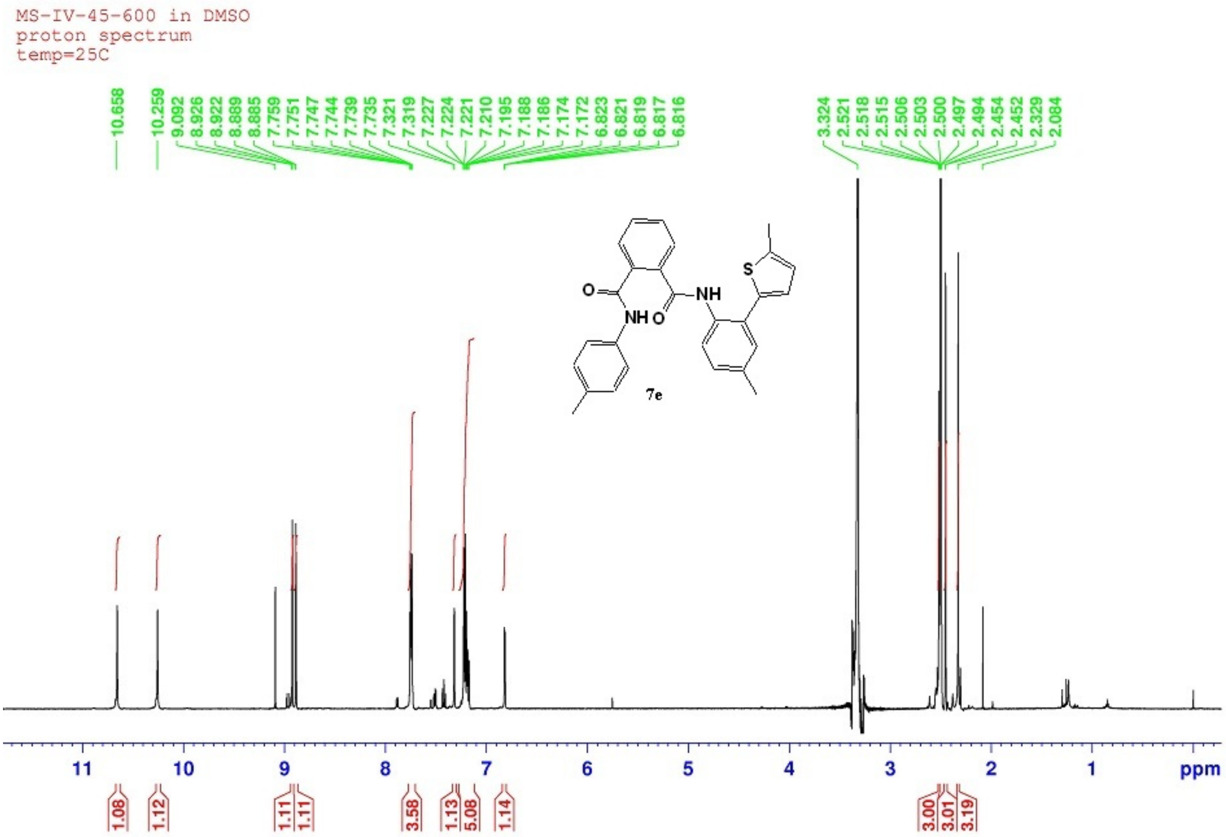

^{13}C NMR (125 MHz, DMSO)

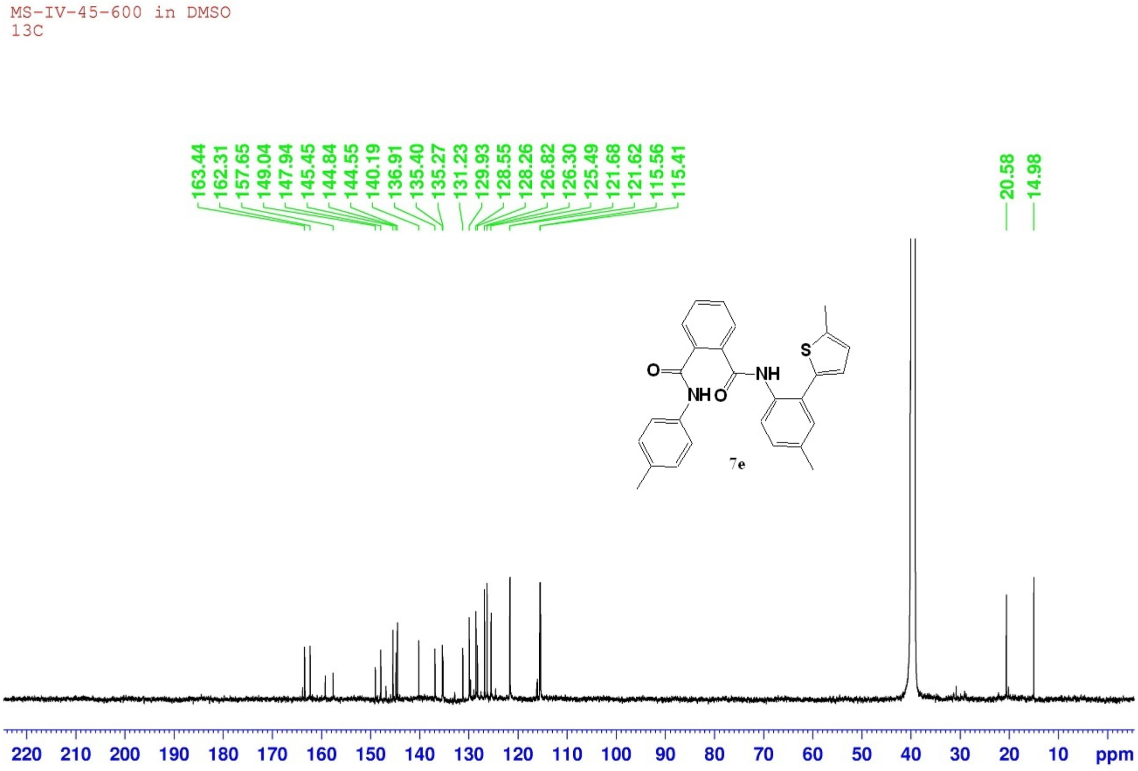

N^1-(4-fluorophenyl)-N^2-(4-methyl-2-(5-methylthiophen-2-yl)phenyl)phthalamide (7f)

HRMS

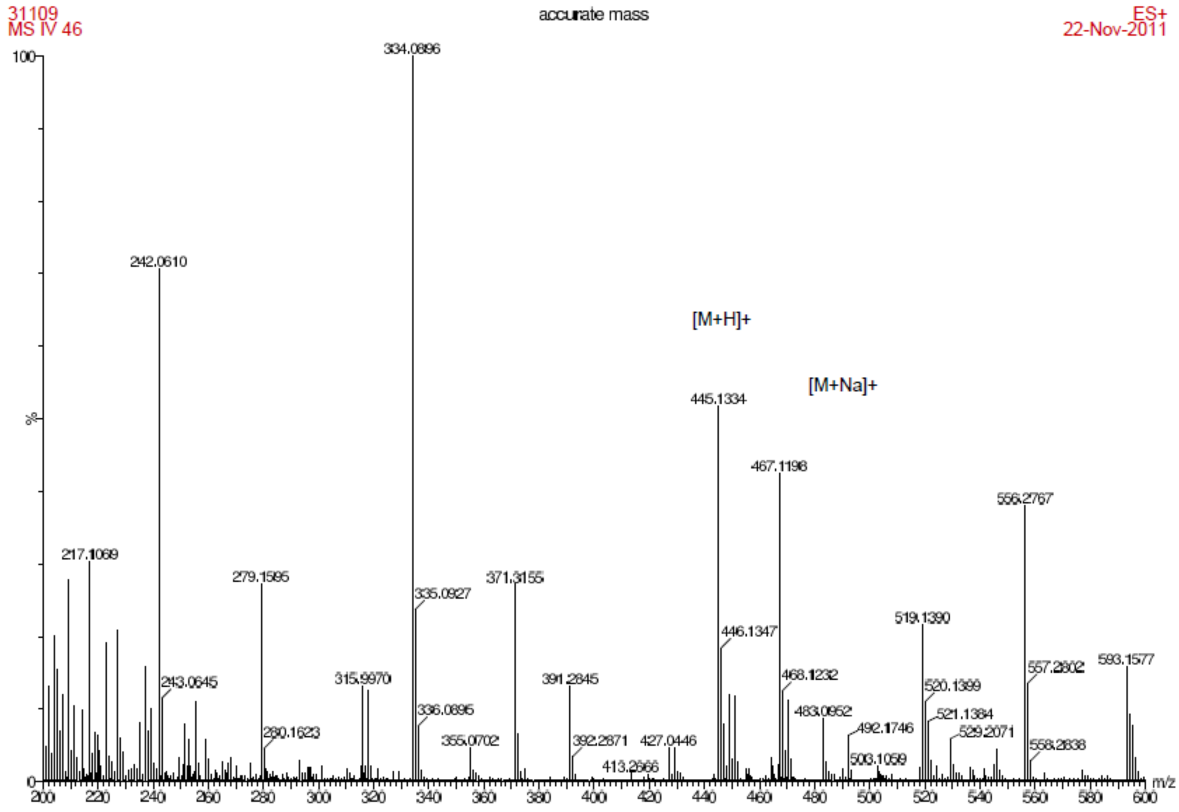

^{1}H NMR (500 MHz, DMSO)

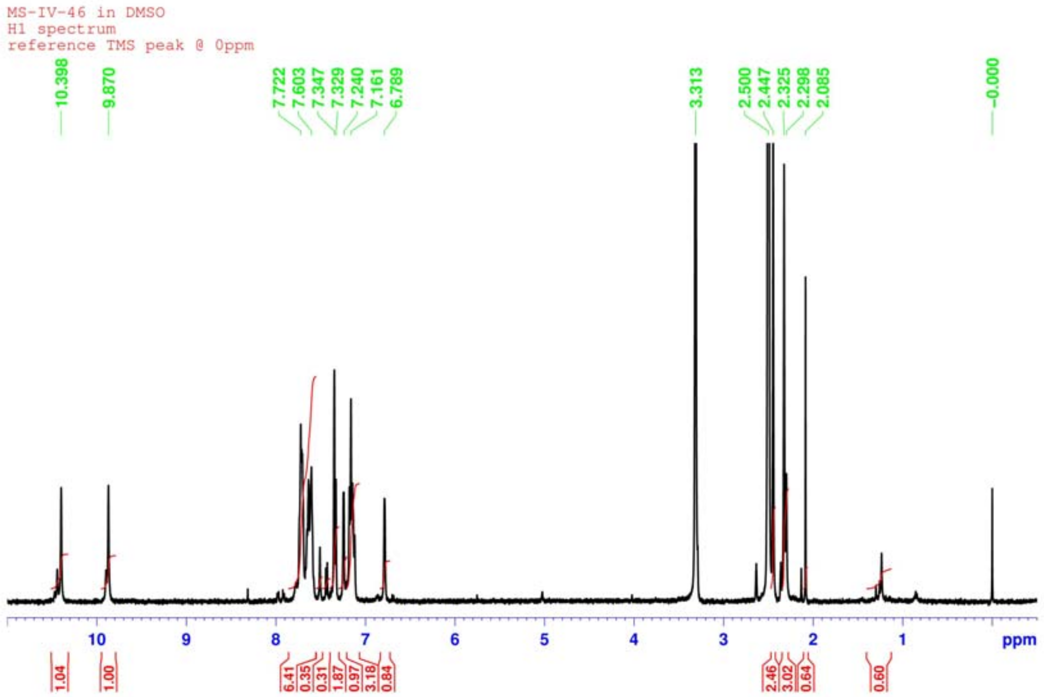

^{13}C NMR (150 MHz, DMSO)

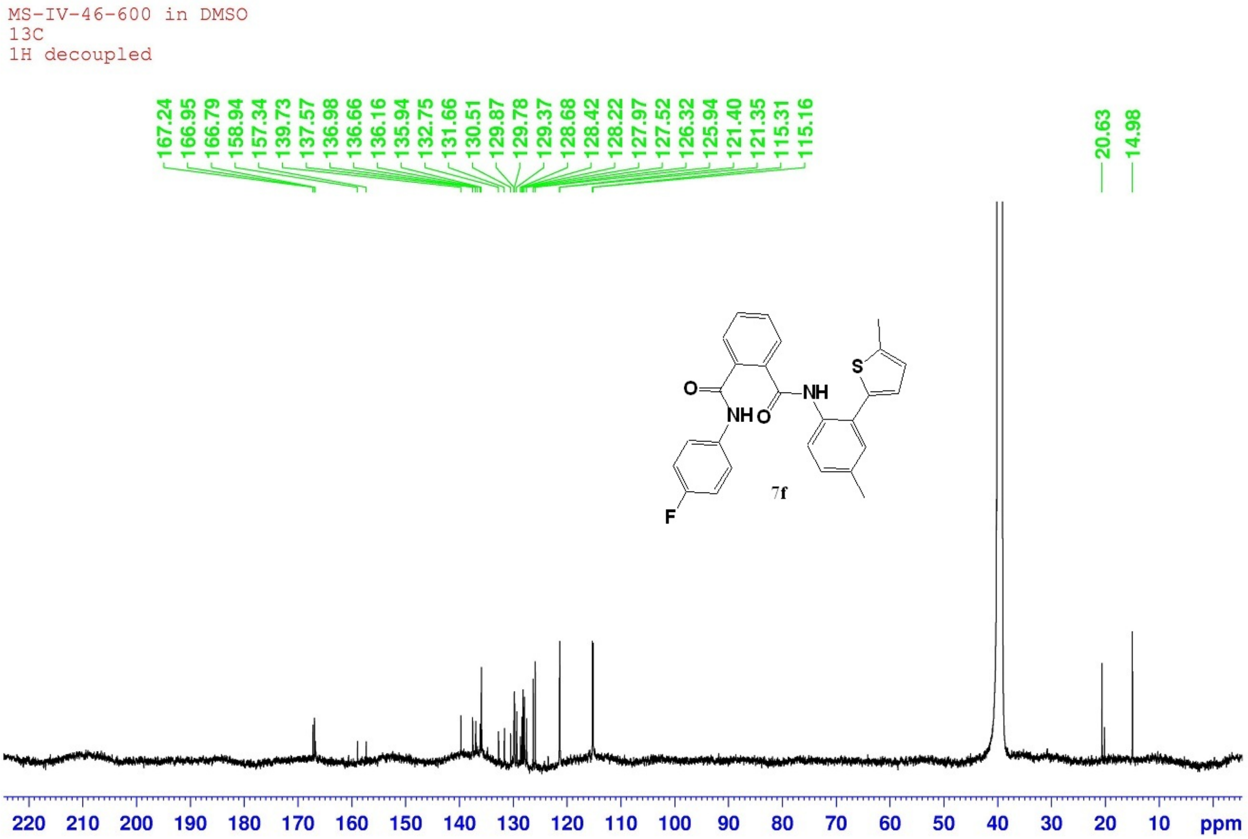

N^1-(4-methyl-2-(thiophen-3-yl)phenyl)-N^2-(p-tolyl)phthalamide (7g)

HRMS

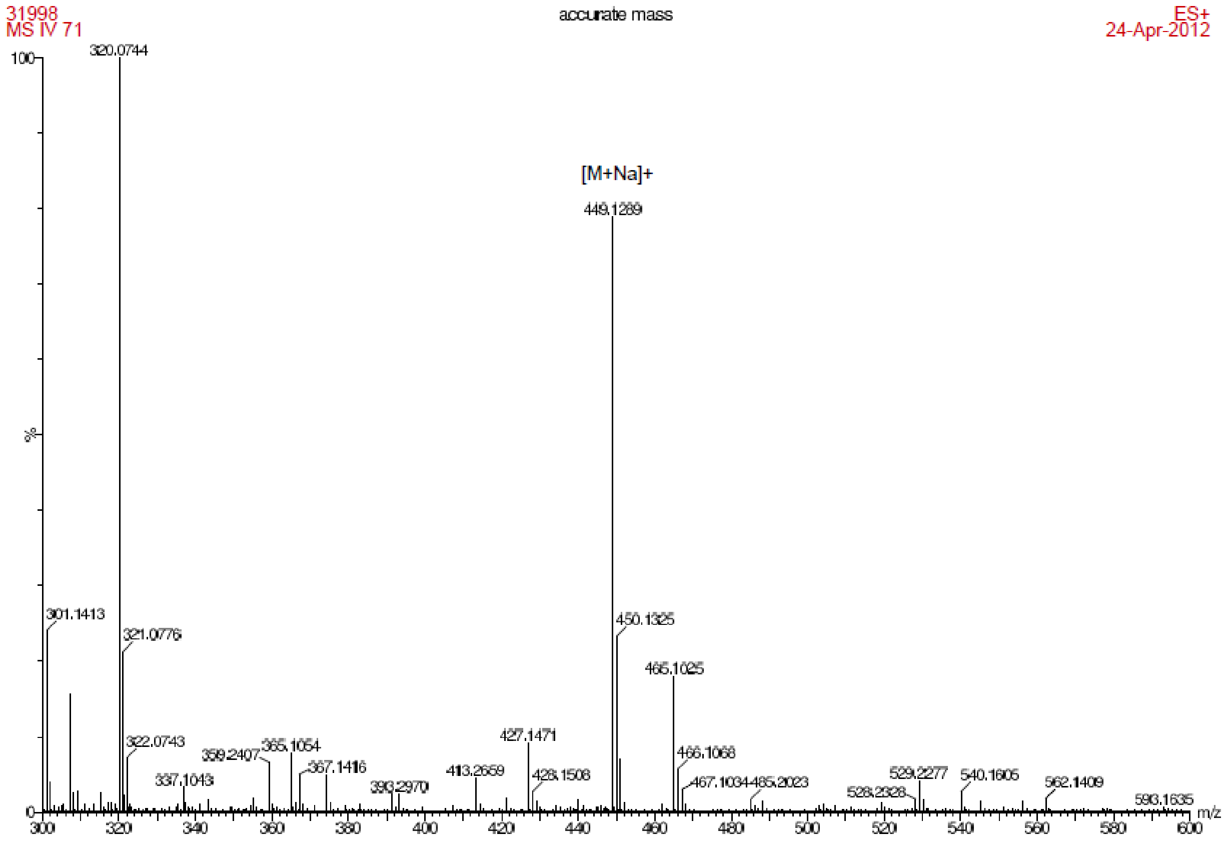

^{1}H NMR (500 MHz, $CDCl_3$)

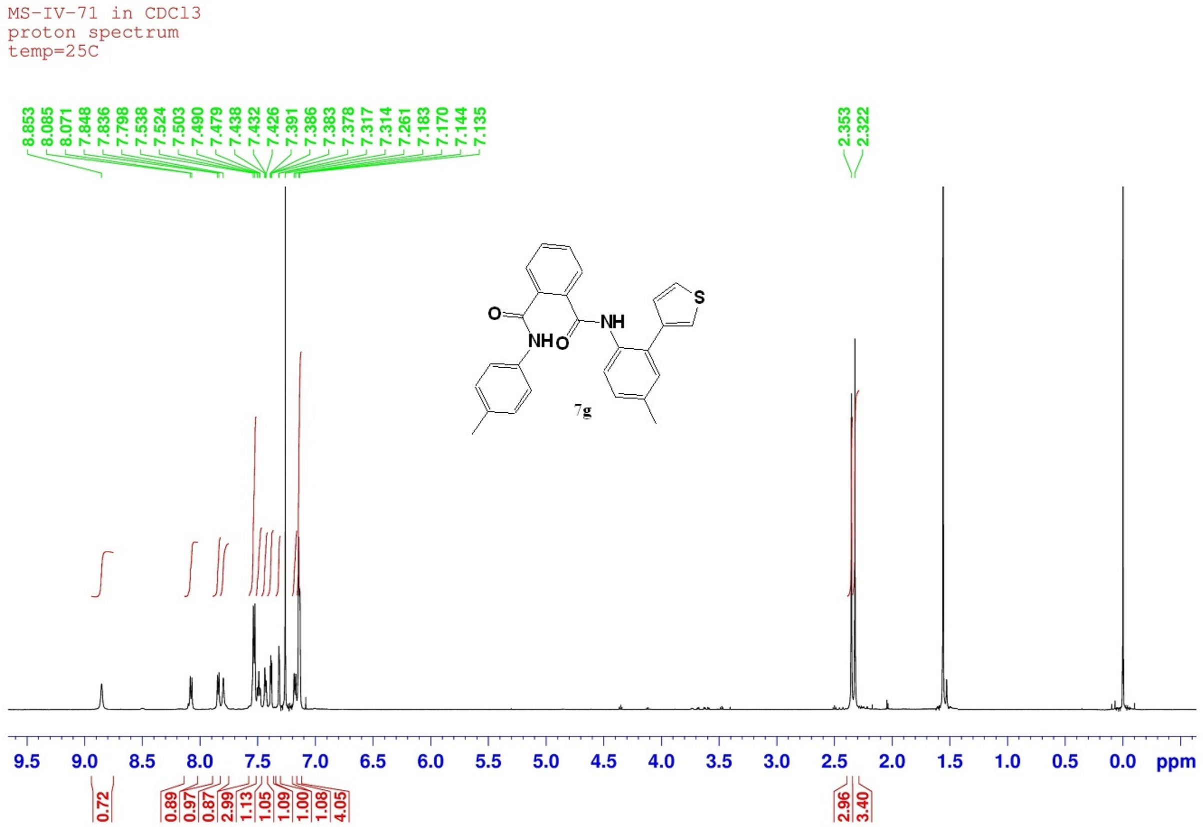

^{13}C NMR (125 MHz, $CDCl_3$)

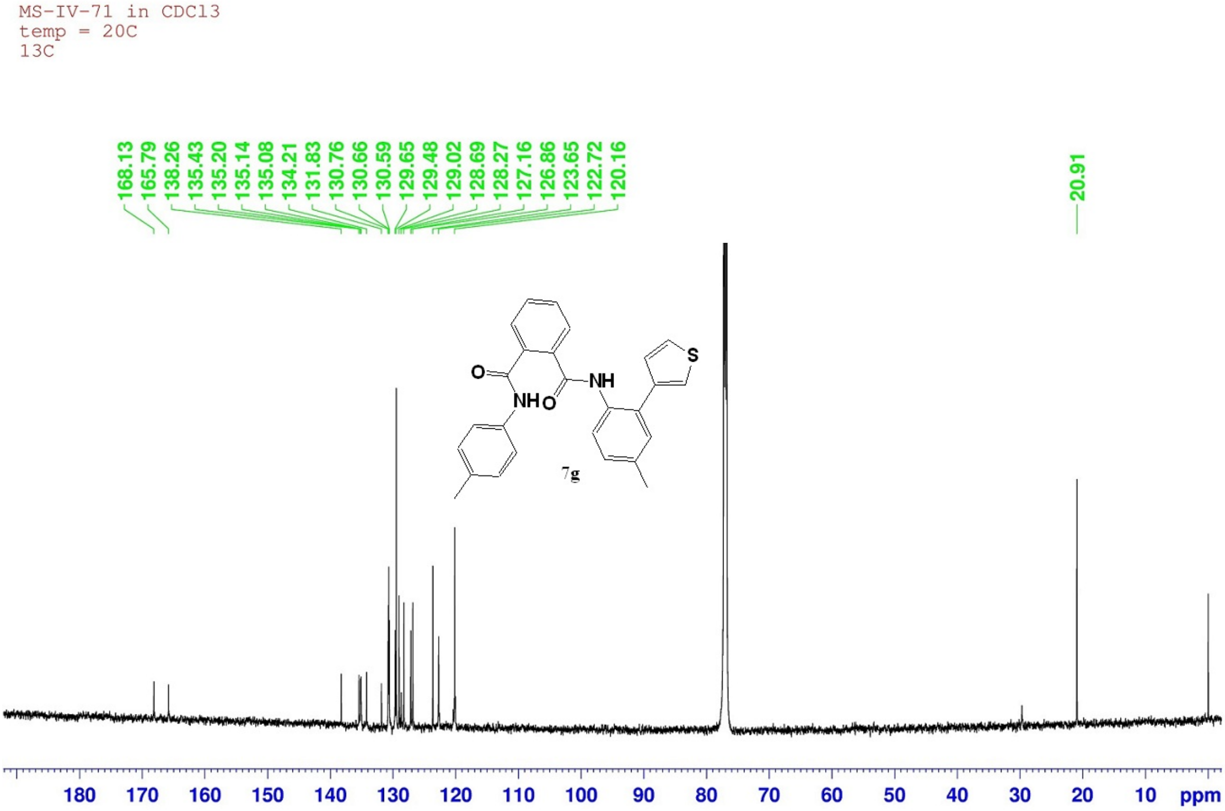

N^1-(4-fluorophenyl)-N^2-(4-methyl-2-(thiophen-3-yl)phenyl)phthalamide (7h)

HRMS

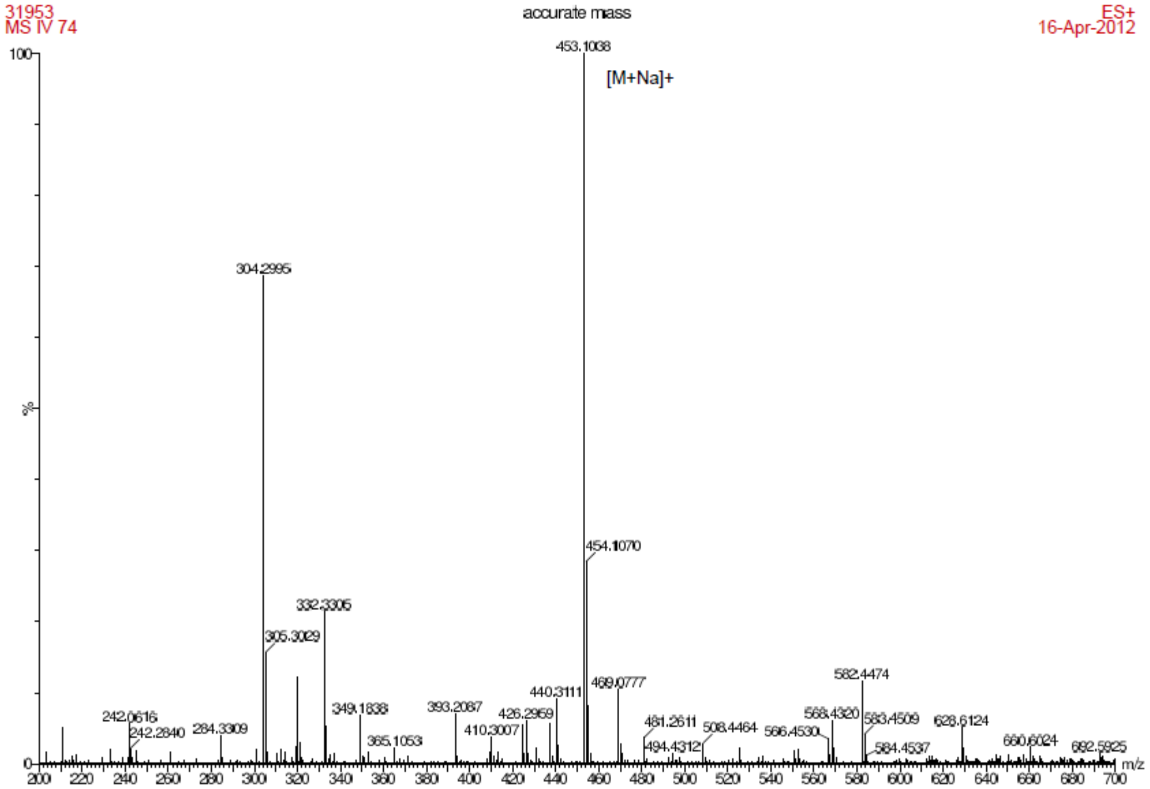

^{1}H NMR (500 MHz, $CDCl_3$)

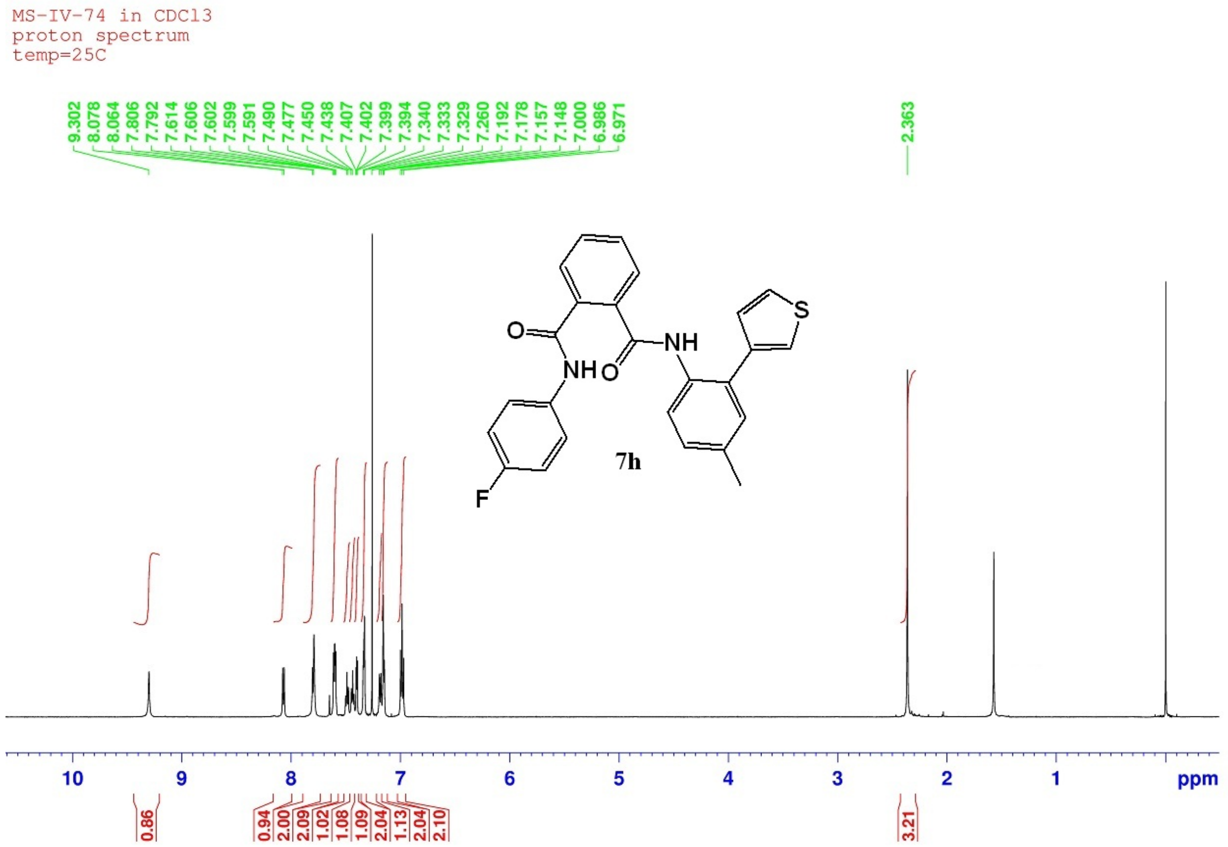

^{13}C NMR (125 MHz, $CDCl_3$)

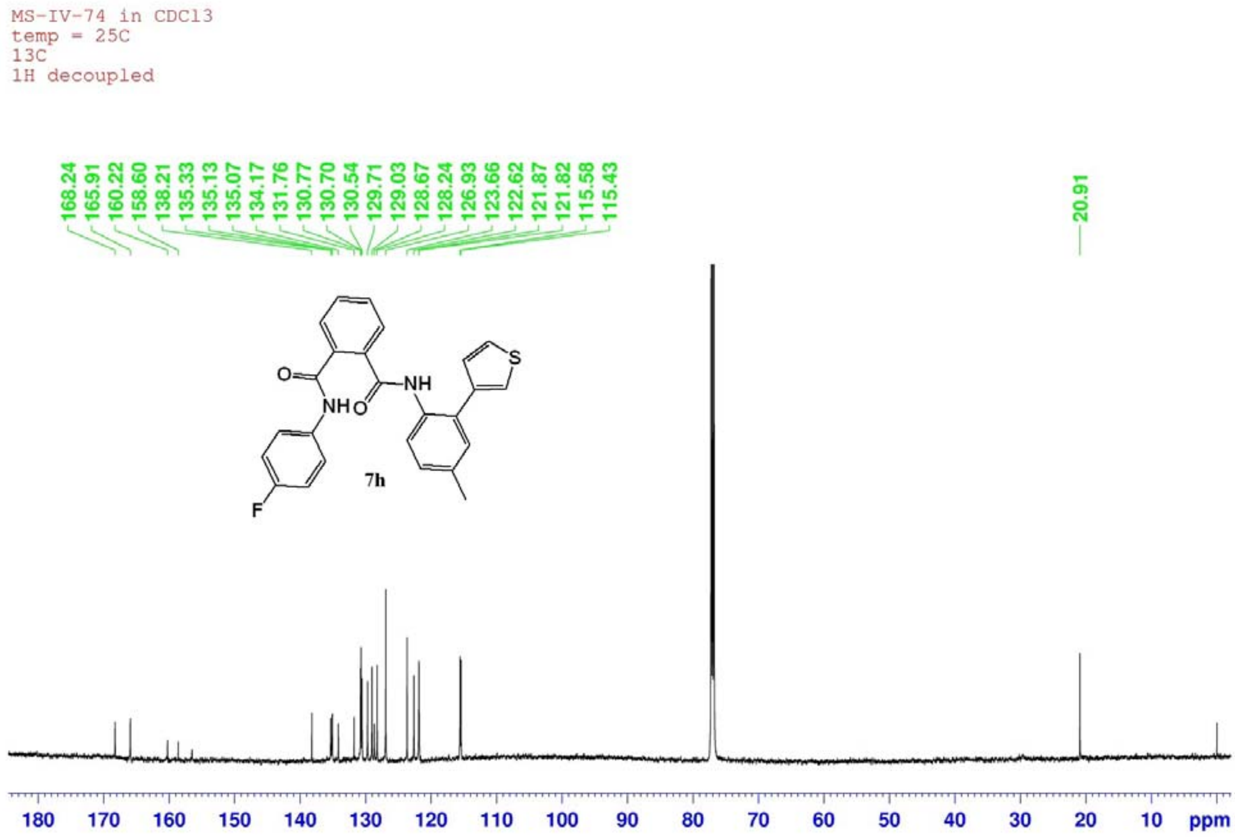

N^1-(4-fluorophenyl)-N^2-(2-(thiophen-3-yl)benzyl)phthalamide (7i)

HRMS

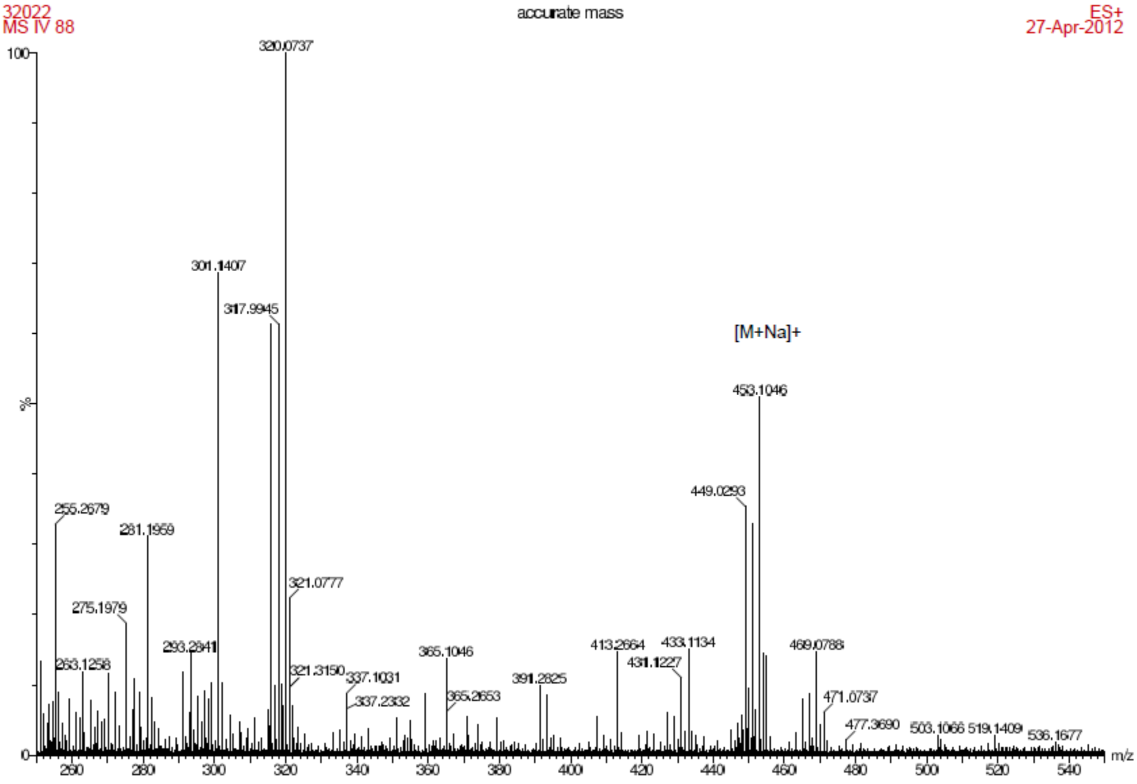

^{1}H NMR (500 MHz, DMSO)

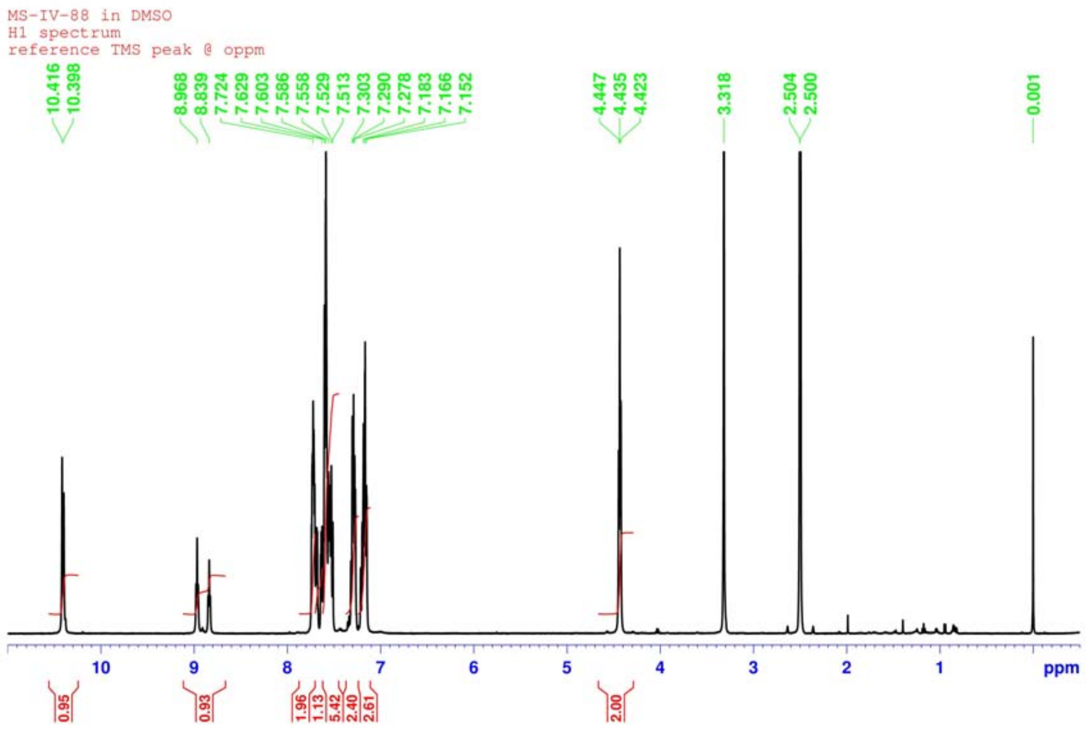

^{13}C NMR (75 MHz, DMSO)

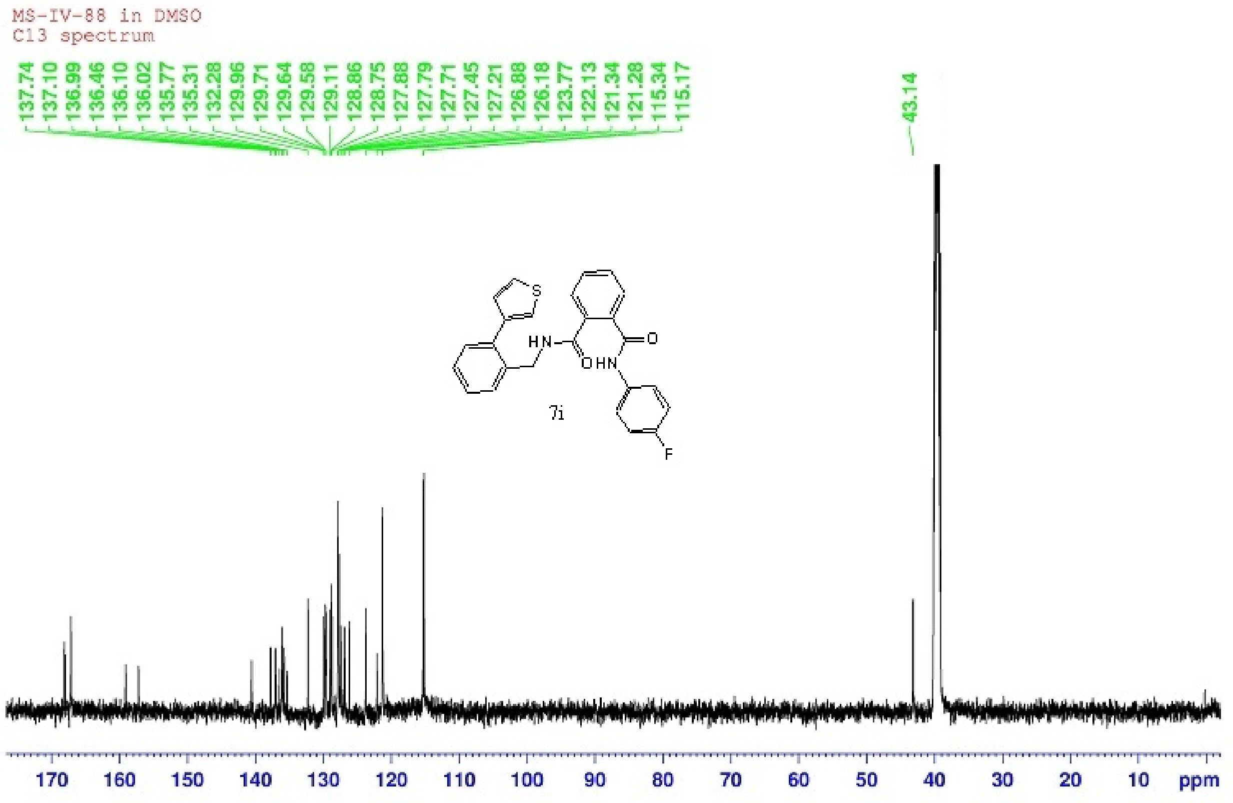

N[1]-(2-(thiophen-3-yl)benzyl)-*N*[2]-(p-tolyl)phthalamide (7j)

HRMS

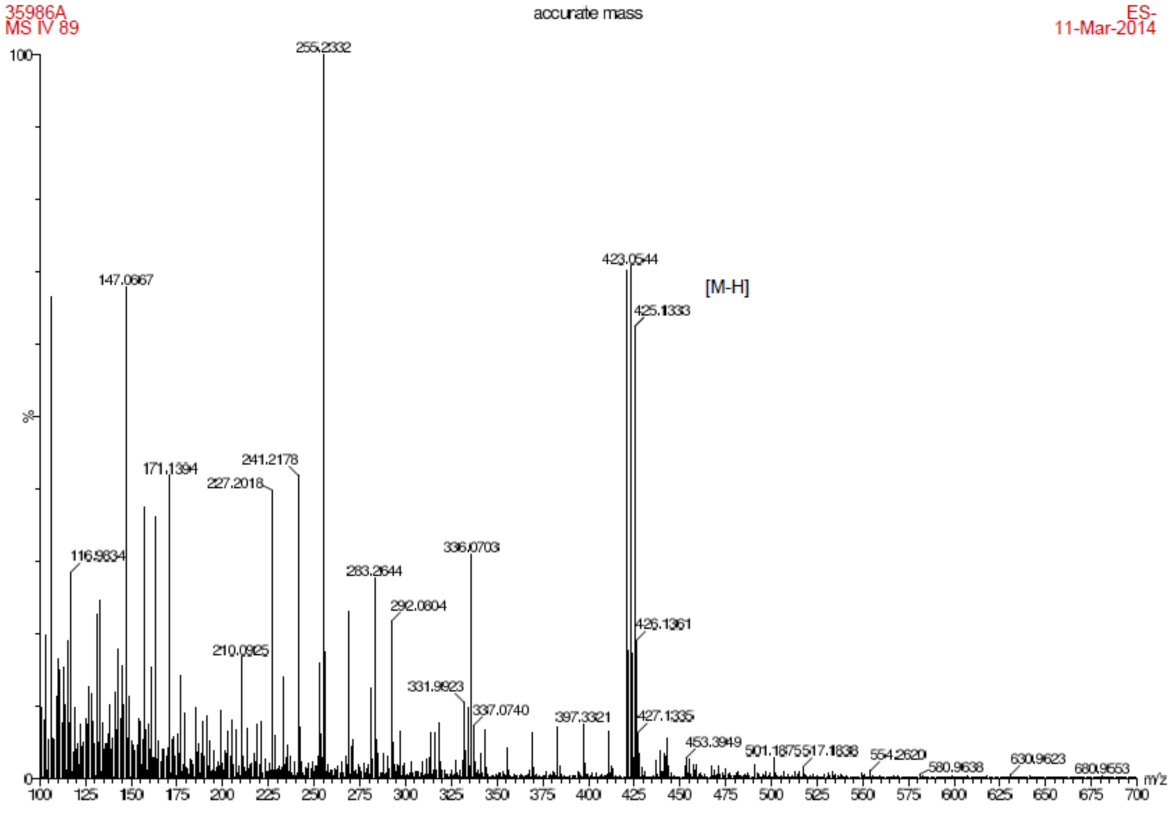

^{1}H NMR (300 MHz, DMSO)

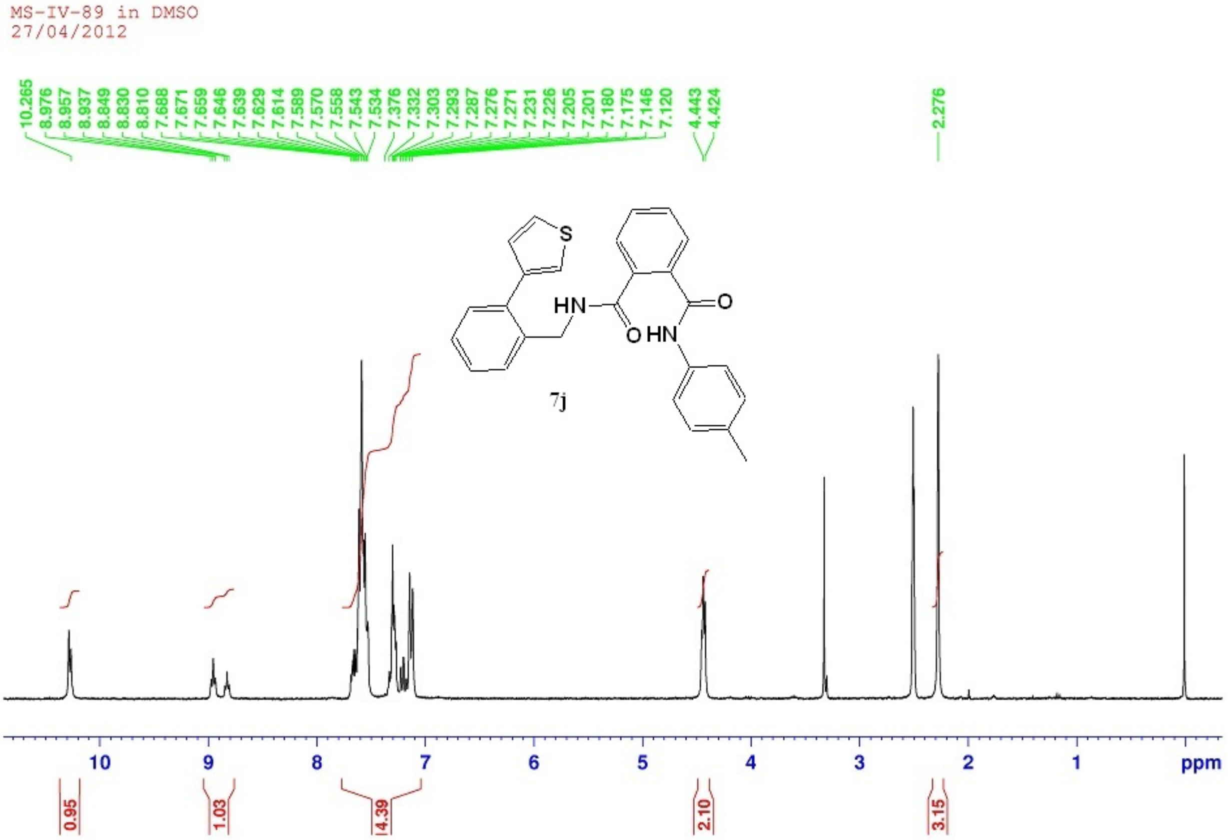

^{13}C NMR (75 MHz, DMSO)

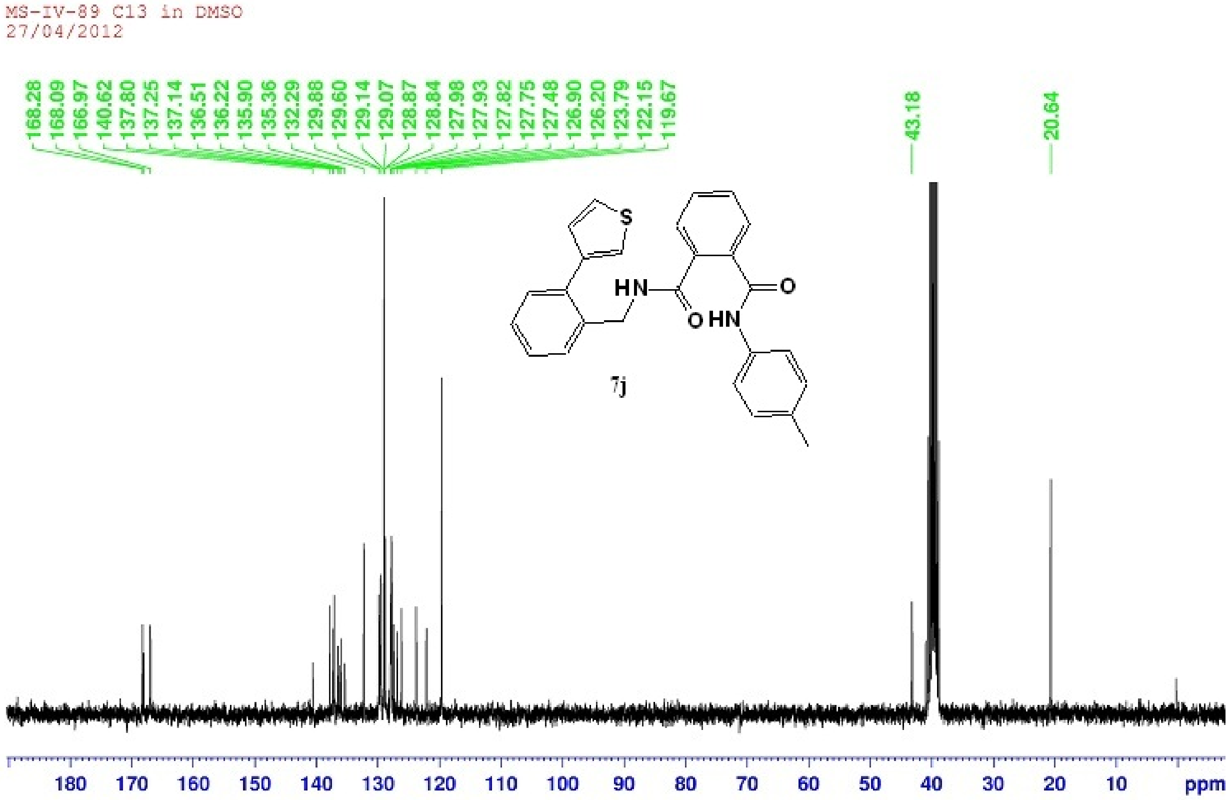

N^1-(4-fluorophenyl)-N^2-(4-(thiophen-3-yl)benzyl)phthalamide (7k)

HRMS

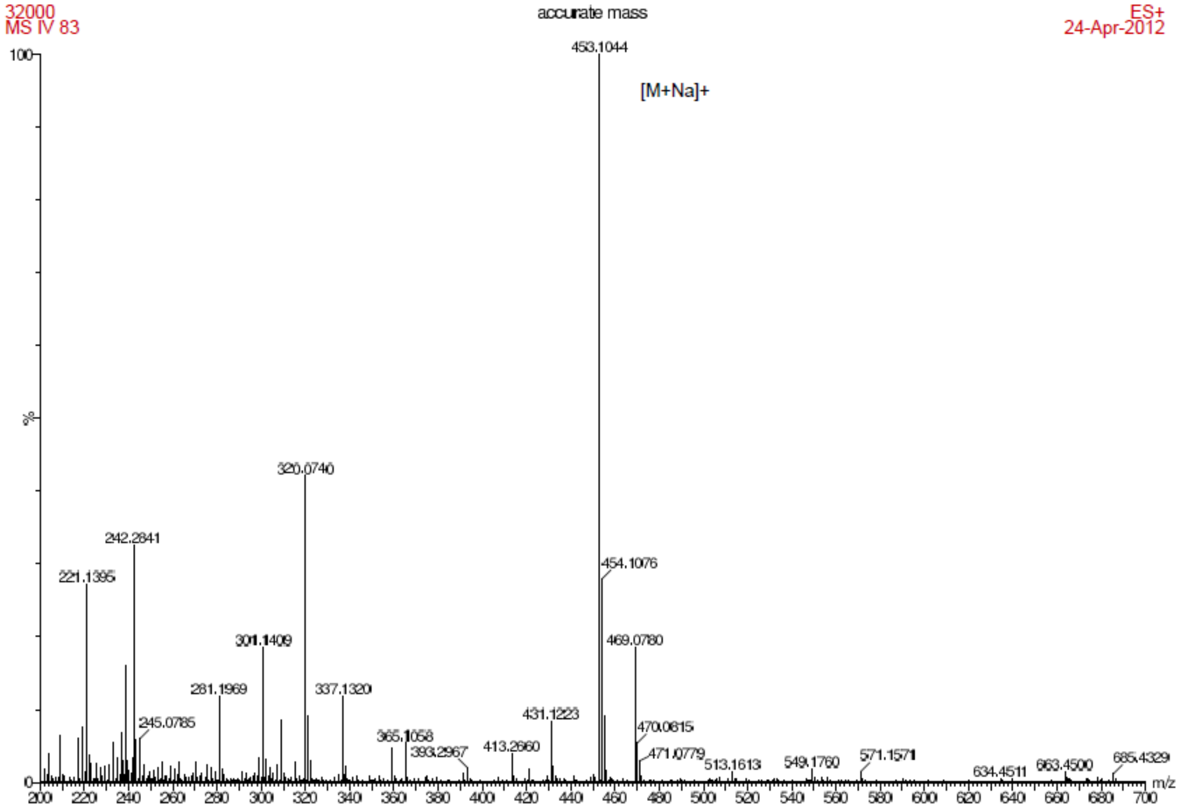

^{1}H NMR (500 MHz, DMSO)

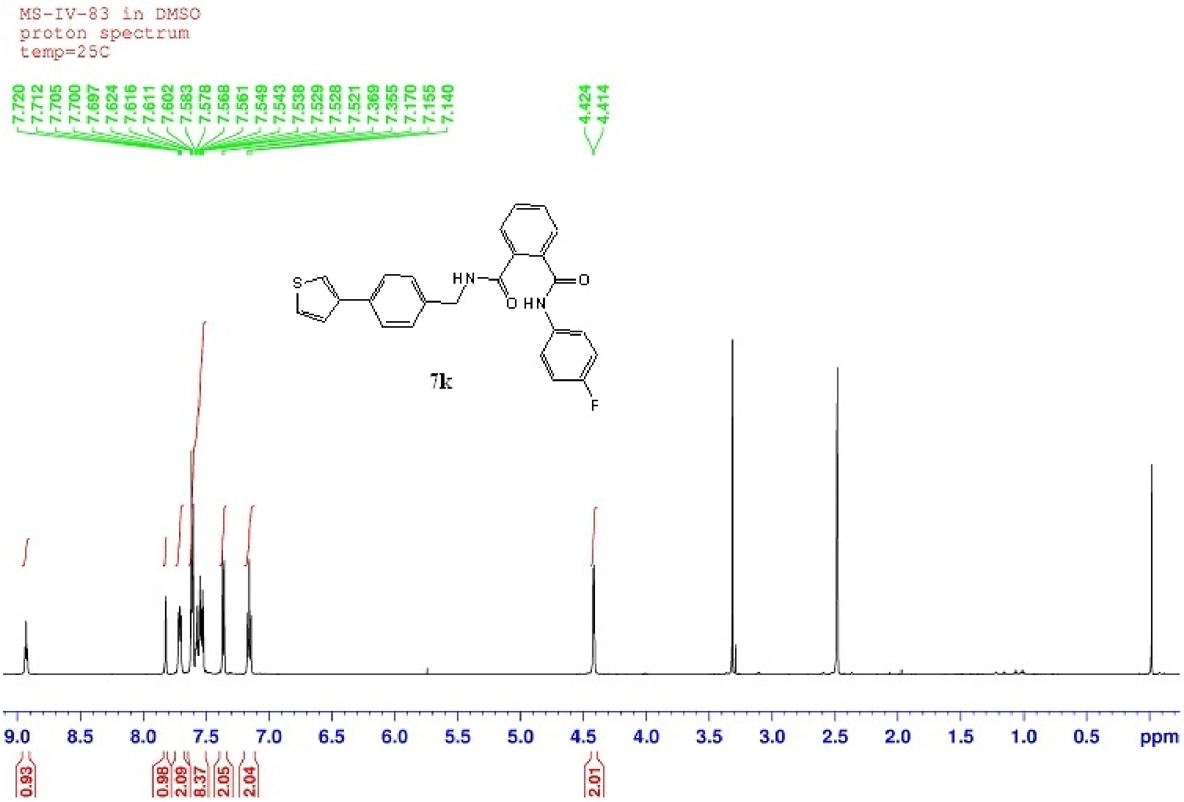

^{13}C NMR (125 MHz, DMSO)

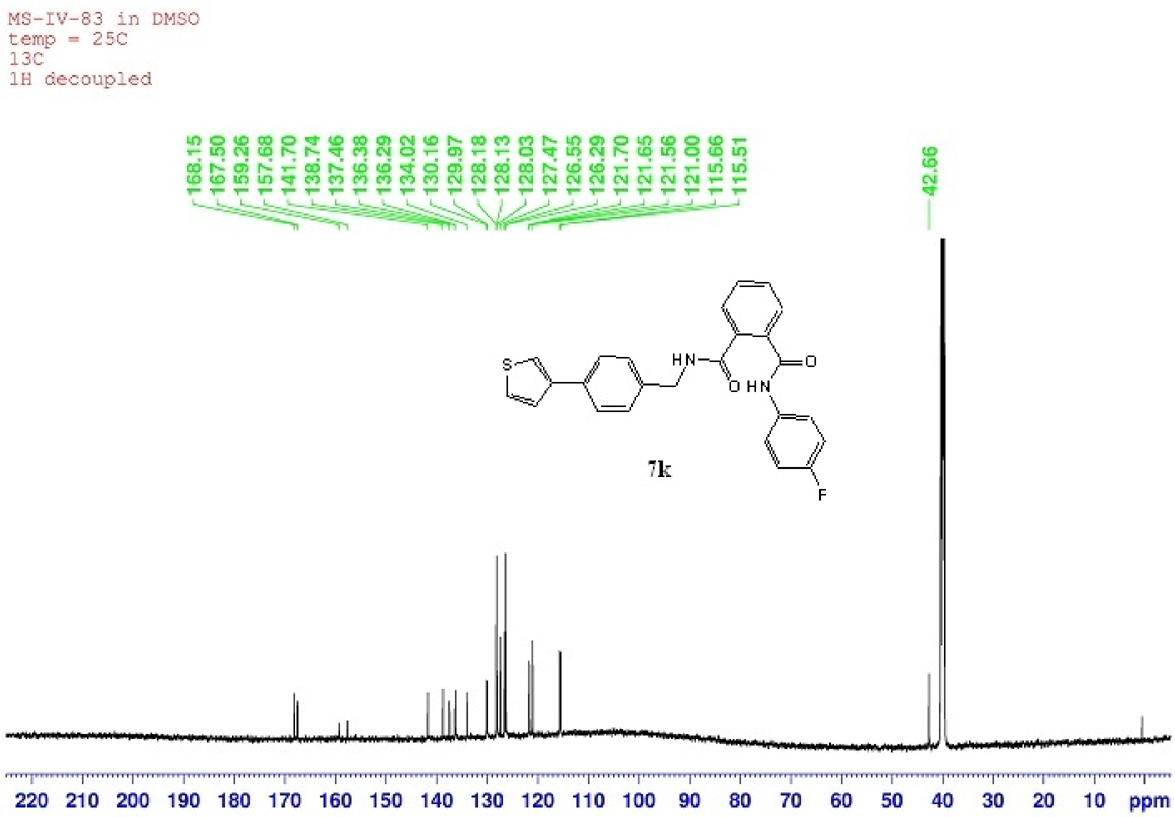

N[1]-(4-(thiophen-3-yl)benzyl)-*N*[2]-(p-tolyl)phthalamide (7l):

HRMS

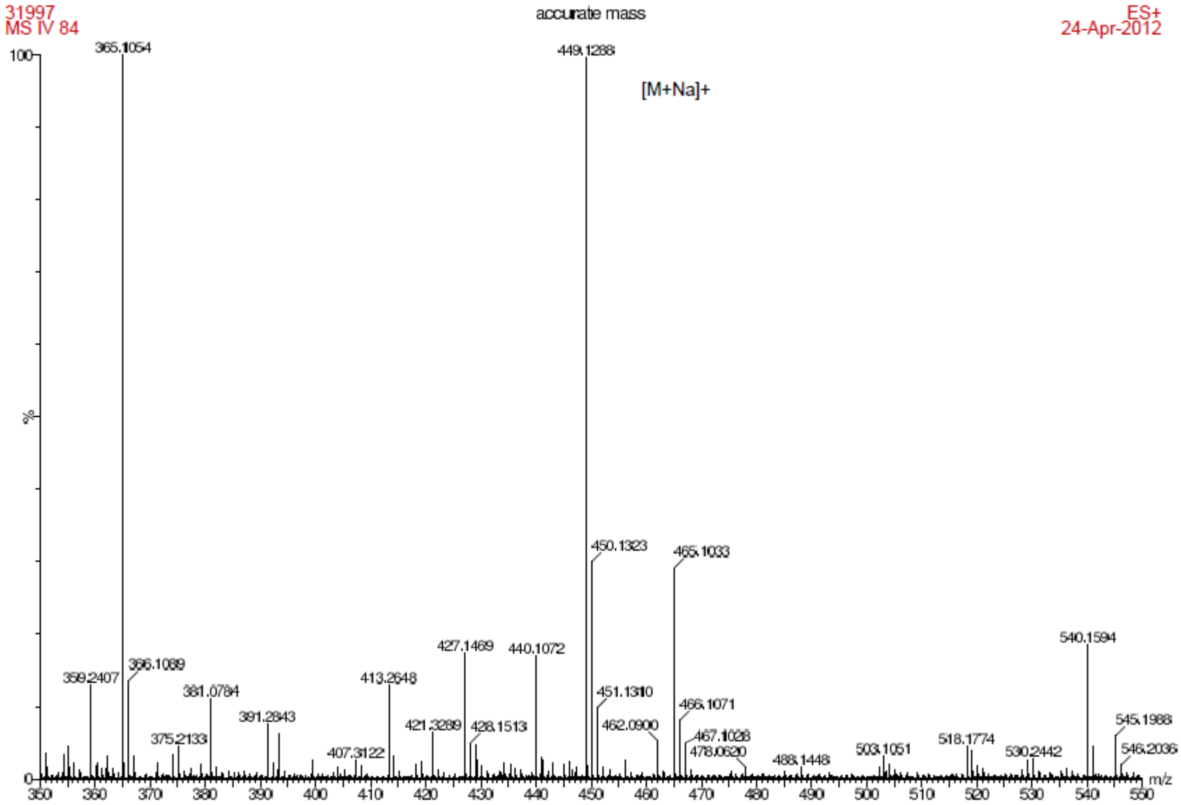

^{1}H NMR (300 MHz, DMSO)

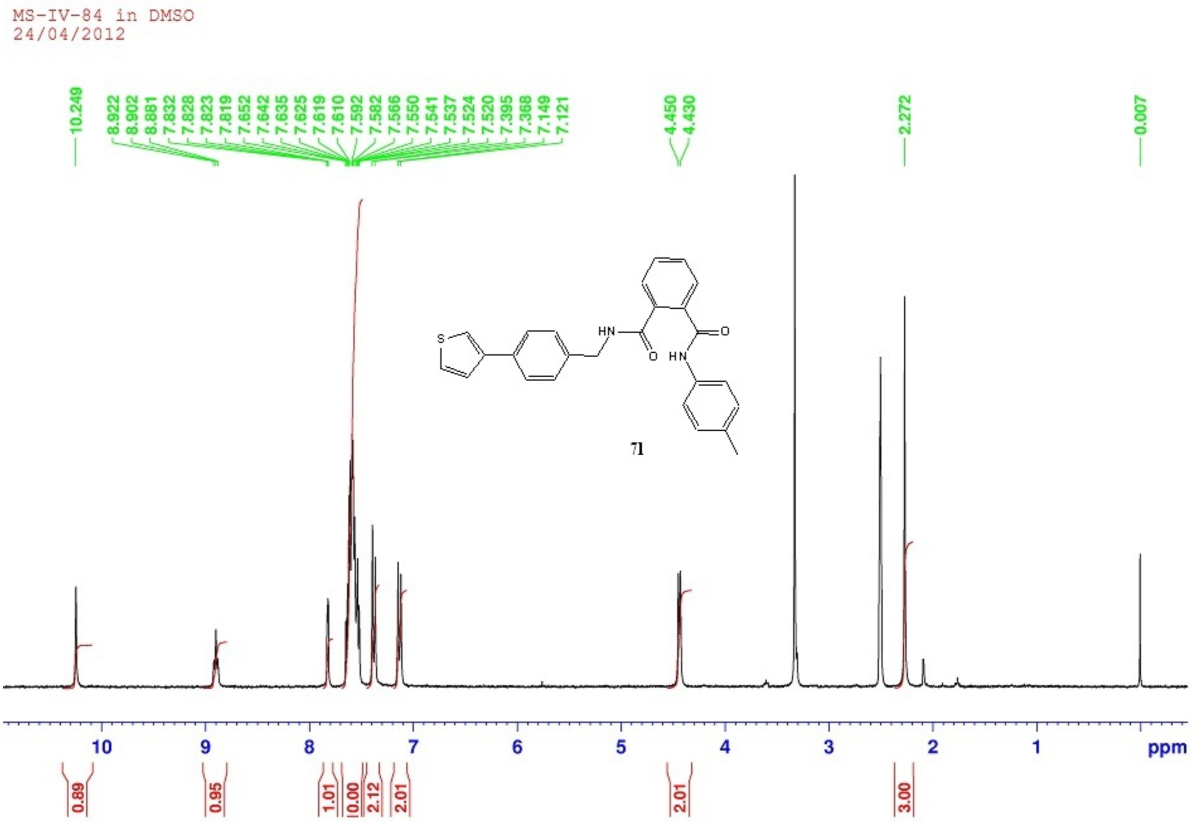

^{13}C NMR (75 MHz, DMSO)

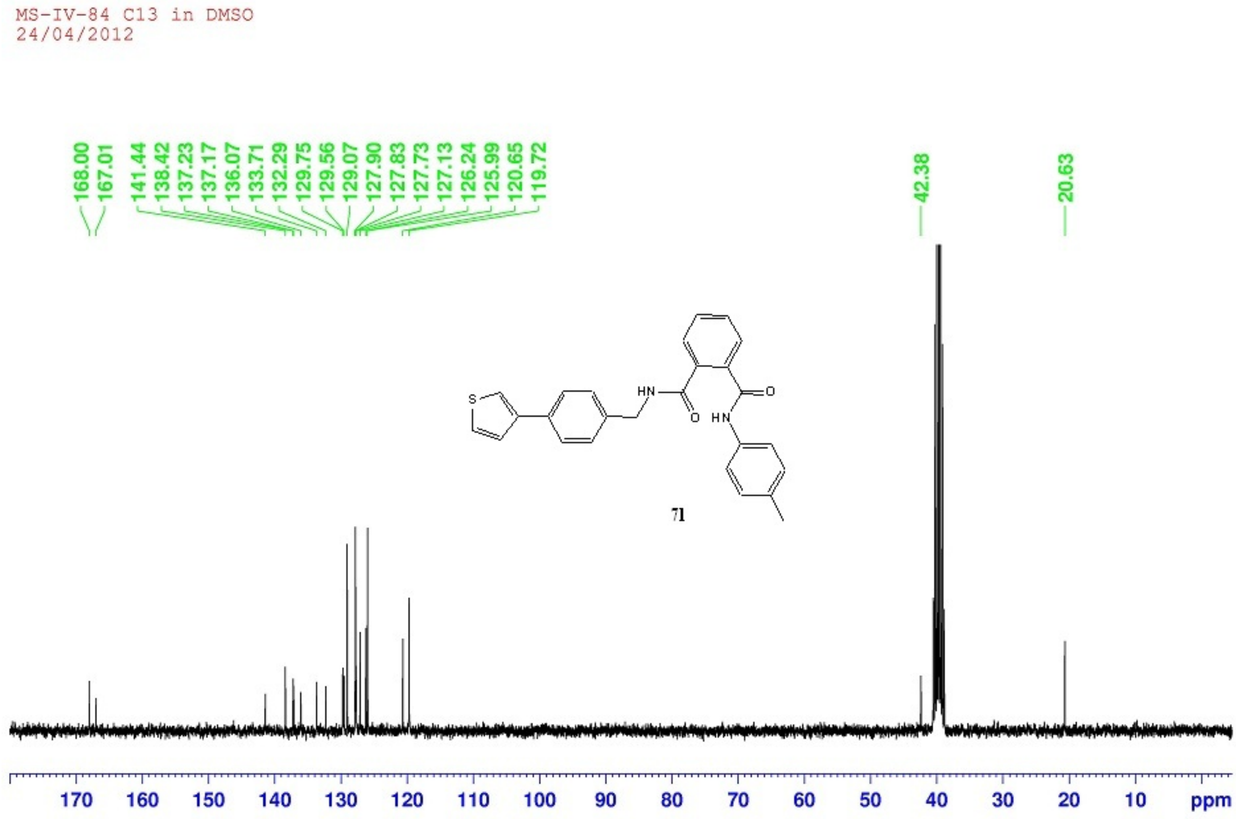

N[1]-(4-fluorophenyl)-*N*[2]-(2-(3-methyl-1H-pyrazol-5-yl)phenyl)phthalamide (7m):
HRMS

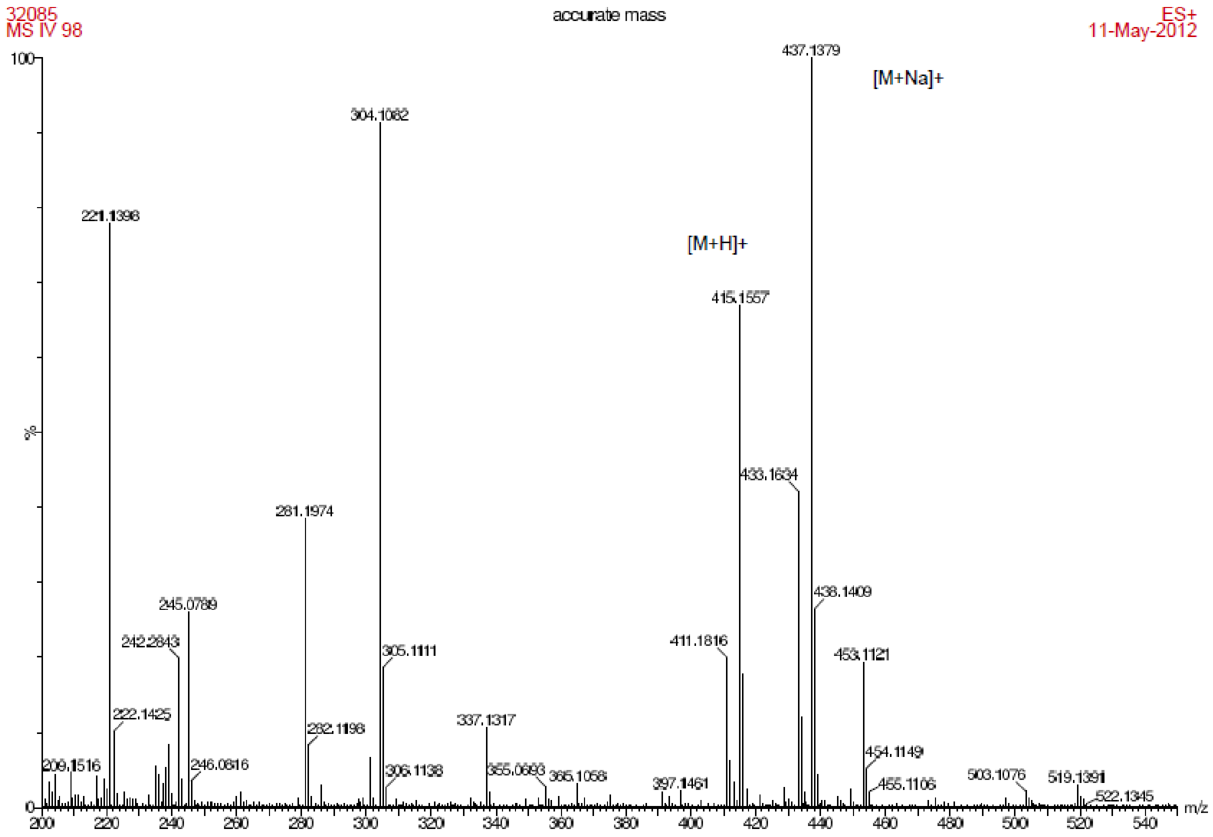

^{1}H NMR (500 MHz, DMSO)

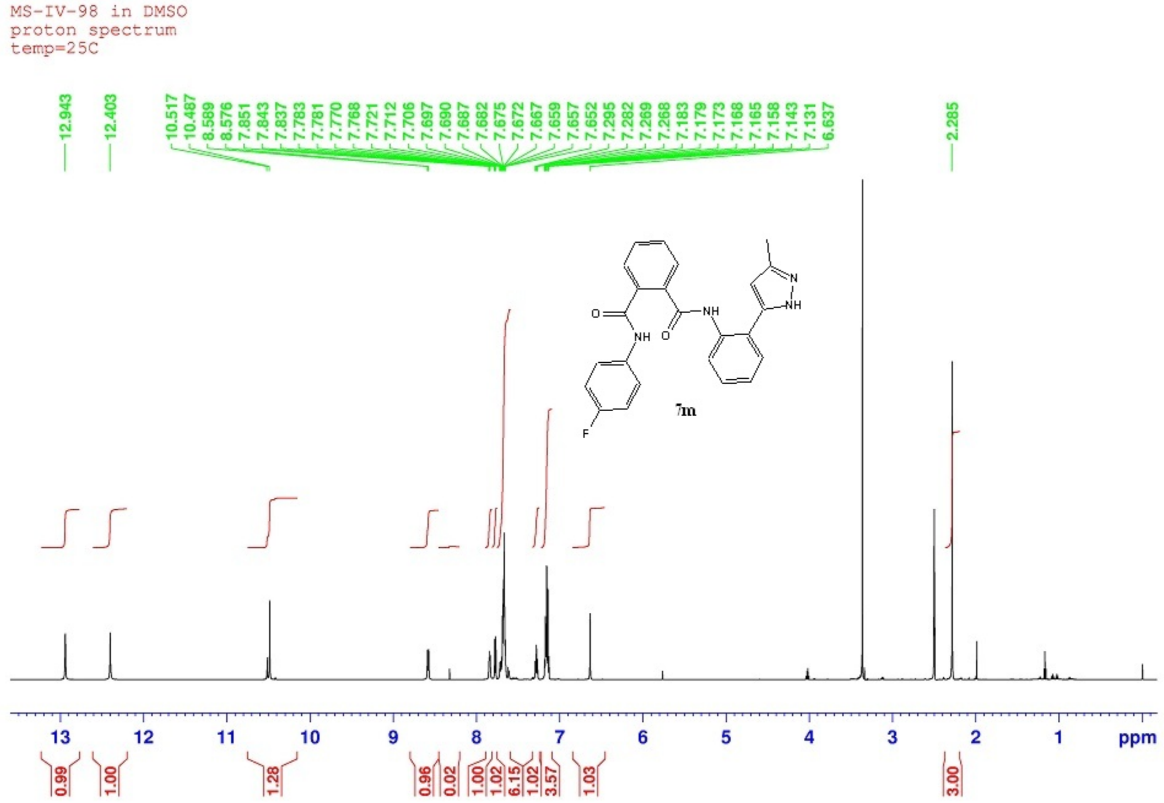

^{13}C NMR (125 MHz, DMSO)

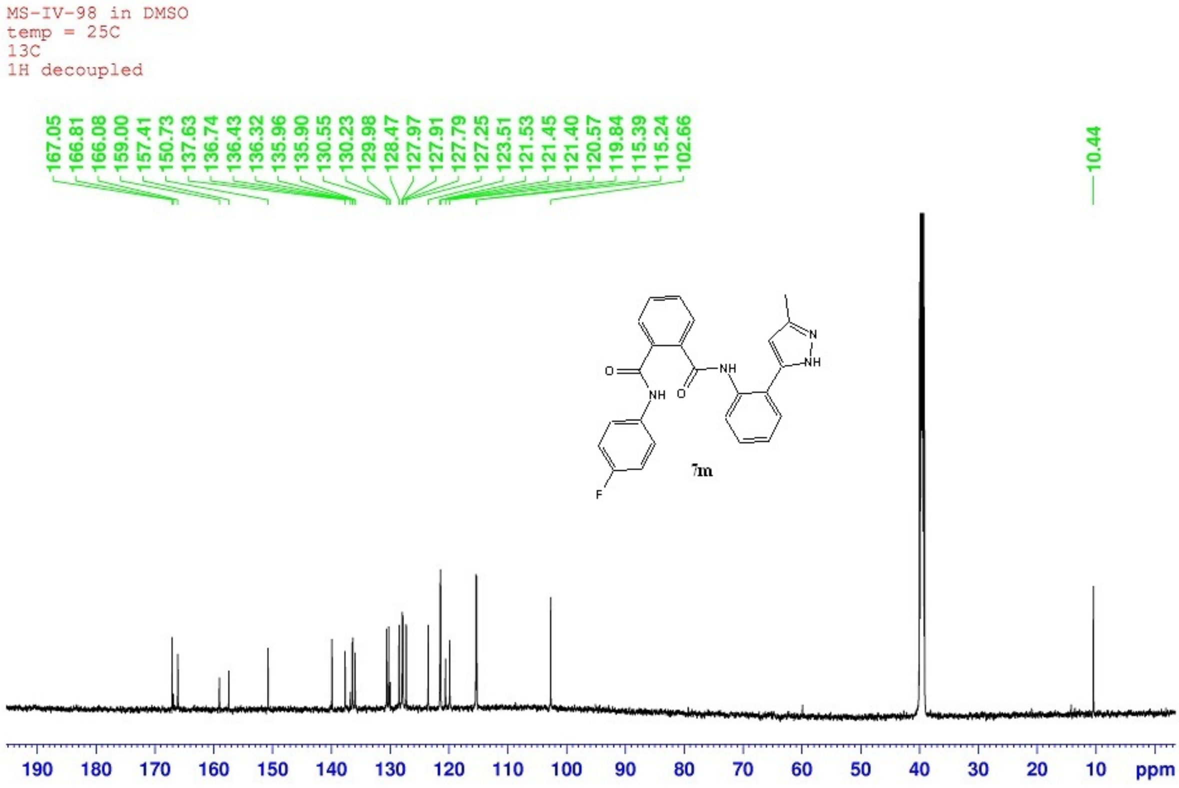

N[1]-(2-(3-methyl-1H-pyrazol-5-yl)phenyl)-*N*[2]-(p-tolyl)phthalamide (7n)

HRMS

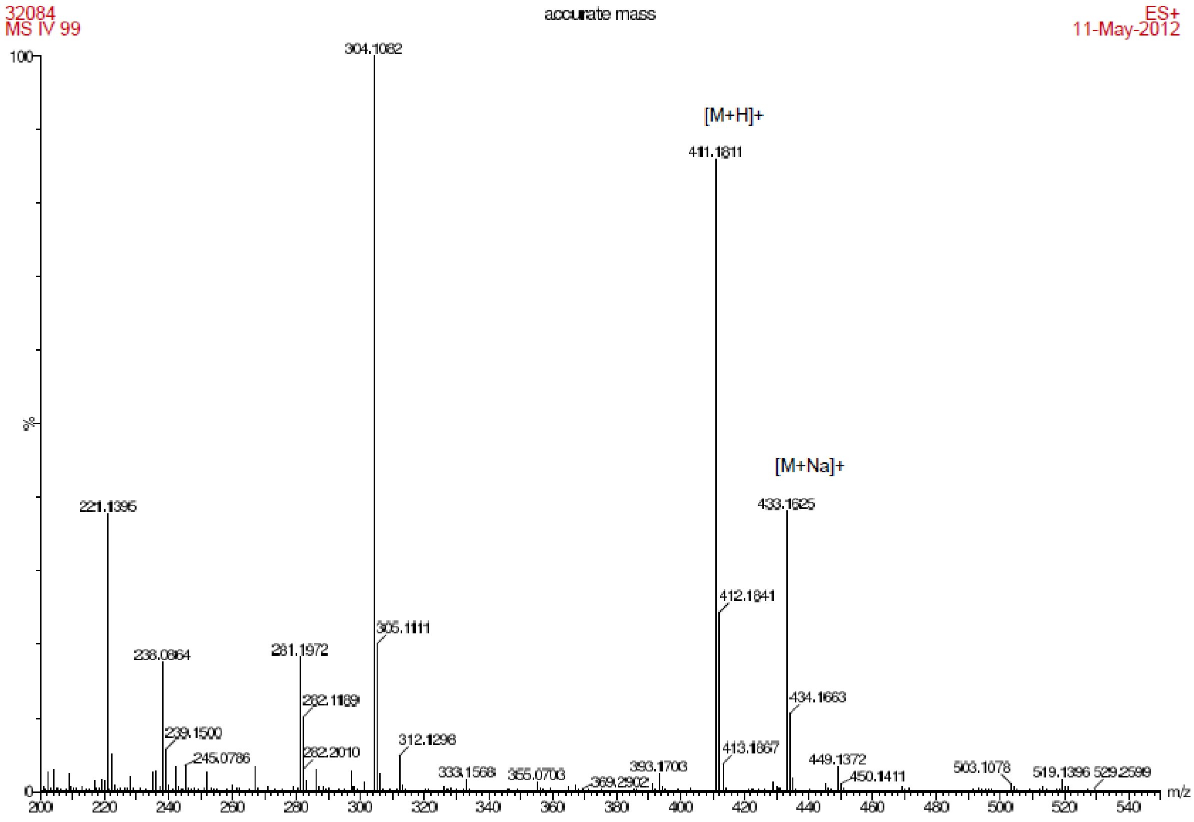

^{1}H NMR (500 MHz, DMSO)

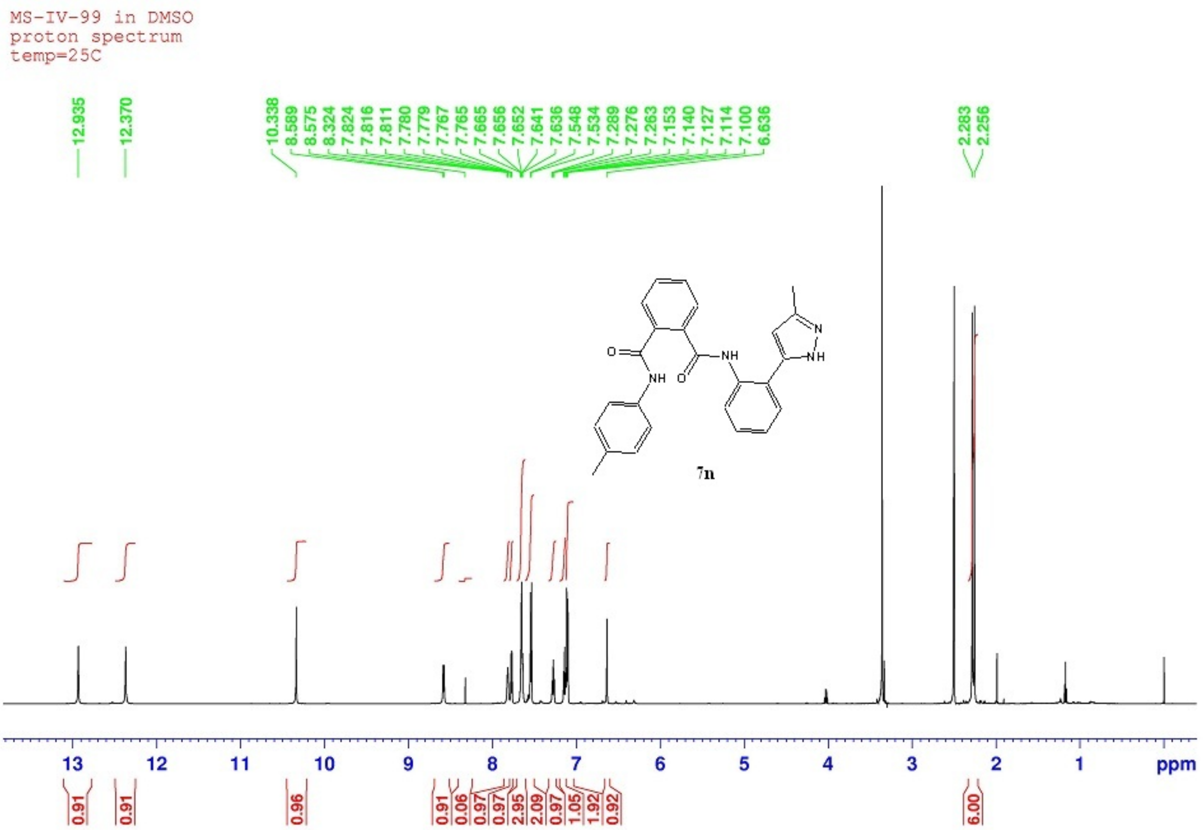

^{13}C NMR (125 MHz, DMSO)

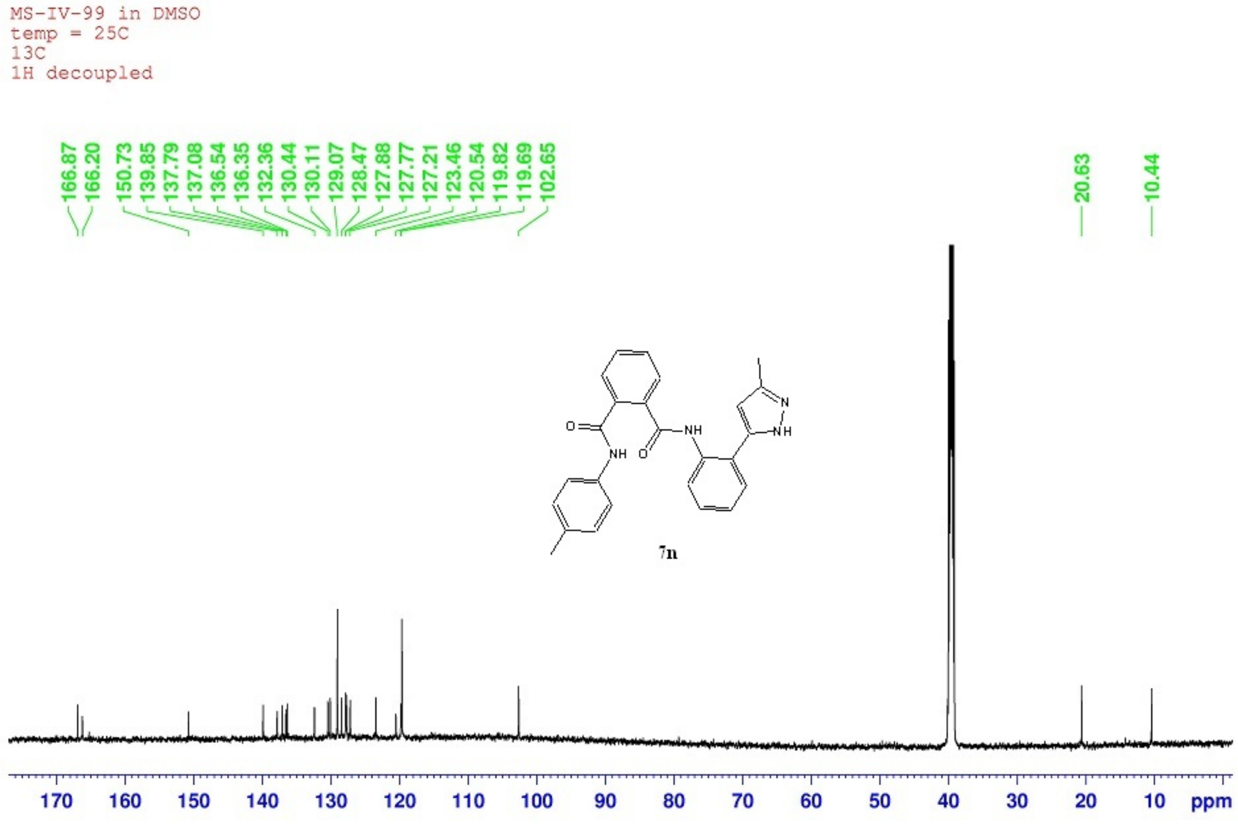

2-(1,3-dioxoisoindolin-2-yl)benzoic acid (9)

HRMS

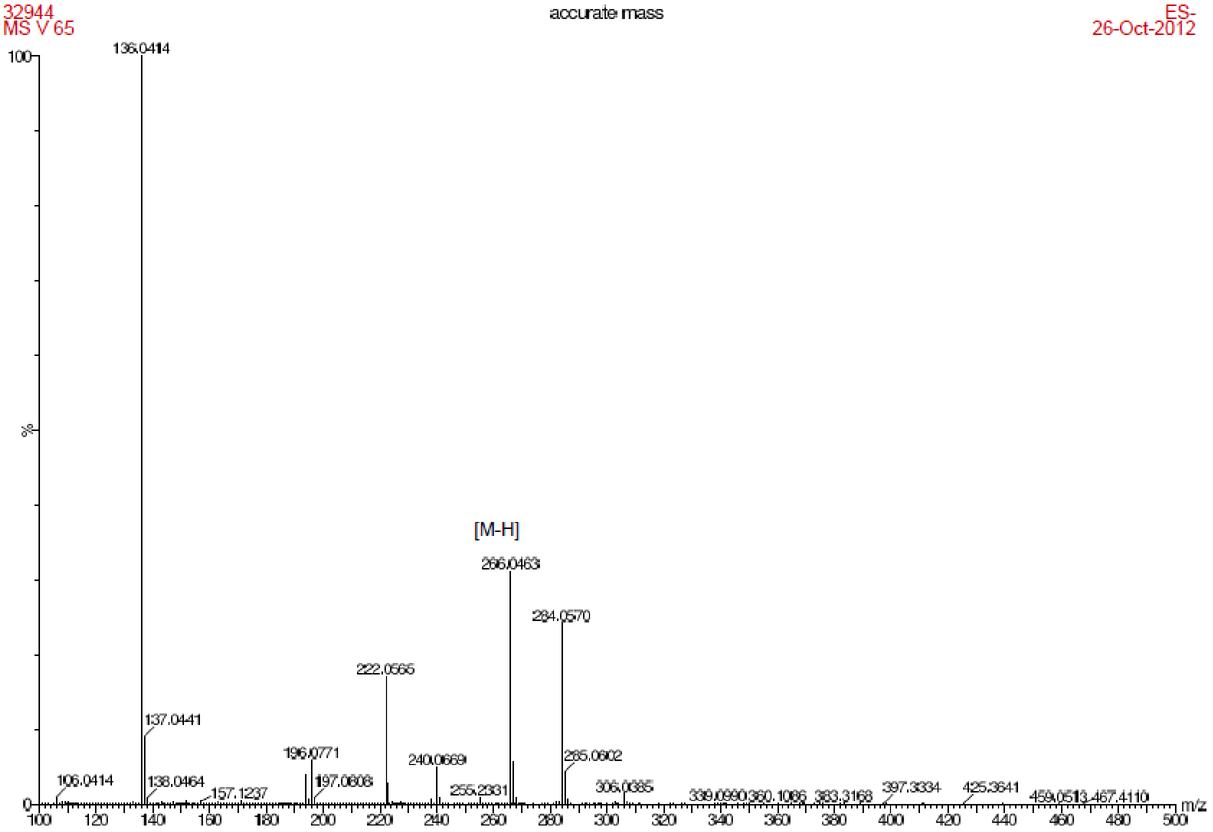

^{1}H NMR (300 MHz, DMSO)

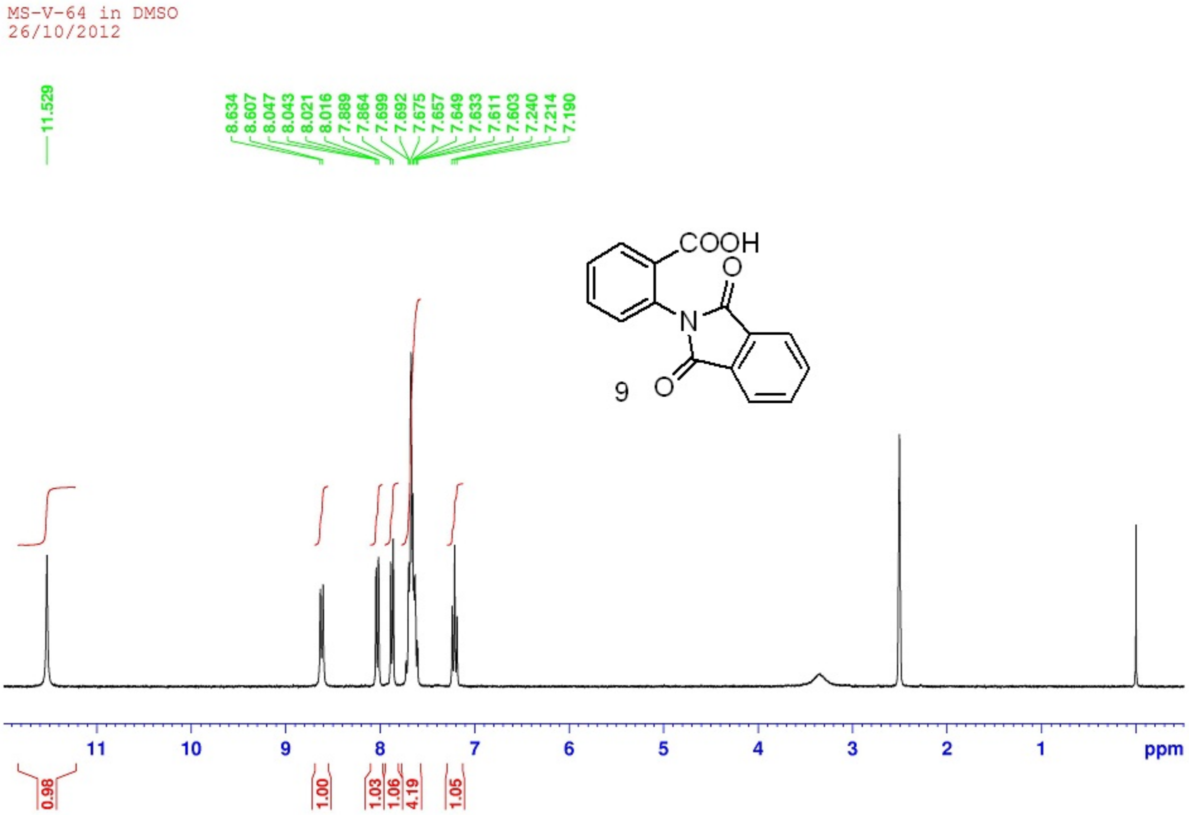

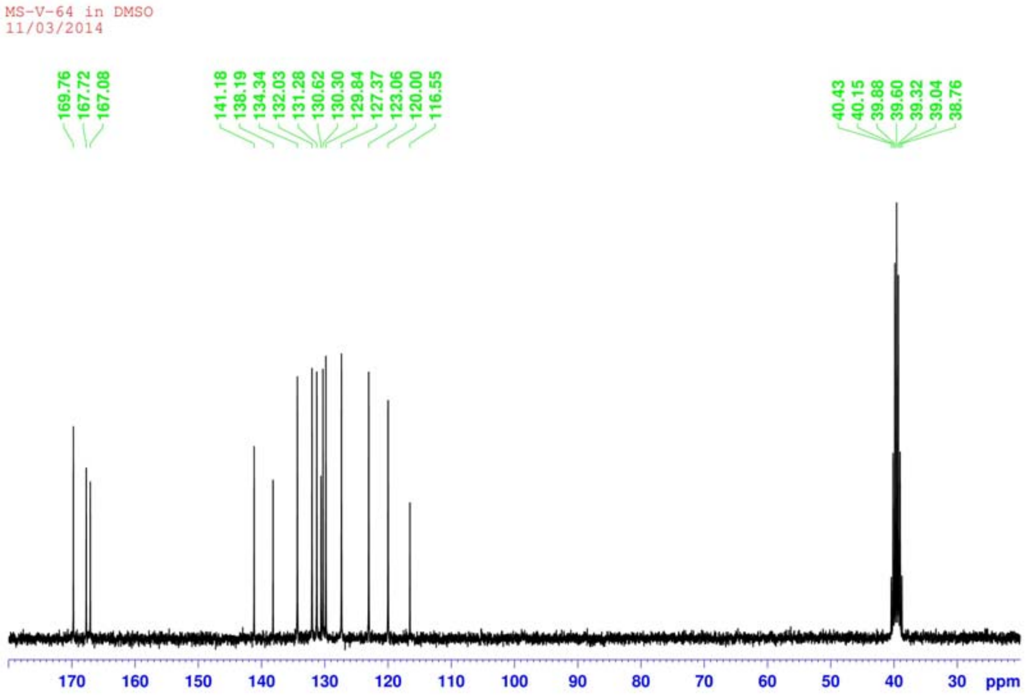

N-(2-bromo-4-methylphenyl)-2-(1,3-dioxoisoindolin-2-yl)benzamide (10):
HRMS

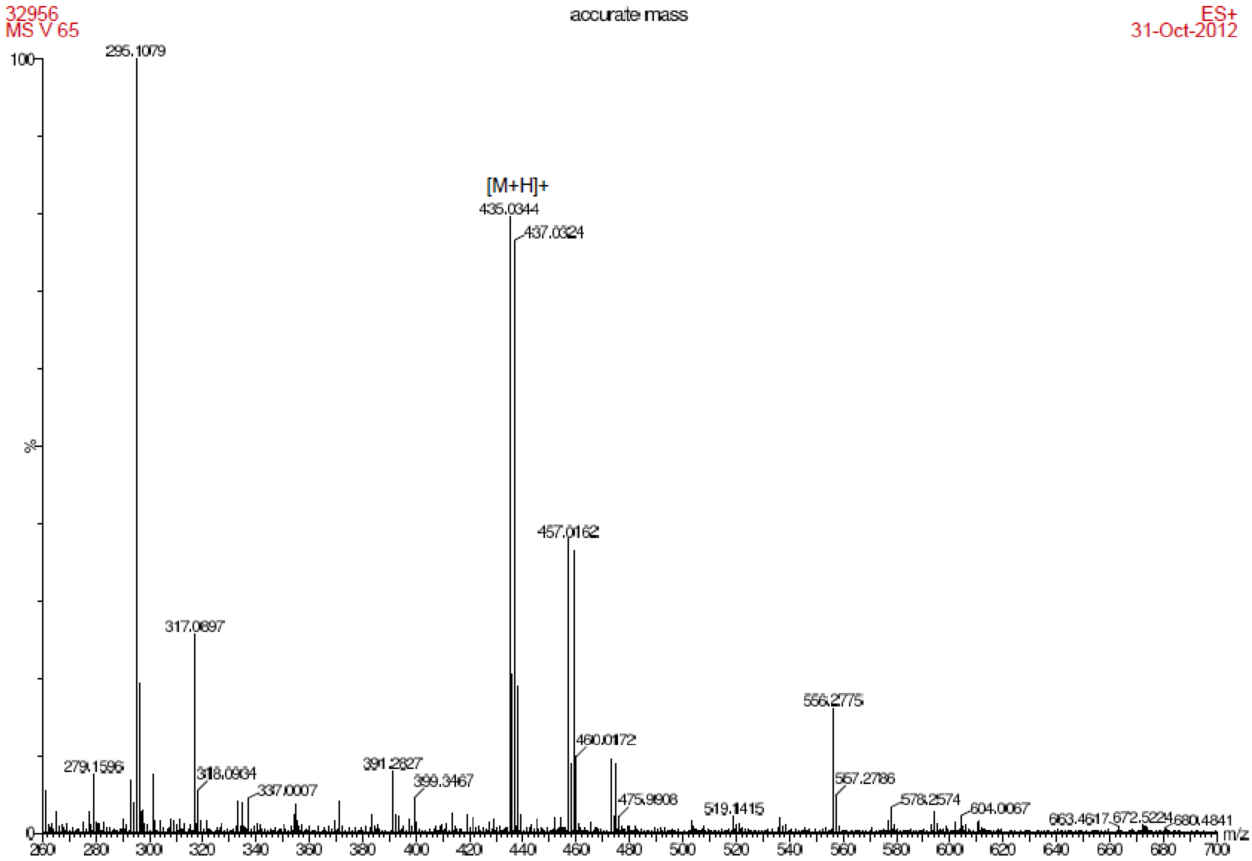

^{1}H NMR (300 MHz, $CDCl_3$)

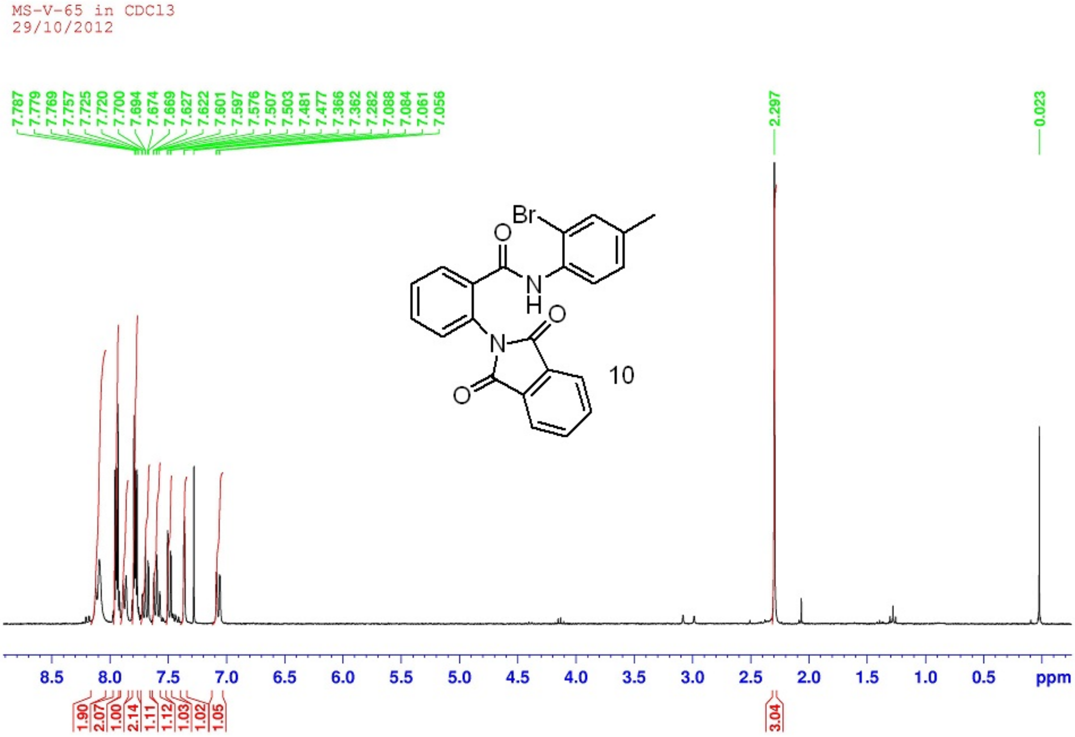

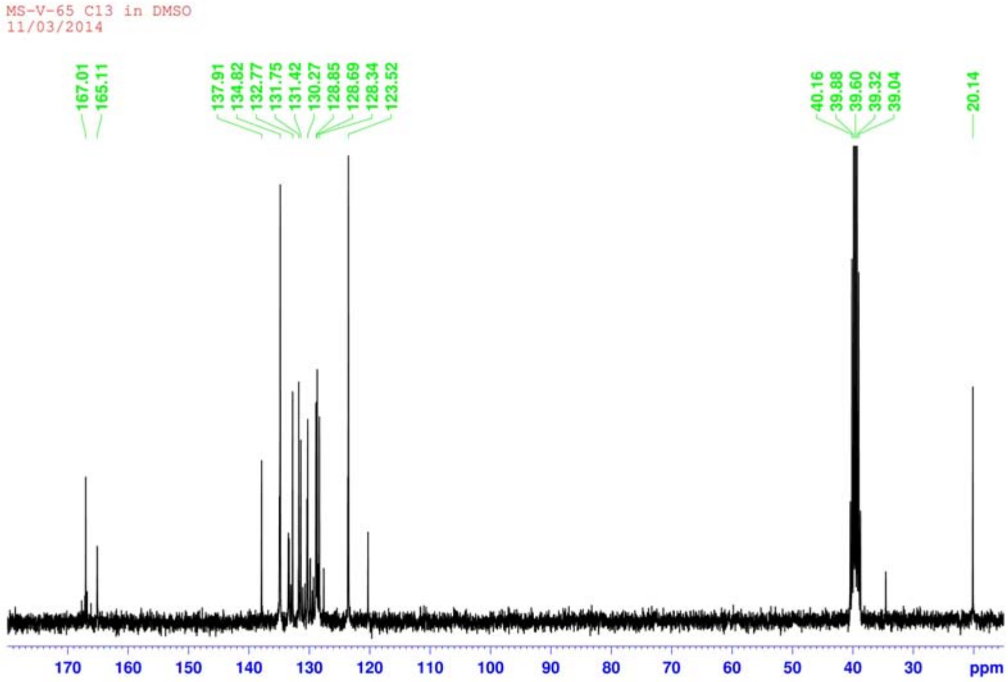

2-amino-*N*-(2-bromo-4-methylphenyl)benzamide (11)

HRMS

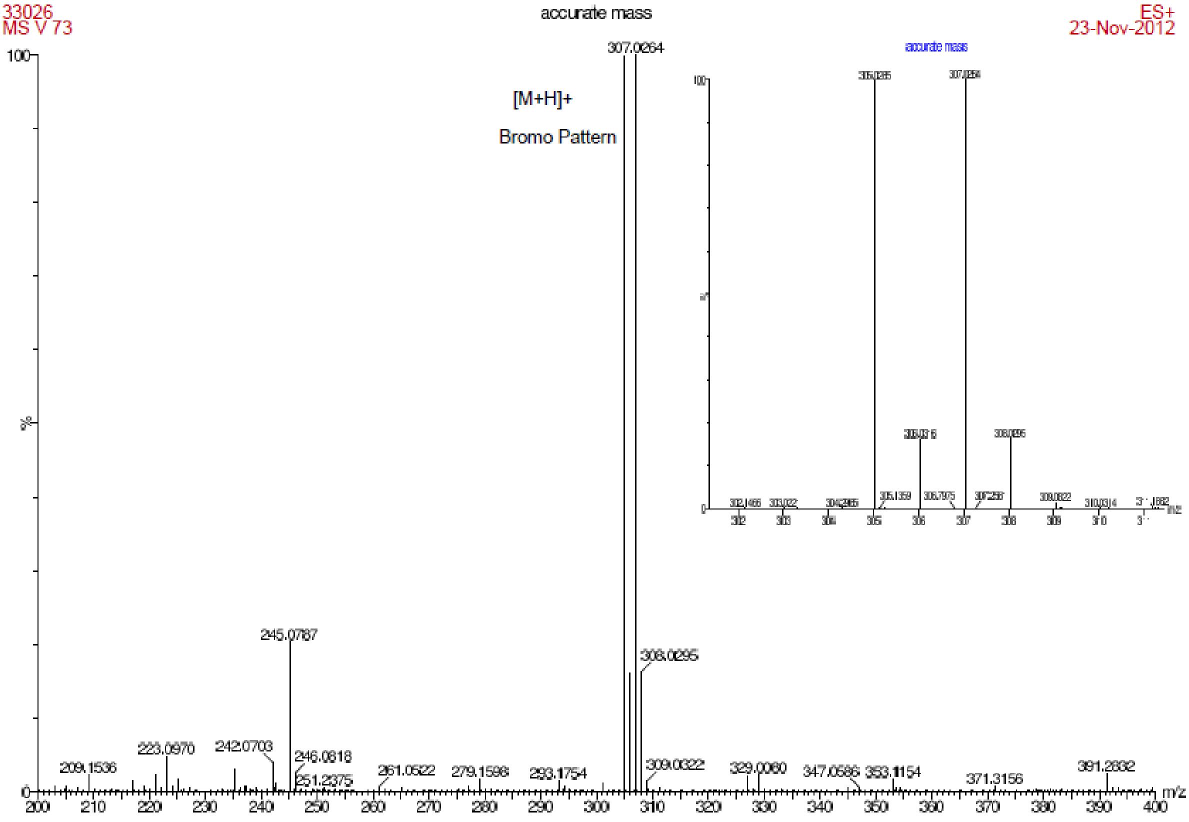

^{1}H NMR (300 MHz, DMSO)

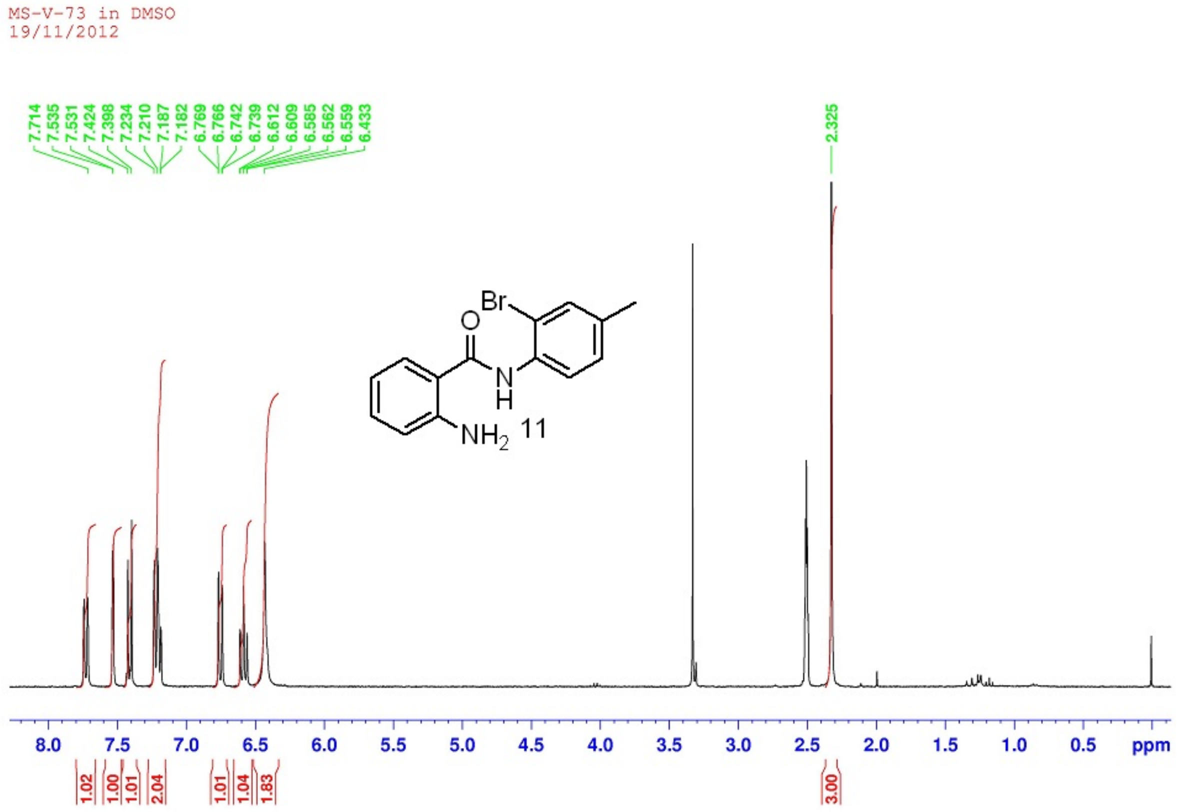

N-(2-bromo-4-methylphenyl)-2-(4-fluorobenzamido)benzamide (12)

HRMS

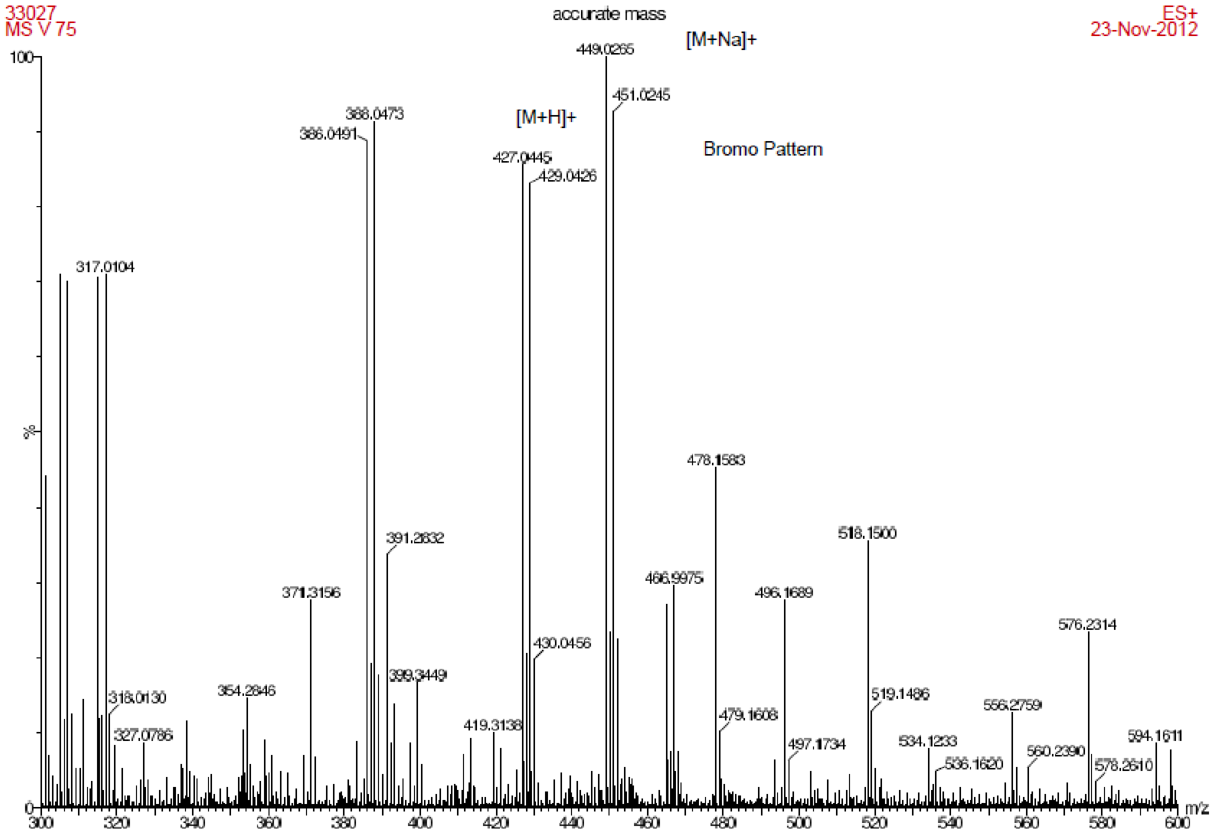

^{1}H NMR (500 MHz, $CDCl_3$)

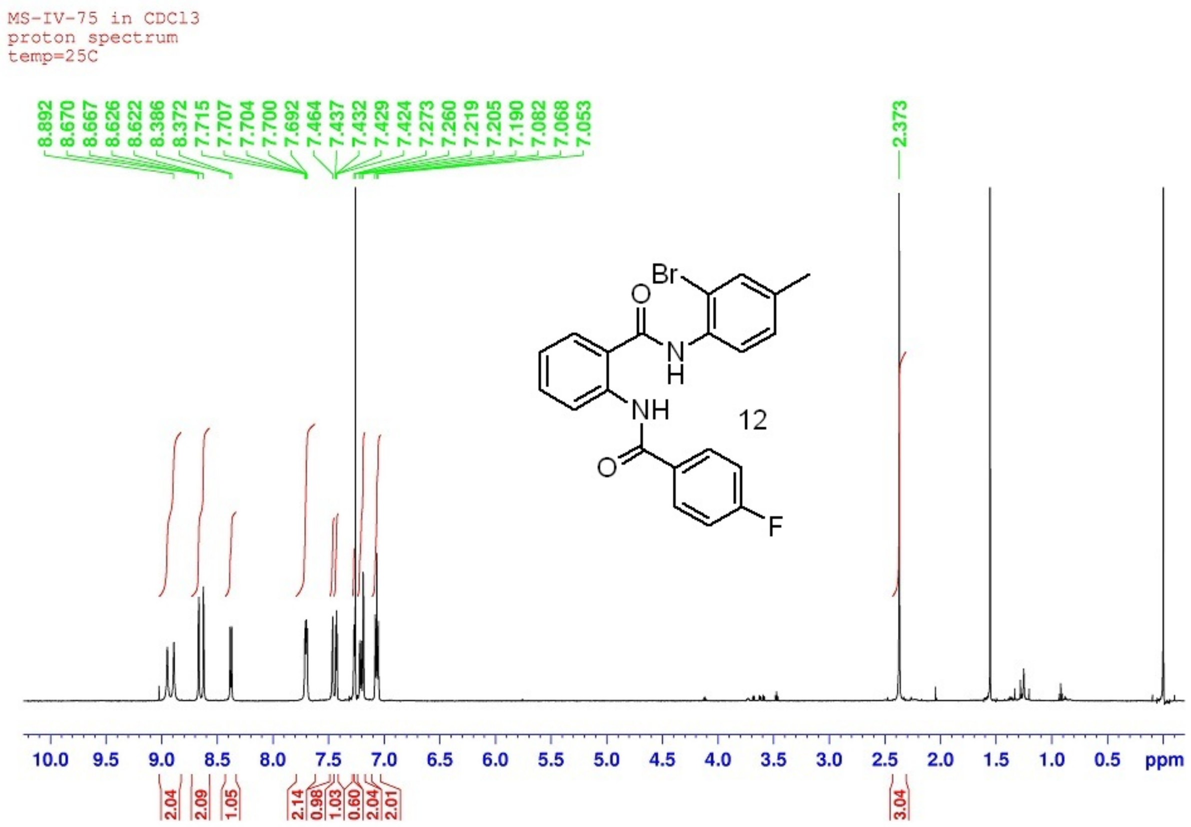

^{13}C NMR (125 MHz, $CDCl_3$)

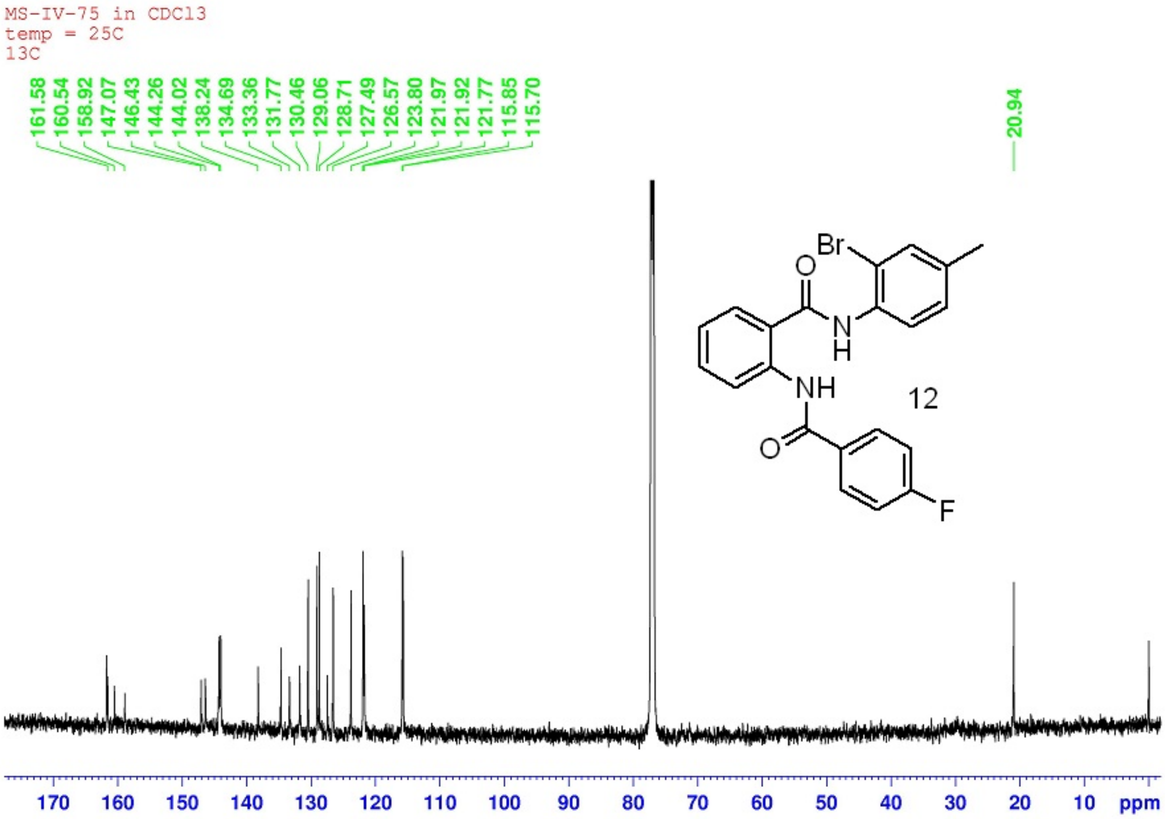

2-(4-fluorobenzamido)-*N*-(4-methyl-2-(thiophen-3-yl)phenyl)benzamide (13a)

HRMS

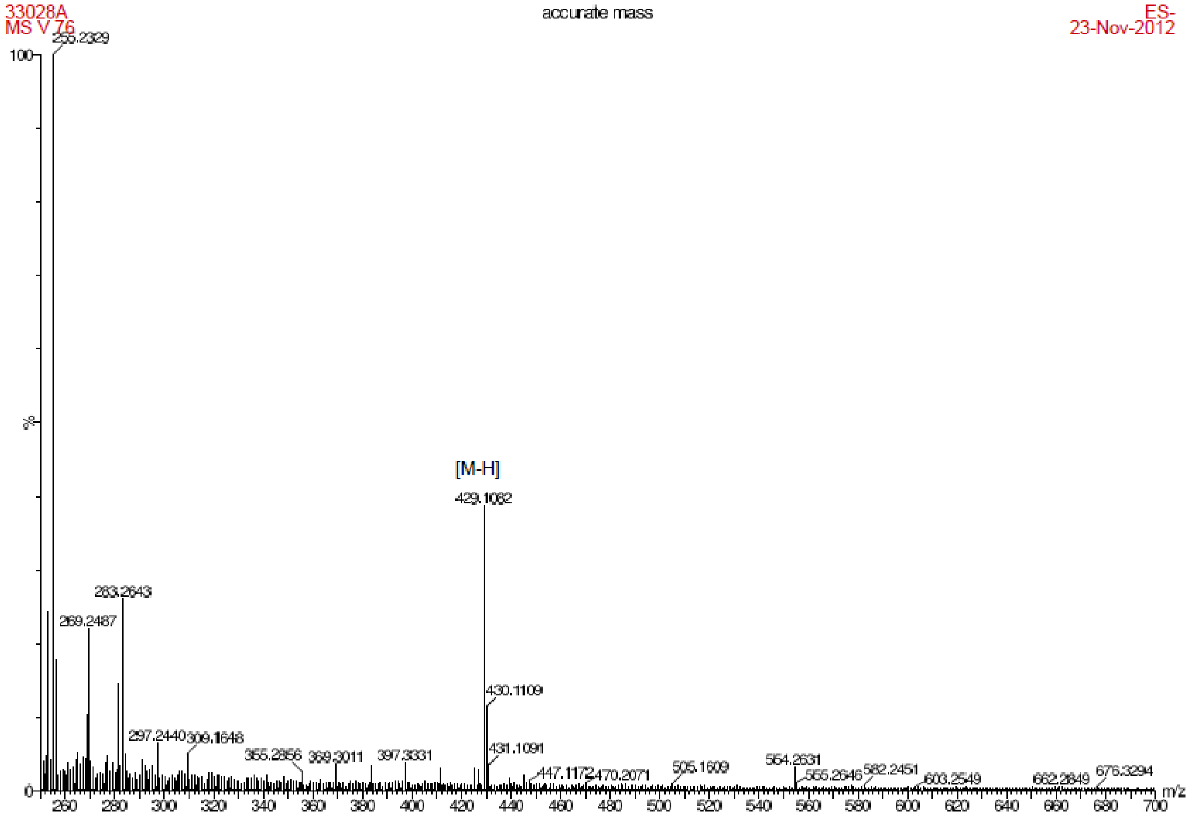

^{1}H NMR (300 MHz, $CDCl_3$)

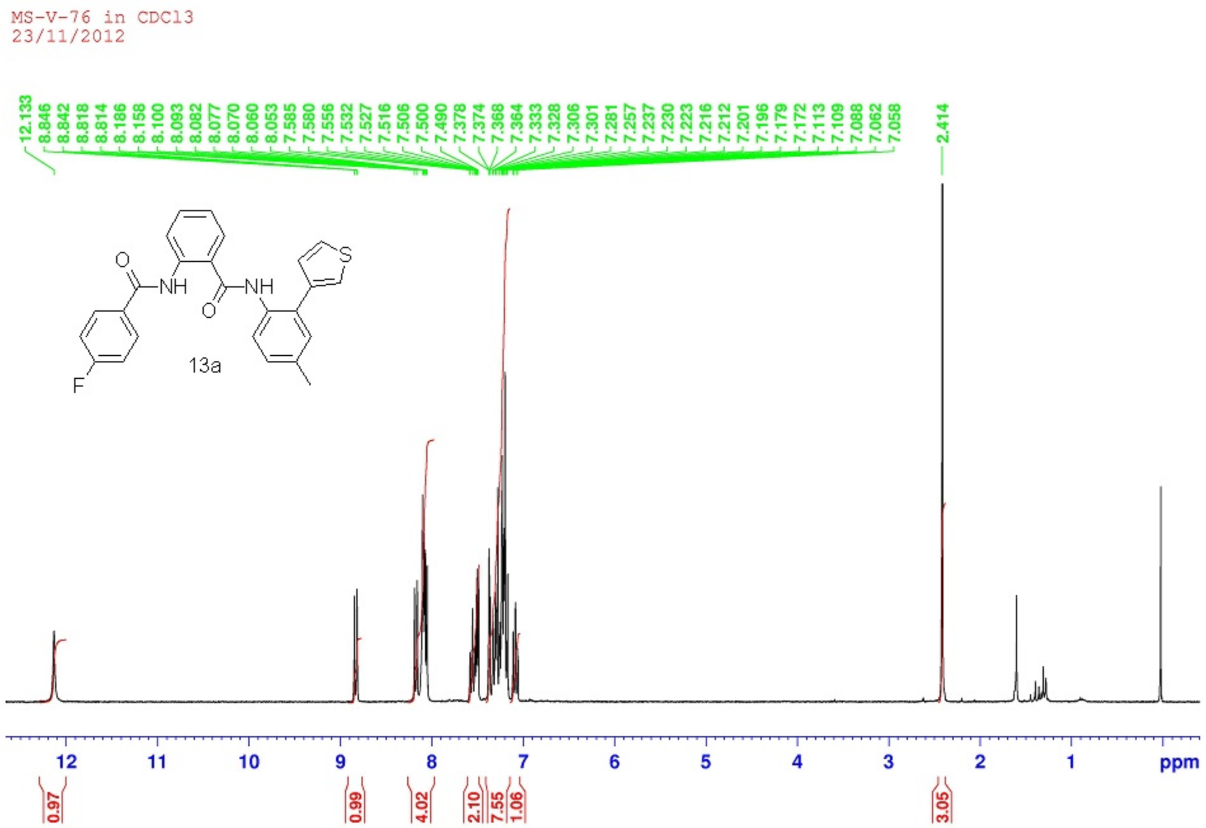

^{13}C NMR (75 MHz, $CDCl_3$)

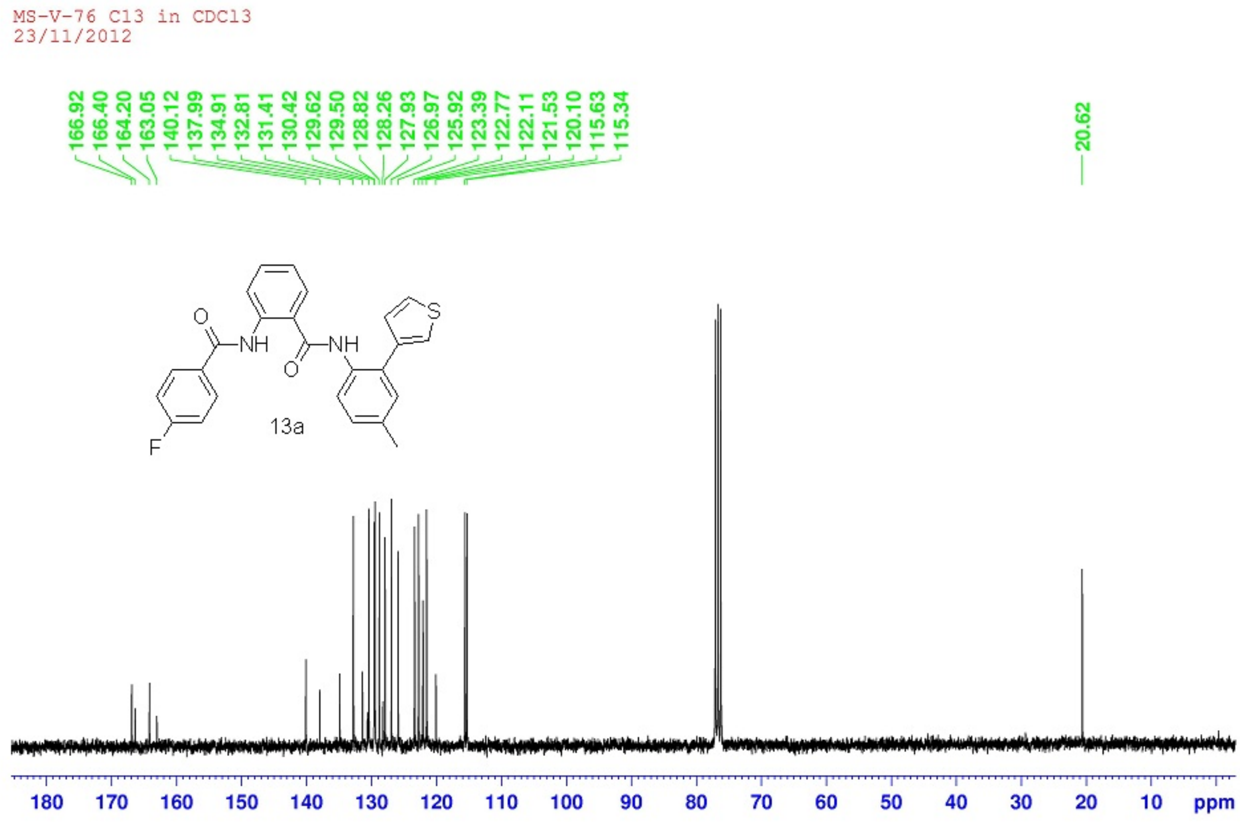

2-(4-fluorobenzamido)-*N*-(4-methyl-2-(5-methylthiophen-2-yl)phenyl)benzamide (13b)

HRMS

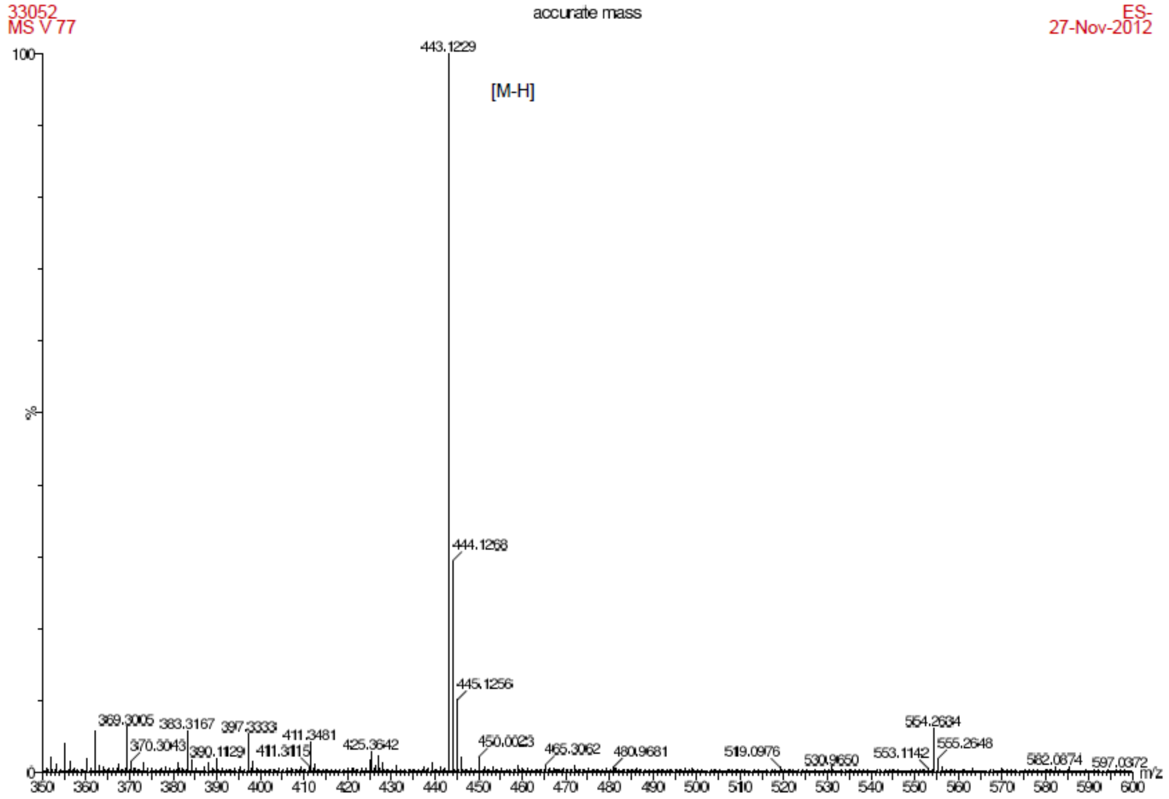

^{1}H NMR (300 MHz, $CDCl_3$)

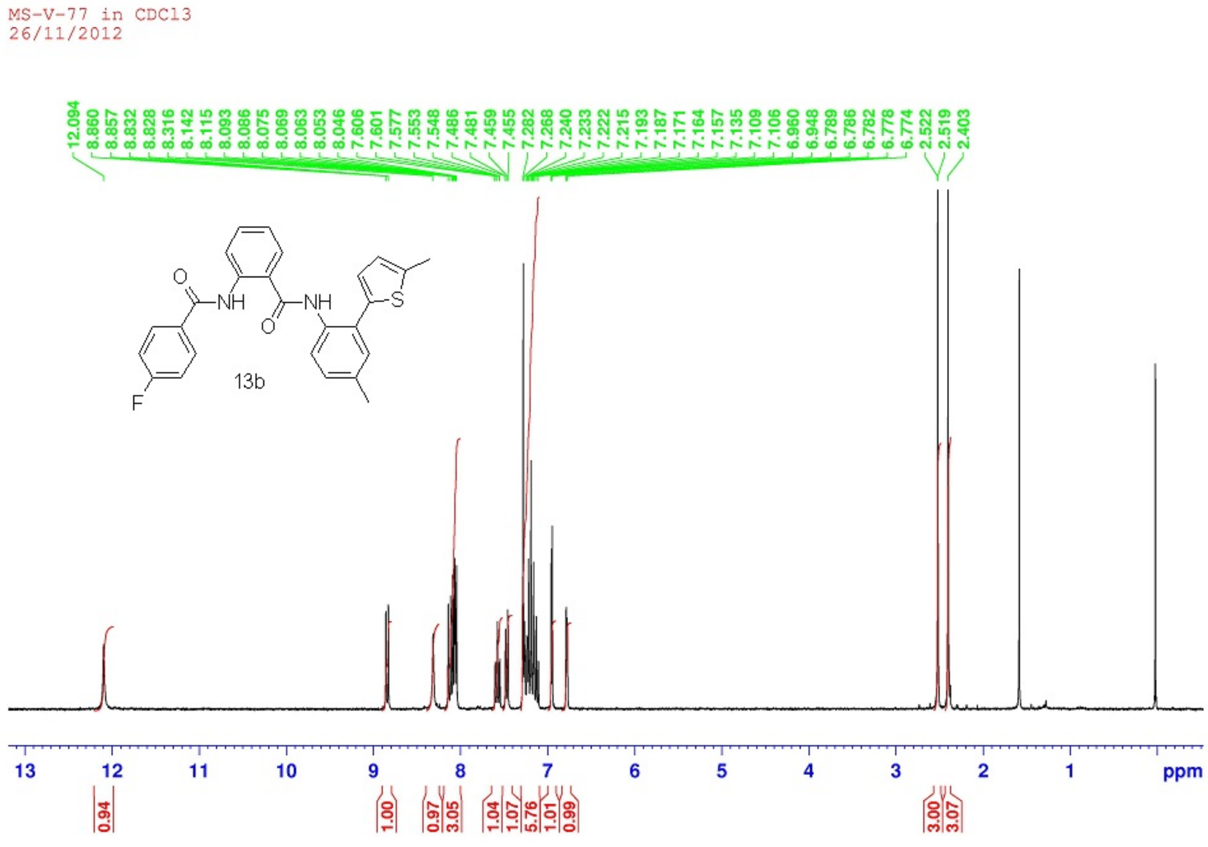

^{13}C NMR (75 MHz, $CDCl_3$)

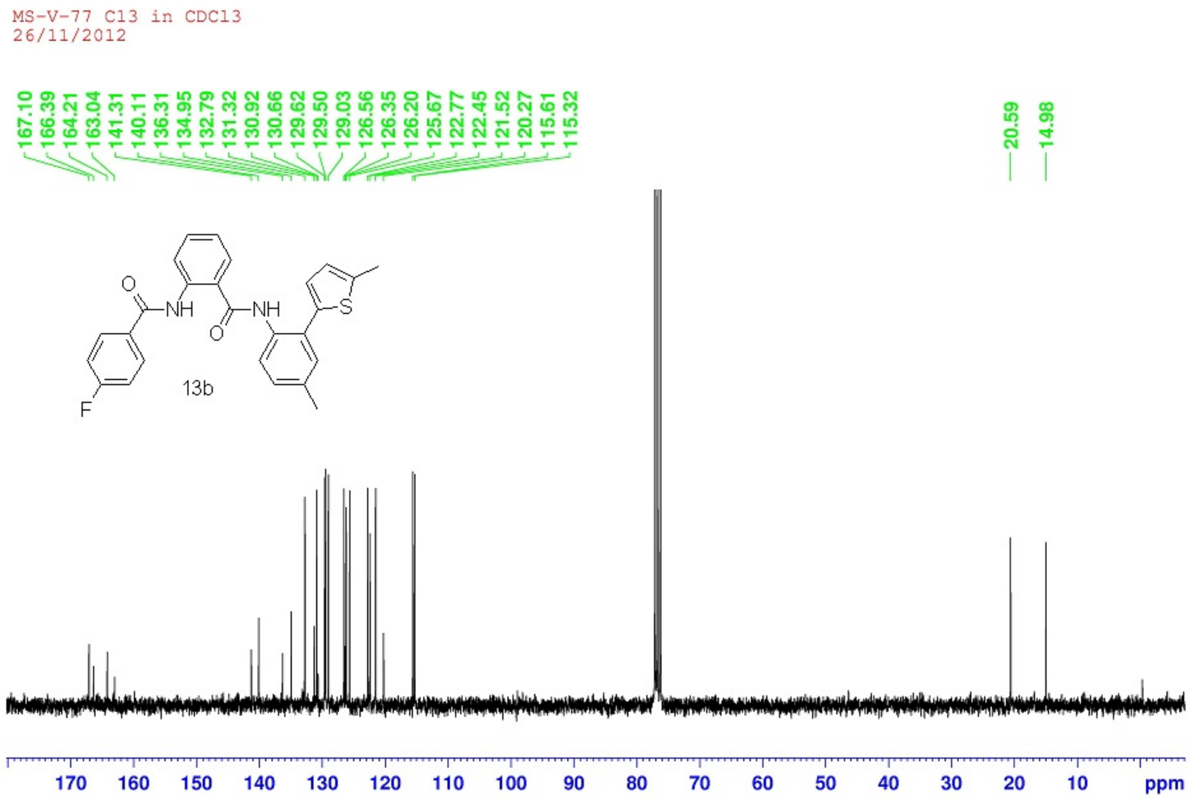

2-(4-fluorobenzamido)-*N*-(4-methyl-2-(pyridin-4-yl)phenyl)benzamide (13c)
HRMS

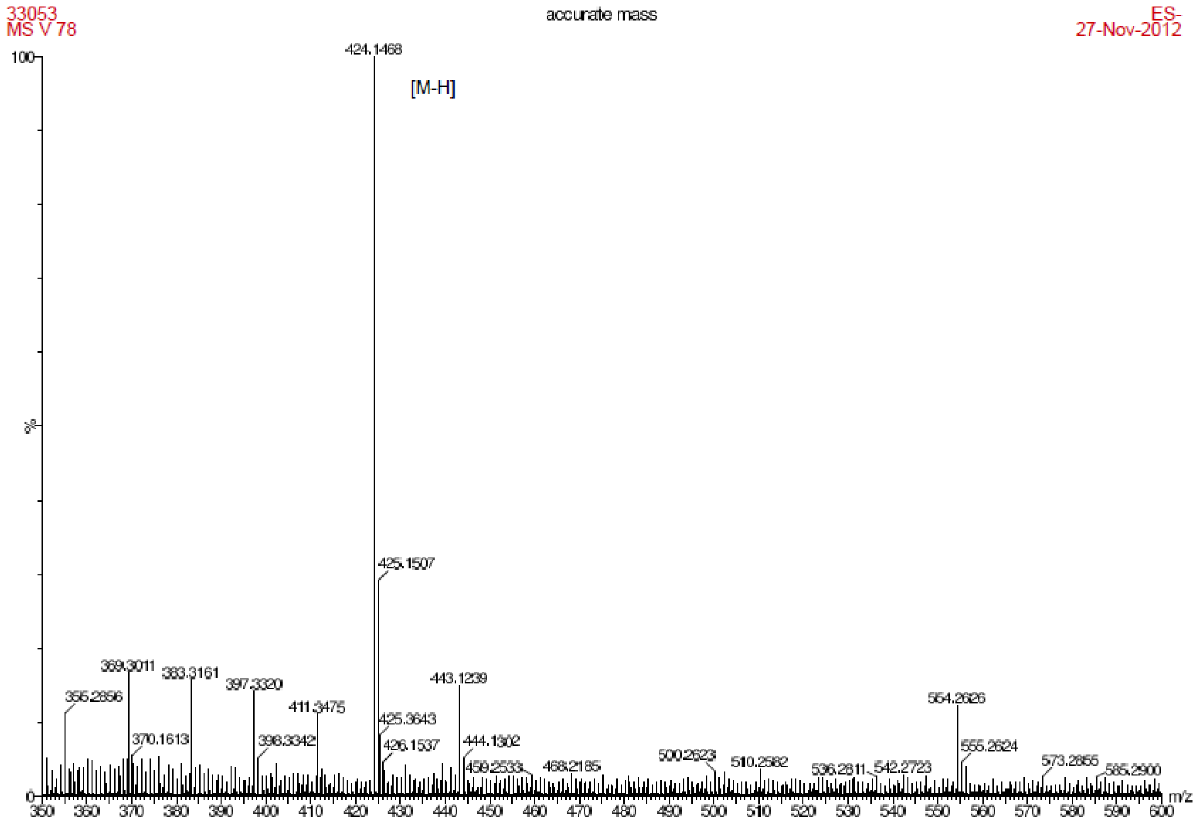

^{1}H NMR (300 MHz, $CDCl_3$)

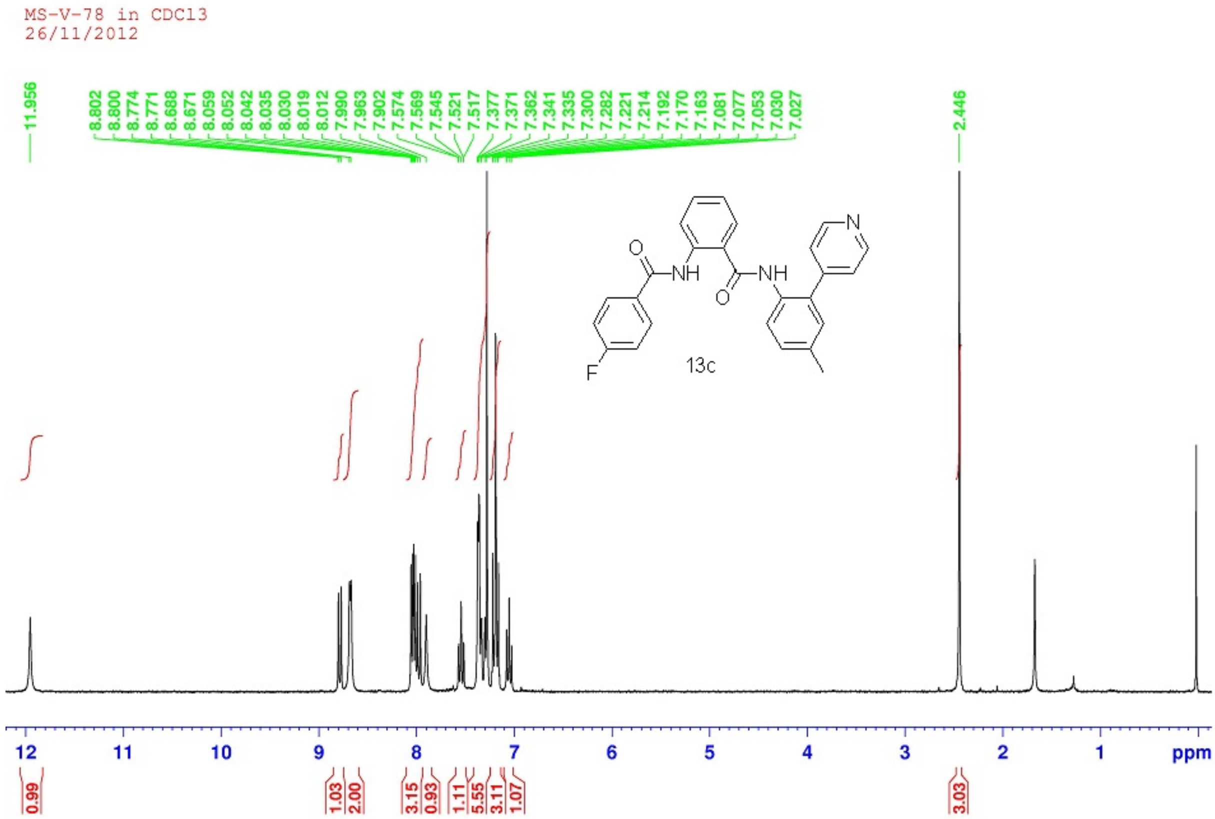

^{13}C NMR (75 MHz, $CDCl_3$)

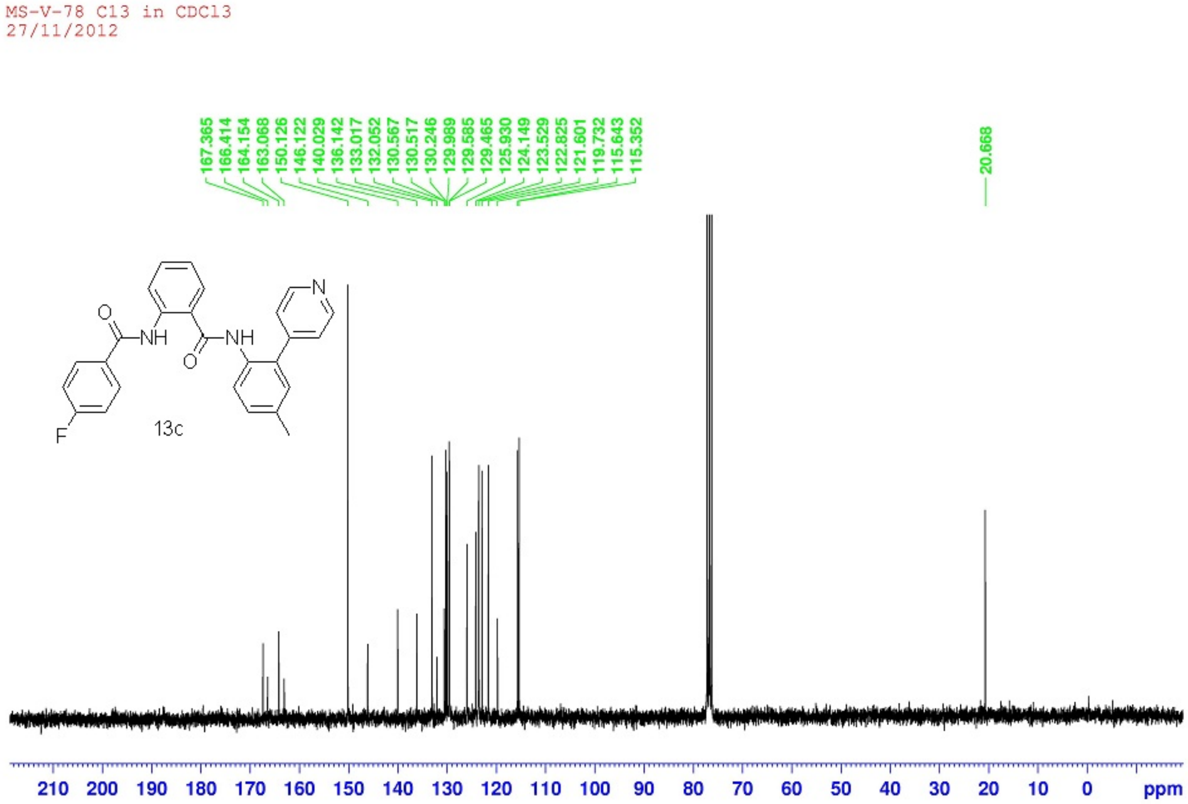

2-(4-fluorobenzamido)-*N*-(4-methyl-2-(pyridin-3-yl)phenyl)benzamide (13d)

HRMS

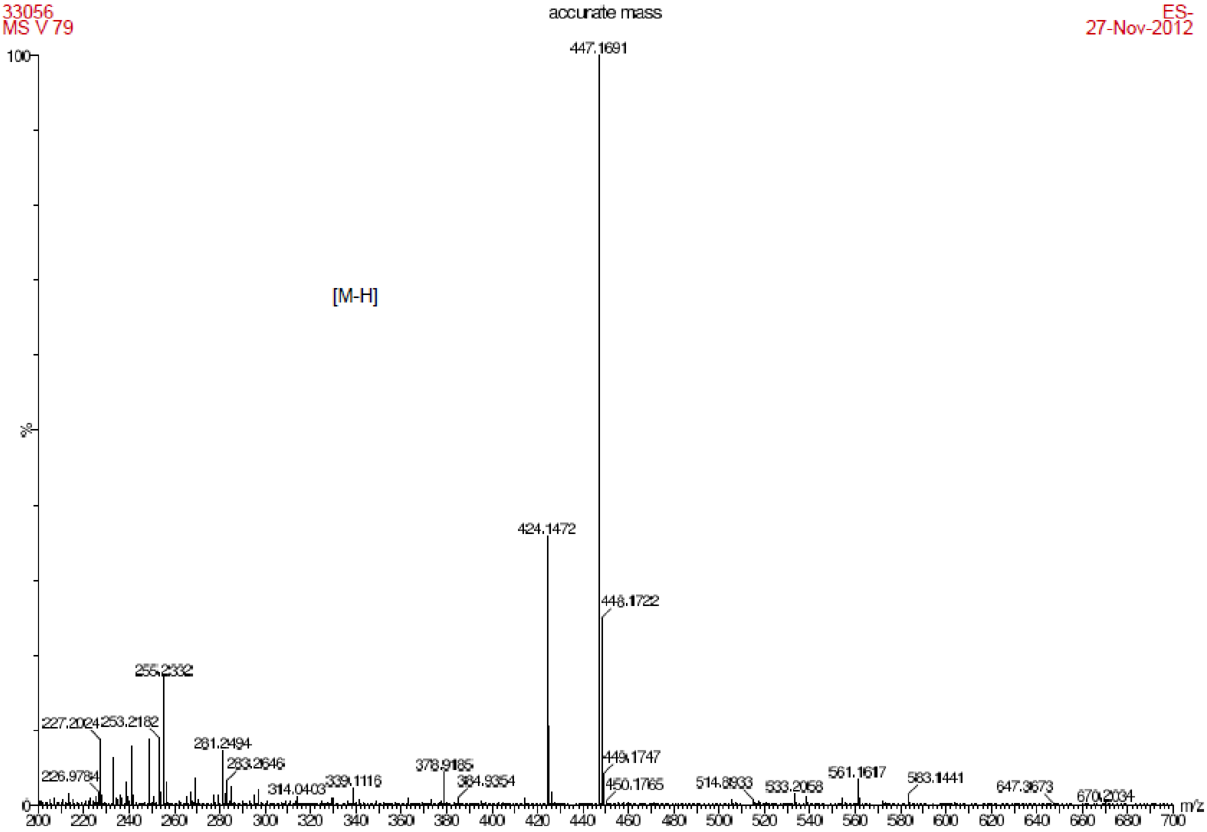

^{1}H NMR (300 MHz, $CDCl_3$)

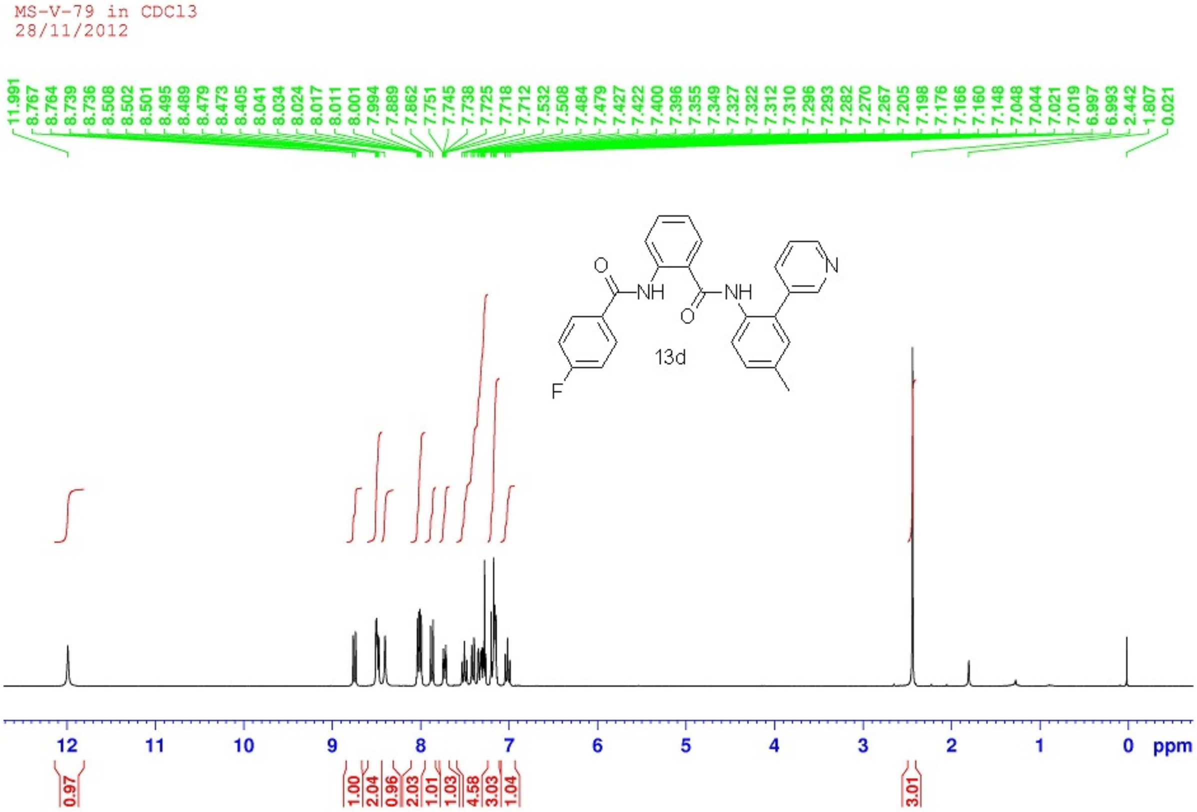

^{13}C NMR (75 MHz, DMSO)

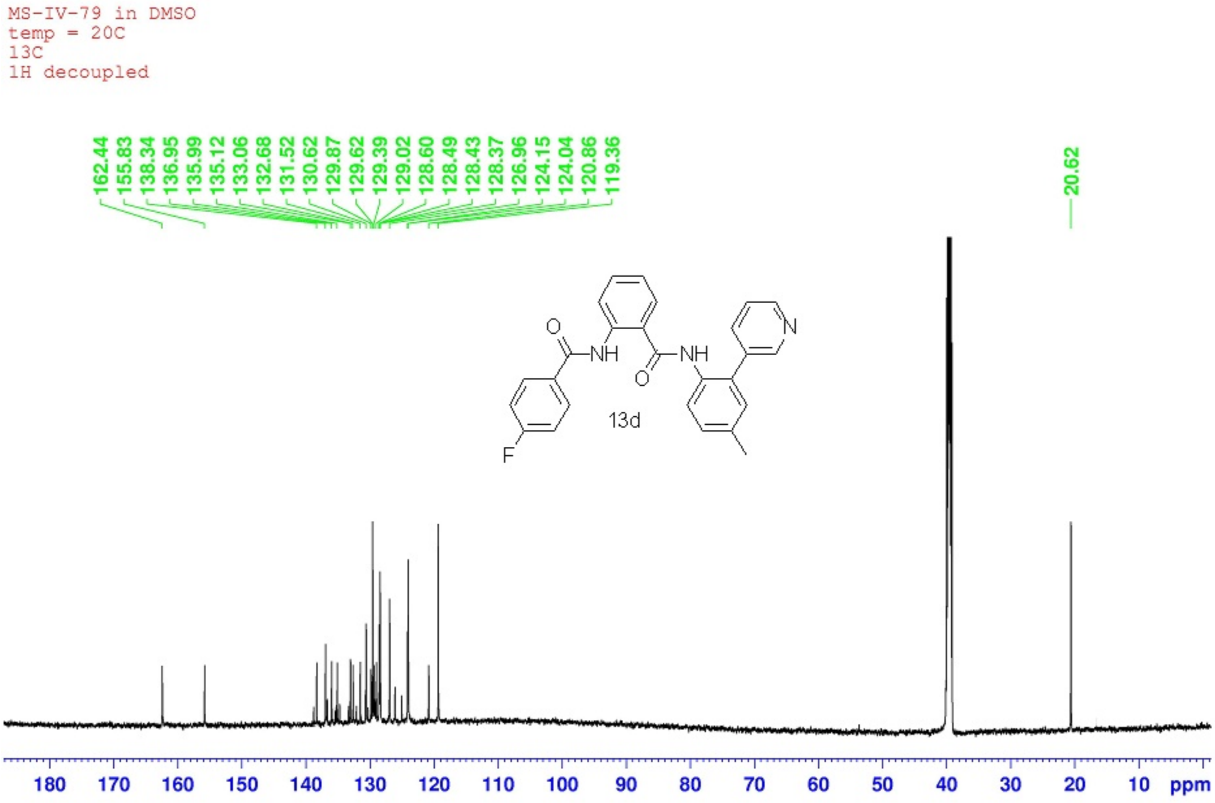

Supplement: Supplementary file 1 [file mmc1.pdf]
